# RNA Structure Duplication in the Dengue Virus 3′ UTR: Redundancy or Host Specificity?

**DOI:** 10.1128/mBio.02506-18

**Published:** 2019-01-08

**Authors:** Luana de Borba, Sergio M. Villordo, Franco L. Marsico, Juan M. Carballeda, Claudia V. Filomatori, Leopoldo G. Gebhard, Horacio M. Pallarés, Sebastian Lequime, Louis Lambrechts, Irma Sánchez Vargas, Carol D. Blair, Andrea V. Gamarnik

**Affiliations:** a Fundación Instituto Leloir-CONICET, Buenos Aires, Argentina; b Arthropod-borne and Infectious Diseases Laboratory, Department of Microbiology, Immunology and Pathology, Colorado State University, Fort Collins, Colorado, USA; c Insect-Virus Interactions Group, Department of Genomes and Genetics, Institut Pasteur, Paris, France; d Evolutionary Genomics, Modeling and Health, Unité Mixte de Recherche 2000, Centre National de la Recherche Scientifique, Paris, France; e KU Leuven Department of Microbiology and Immunology, Rega Institute, Laboratory of Clinical and Epidemiological Virology, Leuven, Belgium; University of Pittsburgh School of Medicine; University of Texas Medical Branch Galveston; Stanford University School of Medicine

**Keywords:** RNA virus, RNA virus evolution, RNA-RNA interactions, dengue virus, flavivirus, host adaptation, viral RNA structures

## Abstract

Flaviviruses include a diverse group of medically important viruses that cycle between mosquitoes and humans. During this natural process of switching hosts, each species imposes different selective forces on the viral population. Using dengue virus (DENV) as model, we found that paralogous RNA structures originating from duplications in the viral 3′ untranslated region (UTR) are under different selective pressures in the two hosts. These RNA structures, known as dumbbells (DB1 and DB2), were originally proposed to be enhancers of viral replication. Analysis of viruses obtained from infected mosquitoes showed selection of mutations that mapped in DB2. Recombinant viruses carrying the identified variations confirmed that these mutations greatly increase viral replication in mosquito cells, with low or no impact in human cells. Use of viruses lacking each of the DB structures revealed opposite viral phenotypes. While deletion of DB1 reduced viral replication about 10-fold, viruses lacking DB2 displayed a great increase of fitness in mosquitoes, confirming a functional diversification of these similar RNA elements. Mechanistic analysis indicated that DB1 and DB2 differentially modulate viral genome cyclization and RNA replication. We found that a pseudoknot formed within DB2 competes with long-range RNA-RNA interactions that are necessary for minus-strand RNA synthesis. Our results support a model in which a functional diversification of duplicated RNA elements in the viral 3′ UTR is driven by host-specific requirements. This study provides new ideas for understanding molecular aspects of the evolution of RNA viruses that naturally jump between different species.

## Abstract (precis)

Flaviviruses constitute the most relevant group of arthropod-transmitted viruses, including important human pathogens such as the dengue, Zika, yellow fever, and West Nile viruses. The natural alternation of these viruses between vertebrate and invertebrate hosts shapes the viral genome population, which leads to selection of different viral variants with potential implications for epidemiological fitness and pathogenesis. However, the selective forces and mechanisms acting on the viral RNA during host adaptation are still largely unknown. Here, we found that two almost identical tandem RNA structures present at the viral 3′ untranslated region are under different selective pressures in the two hosts. Mechanistic studies indicated that the two RNA elements, known as dumbbells, contain sequences that overlap essential RNA cyclization elements involved in viral RNA synthesis. The data support a model in which the duplicated RNA structures differentially evolved to accommodate distinct functions for viral replication in the two hosts.

## INTRODUCTION

The Flavivirus genus includes a large number of emerging and reemerging human pathogens that are transmitted by arthropods, including dengue (DENV), Zika (ZIKV), yellow fever (YFV), and West Nile (WNV) viruses. Dengue is the most prevalent arthropod-borne viral disease around the world. It is endemic in more than 100 countries, with about 390 million infections each year (1). In 2016, Latin America faced the worst DENV and ZIKV epidemics, and since 2017, YFV became, once more, a threat for South America, despite the existence of an effective live attenuated vaccine (2).

The DENV genome is a single-stranded RNA molecule of positive polarity that contains a great deal of information in RNA structures that function as signals to enhance, suppress, or promote viral replication (for review, see reference 3). Natural sequence variations in these RNA structures can be determinants for viral epidemiological fitness, pathogenesis, host adaptation, and transmission between mosquitoes and humans (4–10). Although a great deal has been learned in the last decades about how these RNA signals function during flavivirus infections, little is known about their molecular mechanisms of action.

The DENV 5' untranslated region (UTR) includes two essential elements for genome replication: (i) the promoter for RNA synthesis, known as stem-loop A (SLA), and (ii) cyclization sequences that mediate long-range RNA-RNA interactions (11). The general organization of the DENV 3′ UTR is similar to that in other flaviviruses, containing essential elements for viral replication, and accessory RNA structures that participate in both modulating viral processes and controlling host antiviral responses (for review, see reference 12). An intriguing feature of the 3′ UTR of flavivirus genomes is the evolutionary conservation of sequence repeats and duplicated RNA structures (13, 14). In the case of DENV, the 3′ UTR contains two almost identical stem-loop structures (SLI and SLII), two similar dumbbell elements (DB1 and DB2), an essential small hairpin (sHP), and the 3′ stem-loop (3′ SL) common to all flaviviruses (15–21). The two pairs of duplicated RNA elements (SLI-SLII and DB1-DB2) acquire stable secondary structures, including pseudoknot (PK) interactions that have the ability to stall genome degradation (22, 23). In DENV infections, SLI and SLII are responsible for the generation and accumulation of noncoding viral RNAs as products of incomplete genome degradation, known as subgenomic flavivirus RNAs (sfRNAs) (8, 24). These sfRNAs play important roles counteracting antiviral responses in mosquito and human cells (25–28; for review, see references 19, 29, and 30).

The biological significance of maintaining two almost identical RNA structures in the 3′ UTR of flaviviruses is enigmatic. Redundant functions, as replication enhancers, were proposed for the two DB elements (31, 32). However, predictions of distinct folding intermediates of DB1 and DB2 suggested possible distinct functions (32). Interestingly, an extensive pan-flavivirus sequence analysis proposed that repeated motifs and duplications were associated with the viral evolutionary process of acquiring multiple hosts (for review, see reference 33). More recently, experimental data obtained studying the function of the duplicated SLs in DENV supported a model in which RNA duplication allows the virus to accommodate mutations beneficial in one host (mosquitoes) but deleterious in the other (humans), conferring robustness during host switching (5).

Previous studies have determined other requirements for different viral RNA structures for DENV replication in the two hosts (5, 34–36). These observations raised important questions regarding the mechanisms by which viral RNA structures work in mosquitoes and humans and about the implications of the genetic variations in the 3′ UTR in host adaptation, transmission, and pathogenesis. In this regard, in regions of endemicity and hyperendemicity, cocirculation of different DENV genotypes or serotypes can lead to strain displacements, often associated with different transmission kinetics and clinical outcomes (37–40), highlighting the relevance of understanding the reasons for genome sequence variability in natural settings. Interestingly, sequence variability at the 3' UTR of DENV isolates was recently correlated with distinct epidemiological fitness (4).

Here, using DENV as a model, we found that each of the duplicated DB RNA structures in the viral 3' UTR is under different selective pressures in adult mosquitoes. Sequence analysis of DENV RNA populations showed adaptive mutations mapping in only one of the two DB structures. Using recombinant viruses with the identified mutations, a great advantage for viral replication in mosquito cells was associated with alterations in the DB2 sequence. Mechanistic analysis indicated that the two DB elements differentially regulate genome cyclization, which is a conformation required for viral RNA synthesis. We previously showed that sequences within DB1 hybridize with a region present in the capsid coding sequence, promoting genome cyclization (34). Now, we present experimental evidence showing a competition between a sequence at the top loop of DB2 and a core cyclization element for tertiary interactions. Our data support a model in which superimposed sequences involved in local RNA structures and long-range RNA-RNA interactions regulate different viral RNA conformations that are relevant for infection. Although we experimentally worked with DENV, these overlapping RNA signals were found in the genomes of all mosquito-borne flaviviruses (MBFV), suggesting a widely conserved mechanism. We conclude that the duplicated DB structures present in the DENV 3' UTR have redundant activities in both hosts but evolved divergent sequences with host specific functions. These results provide new ideas that shed light on flavivirus evolution and host adaptation.

## RESULTS

### DENV adaptive mutations associated with mosquito infection.

**FIG 1:**
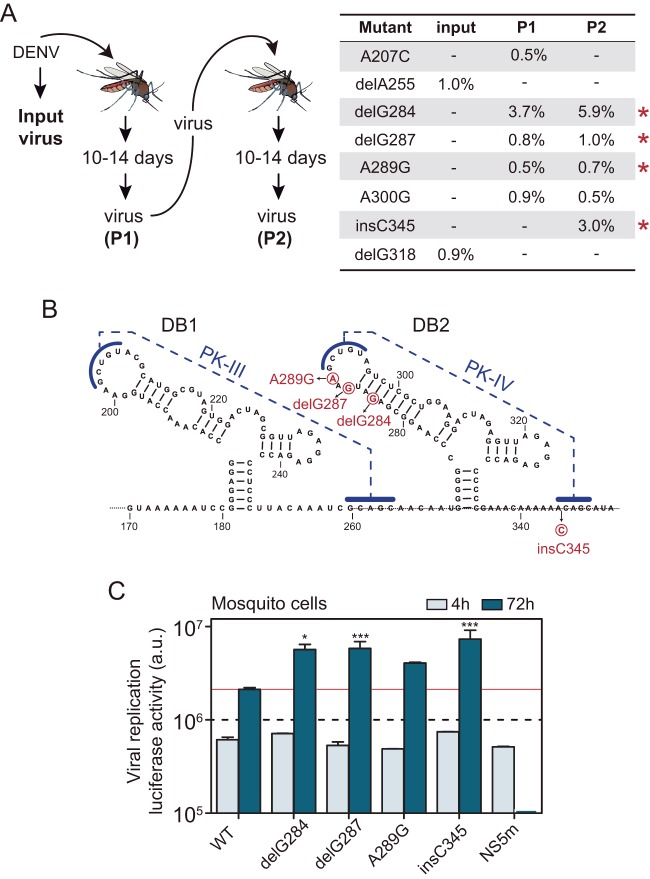
DENV adaptive mutations associated with replication in mosquitoes. (A) (Left) Experimental design of DENV adaptation in adult mosquitoes. (Right) Mutations identified in the input and passages P1 and P2. Red stars indicate mutations that showed increased frequencies from P1 to P2. (B) Location of adaptive mutations in the viral 3′ UTR. The secondary structures of the two DBs are shown. Nucleotide variations are indicated in red. (C) Replication of recombinant reporter DENVs carrying the identified mutations. The plot shows viral replication measured by luciferase activity as a function of time post-RNA transfection into mosquito cells. The luciferase values (in arbitrary units [a.u.]) are means ± standard deviations (representative results of three independent experiments). *, *P* < 0.05; ***, *P* < 0.001.

To study the significance of DENV 3'-UTR variability in host adaptation, we sequenced viral RNA populations obtained from infected Aedes aegypti and Aedes albopictus mosquitoes. Extracts from pools of five A. albopictus mosquitoes 14 days postinfection (dpi) were used for titrations and to infect fresh mosquitoes (passage 1 [P1]) (Fig. 1A). Viruses obtained from the second passage were harvested at 14 dpi (P2). Genomes from the original stock (input) and viral genomes obtained in P1 and P2 from two independent experiments were used for 3'-UTR amplicon sequencing by next-generation sequencing (NGS) and cloning. We searched for mutations that were absent in the input population but showed increased frequencies from P1 to P2. Four mutations with increased frequencies were detected above the threshold of 0.5% of the population (Fig. 1A). These mutations (delG284, delG287, A289G, and InsC345) mapped in the DB2 structure (Fig. 1B). Although mutations were also detected in DB1, no positive selection on any of them was observed from P1 to P2 (Fig. 1A). Because there is a special interest in understanding the function of RNA structure duplication in flavivirus genomes, and a deletion specifically introduced into DB2 results in attenuation of promising DENV vaccine candidates (41, 42), we further investigated the biological significance of these nucleotide variations.

To evaluate the relevance of the variants selected in mosquitoes, recombinant viruses carrying the identified point mutations were designed in the context of a reporter DENV infectious cDNA clone, and replication was tested in a mosquito cell line. Mutant viral RNAs were transfected along with a wild-type (WT) RNA and a replication-defective control with a mutation in the polymerase NS5. Viral replication was assessed by measuring luciferase activity as a function of time (Fig. 1C). The virus with the insertion InsC345, which debilitates formation of the PK that stabilizes DB2 (Fig. 1B), showed a 4-fold increase in luciferase expression with respect to WT virus. The mutants with the other three mutations, delG284, delG287, and A289G, which alter DB loop and DB stability, had between 2- and 3-fold increased viral replication with respect to WT.

We conclude that mutations positively selected in adult mosquitoes that affect DB2 structure result in enhanced RNA replication in mosquito cells. Therefore, we used this model to understand mechanistic aspects and functions of the adaptive mutations.

### Distinct evolution of the two DB elements present at the 3′ UTR of DENV isolates.

**FIG 2:**
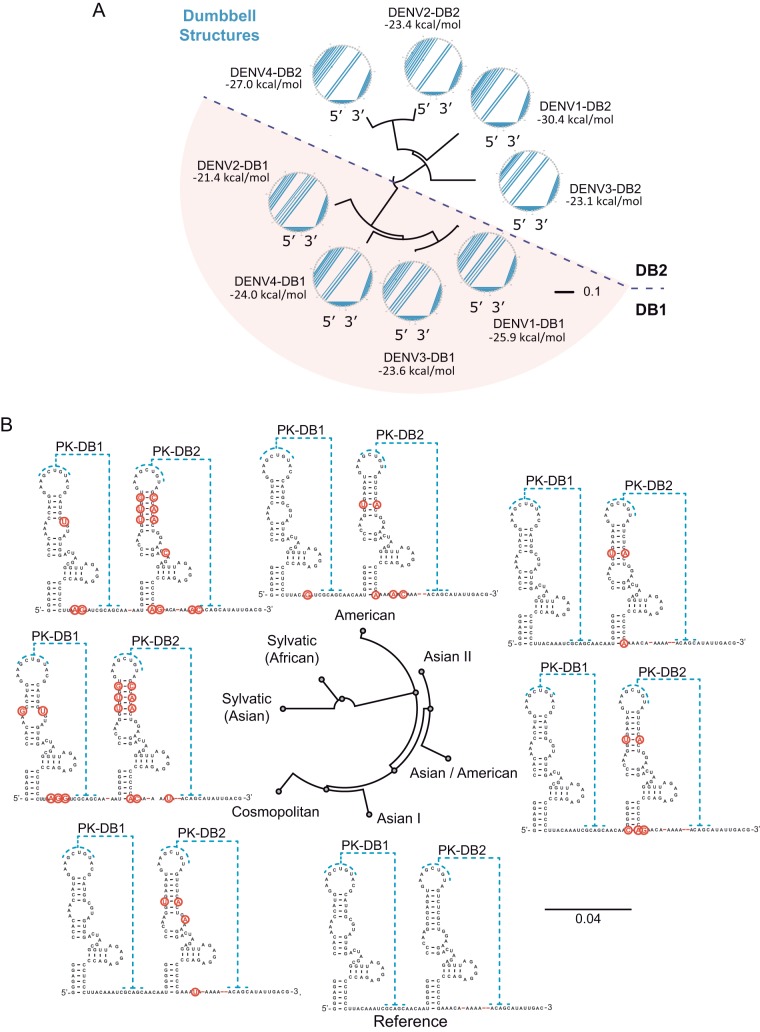
Sequence and structure analysis of the two DB elements. (A) Fan dendrogram indicating the distance of DB structures from different DENV serotypes. The corresponding circle plot for each sequence is shown with arcs denoting base pairs. (B) Comparison of the natural sequence variability of DB1 and DB2 for different DENV2 genotypes. Asian I genotype was used as a reference. Variability of the DB sequences is indicated in red. The size bar indicates number of nucleotide substitution per site.

Based on the selection of advantageous mutations in mosquitoes in one of the two DBs, we examined the evolutionary relationship between the duplicated RNA structures in different DENV serotypes. To this end, we employed a tree alignment model algorithm, RNAforester (43), to compute pairwise alignments between DB secondary structures folded according to previous biochemical and covariation analyses. Results from individual comparisons were used to generate a similarity matrix that was plotted in dendrograms using UPGMA (the unweighted pair group method using average linkages) (Fig. 2A). This method allows determination of the relative similarity between two models of secondary structures by estimating the number of changes necessary to convert one structure to another. Thus, it was possible to determine how similar two structures are, independent of their sequence identity. The results indicate that the DB1 elements from different DENV serotypes are more alike between them than DB1 and DB2 from the same serotype (Fig. 2A). The same observation was valid for DB2, supporting a divergent evolutionary path and specialization of each structure after duplication.

The sequence conservation of the two DBs was also analyzed using different genotypes of all four DENV serotypes. A conservation analysis per site for each structure showed 75% and 63% identity for DB1 and DB2, respectively. For example, in the case of DENV2, a higher sequence variability of DB2 was observed when genotypes from different geographic regions were compared (Fig. 2B). In this analysis, an Asian I genotype was used as a reference and compared to Cosmopolitan, American, Asian-American, Asian II, Sylvatic African, and Sylvatic Asian genotypes (Fig. 2B). The observation that DB2 displays more sequence variation than DB1 within the same serotype suggests that the two paralogous RNA elements are under different selective pressures in nature, which could be associated with the selection observed in mosquitoes (Fig. 1A).

### Contrasting functions of the two DB RNA structures during DENV replication.

**FIG 3:**
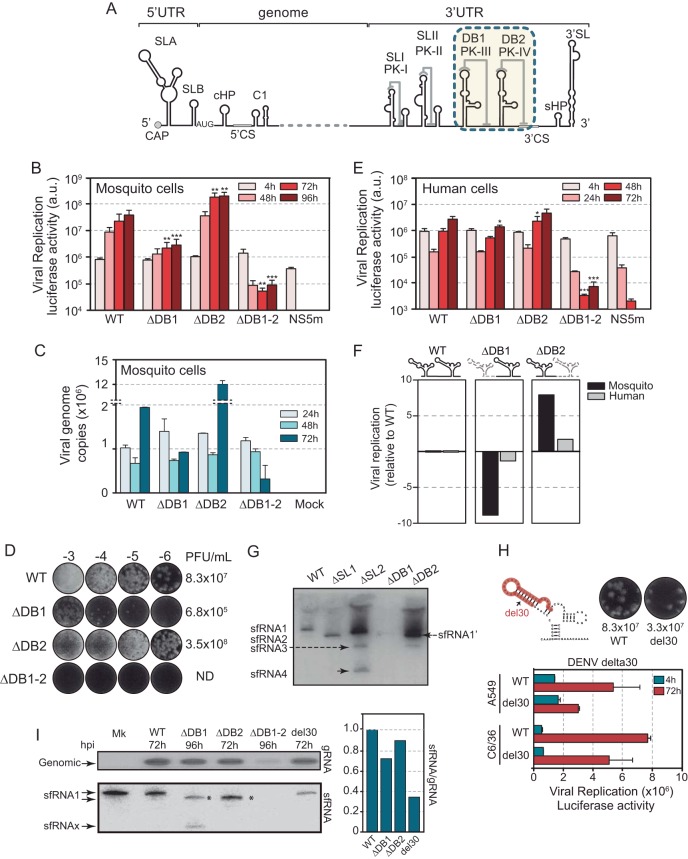
Divergent functions of the two DB structures. (A) Schematic representation of the DENV genome showing the RNA structures in the 5′ and 3′ UTRs. (B and E) Replication of DENV reporter mutants carrying deletions of individual DBs (ΔDB1 and ΔDB1) or both (ΔDB1-2) in mosquito and human cells, respectively. Plots show *Renilla* luciferase activity as a function of time post-RNA transfection. (C and D) Replication of WT DENV or mutants with DB deletions, using the original infectious DENV2 16681 clone. Luciferase values are means ± standard deviations (representative results from three independent experiments). (C) RNA accumulation. The plot indicates number of viral genome copies as a function of time in mosquito cells. (D) Production of infectious particles is expressed as PFU/ml. (F) Impact of the deletion of each DB on viral replication in mosquito and human cells. (G) Accumulation of sfRNAs in mosquito cells. Shown is a Northern blot using a radiolabeled probe that recognizes the viral RNA 3′ UTR. Different sfRNA species are indicated on the left (sfRNA1-4 and sfRNA1′). (H) Schematic representation of the del30 deleted sequence in DB2 and infectious particles produced in mosquito cells (top) and replication of the DENV del30 reporter mutant in mosquito and human cells along with the WT. Luciferase values are means ± standard deviations (representative results from three independent experiments). (I) Accumulation of gRNA (genomic RNA) and sfRNAs in mosquito cells. Shown is a Northern blot using a radiolabeled probe that recognizes the viral RNA 3′ UTR for sfRNA and NS2B for gRNA. Different sfRNA species separated by PAGE are indicated on the left. Asterisks indicate different sizes of sfRNA1 because of the DB deletions. The plot on the right shows the ratio of sfRNA to gRNA for each mutant.

The finding that DB2 displays more natural sequence variation than DB1 in different DENV isolates and that adaptive mutations in mosquitoes mapped to DB2 suggested a possible diversification of functions of the duplicated RNA structures. To examine this possibility, we designed deletions of each or both DB structures in mutated genomes and evaluated viral replication in mosquito cells. Viral RNA from the mutants (DENVΔDB1, DENVΔDB2, and DENVΔDB1-2 [Fig. 3A]) or controls was transfected into mosquito cells, and replication was assessed as a function of time. DENVΔDB1 showed about 10-fold reduction in luciferase expression by 72 hpi (Fig. 3B), in agreement with previous observations (34). However, deletion of DB2 resulted in about 8-fold increased viral replication with respect to WT virus. These results were unexpected because until now the DB elements were considered enhancers of viral replication. In addition, deletion of both DB structures (ΔDB1-2) resulted in a near-lethal phenotype, with a reduction of viral replication of more than 100-fold (Fig. 3B), indicating that although deletion of DB2 enhanced replication, the lack of both DB structures showed a synergistic negative effect on viral fitness. These results indicate that the duplicated RNA elements bear opposite functions during viral infection in the mosquito, but certain structural elements are redundant, and at least one copy is necessary (essential) for viral replication. The phenotypes were confirmed by deleting each of the DB elements in a DENV2 infectious clone and evaluating viral RNA accumulation (Fig. 3C) and production of infectious particles (Fig. 3D). The results indicate that deletions of DB1 or DB2 display opposite effects on DENV RNA accumulation in mosquito cells and deletion of both RNA structures greatly reduced viral RNA synthesis (Fig. 3C and D).

To evaluate whether the phenotype observed with DENVs carrying DB deletions was host specific, the mutant viral RNAs and controls were transfected into A549 human cells. The deletion mutants DENVΔDB1 and DENVΔDB2 showed a subtle but significant reduction or increase of viral replication, respectively (Fig. 3E). Although deletion of each DB impacted replication in the same manner as that observed in mosquito cells, the magnitudes were markedly different. The deletion of both DBs (ΔDB1-2) reduced viral replication in human cells about 400-fold, supporting the idea of the requirement for at least one copy for viral replication. Together, the results indicate a diversification of functions of the two homologous RNA structures that is host specific in magnitude (Fig. 3F). In addition, at least one copy of a crucial *cis*-acting signal that is duplicated appears to be required in both hosts.

The duplicated SL and DB structures in the 3' UTR of flaviviruses are capable of stalling genome degradation by the 5′-exonuclease XRN1 that results in sfRNA accumulation (22). To evaluate whether deletion of DB2 could result in alteration of sfRNA accumulation, we analyzed their production in transfected cells by Northern blotting. In this regard, as previously reported, the replication of viruses with SL2 deletions resulted in the generation of shorter species of sfRNAs (Fig. 3G), which was associated with increased fitness in mosquitoes but a reduction of fitness in human cells (5). DENVΔDB2 generated a large amount of the expected sfRNA1′, which differed in size from the WT sfRNA1 by 70 nucleotides (nt), due to the deletion (Fig. 3G). DENVΔDB1 produced a similar sfRNA1', but in very small amounts associated with lower levels of viral genome accumulation (Fig. 3G). The results suggest that viruses that lack either of the DB elements retain the ability to halt genome degradation at SLI.

A deletion within DB2 is an attenuating mutation of a live vaccine candidate, DENV delta30 (del30), which is currently in advanced clinical trials (42). Because our observations support the idea that deletion or specific mutations in this structure enhance DENV2 replication in mosquitoes, we examined the replication of DENV2 del30 in mosquito and human cells. Full-length RNAs of the WT and del30 mutant were transfected, and viral replication was evaluated. Interestingly, the del30 mutated genome showed about 2-fold reduction of replication with respect to WT virus in both cell types, measured by reporter virus replication and infectious particles produced in mosquito cells (Fig. 3H). Because del30 replication in human cells was shown to produce a defective ratio of sfRNAs to viral genome (44), the ratio of these viral RNAs were analyzed in infected mosquito cells using the del30 mutant and viruses with the complete deletion of each of the DB elements. Due to the different replication kinetics of the DB mutants, cells were infected with different multiplicities of infection (MOI) and harvested at different times after infection to obtain sufficient amounts of viral RNA, and the ratio of sfRNA to genomic RNA (gRNA) was estimated for each virus using specific radiolabeled probes (Fig. 3I). This analysis indicated that del30 displays a significant reduction of sfRNA production in the infected mosquito cell. In addition, a reduction of this ratio and the detection of an uncharacterized small RNA (sfRNAx) were observed with the ΔDB1 mutant (Fig. 3I).

The results support the idea that, although certain mutations or complete deletion of DB2 result in increased viral replication in mosquito cells, del30 in the context of DENV2 RNA is slightly attenuated in these cells, as had been observed earlier for vaccine candidates in *Aedes* sp. mosquitoes (45, 46).

### Mutually exclusive structures due to overlapping sequences in DB2-PK and 3′ CS modulate viral RNA replication.

**FIG 4:**
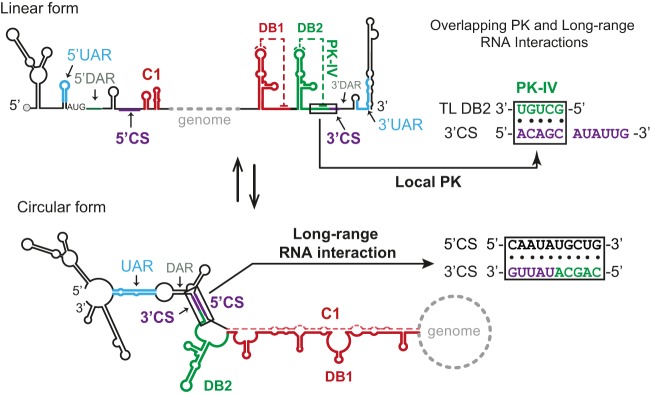
Mutually exclusive local and long-range RNA interactions. Shown is a schematic representation of linear and circular conformations of the DENV genome. Cyclization elements are indicated: 5′ and 3′ UAR (blue), 5′ and 3′ CS (purple), 5′ and 3′ DAR (gray), and C1-DB1 (red) in both structures (top linear and bottom circular). DB2 is indicated in green. In boxes, the sequences of pseudoknot (PK-IV) that overlap the 3′ CS are shown for both forms—linear (PK-IV) and circular (5′-3′ CS).

To investigate the mechanism by which DB2 modulates viral replication, we analyzed the elements known to govern DENV RNA synthesis. The sequence at the top loop of DB2 forms a PK that stabilizes the RNA structure, but this PK includes nucleotides within the essential 3′ conserved sequence (3′ CS) (Fig. 4). This CS is complementary to the 5′ CS, and the long-range RNA-RNA interaction 5′-3′ CS is an essential cyclization element for DENV RNA replication (31, 47). The two secondary structures, local DB2-PK and long-range 5′-3′ CS, are mutually exclusive: one is present in the linear form of the viral genome and the other in the circular form, respectively (Fig. 4). Thus, we hypothesize that the DB2-PK structure modulates genome cyclization.

**FIG 5:**
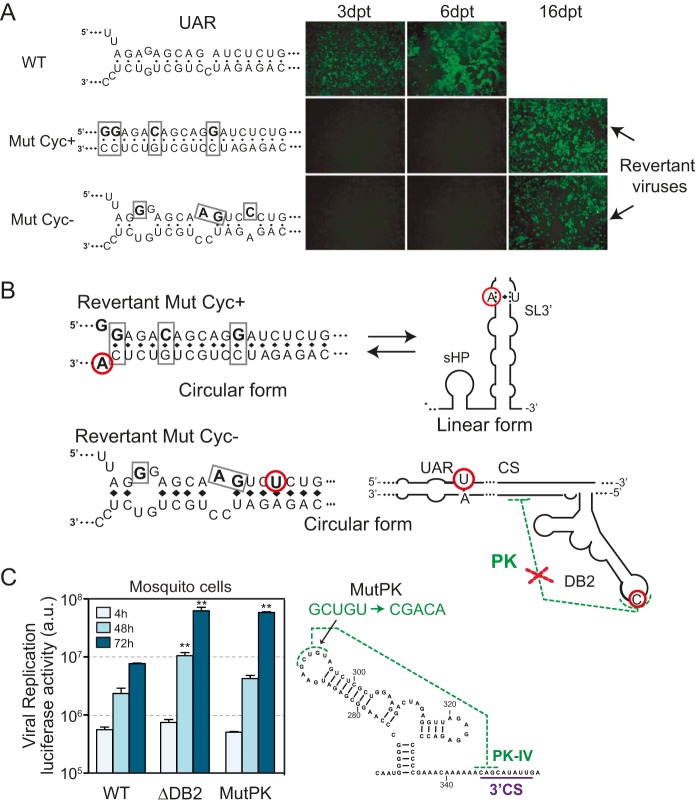
DB2 sequence negatively regulates genome cyclization. (A) Immunofluorescence of mosquito cells transfected with RNA from WT and recombinant viruses carrying the mutations Mut Cyc^+^ and Mut Cyc^−^ in the 5′-3′ UAR sequence, as indicated. (B) Schematic representation of mutations identified in the recovered viruses. In the case of Mut Cyc^+^, the location of the mutations in linear and circular forms of the RNA is shown. In the case of Mut Cyc^−^, the location of mutations in the circular form is shown, indicating both the complementary region and disruption of DB2 PK. (C) (Left) Design of mutation to disrupt the DB2 PK (MutPK). (Right) Replication of DENV reporter mutants in mosquito cells. Luciferase activity measurements as a function of time are shown for each case. The luciferase values are means ± standard deviations (representative results from three independent experiments). **, *P* < 0.01.

We have previously shown a tight regulation of the equilibrium between circular and linear forms of the genome for viral infectivity and analyzed hundreds of DENV mutants that disrupted/restored genome cyclization in mammalian cells (48). To examine the possible involvement of DB elements in modulating cyclization in mosquito cells, we tested mutant DENVs in which the equilibrium between linear and circular forms of the genome was drastically displaced, expecting a stronger selective pressure than in human cells. The mutants used, Mut Cyc^+^ or Mut Cyc^−^, increase or decrease complementarity between the ends of the viral genome, respectively (Fig. 5A). Viral RNAs were transfected into mosquito cells, and propagation was evaluated by immunofluorescence as a function of time. Both Mut Cyc^+^ and Mut Cyc^−^ RNAs were replication impaired; however, 16 days after transfection, replicating viruses were rescued from cell cultures (Fig. 5A). For sequencing the genomes of recovered viruses, RNA was isolated, the viral genomes were circularized by end-to-end ligation after decapping, and both the 5′ and 3′ ends of the same genome were sequenced in single amplicons. The main revertant virus from cells transfected with Mut Cyc^+^ had a mutation that (i) reduced by 1 bp the upstream AUG region (UAR) complementarity (reducing cyclization) and (ii) increased stability of the 3′ SL by 1 bp (increasing stability of the linear form) (Fig. 5B). Interestingly, revertant virus from Mut Cyc^−^-transfected cells had two point mutations that (i) reformed a base pair in UAR, increasing cyclization, and (ii) introduced one mutation in the top loop of DB2, disrupting the PK interaction that competes with cyclization (Fig. 5B). This pseudorevertant virus with a mutation disrupting DB2-PK that compensates for reduced cyclization was particularly informative. This observation links a local RNA structure with long-range interactions.

In order to confirm our hypothesis that formation of the PK of DB2 competes with genome cyclization and negatively regulates viral RNA replication, a recombinant virus was designed impairing formation of the PK (Mut-PK [Fig. 5C]). This mutant RNA was transfected along with controls into mosquito cells, and replication was evaluated as a function of time. Disruption of the PK resulted in about 10-fold increased viral replication, showing a similar phenotype to that observed with the DENVΔDB2 mutant (Fig. 5C). These results support a role of the DB2-PK on genome cyclization, providing a possible explanation for the selection of mutations in mosquitoes that destabilize this structure.

### Mapping 3′-UTR mutations identified in a previous intrahost study with DENV1-infected mosquitoes.

**FIG 6:**
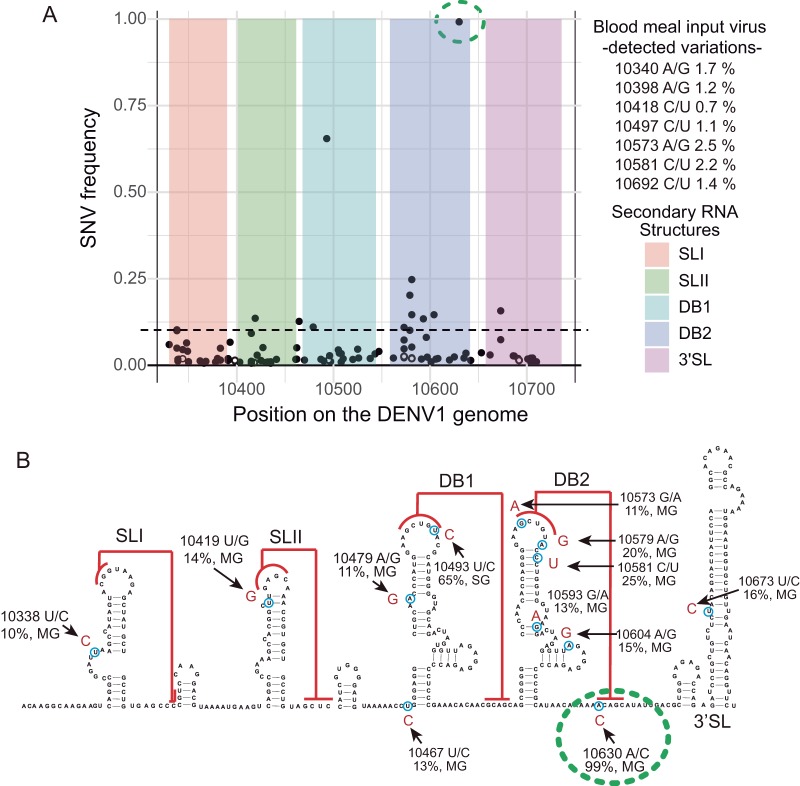
Mapping single nucleotide variations in the viral 3′ UTR from intrahost analysis in DENV1-infected mosquitoes (A) Distribution along the viral 3′ UTR of mutations identified in an intrahost analysis using *A. aegypti* mosquitoes. The plot represents the frequency of mutations identified indicated in the *y* axis and the position in a reference DENV1 genome (GenBank accession no. HG316481) shown in the *x* axis. Colors indicate each of the defined RNA structures along the 3′ UTR, as indicated on the right. Sequence variations detected in viruses included in the blood meal are also shown in the plot by open circles and on the right, indicating the position, nucleotide change, and frequency for each case. For SNVs detected in several samples, only the highest frequency is indicated. (B) Location of the mutations identified with frequencies above 10% on the secondary structure of the DENV1 3′ UTR. As a reference DENV1, GenBank accession no. HG316481 was used. Arrows indicate the position of the substitutions, including the frequency, the nucleotide change, and the source—midgut (MG) or salivary gland (SG). The green circle indicates a mutation found at a frequency of 99% that disrupts DB2-PK formation.

An intrahost diversity study in Aedes aegypti mosquitoes infected with DENV1 has recently been reported. This work investigated viral population expansions after the initial infection, shown to be randomly founded by a few tens of viruses (49). Deep sequencing of viral genomes obtained from midgut and salivary glands of individual mosquitoes at different time points postinfection identified a number of single nucleotide variants (SNVs). Here, we analyzed the original data focusing on viral 3′-UTR SNVs that reached frequencies above 10% in the mosquito organs. Eleven such SNVs were found and mapped to the predicted RNA structures (Fig. 6). In SLI, one mutation was detected in a variable element, while in SLII, the identified mutation disrupted the RNA structure. This observation is in agreement with previous host adaptation studies that showed increased fitness in mosquitoes of DENV with mutations disrupting specifically SLII (5). Within DBI and DBII elements, 2 and 6 mutations were identified, respectively. One mutation in DBI mapped to a bulge, and the other mapped to the top loop, maintaining or even enhancing PK interaction (a UG base pair was replaced by a CG) (Fig. 6B). In DBII structure, from the six mutations identified, three did not change the predicted RNA structure, one partially opened the stem, and the other two directly disrupted PK formation. These two substitutions occurred at each side of the PK, in the top loop and in the sequence downstream of DBII (Fig. 6B). Interestingly, this last mutation, located in the only nucleotide of the PK that is outside the 3′-CS sequence (position 10630 [Fig. 6B]), was almost fixed in the entire viral population in a midgut sample (99.2%), consistent with a replication advantage of a viral genome that released the competition between PK and long-range RNA-RNA interaction.

Although the adaptive nature of these mutations has not been directly tested, this study supports the concept that mutations in DBII disrupting PK interaction may provide a viral fitness advantage in the mosquito. It also shows that this type of variations can spontaneously arise *in vivo* and increase in frequency in the viral population in mosquitoes.

### Conserved overlapping RNA signals in MBFV genomes.

**FIG 7:**
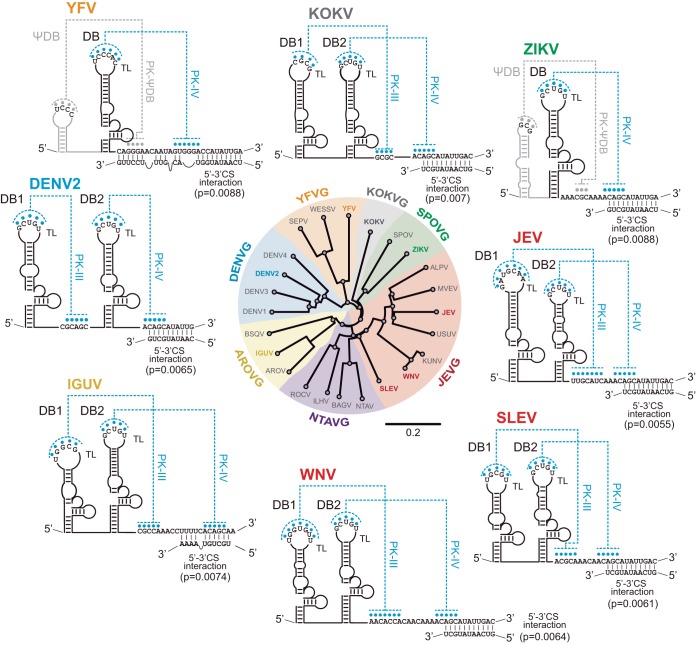
Conserved distribution of DB PK and cyclization elements in the genomes of mosquito-borne flaviviruses (MBFVs). Dumbbell structures of selected viruses from each subgroup of MBFV are shown. Locations and complementary bases that form PKs are indicated in blue. YFV and ZIKV form ΨDBs with PKs that are indicated in gray. Complementary sequences between the 5′ and 3′ ends of the genome that involve DB PKs are shown (5′-3′ interactions). Estimated *P* values show the reliability of indicated long-range RNA-RNA interactions, which were compared with a randomly sampled alignment. The distance tree was drawn using the neighbor-joining method of all complete genome sequences for each virus available in GenBank.

Taking into account that cyclization of the viral genome is a common feature of all flaviviruses, we also analyzed the relative distribution of the DB structures and cyclization signals in different flavivirus 3′ UTRs. MBFVs include a large number of human pathogens that are divided into seven groups: the DENV group (DENVG), Japanese encephalitis group (JEVG), YFV group (YFVG), Kokobera virus group (KOKVG), Aroa virus group (AROVG), Ntaya virus group (NTAVG), and Spondweni virus group (SPOVG) (50). The 3′ UTR of all these viruses bears a conserved DB structure, and in most, the structure is duplicated (Fig. 7). Exceptions are members of the YFVG and members of the SPOVG (including Zika virus). In these two cases, the single DB is preceded by a pseudo-DB (ΨDB, lacking conserved DB structural blocks), but forming a PK with sequences downstream of the DB structure (Fig. 7). An interesting feature in MBFV 3′ UTRs is the distinct distribution of the PKs that stabilize the two DB elements. In most cases, formation of both PKs results from pairing of sequences present in the top loops of the DBs with unstructured sequences located downstream of DB2. In contrast, in the four DENV serotypes, DB1 PK is formed with sequences located upstream of DB2 (Fig. 7). Interestingly, analysis of cyclization signals in each case indicates that, regardless of the location of the PKs, in all MBFVs analyzed, sequences involved in genome cyclization (3′ CS) overlap PK sequences that stabilize DB2 (Fig. 7). This conserved property shared among all MBFVs supports a regulatory role of overlapping sequences with contrasting functions in the viral 3′ UTRs. Special attributes were observed in RNA structures present in the YFV genome. In this case, both PK sequences (stabilizing ΨDB and DB structures) overlap cyclization elements, suggesting an additional function of the ΨDB element in regulating genome conformation of this particular virus.

## DISCUSSION

In these studies, we found that paralogous viral RNA structures originating from duplications in the 3′ UTR of flavivirus genomes are under different selective pressures in different hosts. The data support the hypothesis that duplicated DB elements, present in most MBFV genomes, conserve redundant functions, but also have evolved divergent host-specific activities that modulate viral RNA replication. Our work provides mechanistic details by which DB structures regulate viral genome conformation by either enhancing or competing with long-range RNA-RNA interactions that promote viral RNA synthesis. We propose a model in which different requirements for viral replication in mosquito and human cells may explain the conservation of duplicated RNA structures in flavivirus 3' UTRs.

Formation of two DB structures with PK interactions in the DENV genome was originally predicted (15–17) and then supported by chemical and enzymatic probing (5, 24, 51). Interestingly, predictions of RNA folding intermediates suggested unequal frequencies of occurrence of the two DB structures (32). Previous functional studies supported the idea that both DB elements act as enhancers of DENV replication (31, 32). These previous reports from our laboratory and others used replicon systems lacking *cis*-acting elements at the 5′ end of the genome that could alter DB functions (34, 52, 53). For this reason, in the present work, we used reporter DENV genomes that included all identified RNA structures located in the coding sequence of the capsid protein (34). Using this system, we provide new information about differential roles of the two DB elements in infected cells. Sequence and RNA structure analyses of different DENV serotypes and genotypes suggested that the paralogous DB structures diverged after duplication, also supporting specialized functions (Fig. 2).

We found that DB2 contains structural elements that regulate viral replication. Adaptive mutations selected in mosquitoes were identified that reduced DB2-PK stability, and experiments in mosquito cells with recombinant and rescued viruses, from infections with cyclization-impaired or -enhanced mutants, supported the involvement of DB2 in regulating genome cyclization (Fig. 1, 5, and Fig. 6). Together, the data indicate a function of the local DB2-PK folding in reducing long-range RNA-RNA interactions and viral RNA replication. We previously showed that sequences within DB1 hybridize with a region present in the capsid coding sequence (Fig. 4) and that disassembling DB1 structure promoted long-range interactions and genome cyclization (34). These functions of the two DBs in regulating genome conformations could explain the observed opposite impact of deleting each structure on viral RNA accumulation (Fig. 3C).

It is still intriguing why the overall structure of DB2 is retained in nature. Covariations that maintained the structure suggest that DB2 structure may play additional functions. In this regard, it is important to mention the presence of a conserved sequence (CS2), which is present in both DBs (17, 54). Perhaps a correct architecture around CS2 is important for the redundant function observed in mosquito and human cell infections (Fig. 3B and E). We propose that the two DB structures are complex RNA elements that accommodate multiple signals that modulate viral processes.

The DENV genome functions as a dynamic molecule, and changes in the equilibrium between linear and circular forms of the viral RNA have been previously demonstrated to have a great impact on viral infectivity (47, 48, 55–57). A number of functional RNA structures that exist in the linear form of the genome were found to overlap with cyclization elements (e.g., sHP, 3' SL, SLB, and DB), providing a mechanism for controlling conformations of the viral RNA during replication. Regulation of genome conformations could also modulate the efficiency of viral translation. In fact, mutations of the top loop of both DBs have been previously reported to moderately reduce translation (32). Our results with mutations or deletions of DB2 support its role in regulation of a viral process that requires cyclization. In this regard, it is expected that translation would not be promoted by genome cyclization through RNA-RNA interactions including 5′-UTR sequences. However, more work will be necessary to understand the possible involvement of the DB elements and the requirement of specific genome conformations during early stages of infection, such as uncoating and translation. It is likely that cellular or viral proteins with RNA helicase or chaperone activities also modulate conformations of the viral genome. In this regard, a number of mammalian cellular proteins have been reported as candidates to modulate viral RNA structures (58–63). In particular, a cellular DEAD box RNA helicase known as DDX6 has been demonstrated to interact specifically with DB elements, but the significance of this interaction requires further studies (60).

Interestingly, the windows of tolerance for alterations of the equilibrium between circular and linear forms of the genome for DENV viability were found to be different in infected mosquito or human cells. Mutations that displace this equilibrium that were tolerated for replication in human cells were detrimental in mosquito cells (34, 35). DB mutations that greatly impact viral replication in mosquito but not in human cells may be associated with different requirements in the two hosts for fine-tuning genome conformations. We conclude that evolution of viral sequences involved in mutually exclusive structures, local and long-range RNA-RNA interactions, has led to a mechanism to control utilization of the viral genome for different processes. It is remarkable that in all MBFV genomes, the DB-PK structures overlap cyclization sequences (Fig. 7), suggesting a widely conserved mechanism.

It has been shown recently that DENV genetic variations in mosquitoes alter the transmission kinetics (40). Our data support the idea that DENV infection in mosquitoes is a possible source for 3′-UTR sequence variations (Fig. 1 and 6). In this regard, an entomological surveillance in Brazil also revealed deletions and insertions in the viral 3′ UTR (10), supporting the idea that sequence variability in this region could be associated with different function/requirement of RNA structures in the two hosts. It is important to mention that variations in flavivirus 3′ UTRs can also be originated by selective pressures impinged by the RNA interference (RNAi) response of invertebrate cells. Studies using WNV have previously shown diversification of the viral genome by the mosquito RNAi machinery (64).

The structure of DB2 has been a target for viral attenuation in different flaviviruses (65, 66). In the case of DENVs, the delta30 mutant is a promising vaccine candidate. Thus, defining the mechanisms by which DB2 participates in viral processes is also important for understanding attenuation. Here, we observed that while complete deletion of DB2 resulted in increased replication capacity of the virus in mosquito cells, the delta30 mutation did not (Fig. 3). One possible explanation for these phenotypes is that a partial deletion of DB2 results in RNA misfolding or leads to structural rearrangements that do not occur when the complete structure is deleted (67). This may also explain the reduced amounts of sfRNAs in infected mosquito cells with DENV delta30 (Fig. 3I), in agreement with recent observations in human cells (44), which could also be associated with viral attenuation. Nevertheless, it is important to highlight that in mosquito cells, DENV2 delta30 did not display enhanced viral replication.

MBFV contain two pairs of duplicated RNA structures in the 3′ UTR: two SLs and two DBs. We previously investigated the function of the two SLs in mosquitoes and mosquito and human cultured cells and found that RNA duplication supports efficient DENV host switching (5). In this regard, it was found that SLII was subjected to sequence variations during host adaptation, while SLI was unchanged. In the case of DENV, the two SLs were found to be functionally coupled for sfRNA generation, and sequence variations acquired in mosquito adaptation were detrimental for sfRNA1 formation (8), which is an sfRNA important in counteracting interferon (IFN) responses in human cells. Here, we identified sequence variations in one of the two DB structures in DENVs that were isolated after passage in mosquitoes. This observation was intriguing because of the striking resemblance to that observed in the duplicated SLs. Because deletion of both DB elements was near lethal for viral replication in mosquito and human cells, and deletion of DB2 was greatly advantageous for mosquito infection, we propose that maintenance of double copies of RNA structures is a viral strategy to ensure functionality of one conserved element, while the other is under different selective pressures in the two hosts.

Most MBFVs contain duplications of DB elements in their 3′ UTRs. However, RNA structures containing conserved DB elements are also found in flaviviruses with no known vectors (NKVFs), such as Yokose virus (YOKV), Entebbe bat virus (ENTV) and Modoc virus (MODV), and in insect-specific flaviviruses (ISFs) related to MBFVs, such as Chaoyang virus (CHAOV) and Nhumirim virus (NHUV) (for review see reference 19). These viruses infect either vertebrate or invertebrate cells, lacking the ability to alternate between hosts. Interestingly, single copies of DB structures are found in their 3′ UTRs. This evolutionary trend supports the idea that duplicated DBs are required for flaviviruses with dual hosts.

The mechanisms by which the two pairs of duplicated RNA elements (SLs and DBs) participate in the viral transmission cycle appear to be different; however, both are under host-specific selective pressures. Sequence and RNA structure duplications at the viral 3′ UTR were also reported to be relevant for host adaptation in other arboviruses. In this regard, an interesting report using chikungunya virus (CHIKV) described the process of viral 3′-UTR evolution in different hosts (68). In this case, duplication of direct repeats (DRs) in the CHIKV 3′ UTR was found to be beneficial for viral replication in mosquito cells rather than in human cells. The accumulating data using different arthropod-borne virus systems suggest convergent evolutionary mechanisms associating RNA structure duplications with host adaptation.

In summary, our findings provide new insights about functions and host-specific requirements of duplicated RNA structures in flavivirus genomes. We believe that understanding mechanisms that enable these viruses to replicate in multiple hosts will help to elucidate factors that govern the geographic expansion of these important viral pathogens.

## MATERIALS AND METHODS

### Cell lines.

C6/36HT cells (Aedes albopictus cell line, adapted to grow at 33°C) were cultured in Leibovitz's L-15 medium supplemented with 10% fetal bovine serum, 100 U/ml penicillin, 100 µg/ml streptomycin, 0.3% tryptose phosphate broth, 0.02% glutamine, 1% minimal essential medium (MEM) nonessential amino acids solution, and 0.25 µg/ml amphotericin B (Fungizone). A549 cells (human epithelial lung cell line) were cultured in Dulbecco's modified Eagle’s medium with nutrient mixture F-12 (DMEM/F-12) supplemented with 10% fetal bovine serum, 100 U/ml penicillin, and 100 µg/ml streptomycin.

### Mosquito adaptation experiments.

Viral stocks were prepared in BHK cells. Supernatants were harvested at different times posttransfection, and viruses were quantified by plaque assays. Aedes albopictus, Lake Charles, strain laboratory mosquitoes originating from Louisiana were reared from eggs and maintained as adults at 28°C and 80% relative humidity and given water and sugar until infection. Adult female mosquitoes 5 days postemergence were intrathoracically (i.t.) inoculated with approximately 70 nl of cell culture medium with a total titer of 200 PFU, using a Nanojet II (Drummond Scientific Company). Mosquitoes were maintained for 14 days, and a pool of five mosquitoes was used for RNA extraction and sequencing (P1). In addition, two batches of 10 mosquitoes each were homogenized in 1 ml of grinding medium and virus titer was determined. New females were injected with 70 nl of P1 extracts. After 14 days, RNA from groups of five mosquitoes was extracted and used for sequencing (P2).

### Sequencing analysis.

Viral RNAs were TRIzol extracted (Invitrogen) from homogenized mosquitoes and used for reverse transcription-PCRs (RT-PCRs) in duplicates with tagged primers specifically designed to amplify the last 600 nucleotides of both genome ends, using SuperScript III reverse transcriptase (Invitrogen) and Platinum Pfx DNA polymerase (Invitrogen). As a reference, the *in vitro*-transcribed viral RNA, the DNA plasmid template, and the viral RNA input from BHK supernatants were also sequenced. Libraries were sequenced using a 454 Genome Sequencer FLX Titanium XLR70 system (Roche), and raw data were analyzed as describe below. Reads covering 400 nucleotides of the 3′ and 5' UTRs were aligned to the reference sequence. Positions of alignments from low-complexity regions with repetitions major to four nucleotides were discarded from the analysis. To define selected viral variants, a sequence-to-vector approach was applied (69). For each alignment, a comparative matrix was constructed, and a dimensional reduction of matrices was performed using single-value decomposition. The 10 more important dimensions were used to determine the clusters of sequences using “density-based clustering.” Finally, prototype sequence of each cluster corresponding to each variant was determined by the consensus sequence.

### Cloning and sequencing revertant viruses.

Viral RNA was TRIzol extracted from clarified supernatants. RNA was treated with tobacco acid pyrophosphatase (Epicenter) to remove caps, and 3′ and 5′ ends of the RNA were ligated with T4 RNA ligase (Epicenter) as previously described (70). After phenol-chloroform extraction and ethanol precipitation, the pellet was resuspended in 20 µl of RNase-free water. Five microliters was used for RT using random primers and PCR using primers designed to amplify the 5′-3′ junction region. The RT-PCR products were directly sequenced to determine the consensus sequence and cloned into pGEM-T Easy plasmid (Promega). At least 20 independent clones were sequenced for each PCR product.

### Dumbbell structure analysis.

For RNA structure similarity analysis, the RNAforester software was used (43). Models based on previous biochemical and bioinformatics information were used as input, and from this, similarity matrices were built using “pairwise alignment” function. The matrices were normalized and used to calculate the distances to plot the dendrograms using the UPGMA method (unweighted pair group method using average linkages). For the RNA structure analysis, DENV2 isolates representative of different genotypes were used.

### Construction of recombinant DENVs.

The construction of recombinant DENV cDNAs (DENV2 full-length clones, pDV) was performed as previously described (31). For the reporter constructs, FullCapDVLuc, a monocistronic DENV reporter construct containing the Renilla luciferase (Rluc) gene previously described (34), was modified by replacing the AflII*-*XbaI WT fragment with a fragment containing the mutations. All constructs were confirmed by DNA sequencing analysis using ABI Prism 3130 genetic analyzer and BigDye terminator 3.1 chemistry (Applied Biosystems).

### RNA transcriptions and transfections.

Infectious viral cDNA plasmids were linearized with XbaI (New England Biolabs) and used as the templates for *in vitro* transcription using T7 RNA polymerase (Ambion) in the presence of m7GpppA cap analog (New England Biolabs). RNA integrity was confirmed in 1% agarose RNase-free gels. RNA transcripts were transfected into C6/36HT and A549 cells grown in 24-well plates using Lipofectamine 2000 and Opti-MEM media (Invitrogen). The Rluc activity in transfected cells was analyzed using the Promega *Renilla* luciferase assay system according to the manufacturer’s instructions. Data were analyzed by one-way analysis of variance (ANOVA) with Bonferroni’s or Dunnett’s correction for multiple comparisons.

### RNA extraction, reverse transcription, and real-time PCR.

For quantification by real-time RT-PCR, total viral RNA was TRIzol extracted at various time points posttransfection. RNA samples were reverse transcribed in 20-µl reaction volumes, as previously described (71). For real-time PCR, reactions were performed in duplicates in 96-well plates using 2 µl of the RT reaction mixture as the template, 5 µl FastStart SYBR green Master 2× mix (Roche), 300 nM each primer, and RNase-free water to 10 µl. The primers AVG1117 (5'-ACAAGTCGAACAACCTGGTCCAT-3′) and AVG1118 (5'-GCCGCACCATTGGTCTTCTC-3′) were targeted to amplify nucleotides 9937 to 10113 within the NS5 coding sequence. Reactions were run with the following parameters: 95°C for 10 min and then 40 cycles of 95°C for 30 s and 60°C for 40s.

### Immunofluorescence assays.

C6/36HT cells transfected with WT or mutated DENV RNAs were grown in 24-well plates containing 1-cm^2^ coverslips. At various times posttransfection, coverslips were removed, and cells were fixed with methanol for 15 min at 20°C. For detection of viral antigens, a specific anti-E monoclonal antibody (MAb), E18, was used. Alexa Fluor 488-conjugated rabbit anti-mouse immunoglobulin G (Molecular Probes) was employed to detect the primary antibody under the same conditions.

### Titration.

Viral titers were determined by PFU in BHK-21 cells.

### Northern blotting.

For DENV sfRNA detection, total RNA was obtained from transfected cells using TRIzol reagent (Invitrogen) at different times posttransfection or postinfection and separated on 5% polyacrylamide–7 M urea gel, as previously described (8). For detection, uniformly ^32^P-labeled RNA probe complementary to the complete 3′ UTR (nucleotides 10269 to 10723) was used.

### Slot blots.

For detection of genomic RNA (gRNA), a uniformly ^32^P-labeled RNA probe complementary to a region of NS2B sequence was used. Samples were loaded into a slot blot apparatus using nylon membrane (Hybond-N; GE Healthcare).

### Predicted RNA structures in the genome of mosquito-borne flaviviruses.

Genome sequences of representative viruses corresponding to different subgroups of mosquito-borne flaviviruses were used (yellow fever virus [YFV], Kokobera virus [KOKV], Zika virus [ZIKV], Japanese encephalitis virus [JEV], Saint Louis encephalitis virus [SLEV], West Nile virus [WNV], Iguape virus [IGUV], and dengue virus [DENV]). The RNAfold package software was used to detect thermodynamically stable and evolutionarily conserved RNA secondary structures. To characterize possible tertiary interactions, complementary base pairs, and covariations, Biostring functions and the RNAaliduplex software were used together with predicted secondary structure models. For each long-range interaction, we calculated the Z-score using a methodology previously proposed (72). Detailed information of GenBank ID references of complete genome sequences used throughout this work is shown in Table S1 in the supplemental material.

10.1128/mBio.02506-18.1 TABLE S1 Accession numbers. Download Table S1, PDF file, 0.5 MB. Copyright © 2019 de Borba et al. 2019 de Borba et al. This content is distributed under the terms of the Creative Commons Attribution 4.0 International license.
