# Supplementary material for: RNA Structure Duplication in the Dengue Virus 3′ UTR: Redundancy or Host Specificity?
Source: mBio. 2019 Jan 8;10(1):e02506-18. doi: 10.1128/mBio.02506-18 (PMC6325252; doi:10.1128/mBio.02506-18)
Supplement: TABLE S1 [file mBio.02506-18-st001.pdf]

MBFV	Strain Name	Species Name	GenBank Accession	Sequence Length	Host	Country
ZIKA	SM-6 V-1	Zika virus	DQ859064	10290	Unknown	South Africa
	ARB13565	Zika virus	KF268948	10788	Mosquito	Central African Republic
	ArB1362	Zika virus	KF383115	10272	Unknown	-N/A-
	ARB15076	Zika virus	KF268949	10776	Insect	Central African Republic
	ARB7701	Zika virus	KF268950	10755	Mosquito	Central African Republic
	ArD128000	Zika virus	KF383117	10272	Unknown	-N/A-
	ArD157995	Zika virus	KF383118	10272	Unknown	-N/A-
	ArD158084	Zika virus	KF383119	10272	Unknown	-N/A-
	ArD7117	Zika virus	KF383116	10272	Unknown	-N/A-
	FSM	Zika virus	EU545988	10272	Human	Micronesia
	H/PF/2013	Zika virus	KJ776791	10617	Human	French Polynesia
	MR 766	Zika virus	AY632535	10794	Monkey	Uganda
	MR 766	Zika virus	DQ859059	10254	Unknown	Uganda
	MR 766	Zika virus	NC_012532 *	10794	Monkey	Uganda
	MR766-NIID	Zika virus	LC002520	10807	Unknown	Uganda
	Z1106033	Zika virus	KU312312	10374	Human	-N/A-
	ZikaSPH2015	Zika virus	KU321639	10676	Human	-N/A-
	Zika virus	Zika virus	MF098771	10657	-	Russia
	SG(EHI)ZIKV/33164Y17	Zika virus	MF988734	10415	-	Singapore
	Zika virus/Homo sapiens/Cuba 2017	Zika virus	MF438286	10272	-	Cuba
	ZIKV/Homo_sapiens/Cuba/2017/FL046Se	Zika virus	MH063259	10386	-	Cuba
	ZIKV/Homo_sapiens/Cuba/2017/FL047U	Zika virus	MH063260	10342	-	Cuba
	ZIKV/Homo_sapiens/Cuba/2017/FL049U	Zika virus	MH063261	10385	-	Cuba
	ZIKV/Homo_sapiens/Cuba/2017/FL051Se	Zika virus	MH063262	10619	-	Cuba
	ZIKV/Homo_sapiens/Cuba/2017/FL052U	Zika virus	MH063263	10098	-	Cuba
	ZIKV/Homo_sapiens/Cuba/2017/FL055Se	Zika virus	MH063264	10366	-	Cuba
	ZIKV/Homo_sapiens/Cuba/2017/FL057U	Zika virus	MH063265	10341	-	Cuba
	ZIKV/Homo_sapiens/Cuba/2017/Hu0046Sa	Zika virus	MF159531	10608	-	USA
	ZIKV/Hu/Thai/KngSG/17-D501	Zika virus	LC369584	10718	-	Japan
JEV	14178	Japanese encephalitis virus	EF623987	10976	Unknown	India
	04940-4	Japanese encephalitis virus	EF623989	10976	Unknown	India
	57434	Japanese encephalitis virus	EF623988	10976	Unknown	India
	1070/82 (Subin)	Japanese encephalitis virus	GQ902059	10942	Human	Thailand
	131V	Japanese encephalitis virus	GU205163	10965	Human	China
	3KP"U"CV569	Japanese encephalitis virus	GQ902060	10942	Mosquito	Thailand
	47	Japanese encephalitis virus	JF706269	10977	Unknown	China
	4790-85	Japanese encephalitis virus	GQ902062	10942	Human	Thailand
	90VN70(1)	Japanese encephalitis virus	HM228921	10965	Human	Viet Nam
	Anyang300	Japanese encephalitis virus	KF711994	10299	Swine	South Korea
	at222	Japanese encephalitis virus	AB196924	10977	Unknown	-N/A-
	AT31	Japanese encephalitis virus	AB196923	10977	Unknown	-N/A-
	AT31 recombinant	Japanese encephalitis virus	AB196926	10977	Unknown	-N/A-
	attenuated SA14-12-1-7	Japanese encephalitis virus	AF416457	10976	Unknown	-N/A-
	B-0860/82	Japanese encephalitis virus	GQ902058	10942	Swine	Thailand
	B-1381-85	Japanese encephalitis virus	GQ902061	10942	Swine	Thailand
	B58	Japanese encephalitis virus	FJ185036	10977	Bat	China
	Beijing-1	Japanese encephalitis virus	L48961	10976	Unknown	-N/A-
	BL06-50	Japanese encephalitis virus	JF706270	10965	Mosquito	China
	BL06-54	Japanese encephalitis virus	JF706271	10965	Mosquito	China
	CBH	Japanese encephalitis virus	JN381860	10977	Unknown	-N/A-
	CC27-L1	Japanese encephalitis virus	AY303795	10970	Unknown	-N/A-
	CC27-L3	Japanese encephalitis virus	AY303796	10970	Unknown	-N/A-
	CC27-S6	Japanese encephalitis virus	AY303797	10970	Unknown	-N/A-
	CC27-S8	Japanese encephalitis virus	AY303798	10970	Unknown	-N/A-
	CH13	Japanese encephalitis virus	JN381870	10977	Unknown	-N/A-
	CH1392	Japanese encephalitis virus	AF254452	10970	Mosquito	Taiwan
	CH2195LA	Japanese encephalitis virus	AF221499	10976	Unknown	-N/A-
	CH2195SA	Japanese encephalitis virus	AF221500	10976	Unknown	-N/A-
	CJN-L1	Japanese encephalitis virus	AY303794	10970	Unknown	-N/A-
	CJN-S1	Japanese encephalitis virus	AY303793	10970	Unknown	-N/A-
	CNU-LP2	Japanese encephalitis virus	AY585243	10968	Unknown	South Korea
	CZX	Japanese encephalitis virus	JN381865	10977	Unknown	-N/A-
	DH107	Japanese encephalitis virus	JN381873	10975	Unknown	-N/A-
	DH10M865	Japanese encephalitis virus	KT229572	10965	Mosquito	China
	DH10M978	Japanese encephalitis virus	KT229573	10965	Mosquito	China
	DHL10M62	Japanese encephalitis virus	KT229575	10965	Mosquito	China
	DL04-29	Japanese encephalitis virus	JF706272	10977	Mosquito	China
	DL0445	Japanese encephalitis virus	JN381854	10977	Unknown	-N/A-
	Fj02-29	Japanese encephalitis virus	JF706273	10977	Unknown	China
	Fj0276	Japanese encephalitis virus	JN381867	10977	Unknown	-N/A-
	FJ0339	Japanese encephalitis virus	JN381859	10977	Unknown	-N/A-
	FJ0394	Japanese encephalitis virus	JN381858	10977	Unknown	-N/A-
	FU	Japanese encephalitis virus	AF217620	10964	Unknown	Australia
	GB30	Japanese encephalitis virus	FJ185037	10977	Bat	China
	GD	Japanese encephalitis virus	JN711458	10976	Bat	China
	GP78	Japanese encephalitis virus	AF075723	10976	Unknown	-N/A-
	GS07TS11	Japanese encephalitis virus	JN381843	10965	Unknown	-N/A-
	GSBY0801	Japanese encephalitis virus	JF706274	10965	Mosquito	China
	GSBY0804	Japanese encephalitis virus	JN381844	10965	Unknown	-N/A-
KOKV	MK7979	Kokobera virus	KM225264	10648	Mosquito	Papua New Guinea
	AusMRM 32	Kokobera virus	AY632541	10874	Unknown	-N/A-
	AusMRM 32	Kokobera virus	NC_009029 *	10874	Unknown	-N/A-
	CY1014	Kokobera virus	KC788512	10864	Mosquito	Australia
	C338	Kokobera virus	KF917540	10245	Mosquito	Australia
	C338	Kokobera virus	KM225263	10560	Unknown	Australia
	TS5273	Kokobera virus	KM225265	10735	Mosquito	Australia
NTAVG	Strain Name	Species Name	GenBank Accession	Sequence Length	Host	Country
	IPDIA	Ntaya virus	JX236040	10943	Unknown	-N/A-
	IPDIA	Ntaya virus	NC_018705 *	10943	Unknown	-N/A-
	Original	Ntaya virus	KF917539	10284	Mosquito	Uganda
BAGV	96363	Bagaza virus	EU684972	10284	Mosquito	India
	BAGV/Spain/RLP-Hcc1/2010	Bagaza virus	KR108244	11000	Partridge	Spain
	BAGV/Spain/RLP-Hcc2/2010	Bagaza virus	KR108245	10998	Partridge	Spain
	BAGV/Spain/RLP-Hcc3/2010	Bagaza virus	KR108246	10975	Partridge	Spain
	DakAr B209	Bagaza virus	AY632545	10941	Mosquito	Central African Republic
	DakAr B209	Bagaza virus	NC_012534 *	10941	Mosquito	Central African Republic
	Spain B/2010	Bagaza virus	HQ644144	10785	Partridge	Spain
	Spain H/2010	Bagaza virus	HQ644143	10794	Partridge	Spain
	Israel/105520/2010	Bagaza virus	KC734553	10777	Avian	Israel
	Israel/106819/2010	Bagaza virus	KC734552	10794	Avian	Israel
	Israel/107458/2010	Bagaza virus	KC734551	10794	Avian	Israel
	Israel/618/1995	Bagaza virus	KC734550	10792	Avian	Israel
	ME 30502	Bagaza virus	KF917537	10284	Turkey	Israel
	UNKNOWN-KC734549	Bagaza virus	KC734549	10792	Avian	Israel
ILHV	Ilheus virus strain BrMS-MQ10, complete genome		KC481679.1	10759		
BSQV	Bussuquara virus, complete genome		NC_009026.2	10815		
IGUV	Iguape virus strain SPAn 71686, complete genome		AY632538.4	10922		
ROCV	Rocio virus strain SPH 34675, complete genome		AY632542.4	10794		
SEPV	7148	Sepik virus	DQ859063	10218	Unknown	Papua New Guinea
	MK7148	Sepik virus	DQ837642	10793	Mosquito	Papua New Guinea

	MK7148	Sepik virus	NC_008719 *	10793	Mosquito	Papua New Guinea
	UNKNOWN-AY632543	Sepik virus	AY632543	10218	Unknown	-N/A-
**WESSV**	Wesselsbron virus from South Africa, complete genome		JN226796.1	10814		
**ALPV**	Aphid lethal paralysis virus, complete genome		NC_004365.1	9812		
**USUV**	Usutu virus strain Spain MB119/06, complete genome		KF573410.1	11064		
**MVEV**	MRM3929	Murray Valley encephalitis virus	AY898809	10960	Unknown	Australia
	MK6684	Murray Valley encephalitis virus	KF751869	11014	Mosquito	Papua New Guinea
	MVE-1-51	Murray Valley encephalitis virus	AF161266	11014	Unknown	-N/A-
	MVE-1-51	Murray Valley encephalitis virus	NC_000943 *	11014	Unknown	-N/A-
	MVE-1-51-C10	Murray Valley encephalitis virus	KC852190	11014	Unknown	Australia
	MVE-1-51-CD8	Murray Valley encephalitis virus	KC852192	11014	Unknown	Australia
	MVE-1-51-D10	Murray Valley encephalitis virus	KC852191	11014	Unknown	Australia
	MVE-1-51-P0	Murray Valley encephalitis virus	KC852189	11014	Unknown	Australia
	NG156	Murray Valley encephalitis virus	KF751870	11012	Human	Papua New Guinea
	OR156	Murray Valley encephalitis virus	KC852193	10953	Unknown	Australia
	OR156	Murray Valley encephalitis virus	KC852194	10953	Unknown	Australia
	OR156	Murray Valley encephalitis virus	KC852195	10953	Unknown	Australia
	OR156	Murray Valley encephalitis virus	KC852196	10953	Unknown	Australia
	OR156	Murray Valley encephalitis virus	KF751871	10953	Mosquito	Australia
	UNKNOWN-FV537353	Murray Valley encephalitis virus	FV537353	11014	Unknown	-N/A-
	UNKNOWN-FV537354	Murray Valley encephalitis virus	FV537354	11014	Unknown	-N/A-
	UNKNOWN-FV537355	Murray Valley encephalitis virus	FV537355	11014	Unknown	-N/A-
	UNKNOWN-FV537356	Murray Valley encephalitis virus	FV537356	11014	Unknown	-N/A-
	V11-10	Murray Valley encephalitis virus	JX123032	11005	Horse	Australia
**KUNV**	FLSDX	West Nile virus	AY274504	11022	Unknown	-N/A-
	MRM61C	West Nile virus	D00246	10664	Unknown	-N/A-
	pAKUN	West Nile virus	AY274505	11022	Unknown	-N/A-
**DENV 1**	00407/95-1	Dengue virus 1	JN638344	10735	Unknown	Thailand
	01096/07-1	Dengue virus 1	HM469966	10735	Human	Thailand
	02-20(1)	Dengue virus 1	AB178040	10735	Unknown	Japan
	03881/92-1	Dengue virus 1	JN638343	10735	Unknown	Thailand
	12898/BR-PE/10	Dengue virus 1	JX669462	10737	Human	Brazil
	13501/BR-PE/10	Dengue virus 1	JX669463	10735	Human	Brazil
	13671/BR-PE/10	Dengue virus 1	JX669464	10736	Human	Brazil
	13861/BR-PE/10	Dengue virus 1	JX669465	10736	Human	Brazil
	14985/BR-PE/10	Dengue virus 1	JX669466	10737	Human	Brazil
	16007 (PDK-13)(1)	Dengue virus 1	AF180818	10735	Unknown	-N/A-
	16007(1)	Dengue virus 1	AF180817	10735	Unknown	-N/A-
	2008/00475	Dengue virus 1	HE795086	10179	Human	France
	21814/BR-PE/96	Dengue virus 1	JX669467	10737	Human	Brazil
	259par00(1)	Dengue virus 1	AF514883	10735	Unknown	-N/A-
	280par00(1)	Dengue virus 1	AF514878	10735	Unknown	-N/A-
	293arg00(1)	Dengue virus 1	AY206457	10735	Unknown	-N/A-
	295arg00(1)	Dengue virus 1	AF514885	10735	Unknown	-N/A-
	297arg00(1)	Dengue virus 1	AF514889	10735	Unknown	-N/A-
	301arg00(1)	Dengue virus 1	AF514876	10735	Unknown	-N/A-
	30231/97-1	Dengue virus 1	JN638340	10735	Unknown	Thailand
	30399/95-1	Dengue virus 1	JN638339	10735	Unknown	Thailand
	30597/07-1	Dengue virus 1	HM469967	10735	Human	Thailand
	360052	Dengue virus 1	AB608789	10677	Human	Taiwan
	40604/BR-PE/97	Dengue virus 1	JX669468	10736	Human	Brazil
	41111/BR-PE/97	Dengue virus 1	JX669469	10736	Human	Brazil
	49440-1	Dengue virus 1	JF459993	10735	Human	Myanmar
	52082/BR-PE/98	Dengue virus 1	JX669470	10728	Human	Brazil
	59049/BR-PE/99	Dengue virus 1	JX669471	10736	Human	Brazil
	70523/BR-PE/00	Dengue virus 1	JX669472	10736	Human	Brazil
	71/02GZ(1)	Dengue virus 1	EF025110	10735	Unknown	China
	74488/BR-PE/01	Dengue virus 1	JX669473	10736	Human	Brazil
	75861/BR-PE/01	Dengue virus 1	JX669474	10720	Human	Brazil
	832	Dengue virus 1	AB608788	10693	Human	Taiwan
	88463/BR-PE/02	Dengue virus 1	JX669475	10737	Human	Brazil
	9808/BR-PE/10	Dengue virus 1	JX669461	10736	Human	Brazil
	98901518 DHF DV-1(1)	Dengue virus 1	AB189120	10735	Human	Indonesia
	98901530 DF DV-1(1)	Dengue virus 1	AB189121	10735	Human	Indonesia
	A88(type 1)	Dengue virus 1	AB074761	10735	Unknown	-N/A-
	Abidjan(1)	Dengue virus 1	AF298807	10721	Unknown	Cote D'Ivoire
	Angola_2013-1	Dengue virus 1	KF184975	10602	Human	Angola
	ARG0028	Dengue virus 1	AY277665	10735	Unknown	-N/A-
	ARG0044	Dengue virus 1	AY277659	10735	Unknown	-N/A-
	ARG0048	Dengue virus 1	AY277666	10735	Unknown	-N/A-
	ARG9920	Dengue virus 1	AY277664	10735	Unknown	-N/A-
	BNI-5201	Dengue virus 1	KJ468234	10622	Human	Germany
	BR/01-MR(1)	Dengue virus 1	AF513110	10735	Unknown	-N/A-
	BR/97-111(1)	Dengue virus 1	AF311956	10735	Human	-N/A-
	BR/97-233(1)	Dengue virus 1	AF311958	10735	Human	-N/A-
	BR/97-409(1)	Dengue virus 1	AF311957	10735	Human	-N/A-
	Cambodia(1)	Dengue virus 1	AF309641	10721	Unknown	Cambodia
	CHI3336-02(1)	Dengue virus 1	EU863650	10735	Human	Chile
	CHN/GuangDong/ZhongShan/Guzhen01/2013	Dengue virus 1	KF971870	10583	Human	China
	CHN/GuangDong/ZhongShan/Henglan01/2013	Dengue virus 1	KF971871	10583	Human	China
	CHN/GuangDong/ZhongShan/HuangPu01/2013	Dengue virus 1	KF971869	10583	Human	China
	Comoros 04.329/93(1)	Dengue virus 1	DQ285562	10721	Human	Comoros
	D1.Myanmar.059/01(1)	Dengue virus 1	AY708047	10614	Unknown	Myanmar
	D1.Myanmar.194/01(1)	Dengue virus 1	AY713474	10614	Unknown	Myanmar
	D1.Myanmar.206/01(1)	Dengue virus 1	AY713475	10614	Unknown	Myanmar
	D1.Myanmar.23819/96(1)	Dengue virus 1	AY722802	10614	Unknown	Myanmar
	D1.Myanmar.305/01(1)	Dengue virus 1	AY713476	10614	Unknown	Myanmar
	D1.Myanmar.31459/98(1)	Dengue virus 1	AY726555	10726	Unknown	Myanmar
	D1.Myanmar.31987/98(1)	Dengue virus 1	AY726554	10735	Unknown	Myanmar
	D1.Myanmar.32514/98(1)	Dengue virus 1	AY722803	10614	Unknown	Myanmar
	D1.Myanmar.37726/01(1)	Dengue virus 1	AY726549	10614	Unknown	Myanmar
	D1.Myanmar.38862/01(1)	Dengue virus 1	AY726550	10614	Unknown	Myanmar
	D1.Myanmar.40553/71(1)	Dengue virus 1	AY713473	10614	Unknown	Myanmar
	D1.Myanmar.40568/76(1)	Dengue virus 1	AY722801	10614	Unknown	Myanmar
	D1.Myanmar.44168/01(1)	Dengue virus 1	AY726551	10614	Unknown	Myanmar
	D1.Myanmar.44988/02(1)	Dengue virus 1	AY726552	10614	Unknown	Myanmar
	D1.Myanmar.49440/02(1)	Dengue virus 1	AY726553	10614	Unknown	Myanmar
	D1/H/IMTSSA/98/606(1)	Dengue virus 1	AF298808	10721	Unknown	Djibouti
	D1/Hu/Chiba/NIID153/2014	Dengue virus 1	LC011948	10693	Human	Japan
	D1/Hu/Hyogo/NIID188/2014	Dengue virus 1	LC016760	10693	Human	Japan
	D1/Hu/Saitama/NIID100/2014	Dengue virus 1	LC011945	10693	Human	Japan
	D1/hu/Seychelles/NIID41/2003(1)	Dengue virus 1	AB195673	10718	Human	Japan
	D1/Hu/Shizuoka/NIID181/2014	Dengue virus 1	LC011949	10693	Human	Japan
	D1/Hu/Tokyo/NIID111/2014	Dengue virus 1	LC011946	10693	Human	Japan
	D1/Hu/Tokyo/NIID149/2014	Dengue virus 1	LC011947	10693	Human	Japan
	D1/hu/Yap/NIID27/2004(1)	Dengue virus 1	AB204803	10706	Human	Japan
	D1/SG/05K2398DK1/2005(1)	Dengue virus 1	EU081229	10735	Unknown	Singapore
	D1/SG/05K2402DK1/2005(1)	Dengue virus 1	EU081230	10735	Unknown	Singapore
	D1/SG/05K2887DK1/2005(1)	Dengue virus 1	EU081231	10735	Unknown	Singapore
	D1/SG/05K2895DK1/2005(1)	Dengue virus 1	EU081232	10735	Unknown	Singapore
	D1/SG/05K2901DK1/2005(1)	Dengue virus 1	EU081233	10735	Unknown	Singapore
	D1/SG/05K2916DK1/2005(1)	Dengue virus 1	EU081234	10735	Unknown	Singapore

D1/SG/05K2928DK1/2005(1)	Dengue virus 1	EU081235	10735	Unknown	Singapore
D1/SG/05K3297DK1/2005(1)	Dengue virus 1	EU081236	10735	Unknown	Singapore
D1/SG/05K3300DK1/2005(1)	Dengue virus 1	EU081237	10722	Unknown	Singapore
D1/SG/05K3301DK1/2005(1)	Dengue virus 1	EU081238	10735	Unknown	Singapore
D1/SG/05K3318DK1/2005(1)	Dengue virus 1	EU081239	10735	Unknown	Singapore
D1/SG/05K3886DK1/2005(1)	Dengue virus 1	EU081240	10735	Unknown	Singapore
D1/SG/05K3894DK1/2005(1)	Dengue virus 1	EU081241	10722	Unknown	Singapore
D1/SG/05K3903DK1/2005(1)	Dengue virus 1	EU081242	10735	Unknown	Singapore
D1/SG/05K3904DK1/2005(1)	Dengue virus 1	EU081243	10735	Unknown	Singapore
D1/SG/05K3905DK1/2005(1)	Dengue virus 1	EU081244	10735	Unknown	Singapore
D1/SG/05K3908DK1/2005(1)	Dengue virus 1	EU081245	10735	Unknown	Singapore
D1/SG/05K3910DK1/2005(1)	Dengue virus 1	EU081246	10735	Unknown	Singapore
D1/SG/05K3911DK1/2005(1)	Dengue virus 1	EU081247	10735	Unknown	Singapore
D1/SG/05K3915DK1/2005(1)	Dengue virus 1	EU081248	10735	Unknown	Singapore
D1/SG/05K3916DK1/2005(1)	Dengue virus 1	EU081249	10735	Unknown	Singapore
D1/SG/05K3930DK1/2005(1)	Dengue virus 1	EU081250	10735	Unknown	Singapore
D1/SG/05K3933DK1/2005(1)	Dengue virus 1	EU081251	10735	Unknown	Singapore
D1/SG/05K3934DK1/2005(1)	Dengue virus 1	EU081252	10735	Unknown	Singapore
D1/SG/05K3935DK1/2005(1)	Dengue virus 1	EU081253	10735	Unknown	Singapore
D1/SG/05K4138DK1/2005(1)	Dengue virus 1	EU081254	10735	Unknown	Singapore
D1/SG/05K4139DK1/2005(1)	Dengue virus 1	EU081255	10735	Unknown	Singapore
D1/SG/05K4140DK1/2005(1)	Dengue virus 1	EU081256	10735	Unknown	Singapore
D1/SG/05K4142DK1/2005(1)	Dengue virus 1	EU081257	10735	Unknown	Singapore
D1/SG/05K4147DK1/2005(1)	Dengue virus 1	EU081258	10735	Unknown	Singapore
D1/SG/05K4152DK1/2005(1)	Dengue virus 1	EU081259	10735	Unknown	Singapore
D1/SG/05K4154DK1/2005(1)	Dengue virus 1	EU081260	10735	Unknown	Singapore
D1/SG/05K4172DK1/2005(1)	Dengue virus 1	EU081261	10735	Unknown	Singapore
D1/SG/05K4173DK1/2005(1)	Dengue virus 1	EU081262	10735	Unknown	Singapore
D1/SG/05K4174DK1/2005(1)	Dengue virus 1	EU081263	10735	Unknown	Singapore
D1/SG/05K4175DK1/2005(1)	Dengue virus 1	EU081264	10735	Unknown	Singapore
D1/SG/05K4183DK1/2005(1)	Dengue virus 1	EU081265	10735	Unknown	Singapore
D1/SG/05K4441DK1/2005(1)	Dengue virus 1	EU081266	10735	Unknown	Singapore
D1/SG/05K4443DK1/2005(1)	Dengue virus 1	EU081267	10735	Unknown	Singapore
D1/SG/05K4468DK1/2005(1)	Dengue virus 1	EU081268	10735	Unknown	Singapore
D1/SG/05K4479DK1/2005(1)	Dengue virus 1	EU081269	10735	Unknown	Singapore
D1/SG/05K4480DK1/2005(1)	Dengue virus 1	EU081270	10735	Unknown	Singapore
D1/SG/05K4604DK1/2005(1)	Dengue virus 1	EU081271	10735	Unknown	Singapore
D1/SG/05K4605DK1/2005(1)	Dengue virus 1	EU081272	10735	Unknown	Singapore
D1/SG/05K4606DK1/2005(1)	Dengue virus 1	EU081273	10735	Unknown	Singapore
D1/SG/05K4609DK1/2005(1)	Dengue virus 1	EU081274	10735	Unknown	Singapore
D1/SG/05K4620DK1/2005(1)	Dengue virus 1	EU081275	10735	Unknown	Singapore
D1/SG/05K4621DK1/2005(1)	Dengue virus 1	EU081276	10735	Unknown	Singapore
D1/SG/05K4622DK1/2005(1)	Dengue virus 1	EU081277	10735	Unknown	Singapore
D1/SG/05K4632DK1/2005(1)	Dengue virus 1	EU081278	10735	Unknown	Singapore
D1/SG/05K4820DK1/2005(1)	Dengue virus 1	EU081279	10735	Unknown	Singapore
D1/SG/05K814DK1/2005(1)	Dengue virus 1	EU081226	10735	Unknown	Singapore
D1/SG/05K847DK1/2005(1)	Dengue virus 1	EU081227	10735	Unknown	Singapore
D1/SG/05K872DK1/2005(1)	Dengue virus 1	EU081228	10735	Unknown	Singapore
D1/SG/06K2236DK1/2006(1)	Dengue virus 1	EU081280	10735	Unknown	Singapore
D1/SG/06K2290DK1/2006(1)	Dengue virus 1	EU081281	10735	Unknown	Singapore
D87-116-1	Dengue virus 1	JN638341	10735	Unknown	Thailand
D90-1197-1	Dengue virus 1	JN638342	10735	Unknown	Thailand
DEN1/GZ/OY	Dengue virus 1	FJ176779	10735	Human	China
DEN1/GZ/XNC	Dengue virus 1	FJ176780	10735	Human	China
Den1BR/90(1)	Dengue virus 1	AF226685	10735	Unknown	-N/A-
DenKor-01-1	Dengue virus 1	KP406801	10736	Human	South Korea
DenKor-02-1	Dengue virus 1	KP406802	10735	Human	South Korea
DenKor-07-1	Dengue virus 1	KP406803	10734	Human	South Korea
DENV-1-Jeddah	Dengue virus 1	KJ649286	10622	Human	Saudi Arabia
DENV-1/8/Thailand/01/2013	Dengue virus 1	KF887994	10733	Human	Thailand
DENV-1/BOL-KW010	Dengue virus 1	JQ675358	10605	Human	USA
DENV-1/BR/BID-V2374/2000(1)	Dengue virus 1	FJ850070	10677	Human	Brazil
DENV-1/BR/BID-V2375/2000(1)	Dengue virus 1	FJ850071	10685	Human	Brazil
DENV-1/BR/BID-V2378/2001(1)	Dengue virus 1	FJ850073	10692	Human	Brazil
DENV-1/BR/BID-V2381/2002(1)	Dengue virus 1	FJ850075	10683	Human	Brazil
DENV-1/BR/BID-V2384/2003(1)	Dengue virus 1	FJ850077	10684	Human	Brazil
DENV-1/BR/BID-V2389/2004(1)	Dengue virus 1	FJ850081	10688	Human	Brazil
DENV-1/BR/BID-V2392/2005(1)	Dengue virus 1	FJ850084	10686	Human	Brazil
DENV-1/BR/BID-V2395/2006(1)	Dengue virus 1	FJ850087	10690	Human	Brazil
DENV-1/BR/BID-V2398/2007(1)	Dengue virus 1	FJ850090	10673	Human	Brazil
DENV-1/BR/BID-V2401/2008(1)	Dengue virus 1	FJ850093	10690	Human	Brazil
DENV-1/BR/BID-V3490/2008(1)	Dengue virus 1	GU131863	10532	Human	Brazil
DENV-1/CO/BID-V3376/1998(1)	Dengue virus 1	GQ868559	10690	Human	Colombia
DENV-1/CO/BID-V3377/1998(1)	Dengue virus 1	GQ868560	10690	Human	Colombia
DENV-1/CO/BID-V3378/1999(1)	Dengue virus 1	GQ868561	10673	Human	Colombia
DENV-1/CO/BID-V3379/2001(1)	Dengue virus 1	GU131948	10627	Human	Colombia
DENV-1/CO/BID-V3380/2005(1)	Dengue virus 1	GQ868562	10674	Human	Colombia
DENV-1/CO/BID-V3381/2006(1)	Dengue virus 1	GQ868563	10661	Human	Colombia
DENV-1/CO/BID-V3382/2006(1)	Dengue virus 1	GQ868564	10690	Human	Colombia
DENV-1/CO/BID-V3383/2006(1)	Dengue virus 1	GU131949	10486	Human	Colombia
DENV-1/CO/BID-V3385/2006(1)	Dengue virus 1	GQ868565	10690	Human	Colombia
DENV-1/CO/BID-V3386/2007(1)	Dengue virus 1	GQ868566	10675	Human	Colombia
DENV-1/CO/BID-V3387/2007(1)	Dengue virus 1	GQ868567	10690	Human	Colombia
DENV-1/CO/BID-V3388/2007(1)	Dengue virus 1	GQ868568	10690	Human	Colombia
DENV-1/CO/BID-V3390/2007(1)	Dengue virus 1	GQ868569	10675	Human	Colombia
DENV-1/CO/BID-V3391/2008(1)	Dengue virus 1	GQ868570	10674	Human	Colombia
DENV-1/CO/BID-V7290/1998	Dengue virus 1	KJ189302	10445	Human	Colombia
DENV-1/CO/BID-V7291/1998	Dengue virus 1	KJ189303	10445	Human	Colombia
DENV-1/CO/BID-V7292/2005	Dengue virus 1	KJ189304	10447	Human	Colombia
DENV-1/IND/55290/2005-1	Dengue virus 1	JQ922548	10699	Human	India
DENV-1/IND/631288/1963-1	Dengue virus 1	JQ922544	10698	Human	India
DENV-1/IND/715393/1971-1	Dengue virus 1	JQ922546	10663	Human	India
DENV-1/IND/826883/1982-1	Dengue virus 1	JQ922545	10625	Human	India
DENV-1/IPC/BID-V3775/2006(1)	Dengue virus 1	GU131887	10457	Human	Cambodia
DENV-1/IPC/BID-V3776/2006(1)	Dengue virus 1	GU131888	10536	Human	Cambodia
DENV-1/IPC/BID-V3779/2006(1)	Dengue virus 1	GU131889	10479	Human	Cambodia
DENV-1/IPC/BID-V3780/2006(1)	Dengue virus 1	GU131890	10476	Human	Cambodia
DENV-1/IPC/BID-V3781/2006(1)	Dengue virus 1	GQ868630	10673	Human	Cambodia
DENV-1/IPC/BID-V3782/2006(1)	Dengue virus 1	GU131891	10464	Human	Cambodia
DENV-1/IPC/BID-V3783/2006(1)	Dengue virus 1	GU131892	10518	Human	Cambodia
DENV-1/IPC/BID-V3785/2007(1)	Dengue virus 1	GU131893	10478	Human	Cambodia
DENV-1/IPC/BID-V3786/2008(1)	Dengue virus 1	GU131894	10663	Human	Cambodia
DENV-1/IPC/BID-V3787/2009(1)	Dengue virus 1	GU131895	10474	Human	Cambodia
DENV-1/IPC/BID-V3792/2008(1)	Dengue virus 1	GQ868632	10688	Human	Cambodia
DENV-1/IPC/BID-V3793/2008(1)	Dengue virus 1	GQ868633	10691	Human	Cambodia
DENV-1/IPC/BID-V3912/2008(1)	Dengue virus 1	GU131919	10486	Human	Cambodia
DENV-1/IPC/BID-V3913/2008(1)	Dengue virus 1	GU131920	10486	Human	Cambodia
DENV-1/IPC/BID-V3914/2008(1)	Dengue virus 1	GU131921	10665	Human	Cambodia
DENV-1/IPC/BID-V3915/2008(1)	Dengue virus 1	GQ868635	10690	Human	Cambodia
DENV-1/IPC/BID-V3916/2008(1)	Dengue virus 1	GQ868636	10690	Human	Cambodia
DENV-1/IPC/BID-V3917/2008(1)	Dengue virus 1	GU131922	10486	Human	Cambodia
DENV-1/IPC/BID-V3918/2005(1)	Dengue virus 1	GU131923	10692	Human	Cambodia
DENV-1/IPC/BID-V3919/2000(1)	Dengue virus 1	GQ868637	10684	Human	Cambodia

DENV-1/IPC/BID-V3925/2006(1)	Dengue virus 1	GQ868639	10690	Human	Cambodia
DENV-1/IPC/BID-V3926/2006(1)	Dengue virus 1	GU131925	10482	Human	Cambodia
DENV-1/IPC/BID-V3927/2006(1)	Dengue virus 1	GU131926	10486	Human	Cambodia
DENV-1/KH/BID-V1978/2000(1)	Dengue virus 1	FJ639669	10690	Human	Cambodia
DENV-1/KH/BID-V1979/2001(1)	Dengue virus 1	FJ639670	10693	Human	Cambodia
DENV-1/KH/BID-V1981/2001(1)	Dengue virus 1	FJ639671	10690	Human	Cambodia
DENV-1/KH/BID-V1982/2001	Dengue virus 1	JN819423	10680	Human	Cambodia
DENV-1/KH/BID-V1983/2001(1)	Dengue virus 1	FJ639672	10682	Human	Cambodia
DENV-1/KH/BID-V1984/2001(1)	Dengue virus 1	FJ639673	10690	Human	Cambodia
DENV-1/KH/BID-V1985/2002(1)	Dengue virus 1	FJ639674	10673	Human	Cambodia
DENV-1/KH/BID-V1987/2003(1)	Dengue virus 1	FJ639675	10684	Human	Cambodia
DENV-1/KH/BID-V1988/2003(1)	Dengue virus 1	FJ639676	10690	Human	Cambodia
DENV-1/KH/BID-V1989/2003(1)	Dengue virus 1	FJ639677	10690	Human	Cambodia
DENV-1/KH/BID-V1990/2003(1)	Dengue virus 1	GQ868618	10684	Human	Cambodia
DENV-1/KH/BID-V1991/2003(1)	Dengue virus 1	GQ868619	10690	Human	Cambodia
DENV-1/KH/BID-V1992/2003(1)	Dengue virus 1	FJ639678	10690	Human	Cambodia
DENV-1/KH/BID-V1993/2003(1)	Dengue virus 1	FJ639679	10690	Human	Cambodia
DENV-1/KH/BID-V1994/2003(1)	Dengue virus 1	FJ850069	10646	Human	Cambodia
DENV-1/KH/BID-V1995/2003(1)	Dengue virus 1	FJ639680	10690	Human	Cambodia
DENV-1/KH/BID-V1996/2003(1)	Dengue virus 1	FJ639681	10676	Human	Cambodia
DENV-1/KH/BID-V1997/2004(1)	Dengue virus 1	FJ639682	10690	Human	Cambodia
DENV-1/KH/BID-V1998/2004	Dengue virus 1	JQ287664	10654	Human	Cambodia
DENV-1/KH/BID-V1999/2005(1)	Dengue virus 1	FJ639683	10674	Human	Cambodia
DENV-1/KH/BID-V2000/2005(1)	Dengue virus 1	FJ639684	10676	Human	Cambodia
DENV-1/KH/BID-V2001/2005(1)	Dengue virus 1	FJ639685	10690	Human	Cambodia
DENV-1/KH/BID-V2002/2006(1)	Dengue virus 1	FJ639686	10690	Human	Cambodia
DENV-1/KH/BID-V2003/2006(1)	Dengue virus 1	FJ744702	10690	Human	Cambodia
DENV-1/KH/BID-V2004/2006(1)	Dengue virus 1	FJ639687	10690	Human	Cambodia
DENV-1/KH/BID-V2005/2007(1)	Dengue virus 1	FJ639688	10690	Human	Cambodia
DENV-1/KH/BID-V2006/2007(1)	Dengue virus 1	FJ639689	10684	Human	Cambodia
DENV-1/KH/BID-V2007/2007(1)	Dengue virus 1	FJ639690	10690	Human	Cambodia
DENV-1/KH/BID-V2008/2007(1)	Dengue virus 1	FJ639691	10690	Human	Cambodia
DENV-1/KH/BID-V2010/2007(1)	Dengue virus 1	FJ639692	10690	Human	Cambodia
DENV-1/KH/BID-V2011/2007(1)	Dengue virus 1	FJ639693	10684	Human	Cambodia
DENV-1/KH/BID-V2012/2007(1)	Dengue virus 1	FJ639694	10684	Human	Cambodia
DENV-1/KH/BID-V2013/2007(1)	Dengue virus 1	FJ639695	10636	Human	Cambodia
DENV-1/KH/BID-V2014/2007(1)	Dengue virus 1	FJ639696	10684	Human	Cambodia
DENV-1/KH/BID-V3773/2006	Dengue virus 1	HM631852	10674	Human	Cambodia
DENV-1/KH/BID-V3784/2007	Dengue virus 1	KF955444	10407	Human	Cambodia
DENV-1/KH/BID-V4234/2006	Dengue virus 1	HM181936	10468	Human	Cambodia
DENV-1/KH/BID-V4235/2006	Dengue virus 1	HM181937	10468	Human	Cambodia
DENV-1/KH/BID-V4236/2006	Dengue virus 1	HM181938	10486	Human	Cambodia
DENV-1/KH/BID-V4237/2006	Dengue virus 1	HM181939	10690	Human	Cambodia
DENV-1/KH/BID-V4238/2006	Dengue virus 1	HM181940	10473	Human	Cambodia
DENV-1/KH/BID-V4239/2006	Dengue virus 1	HM181941	10663	Human	Cambodia
DENV-1/KH/BID-V4240/2006	Dengue virus 1	HM181942	10482	Human	Cambodia
DENV-1/KH/BID-V4241/2007	Dengue virus 1	HM181943	10672	Human	Cambodia
DENV-1/KH/BID-V4242/2007	Dengue virus 1	HM488255	10636	Human	Cambodia
DENV-1/KH/BID-V4243/2007	Dengue virus 1	HM631853	10657	Human	Cambodia
DENV-1/KH/BID-V4244/2007	Dengue virus 1	HM181944	10482	Human	Cambodia
DENV-1/KH/BID-V4245/2007	Dengue virus 1	HM181945	10685	Human	Cambodia
DENV-1/KH/BID-V4246/2007	Dengue virus 1	HM181946	10524	Human	Cambodia
DENV-1/KH/BID-V4247/2007	Dengue virus 1	HM181947	10473	Human	Cambodia
DENV-1/KH/BID-V4248/2007	Dengue virus 1	JQ287665	10520	Human	Cambodia
DENV-1/KH/BID-V4249/2007	Dengue virus 1	HM181948	10670	Human	Cambodia
DENV-1/KH/BID-V4250/2007	Dengue virus 1	HM181949	10683	Human	Cambodia
DENV-1/KH/BID-V4251/2007	Dengue virus 1	HM181950	10460	Human	Cambodia
DENV-1/KH/BID-V4252/2007	Dengue virus 1	HM181951	10468	Human	Cambodia
DENV-1/KH/BID-V4253/2007	Dengue virus 1	HM181952	10684	Human	Cambodia
DENV-1/KH/BID-V4254/2007	Dengue virus 1	HM181953	10472	Human	Cambodia
DENV-1/KH/BID-V4256/2007	Dengue virus 1	KF921933	10973	Human	Cambodia
DENV-1/KH/BID-V4257/2007	Dengue virus 1	HM181954	10679	Human	Cambodia
DENV-1/KH/BID-V4258/2007	Dengue virus 1	HM181955	10468	Human	Cambodia
DENV-1/KH/BID-V4259/2007	Dengue virus 1	HM181956	10676	Human	Cambodia
DENV-1/KH/BID-V4260/2007	Dengue virus 1	HM181957	10675	Human	Cambodia
DENV-1/KH/BID-V4261/2007	Dengue virus 1	HM181958	10678	Human	Cambodia
DENV-1/KH/BID-V4262/2007	Dengue virus 1	HM181959	10676	Human	Cambodia
DENV-1/KH/BID-V4264/2008-1	Dengue virus 1	JF937651	10727	Human	Cambodia
DENV-1/MX/BID-V3656/2006(1)	Dengue virus 1	GU131956	10690	Human	Mexico
DENV-1/MX/BID-V3657/2006(1)	Dengue virus 1	GU131957	10677	Human	Mexico
DENV-1/MX/BID-V3658/2006(1)	Dengue virus 1	GU131958	10656	Human	Mexico
DENV-1/MX/BID-V3659/2006(1)	Dengue virus 1	GQ868498	10684	Human	Mexico
DENV-1/MX/BID-V3664/2006(1)	Dengue virus 1	GQ868499	10684	Human	Mexico
DENV-1/MX/BID-V3665/2007(1)	Dengue virus 1	GQ868500	10684	Human	Mexico
DENV-1/MX/BID-V3667/2007(1)	Dengue virus 1	GU131960	10684	Human	Mexico
DENV-1/MX/BID-V3668/2007(1)	Dengue virus 1	GU131961	10688	Human	Mexico
DENV-1/MX/BID-V3669/2007(1)	Dengue virus 1	GU131962	10681	Human	Mexico
DENV-1/MX/BID-V3671/2007(1)	Dengue virus 1	GU131963	10486	Human	Mexico
DENV-1/MX/BID-V3673/2007(1)	Dengue virus 1	GU131964	10690	Human	Mexico
DENV-1/MX/BID-V3676/2007	Dengue virus 1	KF955442	10628	Human	Mexico
DENV-1/MX/BID-V3677/2007(1)	Dengue virus 1	GU131965	10665	Human	Mexico
DENV-1/MX/BID-V3679/2007(1)	Dengue virus 1	GU131966	10650	Human	Mexico
DENV-1/MX/BID-V3680/2007	Dengue virus 1	HM631855	10645	Human	Mexico
DENV-1/MX/BID-V3682/2007(1)	Dengue virus 1	GQ868501	10684	Human	Mexico
DENV-1/MX/BID-V3683/2007(1)	Dengue virus 1	HQ166035	10650	Human	Mexico
DENV-1/MX/BID-V3685/2007(1)	Dengue virus 1	GQ868502	10689	Human	Mexico
DENV-1/MX/BID-V3686/2007(1)	Dengue virus 1	GQ868503	10676	Human	Mexico
DENV-1/MX/BID-V3687/2007(1)	Dengue virus 1	GQ868504	10684	Human	Mexico
DENV-1/MX/BID-V3688/2007(1)	Dengue virus 1	GU131967	10595	Human	Mexico
DENV-1/MX/BID-V3689/2007(1)	Dengue virus 1	GQ868505	10690	Human	Mexico
DENV-1/MX/BID-V3690/2007(1)	Dengue virus 1	GU131968	10487	Human	Mexico
DENV-1/MX/BID-V3693/2007(1)	Dengue virus 1	GU131969	10676	Human	Mexico
DENV-1/MX/BID-V3694/2007(1)	Dengue virus 1	GQ868506	10684	Human	Mexico
DENV-1/MX/BID-V3696/2007(1)	Dengue virus 1	GQ868507	10659	Human	Mexico
DENV-1/MX/BID-V3697/2007(1)	Dengue virus 1	GQ868508	10684	Human	Mexico
DENV-1/MX/BID-V3698/2007(1)	Dengue virus 1	GQ868509	10684	Human	Mexico
DENV-1/MX/BID-V3699/2007(1)	Dengue virus 1	GQ868510	10684	Human	Mexico
DENV-1/MX/BID-V3700/2007(1)	Dengue virus 1	GQ868511	10680	Human	Mexico
DENV-1/MX/BID-V3701/2007(1)	Dengue virus 1	GQ868512	10674	Human	Mexico
DENV-1/MX/BID-V3703/2007(1)	Dengue virus 1	GU131970	10675	Human	Mexico
DENV-1/MX/BID-V3705/2007	Dengue virus 1	KF955427	10695	Human	Mexico
DENV-1/MX/BID-V3707/2007(1)	Dengue virus 1	GU131971	10659	Human	Mexico
DENV-1/MX/BID-V3709/2007(1)	Dengue virus 1	GQ868513	10684	Human	Mexico
DENV-1/MX/BID-V3710/2007(1)	Dengue virus 1	GQ868514	10684	Human	Mexico
DENV-1/MX/BID-V3711/2007(1)	Dengue virus 1	GU131972	10652	Human	Mexico
DENV-1/MX/BID-V3712/2007(1)	Dengue virus 1	GU131973	10560	Human	Mexico
DENV-1/MX/BID-V3721/2007(1)	Dengue virus 1	GQ868517	10690	Human	Mexico
DENV-1/MX/BID-V3722/2007(1)	Dengue virus 1	GU131976	10487	Human	Mexico
DENV-1/MX/BID-V3723/2007(1)	Dengue virus 1	GQ868518	10684	Human	Mexico
DENV-1/MX/BID-V3724/2007(1)	Dengue virus 1	GQ868519	10684	Human	Mexico
DENV-1/MX/BID-V3725/2007	Dengue virus 1	KF955443	10686	Human	Mexico
DENV-1/MX/BID-V3726/2007(1)	Dengue virus 1	GQ868520	10690	Human	Mexico
DENV-1/MX/BID-V3727/2007(1)	Dengue virus 1	GQ868521	10690	Human	Mexico

DENV-1/MX/BID-V3728/2007(1)	Dengue virus 1	GQ868522	10675	Human	Mexico
DENV-1/MX/BID-V3729/2007(1)	Dengue virus 1	GU131977	10658	Human	Mexico
DENV-1/MX/BID-V3730/2007(1)	Dengue virus 1	GQ868523	10675	Human	Mexico
DENV-1/MX/BID-V3731/2007(1)	Dengue virus 1	GQ868524	10690	Human	Mexico
DENV-1/MX/BID-V3732/2007(1)	Dengue virus 1	GU131978	10656	Human	Mexico
DENV-1/MX/BID-V3733/2007(1)	Dengue virus 1	HQ166036	10675	Human	Mexico
DENV-1/MX/BID-V3734/2007(1)	Dengue virus 1	GQ868525	10684	Human	Mexico
DENV-1/MX/BID-V3735/2007(1)	Dengue virus 1	GQ868526	10675	Human	Mexico
DENV-1/MX/BID-V3737/2007(1)	Dengue virus 1	GU131979	10661	Human	Mexico
DENV-1/MX/BID-V3739/2007(1)	Dengue virus 1	GQ868527	10683	Human	Mexico
DENV-1/MX/BID-V3741/2007(1)	Dengue virus 1	GU131980	10608	Human	Mexico
DENV-1/MX/BID-V3742/2007(1)	Dengue virus 1	GU131981	10649	Human	Mexico
DENV-1/MX/BID-V3743/2007(1)	Dengue virus 1	GQ868528	10688	Human	Mexico
DENV-1/MX/BID-V3744/2008(1)	Dengue virus 1	GQ868529	10684	Human	Mexico
DENV-1/MX/BID-V3745/2008(1)	Dengue virus 1	GU131982	10687	Human	Mexico
DENV-1/MX/BID-V3746/2008(1)	Dengue virus 1	GQ868530	10662	Human	Mexico
DENV-1/MX/BID-V3747/2008(1)	Dengue virus 1	GQ868531	10683	Human	Mexico
DENV-1/MX/BID-V3748/2008(1)	Dengue virus 1	GU131983	10690	Human	Mexico
DENV-1/MX/BID-V3749/2008(1)	Dengue virus 1	GQ868532	10681	Human	Mexico
DENV-1/MX/BID-V3752/2008(1)	Dengue virus 1	GQ868533	10690	Human	Mexico
DENV-1/MX/BID-V3753/2008(1)	Dengue virus 1	GQ868534	10684	Human	Mexico
DENV-1/MX/BID-V3754/2008(1)	Dengue virus 1	GQ868535	10690	Human	Mexico
DENV-1/MX/BID-V3756/2008(1)	Dengue virus 1	GQ868536	10684	Mosquito	Mexico
DENV-1/MX/BID-V3757/2008(1)	Dengue virus 1	HQ166037	10678	Mosquito	Mexico
DENV-1/MX/BID-V3758/2008(1)	Dengue virus 1	GQ868537	10684	Mosquito	Mexico
DENV-1/MX/BID-V3759/2008(1)	Dengue virus 1	GQ868538	10690	Mosquito	Mexico
DENV-1/MX/BID-V3760/2008(1)	Dengue virus 1	GQ868539	10663	Mosquito	Mexico
DENV-1/MX/BID-V3761/2008(1)	Dengue virus 1	GU131984	10677	Mosquito	Mexico
DENV-1/MX/BID-V7298/2011	Dengue virus 1	KJ189306	10445	Human	Mexico
DENV-1/MX/BID-V7302/2011	Dengue virus 1	KJ189307	10445	Human	Mexico
DENV-1/MX/BID-V7563/2008	Dengue virus 1	KJ189312	10444	Mosquito	Mexico
DENV-1/MX/BID-V7565/2008	Dengue virus 1	KJ189313	10445	Mosquito	Mexico
DENV-1/MX/BID-V7567/2008	Dengue virus 1	KJ189314	10450	Mosquito	Mexico
DENV-1/MX/BID-V7568/2008	Dengue virus 1	KJ189315	10448	Mosquito	Mexico
DENV-1/MX/BID-V7569/2009	Dengue virus 1	KJ189316	10445	Mosquito	Mexico
DENV-1/MX/BID-V7572/2007	Dengue virus 1	KJ189317	10448	Human	Mexico
DENV-1/MX/BID-V7576/2007	Dengue virus 1	KJ189318	10444	Human	Mexico
DENV-1/MX/BID-V7580/2007	Dengue virus 1	KJ189319	10446	Human	Mexico
DENV-1/MX/BID-V7586/2007	Dengue virus 1	KJ189320	10445	Human	Mexico
DENV-1/MX/BID-V7587/2007	Dengue virus 1	KJ189321	10444	Human	Mexico
DENV-1/MX/BID-V7588/2007	Dengue virus 1	KJ189322	10445	Human	Mexico
DENV-1/MX/BID-V7589/2007	Dengue virus 1	KJ189323	10445	Human	Mexico
DENV-1/MX/BID-V7590/2007	Dengue virus 1	KJ189324	10444	Human	Mexico
DENV-1/MX/BID-V7591/2007	Dengue virus 1	KJ189325	10444	Human	Mexico
DENV-1/MX/BID-V7592/2007	Dengue virus 1	KJ189326	10449	Human	Mexico
DENV-1/MX/BID-V7593/2007	Dengue virus 1	KJ189327	10445	Human	Mexico
DENV-1/MX/BID-V7594/2007	Dengue virus 1	KJ189328	10444	Human	Mexico
DENV-1/MX/BID-V7595/2007	Dengue virus 1	KJ189329	10449	Human	Mexico
DENV-1/MX/BID-V7596/2007	Dengue virus 1	KJ189330	10448	Human	Mexico
DENV-1/MX/BID-V7598/2008	Dengue virus 1	KJ189331	10448	Human	Mexico
DENV-1/MX/BID-V7599/2008	Dengue virus 1	KJ189332	10301	Human	Mexico
DENV-1/MX/BID-V7600/2008	Dengue virus 1	KJ189333	10445	Human	Mexico
DENV-1/MX/BID-V7601/2008	Dengue virus 1	KJ189334	10445	Human	Mexico
DENV-1/MX/BID-V7604/2008	Dengue virus 1	KJ189335	10444	Human	Mexico
DENV-1/MX/BID-V7605/2008	Dengue virus 1	KJ189336	10448	Human	Mexico
DENV-1/MX/BID-V7606/2008	Dengue virus 1	KJ189337	10446	Human	Mexico
DENV-1/MX/BID-V7607/2008	Dengue virus 1	KJ189338	10448	Human	Mexico
DENV-1/MX/BID-V7608/2008	Dengue virus 1	KJ189339	10449	Human	Mexico
DENV-1/MX/BID-V7609/2008	Dengue virus 1	KJ189340	10444	Human	Mexico
DENV-1/MX/BID-V7610/2009	Dengue virus 1	KJ189341	10445	Human	Mexico
DENV-1/MX/BID-V7611/2009	Dengue virus 1	KJ189342	10444	Human	Mexico
DENV-1/MX/BID-V7612/2009	Dengue virus 1	KJ189343	10444	Human	Mexico
DENV-1/MX/BID-V7613/2009	Dengue virus 1	KJ189344	10444	Human	Mexico
DENV-1/MX/BID-V7614/2009	Dengue virus 1	KJ189345	10444	Human	Mexico
DENV-1/MX/BID-V7615/2009	Dengue virus 1	KJ189346	10442	Human	Mexico
DENV-1/MX/BID-V7619/2009	Dengue virus 1	KJ189347	10444	Human	Mexico
DENV-1/MX/BID-V7624/2011	Dengue virus 1	KJ189348	10445	Human	Mexico
DENV-1/MX/BID-V7625/2011	Dengue virus 1	KJ189349	10384	Human	Mexico
DENV-1/MX/BID-V8195/2012	Dengue virus 1	KJ189368	10480	Human	Mexico
DENV-1/MX/BID-V8196/2011	Dengue virus 1	KJ189369	10456	Human	Mexico
DENV-1/NI/BID-V1069/2005(1)	Dengue virus 1	EU482615	10690	Human	Nicaragua
DENV-1/NI/BID-V1070/2005(1)	Dengue virus 1	EU482616	10756	Human	Nicaragua
DENV-1/NI/BID-V1071/2005(1)	Dengue virus 1	EU482617	10690	Human	Nicaragua
DENV-1/NI/BID-V1072/2005(1)	Dengue virus 1	EU482618	10690	Human	Nicaragua
DENV-1/NI/BID-V1073/2005(1)	Dengue virus 1	EU482619	10690	Human	Nicaragua
DENV-1/NI/BID-V1223/2007(1)	Dengue virus 1	FJ898433	10684	Human	Nicaragua
DENV-1/NI/BID-V2330/2008(1)	Dengue virus 1	FJ547088	10690	Human	Nicaragua
DENV-1/NI/BID-V2341/2006(1)	Dengue virus 1	FJ810419	10690	Human	Nicaragua
DENV-1/NI/BID-V2342/2006(1)	Dengue virus 1	FJ547068	10675	Human	Nicaragua
DENV-1/NI/BID-V2343/2006(1)	Dengue virus 1	FJ562104	10688	Human	Nicaragua
DENV-1/NI/BID-V2642/2004(1)	Dengue virus 1	GQ199867	10690	Human	Nicaragua
DENV-1/NI/BID-V2645/2008(1)	Dengue virus 1	GQ199857	10690	Human	Nicaragua
DENV-1/NI/BID-V2646/2008(1)	Dengue virus 1	GQ199858	10690	Human	Nicaragua
DENV-1/NI/BID-V2652/2008(1)	Dengue virus 1	GQ199859	10690	Human	Nicaragua
DENV-1/NI/BID-V5067/2009-1	Dengue virus 1	JF937644	10443	Human	Nicaragua
DENV-1/NI/BID-V5068/2009	Dengue virus 1	JQ287666	10461	Human	Nicaragua
DENV-1/NI/BID-V5071/2009-1	Dengue virus 1	JF937645	10443	Human	Nicaragua
DENV-1/NI/BID-V519/2005(1)	Dengue virus 1	FJ850114	10714	Human	Nicaragua
DENV-1/NI/BID-V534/2005	Dengue virus 1	JN819402	10695	Human	Nicaragua
DENV-1/NI/BID-V536/2005(1)	Dengue virus 1	FJ850113	10690	Human	Nicaragua
DENV-1/NI/BID-V540/2005(1)	Dengue virus 1	FJ432721	10690	Human	Nicaragua
DENV-1/NI/BID-V5504/2009-1	Dengue virus 1	JF937635	10465	Human	Nicaragua
DENV-1/NI/BID-V602/2005(1)	Dengue virus 1	FJ024483	10690	Human	Nicaragua
DENV-1/NI/BID-V604/2005(1)	Dengue virus 1	FJ024478	10690	Human	Nicaragua
DENV-1/NI/BID-V606/2005(1)	Dengue virus 1	EU596504	10690	Human	Nicaragua
DENV-1/NI/BID-V610/2005(1)	Dengue virus 1	FJ024481	10690	Human	Nicaragua
DENV-1/NI/BID-V614/2005(1)	Dengue virus 1	EU596502	10690	Human	Nicaragua
DENV-1/NI/BID-V617/2004(1)	Dengue virus 1	GQ199872	10690	Human	Nicaragua
DENV-1/NI/BID-V621/2005(1)	Dengue virus 1	FJ410290	10690	Human	Nicaragua
DENV-1/NI/BID-V624/2005(1)	Dengue virus 1	FJ432720	10690	Human	Nicaragua
DENV-1/NI/BID-V628/2005(1)	Dengue virus 1	FJ024423	10690	Human	Nicaragua
DENV-1/NI/BID-V629/2005(1)	Dengue virus 1	FJ024485	10690	Human	Nicaragua
DENV-1/NI/BID-V630/2005(1)	Dengue virus 1	FJ547089	10690	Human	Nicaragua
DENV-1/NI/BID-V635/2005(1)	Dengue virus 1	FJ024484	10690	Human	Nicaragua
DENV-1/NI/BID-V641/2005(1)	Dengue virus 1	EU596503	10690	Human	Nicaragua
DENV-1/NI/BID-V642/2006(1)	Dengue virus 1	FJ024479	10690	Human	Nicaragua
DENV-1/NI/BID-V646/2005(1)	Dengue virus 1	FJ182002	10690	Human	Nicaragua
DENV-1/NI/BID-V652/2005(1)	Dengue virus 1	FJ024482	10690	Human	Nicaragua
DENV-1/NI/BID-V653/2004(1)	Dengue virus 1	EU596501	10690	Human	Nicaragua
DENV-1/NI/BID-V656/2004(1)	Dengue virus 1	FJ898437	10678	Human	Nicaragua
DENV-1/NI/BID-V657/2005(1)	Dengue virus 1	FJ024480	10690	Human	Nicaragua
DENV-1/NI/BID-V664/2004(1)	Dengue virus 1	GQ199873	10690	Human	Nicaragua
DENV-1/NI/BID-V667/2005(1)	Dengue virus 1	FJ873814	10672	Human	Nicaragua

DENV-1/NI/BID-V669/2004(1)	Dengue virus 1	GQ199875	10690	Human	Nicaragua
DENV-1/NI/BID-V746/2006	Dengue virus 1	JN819403	10462	Human	Nicaragua
DENV-1/NI/BID-V7640/2011	Dengue virus 1	KF973453	10303	Human	Nicaragua
DENV-1/NI/BID-V7644/2012	Dengue virus 1	KF973454	10619	Human	Nicaragua
DENV-1/NI/BID-V7650/2012	Dengue virus 1	KF973455	10606	Human	Nicaragua
DENV-1/NI/BID-V7651/2012	Dengue virus 1	KF973456	10327	Human	Nicaragua
DENV-1/NI/BID-V7654/2012	Dengue virus 1	KF973457	10615	Human	Nicaragua
DENV-1/NI/BID-V7655/2012	Dengue virus 1	KF973458	10290	Human	Nicaragua
DENV-1/NI/BID-V7659/2011	Dengue virus 1	KF973459	10238	Human	Nicaragua
DENV-1/NI/BID-V7672/2011	Dengue virus 1	KF973460	10630	Human	Nicaragua
DENV-1/NI/BID-V7673/2012	Dengue virus 1	KF973461	10580	Human	Nicaragua
DENV-1/NI/BID-V7675/2011	Dengue virus 1	KF973462	10336	Human	Nicaragua
DENV-1/NI/BID-V7676/2012	Dengue virus 1	KF973463	10608	Human	Nicaragua
DENV-1/NI/BID-V7677/2012	Dengue virus 1	KF973464	10608	Human	Nicaragua
DENV-1/NI/BID-V7678/2012	Dengue virus 1	KF973465	10559	Human	Nicaragua
DENV-1/NI/BID-V7680/2012	Dengue virus 1	KF973466	10618	Human	Nicaragua
DENV-1/NI/BID-V7681/2012	Dengue virus 1	KF973467	10198	Human	Nicaragua
DENV-1/NI/BID-V7682/2012	Dengue virus 1	KF973468	10599	Human	Nicaragua
DENV-1/NI/BID-V7684/2012	Dengue virus 1	KF973469	10620	Human	Nicaragua
DENV-1/NI/BID-V7685/2012	Dengue virus 1	KF973470	10731	Human	Nicaragua
DENV-1/NI/BID-V7686/2012	Dengue virus 1	KF973471	10625	Human	Nicaragua
DENV-1/NI/BID-V7690/2012	Dengue virus 1	KF973472	10616	Human	Nicaragua
DENV-1/NI/BID-V7691/2012	Dengue virus 1	KF973473	10304	Human	Nicaragua
DENV-1/NI/BID-V7692/2012	Dengue virus 1	KF973474	10616	Human	Nicaragua
DENV-1/NI/BID-V7696/2012	Dengue virus 1	KF973475	10615	Human	Nicaragua
DENV-1/PF/BID-V2939/2001(1)	Dengue virus 1	FJ898448	10676	Human	French Polynesia
DENV-1/PH/BID-V2940/2004(1)	Dengue virus 1	GQ868602	10677	Human	Philippines
DENV-1/PR/BID-V2141/1996	Dengue virus 1	KF921911	10609	Human	Puerto Rico
DENV-1/PR/BID-V7704/2012	Dengue virus 1	KJ189350	10445	Human	Puerto Rico
DENV-1/PR/BID-V7705/2012	Dengue virus 1	KJ189351	10448	Human	Puerto Rico
DENV-1/PR/BID-V7706/2012	Dengue virus 1	KJ189352	10448	Human	Puerto Rico
DENV-1/PR/BID-V7707/2012	Dengue virus 1	KJ189353	10444	Human	Puerto Rico
DENV-1/PR/BID-V7708/2012	Dengue virus 1	KJ189354	10443	Human	Puerto Rico
DENV-1/PR/BID-V7709/2012	Dengue virus 1	KJ189355	10444	Human	Puerto Rico
DENV-1/PR/BID-V7710/2012	Dengue virus 1	KJ189356	10442	Human	Puerto Rico
DENV-1/PR/BID-V7712/2012	Dengue virus 1	KJ189357	10444	Human	Puerto Rico
DENV-1/PR/BID-V7713/2012	Dengue virus 1	KJ189358	10444	Human	Puerto Rico
DENV-1/PR/BID-V7719/2012	Dengue virus 1	KJ189359	10444	Human	Puerto Rico
DENV-1/PR/BID-V7722/2010	Dengue virus 1	KJ189360	10265	Human	Puerto Rico
DENV-1/PR/BID-V8181/2010	Dengue virus 1	KJ189361	10522	Human	Puerto Rico
DENV-1/PR/BID-V8182/2010	Dengue virus 1	KJ189362	10456	Human	Puerto Rico
DENV-1/PR/BID-V8183/2010	Dengue virus 1	KJ189363	10648	Human	Puerto Rico
DENV-1/PR/BID-V8184/2010	Dengue virus 1	KJ189364	10444	Human	Puerto Rico
DENV-1/PR/BID-V8185/2010	Dengue virus 1	KJ189365	10798	Human	Puerto Rico
DENV-1/PR/BID-V8187/2010	Dengue virus 1	KJ189366	10453	Human	Puerto Rico
DENV-1/PR/BID-V8188/2010	Dengue virus 1	KJ189367	10452	Human	Puerto Rico
DENV-1/SG/07K3640DK1/2008(1)	Dengue virus 1	GQ398255	10735	Human	Singapore
DENV-1/SV/BID-V2938/1993	Dengue virus 1	JN819417	10676	Human	El Salvador
DENV-1/TH/BID-V2269/2001(1)	Dengue virus 1	FJ687426	10676	Human	Thailand
DENV-1/TH/BID-V2270/2001(1)	Dengue virus 1	FJ687427	10672	Human	Thailand
DENV-1/TH/BID-V2271/2001(1)	Dengue virus 1	FJ687428	10677	Human	Thailand
DENV-1/TH/BID-V2272/2001(1)	Dengue virus 1	FJ687429	10676	Human	Thailand
DENV-1/TH/BID-V2273/2001(1)	Dengue virus 1	FJ850068	10676	Human	Thailand
DENV-1/TH/BID-V2274/2001(1)	Dengue virus 1	FJ687430	10676	Human	Thailand
DENV-1/TH/BID-V2275/2001(1)	Dengue virus 1	FJ687431	10676	Human	Thailand
DENV-1/TH/BID-V2276/2001(1)	Dengue virus 1	FJ687432	10690	Human	Thailand
DENV-1/TH/BID-V2277/2001(1)	Dengue virus 1	FJ687433	10673	Human	Thailand
DENV-1/THAI/606147/1960-1	Dengue virus 1	JQ922547	10395	Human	Thailand
DENV-1/US/BID-V1162/1998(1)	Dengue virus 1	EU482567	10690	Human	USA
DENV-1/US/BID-V1734/1995(1)	Dengue virus 1	FJ390374	10690	Human	USA
DENV-1/US/BID-V1738/1998(1)	Dengue virus 1	FJ390378	10690	Human	USA
DENV-1/US/BID-V1739/1998(1)	Dengue virus 1	FJ205872	10690	Human	USA
DENV-1/US/BID-V1740/1998(1)	Dengue virus 1	FJ205873	10690	Human	USA
DENV-1/US/BID-V1741/1998(1)	Dengue virus 1	FJ390379	10690	Human	USA
DENV-1/US/BID-V1742/1998(1)	Dengue virus 1	FJ390380	10690	Human	USA
DENV-1/US/BID-V1743/1995(1)	Dengue virus 1	FJ205874	10690	Human	USA
DENV-1/US/BID-V1744/1995(1)	Dengue virus 1	FJ205875	10690	Human	USA
DENV-1/US/BID-V2093/1998(1)	Dengue virus 1	FJ410173	10690	Human	USA
DENV-1/US/BID-V2094/1995(1)	Dengue virus 1	FJ410174	10690	Human	USA
DENV-1/US/BID-V2095/1994(1)	Dengue virus 1	FJ410175	10690	Human	USA
DENV-1/US/BID-V2096/1993(1)	Dengue virus 1	FJ562105	10690	Human	USA
DENV-1/US/BID-V2097/1986(1)	Dengue virus 1	FJ562106	10690	Human	USA
DENV-1/US/BID-V2127/1994(1)	Dengue virus 1	FJ410179	10690	Human	USA
DENV-1/US/BID-V2128/1995(1)	Dengue virus 1	FJ410180	10690	Human	USA
DENV-1/US/BID-V2129/1995(1)	Dengue virus 1	FJ410181	10671	Human	USA
DENV-1/US/BID-V2130/1995(1)	Dengue virus 1	FJ547086	10690	Human	USA
DENV-1/US/BID-V2131/1996(1)	Dengue virus 1	FJ410182	10690	Human	USA
DENV-1/US/BID-V2132/1993(1)	Dengue virus 1	FJ410183	10690	Human	USA
DENV-1/US/BID-V2133/1993(1)	Dengue virus 1	FJ410184	10744	Human	USA
DENV-1/US/BID-V2134/1993(1)	Dengue virus 1	FJ410185	10690	Human	USA
DENV-1/US/BID-V2135/1992(1)	Dengue virus 1	FJ547087	10690	Human	USA
DENV-1/US/BID-V2136/1992(1)	Dengue virus 1	FJ410186	10690	Human	USA
DENV-1/US/BID-V2137/1992(1)	Dengue virus 1	FJ410187	10690	Human	USA
DENV-1/US/BID-V2138/1996(1)	Dengue virus 1	FJ478457	10676	Human	USA
DENV-1/US/BID-V2139/1996(1)	Dengue virus 1	FJ410188	10690	Human	USA
DENV-1/US/BID-V2140/1996(1)	Dengue virus 1	FJ410189	10690	Human	USA
DENV-1/US/BID-V2142/1987(1)	Dengue virus 1	FJ478458	10680	Human	USA
DENV-1/US/BID-V2143/1987(1)	Dengue virus 1	FJ410190	10684	Human	USA
DENV-1/US/BID-V852/2006(1)	Dengue virus 1	EU482591	10690	Human	USA
DENV-1/US/BID-V853/1998(1)	Dengue virus 1	EU482592	10690	Human	USA
DENV-1/VE/BID-V1134/2007(1)	Dengue virus 1	EU482609	10690	Human	Venezuela
DENV-1/VE/BID-V1135/2007(1)	Dengue virus 1	EU482610	10690	Human	Venezuela
DENV-1/VE/BID-V1136/2007(1)	Dengue virus 1	EU482611	10690	Human	Venezuela
DENV-1/VE/BID-V2162/1997(1)	Dengue virus 1	FJ639735	10675	Human	Venezuela
DENV-1/VE/BID-V2168/1998(1)	Dengue virus 1	FJ639740	10684	Human	Venezuela
DENV-1/VE/BID-V2169/1998(1)	Dengue virus 1	FJ639741	10690	Human	Venezuela
DENV-1/VE/BID-V2171/1999(1)	Dengue virus 1	FJ639743	10674	Human	Venezuela
DENV-1/VE/BID-V2227/2004(1)	Dengue virus 1	FJ639794	10676	Human	Venezuela
DENV-1/VE/BID-V2229/2004(1)	Dengue virus 1	FJ639796	10676	Human	Venezuela
DENV-1/VE/BID-V2230/2004(1)	Dengue virus 1	FJ639797	10676	Human	Venezuela
DENV-1/VE/BID-V2235/2004(1)	Dengue virus 1	FJ639802	10691	Human	Venezuela
DENV-1/VE/BID-V2237/2004(1)	Dengue virus 1	FJ744701	10641	Human	Venezuela
DENV-1/VE/BID-V2241/2005	Dengue virus 1	JN819410	10486	Human	Venezuela
DENV-1/VE/BID-V2243/2007(1)	Dengue virus 1	FJ639806	10685	Human	Venezuela
DENV-1/VE/BID-V2245/2005(1)	Dengue virus 1	FJ639808	10691	Human	Venezuela
DENV-1/VE/BID-V2248/2005(1)	Dengue virus 1	FJ639811	10684	Human	Venezuela
DENV-1/VE/BID-V2249/2005	Dengue virus 1	JN819411	10493	Human	Venezuela
DENV-1/VE/BID-V2250/2005(1)	Dengue virus 1	FJ639812	10640	Human	Venezuela
DENV-1/VE/BID-V2251/2005(1)	Dengue virus 1	FJ639813	10691	Human	Venezuela
DENV-1/VE/BID-V2252/2005	Dengue virus 1	JN819412	10505	Human	Venezuela
DENV-1/VE/BID-V2253/2005(1)	Dengue virus 1	FJ810415	10685	Human	Venezuela
DENV-1/VE/BID-V2254/2005(1)	Dengue virus 1	FJ639814	10677	Human	Venezuela
DENV-1/VE/BID-V2255/2006(1)	Dengue virus 1	FJ639815	10673	Human	Venezuela

DENV-1/VE/BID-V2258/2006(1)	Dengue virus 1	FJ639818	10690	Human	Venezuela
DENV-1/VE/BID-V2259/2006(1)	Dengue virus 1	FJ639819	10684	Human	Venezuela
DENV-1/VE/BID-V2260/2006(1)	Dengue virus 1	FJ639820	10650	Human	Venezuela
DENV-1/VE/BID-V2261/2006(1)	Dengue virus 1	FJ639821	10684	Human	Venezuela
DENV-1/VE/BID-V2263/2006(1)	Dengue virus 1	FJ639823	10675	Human	Venezuela
DENV-1/VE/BID-V2264/2006(1)	Dengue virus 1	FJ639824	10691	Human	Venezuela
DENV-1/VE/BID-V2265/2006	Dengue virus 1	JN819413	10527	Human	Venezuela
DENV-1/VE/BID-V2423/2004	Dengue virus 1	JN819425	10485	Human	Venezuela
DENV-1/VE/BID-V2459/2007(1)	Dengue virus 1	FJ850099	10685	Human	Venezuela
DENV-1/VE/BID-V2461/2007(1)	Dengue virus 1	FJ850100	10690	Human	Venezuela
DENV-1/VE/BID-V2462/2007(1)	Dengue virus 1	FJ873809	10684	Human	Venezuela
DENV-1/VE/BID-V2463/2007(1)	Dengue virus 1	FJ850101	10690	Human	Venezuela
DENV-1/VE/BID-V2464/2007(1)	Dengue virus 1	FJ850102	10690	Human	Venezuela
DENV-1/VE/BID-V2465/2007(1)	Dengue virus 1	FJ882579	10674	Human	Venezuela
DENV-1/VE/BID-V2466/2007	Dengue virus 1	JN819414	10466	Human	Venezuela
DENV-1/VE/BID-V2467/2007(1)	Dengue virus 1	FJ873810	10690	Human	Venezuela
DENV-1/VE/BID-V2468/2008(1)	Dengue virus 1	FJ850103	10678	Human	Venezuela
DENV-1/VE/BID-V2469/2008(1)	Dengue virus 1	FJ850104	10690	Human	Venezuela
DENV-1/VE/BID-V2606/2006	Dengue virus 1	JN819405	10467	Human	Venezuela
DENV-1/VE/BID-V2615/2007(1)	Dengue virus 1	GQ199877	10690	Human	Venezuela
DENV-1/VE/BID-V3540/1997(1)	Dengue virus 1	GU056029	10517	Human	Venezuela
DENV-1/VE/BID-V3541/1997(1)	Dengue virus 1	GU056030	10486	Human	Venezuela
DENV-1/VE/BID-V3543/1998(1)	Dengue virus 1	GU056031	10475	Human	Venezuela
DENV-1/VE/BID-V3544/1998(1)	Dengue virus 1	GU056032	10600	Human	Venezuela
DENV-1/VE/BID-V3545/1998(1)	Dengue virus 1	GU056033	10471	Human	Venezuela
DENV-1/VE/BID-V3548/2000(1)	Dengue virus 1	GU131832	10460	Human	Venezuela
DENV-1/VE/BID-V3549/2000(1)	Dengue virus 1	GU131833	10610	Human	Venezuela
DENV-1/VE/BID-V3550/2001(1)	Dengue virus 1	GU131834	10468	Human	Venezuela
DENV-1/VE/BID-V3552/2004(1)	Dengue virus 1	GU131835	10479	Human	Venezuela
DENV-1/VE/BID-V3553/2004(1)	Dengue virus 1	GU131836	10556	Human	Venezuela
DENV-1/VE/BID-V3558/2005(1)	Dengue virus 1	GU131837	10455	Human	Venezuela
DENV-1/VE/BID-V3561/2006	Dengue virus 1	JN819415	10498	Human	Venezuela
DENV-1/VE/BID-V3562/2006(1)	Dengue virus 1	GU131838	10468	Human	Venezuela
DENV-1/VE/BID-V3565/2006(1)	Dengue virus 1	GU131839	10456	Human	Venezuela
DENV-1/VE/BID-V3573/2007(1)	Dengue virus 1	GU131840	10485	Human	Venezuela
DENV-1/VE/BID-V3576/2007(1)	Dengue virus 1	GU131841	10486	Human	Venezuela
DENV-1/VE/BID-V3578/2007(1)	Dengue virus 1	GU131842	10478	Human	Venezuela
DENV-1/VG/BID-V2937/1985(1)	Dengue virus 1	GQ868601	10690	Human	British Virgin Islands
DENV-1/VN/BID-V1319/2006(1)	Dengue virus 1	EU660396	10690	Human	Viet Nam
DENV-1/VN/BID-V1320/2006(1)	Dengue virus 1	EU660390	10690	Human	Viet Nam
DENV-1/VN/BID-V1321/2006(1)	Dengue virus 1	EU660391	10690	Human	Viet Nam
DENV-1/VN/BID-V1322/2006(1)	Dengue virus 1	EU660392	10690	Human	Viet Nam
DENV-1/VN/BID-V1323/2006(1)	Dengue virus 1	EU660393	10690	Human	Viet Nam
DENV-1/VN/BID-V1324/2006(1)	Dengue virus 1	EU660394	10690	Human	Viet Nam
DENV-1/VN/BID-V1499/2006(1)	Dengue virus 1	EU660401	10690	Human	Viet Nam
DENV-1/VN/BID-V1500/2006(1)	Dengue virus 1	FJ373305	10690	Human	Viet Nam
DENV-1/VN/BID-V1501/2006(1)	Dengue virus 1	EU660402	10690	Human	Viet Nam
DENV-1/VN/BID-V1502/2006(1)	Dengue virus 1	EU660403	10679	Human	Viet Nam
DENV-1/VN/BID-V1503/2006(1)	Dengue virus 1	EU660397	10690	Human	Viet Nam
DENV-1/VN/BID-V1504/2006(1)	Dengue virus 1	EU687247	10690	Human	Viet Nam
DENV-1/VN/BID-V1505/2007(1)	Dengue virus 1	EU660395	10690	Human	Viet Nam
DENV-1/VN/BID-V1506/2007(1)	Dengue virus 1	EU660412	10690	Human	Viet Nam
DENV-1/VN/BID-V1516/2007(1)	Dengue virus 1	EU660418	10690	Human	Viet Nam
DENV-1/VN/BID-V1521/2007(1)	Dengue virus 1	EU677150	10691	Human	Viet Nam
DENV-1/VN/BID-V1523/2007(1)	Dengue virus 1	EU677151	10690	Human	Viet Nam
DENV-1/VN/BID-V1524/2007(1)	Dengue virus 1	EU677152	10690	Human	Viet Nam
DENV-1/VN/BID-V1525/2007(1)	Dengue virus 1	EU677153	10690	Human	Viet Nam
DENV-1/VN/BID-V1526/2007(1)	Dengue virus 1	EU677154	10690	Human	Viet Nam
DENV-1/VN/BID-V1527/2007(1)	Dengue virus 1	EU677155	10690	Human	Viet Nam
DENV-1/VN/BID-V1528/2007(1)	Dengue virus 1	EU677156	10690	Human	Viet Nam
DENV-1/VN/BID-V1529/2007(1)	Dengue virus 1	EU677157	10690	Human	Viet Nam
DENV-1/VN/BID-V1530/2007(1)	Dengue virus 1	EU726777	10690	Human	Viet Nam
DENV-1/VN/BID-V1531/2007(1)	Dengue virus 1	EU677158	10690	Human	Viet Nam
DENV-1/VN/BID-V1532/2007(1)	Dengue virus 1	EU677159	10690	Human	Viet Nam
DENV-1/VN/BID-V1533/2007(1)	Dengue virus 1	EU677160	10690	Human	Viet Nam
DENV-1/VN/BID-V1534/2007(1)	Dengue virus 1	EU677161	10690	Human	Viet Nam
DENV-1/VN/BID-V1535/2007(1)	Dengue virus 1	EU677162	10691	Human	Viet Nam
DENV-1/VN/BID-V1536/2007(1)	Dengue virus 1	EU677163	10690	Human	Viet Nam
DENV-1/VN/BID-V1537/2007(1)	Dengue virus 1	EU677164	10690	Human	Viet Nam
DENV-1/VN/BID-V1538/2007(1)	Dengue virus 1	EU677165	10690	Human	Viet Nam
DENV-1/VN/BID-V1539/2007(1)	Dengue virus 1	EU677139	10690	Human	Viet Nam
DENV-1/VN/BID-V1540/2007(1)	Dengue virus 1	EU677140	10690	Human	Viet Nam
DENV-1/VN/BID-V1541/2007(1)	Dengue virus 1	EU677166	10690	Human	Viet Nam
DENV-1/VN/BID-V1542/2007(1)	Dengue virus 1	EU677167	10690	Human	Viet Nam
DENV-1/VN/BID-V1543/2007(1)	Dengue virus 1	EU677168	10690	Human	Viet Nam
DENV-1/VN/BID-V1544/2007(1)	Dengue virus 1	EU677169	10690	Human	Viet Nam
DENV-1/VN/BID-V1545/2007(1)	Dengue virus 1	EU677170	10690	Human	Viet Nam
DENV-1/VN/BID-V1546/2007(1)	Dengue virus 1	EU726778	10690	Human	Viet Nam
DENV-1/VN/BID-V1547/2007(1)	Dengue virus 1	EU677171	10690	Human	Viet Nam
DENV-1/VN/BID-V1548/2007(1)	Dengue virus 1	EU677172	10690	Human	Viet Nam
DENV-1/VN/BID-V1549/2007(1)	Dengue virus 1	EU677173	10690	Human	Viet Nam
DENV-1/VN/BID-V1550/2007(1)	Dengue virus 1	EU677174	10690	Human	Viet Nam
DENV-1/VN/BID-V1551/2007(1)	Dengue virus 1	EU726779	10690	Human	Viet Nam
DENV-1/VN/BID-V1552/2007(1)	Dengue virus 1	EU660419	10690	Human	Viet Nam
DENV-1/VN/BID-V1553/2007(1)	Dengue virus 1	EU677175	10691	Human	Viet Nam
DENV-1/VN/BID-V1555/2007(1)	Dengue virus 1	EU677176	10690	Human	Viet Nam
DENV-1/VN/BID-V1556/2007(1)	Dengue virus 1	EU726780	10690	Human	Viet Nam
DENV-1/VN/BID-V1557/2007(1)	Dengue virus 1	EU726781	10690	Human	Viet Nam
DENV-1/VN/BID-V1558/2007(1)	Dengue virus 1	EU677177	10690	Human	Viet Nam
DENV-1/VN/BID-V1559/2007(1)	Dengue virus 1	EU726782	10690	Human	Viet Nam
DENV-1/VN/BID-V1560/2007(1)	Dengue virus 1	EU677178	10690	Human	Viet Nam
DENV-1/VN/BID-V1561/2007(1)	Dengue virus 1	EU687251	10690	Human	Viet Nam
DENV-1/VN/BID-V1565/2007(1)	Dengue virus 1	FJ024425	10690	Human	Viet Nam
DENV-1/VN/BID-V1566/2007(1)	Dengue virus 1	FJ024426	10690	Human	Viet Nam
DENV-1/VN/BID-V1567/2007(1)	Dengue virus 1	FJ024427	10690	Human	Viet Nam
DENV-1/VN/BID-V1568/2007(1)	Dengue virus 1	FJ024428	10690	Human	Viet Nam
DENV-1/VN/BID-V1569/2007(1)	Dengue virus 1	FJ024429	10690	Human	Viet Nam
DENV-1/VN/BID-V1570/2007(1)	Dengue virus 1	FJ024430	10690	Human	Viet Nam
DENV-1/VN/BID-V1571/2007(1)	Dengue virus 1	FJ024431	10690	Human	Viet Nam
DENV-1/VN/BID-V1572/2007(1)	Dengue virus 1	FJ024432	10689	Human	Viet Nam
DENV-1/VN/BID-V1573/2007(1)	Dengue virus 1	FJ024433	10690	Human	Viet Nam
DENV-1/VN/BID-V1574/2007(1)	Dengue virus 1	FJ024434	10690	Human	Viet Nam
DENV-1/VN/BID-V1575/2007(1)	Dengue virus 1	FJ024435	10690	Human	Viet Nam
DENV-1/VN/BID-V1576/2007(1)	Dengue virus 1	FJ024436	10690	Human	Viet Nam
DENV-1/VN/BID-V1577/2007(1)	Dengue virus 1	FJ024437	10690	Human	Viet Nam
DENV-1/VN/BID-V1578/2007(1)	Dengue virus 1	FJ024472	10690	Human	Viet Nam
DENV-1/VN/BID-V1579/2007(1)	Dengue virus 1	FJ024438	10690	Human	Viet Nam
DENV-1/VN/BID-V1580/2007(1)	Dengue virus 1	FJ024439	10690	Human	Viet Nam
DENV-1/VN/BID-V1581/2007(1)	Dengue virus 1	FJ024440	10690	Human	Viet Nam
DENV-1/VN/BID-V1582/2007(1)	Dengue virus 1	FJ024441	10690	Human	Viet Nam
DENV-1/VN/BID-V1583/2007(1)	Dengue virus 1	FJ024442	10690	Human	Viet Nam
DENV-1/VN/BID-V1584/2007(1)	Dengue virus 1	FJ024443	10690	Human	Viet Nam
DENV-1/VN/BID-V1628/2006(1)	Dengue virus 1	FJ024444	10690	Human	Viet Nam

DENV-1/VN/BID-V1629/2006(1)	Dengue virus 1	FJ373296	10690	Human	Viet Nam
DENV-1/VN/BID-V1632/2006(1)	Dengue virus 1	FJ182018	10690	Human	Viet Nam
DENV-1/VN/BID-V1634/2006(1)	Dengue virus 1	FJ410287	10690	Human	Viet Nam
DENV-1/VN/BID-V1635/2007(1)	Dengue virus 1	FJ182019	10690	Human	Viet Nam
DENV-1/VN/BID-V1636/2007(1)	Dengue virus 1	FJ432719	10690	Human	Viet Nam
DENV-1/VN/BID-V1637/2007(1)	Dengue virus 1	FJ182020	10675	Human	Viet Nam
DENV-1/VN/BID-V1638/2007(1)	Dengue virus 1	FJ024445	10690	Human	Viet Nam
DENV-1/VN/BID-V1639/2007(1)	Dengue virus 1	FJ182021	10690	Human	Viet Nam
DENV-1/VN/BID-V1640/2007(1)	Dengue virus 1	FJ182022	10690	Human	Viet Nam
DENV-1/VN/BID-V1641/2007(1)	Dengue virus 1	FJ024446	10690	Human	Viet Nam
DENV-1/VN/BID-V1642/2007(1)	Dengue virus 1	FJ182023	10690	Human	Viet Nam
DENV-1/VN/BID-V1643/2007(1)	Dengue virus 1	FJ024447	10690	Human	Viet Nam
DENV-1/VN/BID-V1644/2007(1)	Dengue virus 1	FJ182024	10675	Human	Viet Nam
DENV-1/VN/BID-V1646/2007(1)	Dengue virus 1	FJ182025	10690	Human	Viet Nam
DENV-1/VN/BID-V1647/2007(1)	Dengue virus 1	FJ373297	10690	Human	Viet Nam
DENV-1/VN/BID-V1648/2007(1)	Dengue virus 1	FJ024448	10690	Human	Viet Nam
DENV-1/VN/BID-V1649/2007(1)	Dengue virus 1	FJ182026	10690	Human	Viet Nam
DENV-1/VN/BID-V1650/2007(1)	Dengue virus 1	FJ373298	10690	Human	Viet Nam
DENV-1/VN/BID-V1651/2007(1)	Dengue virus 1	FJ182027	10690	Human	Viet Nam
DENV-1/VN/BID-V1652/2007(1)	Dengue virus 1	FJ390381	10690	Human	Viet Nam
DENV-1/VN/BID-V1653/2007(1)	Dengue virus 1	FJ182028	10690	Human	Viet Nam
DENV-1/VN/BID-V1655/2007(1)	Dengue virus 1	FJ024449	10690	Human	Viet Nam
DENV-1/VN/BID-V1656/2007(1)	Dengue virus 1	FJ390382	10690	Human	Viet Nam
DENV-1/VN/BID-V1657/2007(1)	Dengue virus 1	FJ182003	10690	Human	Viet Nam
DENV-1/VN/BID-V1658/2007(1)	Dengue virus 1	FJ182029	10690	Human	Viet Nam
DENV-1/VN/BID-V1659/2007(1)	Dengue virus 1	FJ390383	10713	Human	Viet Nam
DENV-1/VN/BID-V1660/2007(1)	Dengue virus 1	FJ024450	10690	Human	Viet Nam
DENV-1/VN/BID-V1662/2007(1)	Dengue virus 1	FJ182030	10690	Human	Viet Nam
DENV-1/VN/BID-V1663/2007(1)	Dengue virus 1	FJ182031	10690	Human	Viet Nam
DENV-1/VN/BID-V1664/2007(1)	Dengue virus 1	FJ182032	10691	Human	Viet Nam
DENV-1/VN/BID-V1665/2007(1)	Dengue virus 1	FJ024451	10690	Human	Viet Nam
DENV-1/VN/BID-V1666/2007(1)	Dengue virus 1	FJ182033	10690	Human	Viet Nam
DENV-1/VN/BID-V1667/2007(1)	Dengue virus 1	FJ182034	10676	Human	Viet Nam
DENV-1/VN/BID-V1668/2007(1)	Dengue virus 1	FJ182035	10691	Human	Viet Nam
DENV-1/VN/BID-V1670/2007(1)	Dengue virus 1	FJ182036	10690	Human	Viet Nam
DENV-1/VN/BID-V1671/2007(1)	Dengue virus 1	FJ410289	10679	Human	Viet Nam
DENV-1/VN/BID-V1672/2007(1)	Dengue virus 1	FJ024453	10684	Human	Viet Nam
DENV-1/VN/BID-V1673/2007(1)	Dengue virus 1	FJ205876	10690	Human	Viet Nam
DENV-1/VN/BID-V1676/2007(1)	Dengue virus 1	FJ024455	10690	Human	Viet Nam
DENV-1/VN/BID-V1677/2007(1)	Dengue virus 1	FJ024456	10690	Human	Viet Nam
DENV-1/VN/BID-V1683/2007(1)	Dengue virus 1	FJ024457	10690	Human	Viet Nam
DENV-1/VN/BID-V1686/2007(1)	Dengue virus 1	FJ390386	10690	Human	Viet Nam
DENV-1/VN/BID-V1689/2007(1)	Dengue virus 1	FJ024459	10691	Human	Viet Nam
DENV-1/VN/BID-V1691/2007(1)	Dengue virus 1	FJ205881	10690	Human	Viet Nam
DENV-1/VN/BID-V1692/2007(1)	Dengue virus 1	FJ024460	10690	Human	Viet Nam
DENV-1/VN/BID-V1693/2007(1)	Dengue virus 1	FJ205882	10684	Human	Viet Nam
DENV-1/VN/BID-V1694/2007(1)	Dengue virus 1	FJ205883	10690	Human	Viet Nam
DENV-1/VN/BID-V1698/2007(1)	Dengue virus 1	FJ205884	10690	Human	Viet Nam
DENV-1/VN/BID-V1700/2007(1)	Dengue virus 1	FJ024462	10690	Human	Viet Nam
DENV-1/VN/BID-V1701/2007(1)	Dengue virus 1	FJ024463	10690	Human	Viet Nam
DENV-1/VN/BID-V1702/2007(1)	Dengue virus 1	FJ390388	10690	Human	Viet Nam
DENV-1/VN/BID-V1703/2007(1)	Dengue virus 1	FJ024464	10684	Human	Viet Nam
DENV-1/VN/BID-V1768/2008(1)	Dengue virus 1	FJ410191	10674	Human	Viet Nam
DENV-1/VN/BID-V1771/2007(1)	Dengue virus 1	FJ432723	10690	Human	Viet Nam
DENV-1/VN/BID-V1772/2008(1)	Dengue virus 1	FJ410192	10658	Human	Viet Nam
DENV-1/VN/BID-V1774/2007(1)	Dengue virus 1	FJ410194	10681	Human	Viet Nam
DENV-1/VN/BID-V1779/2007(1)	Dengue virus 1	FJ432725	10676	Human	Viet Nam
DENV-1/VN/BID-V1782/2007(1)	Dengue virus 1	FJ432727	10688	Human	Viet Nam
DENV-1/VN/BID-V1784/2007(1)	Dengue virus 1	FJ432729	10689	Human	Viet Nam
DENV-1/VN/BID-V1785/2007(1)	Dengue virus 1	FJ432730	10688	Human	Viet Nam
DENV-1/VN/BID-V1787/2007(1)	Dengue virus 1	FJ432732	10713	Human	Viet Nam
DENV-1/VN/BID-V1788/2007(1)	Dengue virus 1	FJ432733	10689	Human	Viet Nam
DENV-1/VN/BID-V1789/2007(1)	Dengue virus 1	FJ410196	10689	Human	Viet Nam
DENV-1/VN/BID-V1791/2007(1)	Dengue virus 1	FJ410197	10683	Human	Viet Nam
DENV-1/VN/BID-V1792/2007(1)	Dengue virus 1	FJ432734	10689	Human	Viet Nam
DENV-1/VN/BID-V1793/2007(1)	Dengue virus 1	FJ432735	10690	Human	Viet Nam
DENV-1/VN/BID-V1795/2007(1)	Dengue virus 1	FJ432736	10690	Human	Viet Nam
DENV-1/VN/BID-V1797/2007(1)	Dengue virus 1	FJ410198	10675	Human	Viet Nam
DENV-1/VN/BID-V1798/2007(1)	Dengue virus 1	FJ432737	10690	Human	Viet Nam
DENV-1/VN/BID-V1799/2007(1)	Dengue virus 1	FJ410199	10676	Human	Viet Nam
DENV-1/VN/BID-V1800/2007(1)	Dengue virus 1	FJ432738	10689	Human	Viet Nam
DENV-1/VN/BID-V1802/2007(1)	Dengue virus 1	FJ432739	10690	Human	Viet Nam
DENV-1/VN/BID-V1805/2007(1)	Dengue virus 1	FJ432740	10682	Human	Viet Nam
DENV-1/VN/BID-V1806/2007(1)	Dengue virus 1	FJ410201	10674	Human	Viet Nam
DENV-1/VN/BID-V1808/2007(1)	Dengue virus 1	FJ410203	10686	Human	Viet Nam
DENV-1/VN/BID-V1809/2007(1)	Dengue virus 1	FJ410204	10671	Human	Viet Nam
DENV-1/VN/BID-V1811/2007(1)	Dengue virus 1	FJ432742	10671	Human	Viet Nam
DENV-1/VN/BID-V1812/2007(1)	Dengue virus 1	FJ410205	10676	Human	Viet Nam
DENV-1/VN/BID-V1813/2007(1)	Dengue virus 1	FJ410206	10694	Human	Viet Nam
DENV-1/VN/BID-V1814/2007(1)	Dengue virus 1	FJ410207	10683	Human	Viet Nam
DENV-1/VN/BID-V1820/2007(1)	Dengue virus 1	FJ432744	10690	Human	Viet Nam
DENV-1/VN/BID-V1822/2007(1)	Dengue virus 1	FJ547060	10684	Human	Viet Nam
DENV-1/VN/BID-V1823/2007(1)	Dengue virus 1	FJ432745	10680	Human	Viet Nam
DENV-1/VN/BID-V1825/2007(1)	Dengue virus 1	FJ410209	10678	Human	Viet Nam
DENV-1/VN/BID-V1826/2007(1)	Dengue virus 1	FJ410210	10672	Human	Viet Nam
DENV-1/VN/BID-V1827/2007(1)	Dengue virus 1	FJ432746	10684	Human	Viet Nam
DENV-1/VN/BID-V1830/2007(1)	Dengue virus 1	FJ410211	10672	Human	Viet Nam
DENV-1/VN/BID-V1832/2007(1)	Dengue virus 1	FJ432747	10690	Human	Viet Nam
DENV-1/VN/BID-V1834/2007(1)	Dengue virus 1	FJ432748	10675	Human	Viet Nam
DENV-1/VN/BID-V1836/2007(1)	Dengue virus 1	FJ432749	10690	Human	Viet Nam
DENV-1/VN/BID-V1838/2007(1)	Dengue virus 1	FJ461306	10690	Human	Viet Nam
DENV-1/VN/BID-V1839/2007(1)	Dengue virus 1	FJ410212	10672	Human	Viet Nam
DENV-1/VN/BID-V1840/2008(1)	Dengue virus 1	FJ461307	10689	Human	Viet Nam
DENV-1/VN/BID-V1841/2008(1)	Dengue virus 1	FJ410213	10670	Human	Viet Nam
DENV-1/VN/BID-V1842/2008(1)	Dengue virus 1	FJ461308	10674	Human	Viet Nam
DENV-1/VN/BID-V1843/2008(1)	Dengue virus 1	FJ410214	10672	Human	Viet Nam
DENV-1/VN/BID-V1846/2008(1)	Dengue virus 1	FJ461310	10676	Human	Viet Nam
DENV-1/VN/BID-V1849/2007(1)	Dengue virus 1	FJ410216	10653	Human	Viet Nam
DENV-1/VN/BID-V1850/2007(1)	Dengue virus 1	FJ562101	10690	Human	Viet Nam
DENV-1/VN/BID-V1851/2007(1)	Dengue virus 1	FJ461312	10690	Human	Viet Nam
DENV-1/VN/BID-V1852/2007(1)	Dengue virus 1	FJ906728	10676	Human	Viet Nam
DENV-1/VN/BID-V1854/2007(1)	Dengue virus 1	FJ461313	10690	Human	Viet Nam
DENV-1/VN/BID-V1855/2007(1)	Dengue virus 1	FJ547063	10672	Human	Viet Nam
DENV-1/VN/BID-V1856/2007(1)	Dengue virus 1	FJ410218	10676	Human	Viet Nam
DENV-1/VN/BID-V1859/2007(1)	Dengue virus 1	FJ461315	10690	Human	Viet Nam
DENV-1/VN/BID-V1860/2007(1)	Dengue virus 1	FJ461316	10690	Human	Viet Nam
DENV-1/VN/BID-V1861/2007(1)	Dengue virus 1	FJ461317	10690	Human	Viet Nam
DENV-1/VN/BID-V1862/2008(1)	Dengue virus 1	FJ410220	10672	Human	Viet Nam
DENV-1/VN/BID-V1865/2007-1	Dengue virus 1	JF937649	10443	Human	Viet Nam
DENV-1/VN/BID-V1866/2007(1)	Dengue virus 1	FJ410222	10669	Human	Viet Nam
DENV-1/VN/BID-V1867/2007(1)	Dengue virus 1	FJ461318	10690	Human	Viet Nam
DENV-1/VN/BID-V1870/2007(1)	Dengue virus 1	FJ461319	10690	Human	Viet Nam
DENV-1/VN/BID-V1871/2007(1)	Dengue virus 1	FJ461320	10690	Human	Viet Nam

DENV-1/VN/BID-V1875/2007(1)	Dengue virus 1	FJ410225	10674	Human	Viet Nam
DENV-1/VN/BID-V1878/2008(1)	Dengue virus 1	FJ410226	10690	Human	Viet Nam
DENV-1/VN/BID-V1879/2007(1)	Dengue virus 1	FJ410227	10680	Human	Viet Nam
DENV-1/VN/BID-V1880/2007(1)	Dengue virus 1	FJ859029	10676	Human	Viet Nam
DENV-1/VN/BID-V1883/2007(1)	Dengue virus 1	FJ547065	10674	Human	Viet Nam
DENV-1/VN/BID-V1884/2008(1)	Dengue virus 1	FJ410230	10676	Human	Viet Nam
DENV-1/VN/BID-V1885/2007(1)	Dengue virus 1	FJ410231	10671	Human	Viet Nam
DENV-1/VN/BID-V1887/2007(1)	Dengue virus 1	FJ461323	10676	Human	Viet Nam
DENV-1/VN/BID-V1888/2007(1)	Dengue virus 1	FJ461324	10690	Human	Viet Nam
DENV-1/VN/BID-V1889/2007(1)	Dengue virus 1	FJ461325	10677	Human	Viet Nam
DENV-1/VN/BID-V1890/2007(1)	Dengue virus 1	FJ410232	10679	Human	Viet Nam
DENV-1/VN/BID-V1892/2007(1)	Dengue virus 1	FJ461327	10676	Human	Viet Nam
DENV-1/VN/BID-V1893/2007(1)	Dengue virus 1	FJ461328	10690	Human	Viet Nam
DENV-1/VN/BID-V1896/2007(1)	Dengue virus 1	FJ410234	10690	Human	Viet Nam
DENV-1/VN/BID-V1898/2008(1)	Dengue virus 1	FJ410235	10679	Human	Viet Nam
DENV-1/VN/BID-V1899/2008(1)	Dengue virus 1	FJ410236	10690	Human	Viet Nam
DENV-1/VN/BID-V1901/2008(1)	Dengue virus 1	FJ410238	10690	Human	Viet Nam
DENV-1/VN/BID-V1902/2008(1)	Dengue virus 1	FJ410239	10690	Human	Viet Nam
DENV-1/VN/BID-V1904/2008(1)	Dengue virus 1	FJ410240	10690	Human	Viet Nam
DENV-1/VN/BID-V1906/2008(1)	Dengue virus 1	FJ410242	10690	Human	Viet Nam
DENV-1/VN/BID-V1907/2008(1)	Dengue virus 1	FJ410243	10690	Human	Viet Nam
DENV-1/VN/BID-V1908/2008(1)	Dengue virus 1	FJ410244	10690	Human	Viet Nam
DENV-1/VN/BID-V1909/2008(1)	Dengue virus 1	FJ410245	10684	Human	Viet Nam
DENV-1/VN/BID-V1910/2008(1)	Dengue virus 1	FJ461330	10635	Human	Viet Nam
DENV-1/VN/BID-V1912/2008(1)	Dengue virus 1	FJ410246	10688	Human	Viet Nam
DENV-1/VN/BID-V1913/2008(1)	Dengue virus 1	FJ410247	10690	Human	Viet Nam
DENV-1/VN/BID-V1914/2008(1)	Dengue virus 1	FJ410248	10690	Human	Viet Nam
DENV-1/VN/BID-V1915/2008(1)	Dengue virus 1	FJ410249	10690	Human	Viet Nam
DENV-1/VN/BID-V1917/2007(1)	Dengue virus 1	FJ410250	10688	Human	Viet Nam
DENV-1/VN/BID-V1918/2008(1)	Dengue virus 1	FJ410251	10690	Human	Viet Nam
DENV-1/VN/BID-V1919/2007(1)	Dengue virus 1	FJ410252	10684	Human	Viet Nam
DENV-1/VN/BID-V1920/2007(1)	Dengue virus 1	FJ410253	10690	Human	Viet Nam
DENV-1/VN/BID-V1921/2008(1)	Dengue virus 1	FJ410254	10690	Human	Viet Nam
DENV-1/VN/BID-V1922/2008(1)	Dengue virus 1	FJ461331	10690	Human	Viet Nam
DENV-1/VN/BID-V1923/2008(1)	Dengue virus 1	FJ410255	10690	Human	Viet Nam
DENV-1/VN/BID-V1924/2008(1)	Dengue virus 1	FJ410256	10690	Human	Viet Nam
DENV-1/VN/BID-V1925/2008(1)	Dengue virus 1	FJ410257	10690	Human	Viet Nam
DENV-1/VN/BID-V1926/2008(1)	Dengue virus 1	FJ461332	10690	Human	Viet Nam
DENV-1/VN/BID-V1927/2008(1)	Dengue virus 1	FJ410258	10690	Human	Viet Nam
DENV-1/VN/BID-V1931/2007(1)	Dengue virus 1	FJ461333	10690	Human	Viet Nam
DENV-1/VN/BID-V1932/2008(1)	Dengue virus 1	FJ410260	10690	Human	Viet Nam
DENV-1/VN/BID-V1934/2008-1	Dengue virus 1	JF937650	10454	Human	Viet Nam
DENV-1/VN/BID-V1935/2008(1)	Dengue virus 1	FJ410261	10690	Human	Viet Nam
DENV-1/VN/BID-V1937/2008(1)	Dengue virus 1	FJ461335	10690	Human	Viet Nam
DENV-1/VN/BID-V1939/2008(1)	Dengue virus 1	FJ410262	10690	Human	Viet Nam
DENV-1/VN/BID-V1940/2008(1)	Dengue virus 1	FJ410263	10684	Human	Viet Nam
DENV-1/VN/BID-V1941/2008(1)	Dengue virus 1	FJ410264	10685	Human	Viet Nam
DENV-1/VN/BID-V1942/2008(1)	Dengue virus 1	FJ410265	10690	Human	Viet Nam
DENV-1/VN/BID-V1945/2008(1)	Dengue virus 1	FJ461336	10690	Human	Viet Nam
DENV-1/VN/BID-V1947/2008(1)	Dengue virus 1	FJ410266	10688	Human	Viet Nam
DENV-1/VN/BID-V1948/2008(1)	Dengue virus 1	FJ410267	10690	Human	Viet Nam
DENV-1/VN/BID-V1949/2008(1)	Dengue virus 1	FJ410268	10690	Human	Viet Nam
DENV-1/VN/BID-V1950/2008(1)	Dengue virus 1	FJ410269	10688	Human	Viet Nam
DENV-1/VN/BID-V1951/2008(1)	Dengue virus 1	FJ410270	10688	Human	Viet Nam
DENV-1/VN/BID-V1954/2008(1)	Dengue virus 1	FJ410272	10690	Human	Viet Nam
DENV-1/VN/BID-V1955/2008(1)	Dengue virus 1	FJ410273	10690	Human	Viet Nam
DENV-1/VN/BID-V1956/2008(1)	Dengue virus 1	FJ410274	10685	Human	Viet Nam
DENV-1/VN/BID-V1958/2008(1)	Dengue virus 1	FJ410275	10721	Human	Viet Nam
DENV-1/VN/BID-V1960/2008(1)	Dengue virus 1	FJ410276	10690	Human	Viet Nam
DENV-1/VN/BID-V1961/2008(1)	Dengue virus 1	FJ410277	10690	Human	Viet Nam
DENV-1/VN/BID-V1962/2008(1)	Dengue virus 1	FJ410278	10690	Human	Viet Nam
DENV-1/VN/BID-V1963/2008	Dengue virus 1	JQ287667	10962	Human	Viet Nam
DENV-1/VN/BID-V1965/2008(1)	Dengue virus 1	FJ410279	10690	Human	Viet Nam
DENV-1/VN/BID-V1966/2008(1)	Dengue virus 1	FJ410280	10690	Human	Viet Nam
DENV-1/VN/BID-V1967/2008(1)	Dengue virus 1	FJ410281	10690	Human	Viet Nam
DENV-1/VN/BID-V1968/2008(1)	Dengue virus 1	FJ410282	10690	Human	Viet Nam
DENV-1/VN/BID-V1970/2008(1)	Dengue virus 1	FJ410283	10688	Human	Viet Nam
DENV-1/VN/BID-V1971/2008(1)	Dengue virus 1	FJ410284	10690	Human	Viet Nam
DENV-1/VN/BID-V1972/2008(1)	Dengue virus 1	FJ410285	10690	Human	Viet Nam
DENV-1/VN/BID-V1974/2008(1)	Dengue virus 1	FJ410286	10690	Human	Viet Nam
DENV-1/VN/BID-V1975/2008(1)	Dengue virus 1	FJ461339	10690	Human	Viet Nam
DENV-1/VN/BID-V1976/2008(1)	Dengue virus 1	FJ461340	10690	Human	Viet Nam
DENV-1/VN/BID-V1977/2008(1)	Dengue virus 1	FJ461341	10690	Human	Viet Nam
DENV-1/VN/BID-V2684/2006(1)	Dengue virus 1	FJ882515	10688	Human	Viet Nam
DENV-1/VN/BID-V2685/2006(1)	Dengue virus 1	FJ882516	10690	Human	Viet Nam
DENV-1/VN/BID-V2686/2006(1)	Dengue virus 1	FJ882517	10688	Human	Viet Nam
DENV-1/VN/BID-V2687/2006(1)	Dengue virus 1	GQ199771	10684	Human	Viet Nam
DENV-1/VN/BID-V2688/2006	Dengue virus 1	KF921934	10447	Human	Viet Nam
DENV-1/VN/BID-V2689/2006	Dengue virus 1	JQ287660	10766	Human	Viet Nam
DENV-1/VN/BID-V2690/2006	Dengue virus 1	JQ287661	10794	Human	Viet Nam
DENV-1/VN/BID-V2691/2006(1)	Dengue virus 1	FJ882518	10684	Human	Viet Nam
DENV-1/VN/BID-V2693/2006(1)	Dengue virus 1	FJ882519	10690	Human	Viet Nam
DENV-1/VN/BID-V2694/2006(1)	Dengue virus 1	FJ882520	10690	Human	Viet Nam
DENV-1/VN/BID-V2695/2006(1)	Dengue virus 1	FJ882521	10690	Human	Viet Nam
DENV-1/VN/BID-V2696/2006(1)	Dengue virus 1	FJ882522	10690	Human	Viet Nam
DENV-1/VN/BID-V2697/2006(1)	Dengue virus 1	FJ882523	10690	Human	Viet Nam
DENV-1/VN/BID-V2698/2006(1)	Dengue virus 1	FJ882524	10690	Human	Viet Nam
DENV-1/VN/BID-V2699/2006(1)	Dengue virus 1	FJ882525	10690	Human	Viet Nam
DENV-1/VN/BID-V2700/2006(1)	Dengue virus 1	FJ882526	10690	Human	Viet Nam
DENV-1/VN/BID-V2701/2006(1)	Dengue virus 1	FJ882527	10690	Human	Viet Nam
DENV-1/VN/BID-V2702/2006(1)	Dengue virus 1	FJ882528	10690	Human	Viet Nam
DENV-1/VN/BID-V2703/2006(1)	Dengue virus 1	FJ882529	10690	Human	Viet Nam
DENV-1/VN/BID-V2704/2006(1)	Dengue virus 1	FJ882530	10690	Human	Viet Nam
DENV-1/VN/BID-V2705/2006(1)	Dengue virus 1	FJ882531	10690	Human	Viet Nam
DENV-1/VN/BID-V2706/2006(1)	Dengue virus 1	FJ882532	10690	Human	Viet Nam
DENV-1/VN/BID-V2707/2006(1)	Dengue virus 1	GQ868605	10690	Human	Viet Nam
DENV-1/VN/BID-V2708/2006(1)	Dengue virus 1	FJ882533	10690	Human	Viet Nam
DENV-1/VN/BID-V2709/2006(1)	Dengue virus 1	FJ882534	10690	Human	Viet Nam
DENV-1/VN/BID-V2710/2006(1)	Dengue virus 1	FJ882535	10690	Human	Viet Nam
DENV-1/VN/BID-V2711/2006(1)	Dengue virus 1	FJ882536	10690	Human	Viet Nam
DENV-1/VN/BID-V2712/2006(1)	Dengue virus 1	FJ882537	10690	Human	Viet Nam
DENV-1/VN/BID-V2713/2006(1)	Dengue virus 1	GQ868606	10690	Human	Viet Nam
DENV-1/VN/BID-V2714/2006(1)	Dengue virus 1	FJ882538	10690	Human	Viet Nam
DENV-1/VN/BID-V2715/2006(1)	Dengue virus 1	FJ882539	10690	Human	Viet Nam
DENV-1/VN/BID-V2716/2006(1)	Dengue virus 1	FJ882540	10690	Human	Viet Nam
DENV-1/VN/BID-V2717/2006(1)	Dengue virus 1	FJ882541	10690	Human	Viet Nam
DENV-1/VN/BID-V2718/2006(1)	Dengue virus 1	GQ199772	10690	Human	Viet Nam
DENV-1/VN/BID-V2719/2006(1)	Dengue virus 1	FJ882542	10684	Human	Viet Nam
DENV-1/VN/BID-V2720/2006(1)	Dengue virus 1	FJ882543	10690	Human	Viet Nam
DENV-1/VN/BID-V2721/2006(1)	Dengue virus 1	FJ882544	10673	Human	Viet Nam
DENV-1/VN/BID-V2722/2007(1)	Dengue virus 1	FJ898371	10674	Human	Viet Nam
DENV-1/VN/BID-V2723/2007(1)	Dengue virus 1	FJ882545	10690	Human	Viet Nam
DENV-1/VN/BID-V2724/2007(1)	Dengue virus 1	FJ898372	10690	Human	Viet Nam

DENV-1/VN/BID-V2725/2007(1)	Dengue virus 1	FJ898373	10680	Human	Viet Nam
DENV-1/VN/BID-V2726/2007(1)	Dengue virus 1	FJ898374	10674	Human	Viet Nam
DENV-1/VN/BID-V2727/2007(1)	Dengue virus 1	FJ898375	10690	Human	Viet Nam
DENV-1/VN/BID-V2728/2007(1)	Dengue virus 1	FJ898376	10689	Human	Viet Nam
DENV-1/VN/BID-V2729/2007(1)	Dengue virus 1	FJ898377	10690	Human	Viet Nam
DENV-1/VN/BID-V2730/2007(1)	Dengue virus 1	FJ898378	10677	Human	Viet Nam
DENV-1/VN/BID-V2731/2007(1)	Dengue virus 1	FJ898379	10674	Human	Viet Nam
DENV-1/VN/BID-V2732/2007(1)	Dengue virus 1	GQ199773	10676	Human	Viet Nam
DENV-1/VN/BID-V2733/2007(1)	Dengue virus 1	GQ868607	10690	Human	Viet Nam
DENV-1/VN/BID-V2734/2007-1	Dengue virus 1	JF937596	10444	Human	Viet Nam
DENV-1/VN/BID-V2735/2007(1)	Dengue virus 1	FJ906963	10675	Human	Viet Nam
DENV-1/VN/BID-V2736/2007(1)	Dengue virus 1	FJ906964	10672	Human	Viet Nam
DENV-1/VN/BID-V2737/2007(1)	Dengue virus 1	FJ906965	10676	Human	Viet Nam
DENV-1/VN/BID-V2738/2007(1)	Dengue virus 1	FJ898380	10676	Human	Viet Nam
DENV-1/VN/BID-V2739/2007(1)	Dengue virus 1	FJ898381	10680	Human	Viet Nam
DENV-1/VN/BID-V2740/2007(1)	Dengue virus 1	FJ898382	10690	Human	Viet Nam
DENV-1/VN/BID-V2741/2007(1)	Dengue virus 1	FJ898383	10690	Human	Viet Nam
DENV-1/VN/BID-V2742/2007(1)	Dengue virus 1	FJ898384	10690	Human	Viet Nam
DENV-1/VN/BID-V2743/2007(1)	Dengue virus 1	FJ898385	10690	Human	Viet Nam
DENV-1/VN/BID-V2745/2007(1)	Dengue virus 1	GQ868608	10690	Human	Viet Nam
DENV-1/VN/BID-V2746/2007(1)	Dengue virus 1	GQ199774	10684	Human	Viet Nam
DENV-1/VN/BID-V2747/2007(1)	Dengue virus 1	GQ199775	10690	Human	Viet Nam
DENV-1/VN/BID-V2748/2007	Dengue virus 1	JQ287662	10904	Human	Viet Nam
DENV-1/VN/BID-V2749/2007(1)	Dengue virus 1	FJ882546	10681	Human	Viet Nam
DENV-1/VN/BID-V2750/2007	Dengue virus 1	KF921935	10528	Human	Viet Nam
DENV-1/VN/BID-V2751/2007(1)	Dengue virus 1	GQ199776	10676	Human	Viet Nam
DENV-1/VN/BID-V2752/2007(1)	Dengue virus 1	GQ199777	10690	Human	Viet Nam
DENV-1/VN/BID-V2753/2007	Dengue virus 1	JQ287663	11195	Human	Viet Nam
DENV-1/VN/BID-V2754/2007(1)	Dengue virus 1	GQ199778	10690	Human	Viet Nam
DENV-1/VN/BID-V2755/2007(1)	Dengue virus 1	GQ199779	10690	Human	Viet Nam
DENV-1/VN/BID-V2756/2007(1)	Dengue virus 1	GQ199780	10690	Human	Viet Nam
DENV-1/VN/BID-V2757/2007(1)	Dengue virus 1	GQ199781	10690	Human	Viet Nam
DENV-1/VN/BID-V2758/2007(1)	Dengue virus 1	GQ199782	10690	Human	Viet Nam
DENV-1/VN/BID-V2759/2007(1)	Dengue virus 1	GQ199783	10690	Human	Viet Nam
DENV-1/VN/BID-V2760/2007(1)	Dengue virus 1	GQ199784	10689	Human	Viet Nam
DENV-1/VN/BID-V2761/2007(1)	Dengue virus 1	GQ199785	10690	Human	Viet Nam
DENV-1/VN/BID-V2762/2007(1)	Dengue virus 1	GQ199786	10690	Human	Viet Nam
DENV-1/VN/BID-V2763/2007(1)	Dengue virus 1	GQ199787	10690	Human	Viet Nam
DENV-1/VN/BID-V2764/2007(1)	Dengue virus 1	GQ199788	10690	Human	Viet Nam
DENV-1/VN/BID-V2765/2007(1)	Dengue virus 1	GQ199789	10690	Human	Viet Nam
DENV-1/VN/BID-V2766/2007(1)	Dengue virus 1	GQ199790	10689	Human	Viet Nam
DENV-1/VN/BID-V2767/2007(1)	Dengue virus 1	GQ199791	10690	Human	Viet Nam
DENV-1/VN/BID-V2768/2007(1)	Dengue virus 1	GQ199792	10685	Human	Viet Nam
DENV-1/VN/BID-V2769/2007(1)	Dengue virus 1	GQ199793	10684	Human	Viet Nam
DENV-1/VN/BID-V2770/2007(1)	Dengue virus 1	GQ199794	10684	Human	Viet Nam
DENV-1/VN/BID-V2771/2007(1)	Dengue virus 1	GQ199795	10690	Human	Viet Nam
DENV-1/VN/BID-V2772/2007(1)	Dengue virus 1	FJ882547	10664	Human	Viet Nam
DENV-1/VN/BID-V2773/2007(1)	Dengue virus 1	GQ199796	10690	Human	Viet Nam
DENV-1/VN/BID-V2774/2007(1)	Dengue virus 1	GQ199797	10690	Human	Viet Nam
DENV-1/VN/BID-V2775/2007(1)	Dengue virus 1	GQ199798	10690	Human	Viet Nam
DENV-1/VN/BID-V2776/2007(1)	Dengue virus 1	GQ199799	10690	Human	Viet Nam
DENV-1/VN/BID-V2777/2007(1)	Dengue virus 1	GQ199800	10690	Human	Viet Nam
DENV-1/VN/BID-V2778/2007(1)	Dengue virus 1	FJ882548	10684	Human	Viet Nam
DENV-1/VN/BID-V2779/2007(1)	Dengue virus 1	GQ199801	10690	Human	Viet Nam
DENV-1/VN/BID-V2780/2007(1)	Dengue virus 1	GQ199802	10690	Human	Viet Nam
DENV-1/VN/BID-V2781/2007-1	Dengue virus 1	JF937597	10379	Human	Viet Nam
DENV-1/VN/BID-V2782/2007(1)	Dengue virus 1	GQ868609	10690	Human	Viet Nam
DENV-1/VN/BID-V2783/2007(1)	Dengue virus 1	GQ199803	10690	Human	Viet Nam
DENV-1/VN/BID-V2784/2007(1)	Dengue virus 1	GQ199804	10679	Human	Viet Nam
DENV-1/VN/BID-V2785/2007(1)	Dengue virus 1	GQ199805	10689	Human	Viet Nam
DENV-1/VN/BID-V2786/2007(1)	Dengue virus 1	GQ199806	10689	Human	Viet Nam
DENV-1/VN/BID-V2787/2007(1)	Dengue virus 1	GQ199807	10690	Human	Viet Nam
DENV-1/VN/BID-V2788/2007(1)	Dengue virus 1	GQ199808	10690	Human	Viet Nam
DENV-1/VN/BID-V2789/2007(1)	Dengue virus 1	GQ199809	10690	Human	Viet Nam
DENV-1/VN/BID-V2790/2007(1)	Dengue virus 1	GQ199810	10690	Human	Viet Nam
DENV-1/VN/BID-V2791/2007(1)	Dengue virus 1	GQ199811	10690	Human	Viet Nam
DENV-1/VN/BID-V2792/2007(1)	Dengue virus 1	GQ199812	10690	Human	Viet Nam
DENV-1/VN/BID-V2793/2007(1)	Dengue virus 1	GQ199813	10690	Human	Viet Nam
DENV-1/VN/BID-V2794/2007(1)	Dengue virus 1	FJ882549	10665	Human	Viet Nam
DENV-1/VN/BID-V2795/2007(1)	Dengue virus 1	GQ199814	10690	Human	Viet Nam
DENV-1/VN/BID-V2796/2007(1)	Dengue virus 1	GQ199815	10690	Human	Viet Nam
DENV-1/VN/BID-V2797/2007(1)	Dengue virus 1	GQ199816	10690	Human	Viet Nam
DENV-1/VN/BID-V2798/2007(1)	Dengue virus 1	GQ199817	10690	Human	Viet Nam
DENV-1/VN/BID-V2799/2007(1)	Dengue virus 1	GQ199818	10690	Human	Viet Nam
DENV-1/VN/BID-V2800/2007(1)	Dengue virus 1	FJ882550	10690	Human	Viet Nam
DENV-1/VN/BID-V2801/2007(1)	Dengue virus 1	FJ882551	10690	Human	Viet Nam
DENV-1/VN/BID-V2802/2007(1)	Dengue virus 1	GQ199819	10690	Human	Viet Nam
DENV-1/VN/BID-V2803/2007(1)	Dengue virus 1	FJ882552	10690	Human	Viet Nam
DENV-1/VN/BID-V2804/2007(1)	Dengue virus 1	FJ882553	10680	Human	Viet Nam
DENV-1/VN/BID-V2805/2007(1)	Dengue virus 1	FJ882554	10676	Human	Viet Nam
DENV-1/VN/BID-V2806/2007(1)	Dengue virus 1	GQ868610	10690	Human	Viet Nam
DENV-1/VN/BID-V2807/2007(1)	Dengue virus 1	GQ868611	10690	Human	Viet Nam
DENV-1/VN/BID-V2808/2007(1)	Dengue virus 1	GQ199820	10690	Human	Viet Nam
DENV-1/VN/BID-V2809/2007(1)	Dengue virus 1	FJ882555	10678	Human	Viet Nam
DENV-1/VN/BID-V2810/2007(1)	Dengue virus 1	GQ199821	10690	Human	Viet Nam
DENV-1/VN/BID-V2811/2007(1)	Dengue virus 1	FJ882556	10690	Human	Viet Nam
DENV-1/VN/BID-V2812/2007(1)	Dengue virus 1	GQ199822	10690	Human	Viet Nam
DENV-1/VN/BID-V2813/2007(1)	Dengue virus 1	FJ882557	10688	Human	Viet Nam
DENV-1/VN/BID-V2814/2007(1)	Dengue virus 1	FJ882558	10690	Human	Viet Nam
DENV-1/VN/BID-V2815/2007(1)	Dengue virus 1	GQ199823	10675	Human	Viet Nam
DENV-1/VN/BID-V2816/2007(1)	Dengue virus 1	GQ199824	10690	Human	Viet Nam
DENV-1/VN/BID-V2817/2007(1)	Dengue virus 1	GQ199825	10690	Human	Viet Nam
DENV-1/VN/BID-V2818/2007(1)	Dengue virus 1	FJ882559	10690	Human	Viet Nam
DENV-1/VN/BID-V2819/2007(1)	Dengue virus 1	FJ882560	10690	Human	Viet Nam
DENV-1/VN/BID-V2820/2007(1)	Dengue virus 1	GQ199826	10690	Human	Viet Nam
DENV-1/VN/BID-V2821/2007(1)	Dengue virus 1	GQ199827	10676	Human	Viet Nam
DENV-1/VN/BID-V2822/2007(1)	Dengue virus 1	GQ199828	10690	Human	Viet Nam
DENV-1/VN/BID-V2823/2007(1)	Dengue virus 1	FJ882561	10676	Human	Viet Nam
DENV-1/VN/BID-V2824/2007(1)	Dengue virus 1	GQ199829	10690	Human	Viet Nam
DENV-1/VN/BID-V2825/2003(1)	Dengue virus 1	FJ882562	10676	Human	Viet Nam
DENV-1/VN/BID-V2826/2003(1)	Dengue virus 1	FJ882563	10674	Human	Viet Nam
DENV-1/VN/BID-V2827/2003(1)	Dengue virus 1	FJ882564	10681	Human	Viet Nam
DENV-1/VN/BID-V2829/2003(1)	Dengue virus 1	FJ882565	10674	Human	Viet Nam
DENV-1/VN/BID-V2830/2003(1)	Dengue virus 1	GQ199830	10690	Human	Viet Nam
DENV-1/VN/BID-V2831/2003(1)	Dengue virus 1	FJ882566	10688	Human	Viet Nam
DENV-1/VN/BID-V2832/2003(1)	Dengue virus 1	FJ882567	10690	Human	Viet Nam
DENV-1/VN/BID-V2833/2003(1)	Dengue virus 1	GQ199831	10690	Human	Viet Nam
DENV-1/VN/BID-V2834/2003(1)	Dengue virus 1	GQ199832	10690	Human	Viet Nam
DENV-1/VN/BID-V2835/2003(1)	Dengue virus 1	FJ882568	10676	Human	Viet Nam
DENV-1/VN/BID-V2836/2004(1)	Dengue virus 1	FJ882569	10690	Human	Viet Nam
DENV-1/VN/BID-V2837/2004(1)	Dengue virus 1	GQ199833	10690	Human	Viet Nam
DENV-1/VN/BID-V2838/2005(1)	Dengue virus 1	FJ882570	10690	Human	Viet Nam
DENV-1/VN/BID-V2839/2005(1)	Dengue virus 1	GQ199834	10677	Human	Viet Nam

DENV-1/VN/BID-V2840/2005(1)	Dengue virus 1	GQ199835	10690	Human	Viet Nam
DENV-1/VN/BID-V2842/2005(1)	Dengue virus 1	FJ898386	10690	Human	Viet Nam
DENV-1/VN/BID-V2845/2005(1)	Dengue virus 1	GQ199836	10690	Human	Viet Nam
DENV-1/VN/BID-V2846/2005(1)	Dengue virus 1	GQ199837	10684	Human	Viet Nam
DENV-1/VN/BID-V2847/2005(1)	Dengue virus 1	FJ898387	10654	Human	Viet Nam
DENV-1/VN/BID-V2848/2005(1)	Dengue virus 1	FJ898388	10690	Human	Viet Nam
DENV-1/VN/BID-V2850/2005(1)	Dengue virus 1	FJ898389	10690	Human	Viet Nam
DENV-1/VN/BID-V2851/2005(1)	Dengue virus 1	FJ898390	10676	Human	Viet Nam
DENV-1/VN/BID-V2852/2005(1)	Dengue virus 1	FJ898391	10690	Human	Viet Nam
DENV-1/VN/BID-V2853/2005(1)	Dengue virus 1	FJ898392	10690	Human	Viet Nam
DENV-1/VN/BID-V2854/2005(1)	Dengue virus 1	FJ898393	10687	Human	Viet Nam
DENV-1/VN/BID-V2856/2005(1)	Dengue virus 1	FJ898395	10681	Human	Viet Nam
DENV-1/VN/BID-V2857/2005(1)	Dengue virus 1	GQ199838	10690	Human	Viet Nam
DENV-1/VN/BID-V2858/2006(1)	Dengue virus 1	FJ898396	10719	Human	Viet Nam
DENV-1/VN/BID-V2859/2006(1)	Dengue virus 1	GQ199839	10690	Human	Viet Nam
DENV-1/VN/BID-V2860/2006(1)	Dengue virus 1	FJ898397	10680	Human	Viet Nam
DENV-1/VN/BID-V2861/2006(1)	Dengue virus 1	FJ898398	10688	Human	Viet Nam
DENV-1/VN/BID-V2862/2006(1)	Dengue virus 1	FJ898399	10690	Human	Viet Nam
DENV-1/VN/BID-V2863/2006(1)	Dengue virus 1	FJ898400	10688	Human	Viet Nam
DENV-1/VN/BID-V2864/2006(1)	Dengue virus 1	FJ898401	10690	Human	Viet Nam
DENV-1/VN/BID-V2865/2006(1)	Dengue virus 1	FJ898402	10690	Human	Viet Nam
DENV-1/VN/BID-V2867/2006(1)	Dengue virus 1	FJ898403	10690	Human	Viet Nam
DENV-1/VN/BID-V2868/2006(1)	Dengue virus 1	FJ898404	10689	Human	Viet Nam
DENV-1/VN/BID-V2870/2006(1)	Dengue virus 1	GQ199840	10690	Human	Viet Nam
DENV-1/VN/BID-V2871/2006(1)	Dengue virus 1	FJ898405	10679	Human	Viet Nam
DENV-1/VN/BID-V2873/2006(1)	Dengue virus 1	FJ898406	10690	Human	Viet Nam
DENV-1/VN/BID-V2874/2006(1)	Dengue virus 1	GQ199841	10690	Human	Viet Nam
DENV-1/VN/BID-V2875/2006(1)	Dengue virus 1	GQ199842	10690	Human	Viet Nam
DENV-1/VN/BID-V2876/2006(1)	Dengue virus 1	FJ898407	10672	Human	Viet Nam
DENV-1/VN/BID-V2877/2006(1)	Dengue virus 1	FJ898408	10690	Human	Viet Nam
DENV-1/VN/BID-V2878/2006(1)	Dengue virus 1	GQ199843	10690	Human	Viet Nam
DENV-1/VN/BID-V2879/2006(1)	Dengue virus 1	FJ898409	10684	Human	Viet Nam
DENV-1/VN/BID-V2880/2006(1)	Dengue virus 1	GQ199844	10690	Human	Viet Nam
DENV-1/VN/BID-V2881/2006(1)	Dengue virus 1	GQ199845	10690	Human	Viet Nam
DENV-1/VN/BID-V2882/2006-1	Dengue virus 1	JF937598	10705	Human	Viet Nam
DENV-1/VN/BID-V2883/2006(1)	Dengue virus 1	GQ199846	10690	Human	Viet Nam
DENV-1/VN/BID-V2884/2006(1)	Dengue virus 1	GQ199847	10690	Human	Viet Nam
DENV-1/VN/BID-V2885/2006(1)	Dengue virus 1	GQ199848	10690	Human	Viet Nam
DENV-1/VN/BID-V2886/2006(1)	Dengue virus 1	GQ199849	10673	Human	Viet Nam
DENV-1/VN/BID-V2887/2006(1)	Dengue virus 1	GQ199850	10689	Human	Viet Nam
DENV-1/VN/BID-V2888/2006(1)	Dengue virus 1	FJ898410	10690	Human	Viet Nam
DENV-1/VN/BID-V2890/2006(1)	Dengue virus 1	GQ199851	10690	Human	Viet Nam
DENV-1/VN/BID-V2891/2006(1)	Dengue virus 1	FJ898411	10690	Human	Viet Nam
DENV-1/VN/BID-V2892/2006(1)	Dengue virus 1	FJ898412	10672	Human	Viet Nam
DENV-1/VN/BID-V2893/2006(1)	Dengue virus 1	GQ199852	10689	Human	Viet Nam
DENV-1/VN/BID-V2894/2006(1)	Dengue virus 1	FJ898413	10684	Human	Viet Nam
DENV-1/VN/BID-V2895/2006(1)	Dengue virus 1	FJ898414	10679	Human	Viet Nam
DENV-1/VN/BID-V2896/2006(1)	Dengue virus 1	FJ898415	10672	Human	Viet Nam
DENV-1/VN/BID-V2897/2006(1)	Dengue virus 1	GQ199853	10690	Human	Viet Nam
DENV-1/VN/BID-V2898/2006(1)	Dengue virus 1	GQ199854	10690	Human	Viet Nam
DENV-1/VN/BID-V2899/2006(1)	Dengue virus 1	FJ898416	10689	Human	Viet Nam
DENV-1/VN/BID-V2900/2006(1)	Dengue virus 1	FJ898417	10690	Human	Viet Nam
DENV-1/VN/BID-V2901/2006(1)	Dengue virus 1	GQ868612	10690	Human	Viet Nam
DENV-1/VN/BID-V2902/2006(1)	Dengue virus 1	GQ199855	10688	Human	Viet Nam
DENV-1/VN/BID-V2903/2006(1)	Dengue virus 1	FJ898418	10683	Human	Viet Nam
DENV-1/VN/BID-V2904/2006(1)	Dengue virus 1	FJ898419	10676	Human	Viet Nam
DENV-1/VN/BID-V2905/2006(1)	Dengue virus 1	FJ898420	10676	Human	Viet Nam
DENV-1/VN/BID-V2906/2006(1)	Dengue virus 1	FJ898421	10690	Human	Viet Nam
DENV-1/VN/BID-V2907/2006(1)	Dengue virus 1	FJ898422	10690	Human	Viet Nam
DENV-1/VN/BID-V2908/2006(1)	Dengue virus 1	FJ898423	10679	Human	Viet Nam
DENV-1/VN/BID-V2909/2006(1)	Dengue virus 1	GQ868613	10679	Human	Viet Nam
DENV-1/VN/BID-V2910/2006(1)	Dengue virus 1	FJ898424	10690	Human	Viet Nam
DENV-1/VN/BID-V2911/2006(1)	Dengue virus 1	FJ898425	10691	Human	Viet Nam
DENV-1/VN/BID-V2912/2006(1)	Dengue virus 1	FJ898426	10688	Human	Viet Nam
DENV-1/VN/BID-V2913/2006(1)	Dengue virus 1	GQ199856	10690	Human	Viet Nam
DENV-1/VN/BID-V2914/2006(1)	Dengue virus 1	FJ898427	10689	Human	Viet Nam
DENV-1/VN/BID-V2915/2006(1)	Dengue virus 1	FJ898428	10691	Human	Viet Nam
DENV-1/VN/BID-V2916/2006(1)	Dengue virus 1	FJ898429	10688	Human	Viet Nam
DENV-1/VN/BID-V2917/2006(1)	Dengue virus 1	FJ898430	10673	Human	Viet Nam
DENV-1/VN/BID-V2918/2006-1	Dengue virus 1	JF937599	10713	Human	Viet Nam
DENV-1/VN/BID-V2919/2006(1)	Dengue virus 1	FJ898431	10690	Human	Viet Nam
DENV-1/VN/BID-V3839/2008(1)	Dengue virus 1	GU131678	10454	Human	Viet Nam
DENV-1/VN/BID-V3840/2008(1)	Dengue virus 1	GU131679	10468	Human	Viet Nam
DENV-1/VN/BID-V3841/2008-1	Dengue virus 1	JF937600	10444	Human	Viet Nam
DENV-1/VN/BID-V3842/2008(1)	Dengue virus 1	GU131680	10462	Human	Viet Nam
DENV-1/VN/BID-V3843/2008(1)	Dengue virus 1	GU131681	10462	Human	Viet Nam
DENV-1/VN/BID-V3844/2008(1)	Dengue virus 1	GU131682	10486	Human	Viet Nam
DENV-1/VN/BID-V3845/2008(1)	Dengue virus 1	GU131683	10552	Human	Viet Nam
DENV-1/VN/BID-V3846/2008(1)	Dengue virus 1	GU131684	10457	Human	Viet Nam
DENV-1/VN/BID-V3847/2008(1)	Dengue virus 1	GU131685	10469	Human	Viet Nam
DENV-1/VN/BID-V3849/2008(1)	Dengue virus 1	GQ868614	10684	Human	Viet Nam
DENV-1/VN/BID-V3850/2008(1)	Dengue virus 1	GU131686	10486	Human	Viet Nam
DENV-1/VN/BID-V3851/2008(1)	Dengue virus 1	GU131687	10467	Human	Viet Nam
DENV-1/VN/BID-V3852/2008(1)	Dengue virus 1	GU131688	10487	Human	Viet Nam
DENV-1/VN/BID-V3853/2008(1)	Dengue virus 1	GU131689	10403	Human	Viet Nam
DENV-1/VN/BID-V3854/2008(1)	Dengue virus 1	GU131690	10482	Human	Viet Nam
DENV-1/VN/BID-V3855/2008(1)	Dengue virus 1	GU131691	10464	Human	Viet Nam
DENV-1/VN/BID-V3856/2008(1)	Dengue virus 1	GU131692	10482	Human	Viet Nam
DENV-1/VN/BID-V3857/2008(1)	Dengue virus 1	GU131693	10472	Human	Viet Nam
DENV-1/VN/BID-V3859/2008(1)	Dengue virus 1	GU131694	10468	Human	Viet Nam
DENV-1/VN/BID-V3860/2008(1)	Dengue virus 1	GU131695	10477	Human	Viet Nam
DENV-1/VN/BID-V3861/2008(1)	Dengue virus 1	GU131696	10469	Human	Viet Nam
DENV-1/VN/BID-V3862/2008(1)	Dengue virus 1	GU131697	10453	Human	Viet Nam
DENV-1/VN/BID-V3863/2008(1)	Dengue virus 1	GU131698	10418	Human	Viet Nam
DENV-1/VN/BID-V3864/2008(1)	Dengue virus 1	GU131699	10486	Human	Viet Nam
DENV-1/VN/BID-V3865/2008(1)	Dengue virus 1	GU131700	10418	Human	Viet Nam
DENV-1/VN/BID-V3866/2008(1)	Dengue virus 1	GU131701	10404	Human	Viet Nam
DENV-1/VN/BID-V3867/2008(1)	Dengue virus 1	GU131702	10482	Human	Viet Nam
DENV-1/VN/BID-V3868/2008(1)	Dengue virus 1	GU131703	10467	Human	Viet Nam
DENV-1/VN/BID-V3869/2008(1)	Dengue virus 1	GU131704	10473	Human	Viet Nam
DENV-1/VN/BID-V3870/2008(1)	Dengue virus 1	GU131705	10408	Human	Viet Nam
DENV-1/VN/BID-V3871/2008(1)	Dengue virus 1	GU131706	10467	Human	Viet Nam
DENV-1/VN/BID-V3872/2008(1)	Dengue virus 1	GU131707	10461	Human	Viet Nam
DENV-1/VN/BID-V3873/2008(1)	Dengue virus 1	GU131708	10472	Human	Viet Nam
DENV-1/VN/BID-V3874/2008(1)	Dengue virus 1	GU131709	10462	Human	Viet Nam
DENV-1/VN/BID-V3876/2008(1)	Dengue virus 1	GU131710	10418	Human	Viet Nam
DENV-1/VN/BID-V3878/2008(1)	Dengue virus 1	GU131711	10454	Human	Viet Nam
DENV-1/VN/BID-V3879/2008(1)	Dengue virus 1	GU131712	10465	Human	Viet Nam
DENV-1/VN/BID-V3881/2008(1)	Dengue virus 1	GU131713	10462	Human	Viet Nam
DENV-1/VN/BID-V3883/2008(1)	Dengue virus 1	GU131714	10457	Human	Viet Nam
DENV-1/VN/BID-V3884/2008-1	Dengue virus 1	JF937601	10390	Human	Viet Nam
DENV-1/VN/BID-V3885/2008(1)	Dengue virus 1	GU131715	10473	Human	Viet Nam
DENV-1/VN/BID-V3886/2008(1)	Dengue virus 1	GU131716	10472	Human	Viet Nam

DENV-1/VN/BID-V3887/2008(1)	Dengue virus 1	GU131717	10470	Human	Viet Nam
DENV-1/VN/BID-V3888/2008(1)	Dengue virus 1	GU131718	10476	Human	Viet Nam
DENV-1/VN/BID-V3889/2008(1)	Dengue virus 1	GU131719	10462	Human	Viet Nam
DENV-1/VN/BID-V3890/2008(1)	Dengue virus 1	GU131720	10472	Human	Viet Nam
DENV-1/VN/BID-V3891/2008	Dengue virus 1	HM181960	10467	Human	Viet Nam
DENV-1/VN/BID-V3892/2008	Dengue virus 1	HM181961	10402	Human	Viet Nam
DENV-1/VN/BID-V3893/2008	Dengue virus 1	HM181962	10467	Human	Viet Nam
DENV-1/VN/BID-V3894/2008	Dengue virus 1	HM181963	10449	Human	Viet Nam
DENV-1/VN/BID-V3895/2008	Dengue virus 1	HM181964	10451	Human	Viet Nam
DENV-1/VN/BID-V3896/2008	Dengue virus 1	HM181965	10418	Human	Viet Nam
DENV-1/VN/BID-V3897/2008	Dengue virus 1	HM181966	10486	Human	Viet Nam
DENV-1/VN/BID-V3898/2008(1)	Dengue virus 1	GU131721	10676	Human	Viet Nam
DENV-1/VN/BID-V3899/2008(1)	Dengue virus 1	GU131722	10486	Human	Viet Nam
DENV-1/VN/BID-V3900/2008(1)	Dengue virus 1	GU131723	10472	Human	Viet Nam
DENV-1/VN/BID-V3902/2008(1)	Dengue virus 1	GU131724	10475	Human	Viet Nam
DENV-1/VN/BID-V3903/2008(1)	Dengue virus 1	GU131725	10511	Human	Viet Nam
DENV-1/VN/BID-V3904/2008(1)	Dengue virus 1	GU131726	10418	Human	Viet Nam
DENV-1/VN/BID-V3905/2008(1)	Dengue virus 1	GU131727	10486	Human	Viet Nam
DENV-1/VN/BID-V3906/2008	Dengue virus 1	HM631850	10480	Human	Viet Nam
DENV-1/VN/BID-V3907/2008(1)	Dengue virus 1	GU131728	10472	Human	Viet Nam
DENV-1/VN/BID-V3909/2008	Dengue virus 1	KF955446	10470	Human	Viet Nam
DENV-1/VN/BID-V3910/2008	Dengue virus 1	KF921942	10374	Human	Viet Nam
DENV-1/VN/BID-V3932/2008-1	Dengue virus 1	JF937602	10444	Human	Viet Nam
DENV-1/VN/BID-V3933/2008(1)	Dengue virus 1	GU131729	10466	Human	Viet Nam
DENV-1/VN/BID-V3934/2008(1)	Dengue virus 1	GU131730	10446	Human	Viet Nam
DENV-1/VN/BID-V3935/2008(1)	Dengue virus 1	GU131731	10660	Human	Viet Nam
DENV-1/VN/BID-V3937/2008(1)	Dengue virus 1	GU131732	10680	Human	Viet Nam
DENV-1/VN/BID-V3938/2008(1)	Dengue virus 1	GU131733	10486	Human	Viet Nam
DENV-1/VN/BID-V3940/2008(1)	Dengue virus 1	GU131734	10400	Human	Viet Nam
DENV-1/VN/BID-V3941/2008(1)	Dengue virus 1	GU131735	10476	Human	Viet Nam
DENV-1/VN/BID-V3943/2008(1)	Dengue virus 1	GU131736	10472	Human	Viet Nam
DENV-1/VN/BID-V3945/2008(1)	Dengue virus 1	GU131737	10472	Human	Viet Nam
DENV-1/VN/BID-V3946/2008-1	Dengue virus 1	JF937603	10383	Human	Viet Nam
DENV-1/VN/BID-V3947/2008(1)	Dengue virus 1	GU131738	10486	Human	Viet Nam
DENV-1/VN/BID-V3950/2008-1	Dengue virus 1	JN000935	10460	Human	Viet Nam
DENV-1/VN/BID-V3952/2008-1	Dengue virus 1	JF937604	10443	Human	Viet Nam
DENV-1/VN/BID-V3953/2008-1	Dengue virus 1	JF937605	10443	Human	Viet Nam
DENV-1/VN/BID-V3955/2008-1	Dengue virus 1	JF937606	10444	Human	Viet Nam
DENV-1/VN/BID-V3956/2008-1	Dengue virus 1	JF937607	10444	Human	Viet Nam
DENV-1/VN/BID-V3958/2008-1	Dengue virus 1	JF937608	10443	Human	Viet Nam
DENV-1/VN/BID-V3959/2008(1)	Dengue virus 1	GU131739	10684	Human	Viet Nam
DENV-1/VN/BID-V3960/2008(1)	Dengue virus 1	GU131740	10418	Human	Viet Nam
DENV-1/VN/BID-V3961/2008(1)	Dengue virus 1	GU131741	10396	Human	Viet Nam
DENV-1/VN/BID-V3962/2008(1)	Dengue virus 1	GU131742	10460	Human	Viet Nam
DENV-1/VN/BID-V3963/2008(1)	Dengue virus 1	GU131743	10468	Human	Viet Nam
DENV-1/VN/BID-V3964/2008(1)	Dengue virus 1	GU131744	10480	Human	Viet Nam
DENV-1/VN/BID-V3965/2008(1)	Dengue virus 1	GU131745	10482	Human	Viet Nam
DENV-1/VN/BID-V3966/2008	Dengue virus 1	JN093516	10393	Human	Viet Nam
DENV-1/VN/BID-V3968/2008(1)	Dengue virus 1	GU131746	10472	Human	Viet Nam
DENV-1/VN/BID-V3971/2008-1	Dengue virus 1	JF937609	10444	Human	Viet Nam
DENV-1/VN/BID-V3972/2008	Dengue virus 1	KF921949	10449	Human	Viet Nam
DENV-1/VN/BID-V3973/2008(1)	Dengue virus 1	GU131747	10486	Human	Viet Nam
DENV-1/VN/BID-V3974/2008(1)	Dengue virus 1	GU131748	10418	Human	Viet Nam
DENV-1/VN/BID-V3975/2008(1)	Dengue virus 1	GQ868615	10684	Human	Viet Nam
DENV-1/VN/BID-V3976/2008(1)	Dengue virus 1	GU131749	10486	Human	Viet Nam
DENV-1/VN/BID-V3977/2008(1)	Dengue virus 1	GU131750	10675	Human	Viet Nam
DENV-1/VN/BID-V3978/2008(1)	Dengue virus 1	GU131751	10385	Human	Viet Nam
DENV-1/VN/BID-V3979/2008(1)	Dengue virus 1	GU131752	10470	Human	Viet Nam
DENV-1/VN/BID-V3980/2008(1)	Dengue virus 1	GU131753	10461	Human	Viet Nam
DENV-1/VN/BID-V3981/2008(1)	Dengue virus 1	GU131754	10482	Human	Viet Nam
DENV-1/VN/BID-V3982/2008(1)	Dengue virus 1	GU131755	10476	Human	Viet Nam
DENV-1/VN/BID-V3983/2008(1)	Dengue virus 1	GU131756	10474	Human	Viet Nam
DENV-1/VN/BID-V3984/2008(1)	Dengue virus 1	GU131757	10471	Human	Viet Nam
DENV-1/VN/BID-V3985/2008(1)	Dengue virus 1	GU131758	10486	Human	Viet Nam
DENV-1/VN/BID-V3986/2008(1)	Dengue virus 1	GU131759	10676	Human	Viet Nam
DENV-1/VN/BID-V3987/2008(1)	Dengue virus 1	GU131760	10474	Human	Viet Nam
DENV-1/VN/BID-V3989/2008(1)	Dengue virus 1	GU131761	10486	Human	Viet Nam
DENV-1/VN/BID-V3990/2008(1)	Dengue virus 1	GU131762	10409	Human	Viet Nam
DENV-1/VN/BID-V3991/2008(1)	Dengue virus 1	GU131763	10474	Human	Viet Nam
DENV-1/VN/BID-V3992/2008(1)	Dengue virus 1	GU131764	10471	Human	Viet Nam
DENV-1/VN/BID-V3994/2008(1)	Dengue virus 1	GU131765	10485	Human	Viet Nam
DENV-1/VN/BID-V3995/2008(1)	Dengue virus 1	GU131766	10486	Human	Viet Nam
DENV-1/VN/BID-V3996/2008-1	Dengue virus 1	JF937610	10444	Human	Viet Nam
DENV-1/VN/BID-V3997/2008(1)	Dengue virus 1	GU131767	10472	Human	Viet Nam
DENV-1/VN/BID-V3998/2008	Dengue virus 1	HM181967	10472	Human	Viet Nam
DENV-1/VN/BID-V4002/2008-1	Dengue virus 1	JF937611	10444	Human	Viet Nam
DENV-1/VN/BID-V4004/2008(1)	Dengue virus 1	GU131768	10479	Human	Viet Nam
DENV-1/VN/BID-V4005/2008(1)	Dengue virus 1	GU131769	10462	Human	Viet Nam
DENV-1/VN/BID-V4006/2008(1)	Dengue virus 1	GU131770	10481	Human	Viet Nam
DENV-1/VN/BID-V4007/2008(1)	Dengue virus 1	GU131771	10462	Human	Viet Nam
DENV-1/VN/BID-V4008/2008(1)	Dengue virus 1	GU131772	10464	Human	Viet Nam
DENV-1/VN/BID-V4009/2008(1)	Dengue virus 1	GU131773	10418	Human	Viet Nam
DENV-1/VN/BID-V4010/2008(1)	Dengue virus 1	GU131774	10468	Human	Viet Nam
DENV-1/VN/BID-V4013/2008(1)	Dengue virus 1	GU131775	10486	Human	Viet Nam
DENV-1/VN/BID-V4014/2008(1)	Dengue virus 1	GU131776	10464	Human	Viet Nam
DENV-1/VN/BID-V4015/2008(1)	Dengue virus 1	GU131777	10478	Human	Viet Nam
DENV-1/VN/BID-V4016/2008(1)	Dengue virus 1	GU131778	10599	Human	Viet Nam
DENV-1/VN/BID-V4017/2008(1)	Dengue virus 1	GU131779	10482	Human	Viet Nam
DENV-1/VN/BID-V4018/2008(1)	Dengue virus 1	GU131780	10475	Human	Viet Nam
DENV-1/VN/BID-V4019/2008(1)	Dengue virus 1	GU131781	10468	Human	Viet Nam
DENV-1/VN/BID-V4020/2008(1)	Dengue virus 1	GU131782	10670	Human	Viet Nam
DENV-1/VN/BID-V4021/2008(1)	Dengue virus 1	GU131783	10482	Human	Viet Nam
DENV-1/VN/BID-V4022/2008(1)	Dengue virus 1	GU131784	10476	Human	Viet Nam
DENV-1/VN/BID-V4023/2008(1)	Dengue virus 1	GU131785	10470	Human	Viet Nam
DENV-1/VN/BID-V4024/2008-1	Dengue virus 1	JF937612	10587	Human	Viet Nam
DENV-1/VN/BID-V4026/2008(1)	Dengue virus 1	GU131786	10472	Human	Viet Nam
DENV-1/VN/BID-V4027/2008(1)	Dengue virus 1	GU131787	10467	Human	Viet Nam
DENV-1/VN/BID-V4028/2008(1)	Dengue virus 1	GU131788	10513	Human	Viet Nam
DENV-1/VN/BID-V4029/2008	Dengue virus 1	HM181968	10476	Human	Viet Nam
DENV-1/VN/BID-V4030/2008(1)	Dengue virus 1	GU131789	10482	Human	Viet Nam
DENV-1/VN/BID-V4031/2008(1)	Dengue virus 1	GU131790	10515	Human	Viet Nam
DENV-1/VN/BID-V4032/2008	Dengue virus 1	HM181969	10454	Human	Viet Nam
DENV-1/VN/BID-V4033/2008(1)	Dengue virus 1	GU131791	10486	Human	Viet Nam
DENV-1/VN/BID-V4034/2008(1)	Dengue virus 1	GU131792	10457	Human	Viet Nam
DENV-1/VN/BID-V4035/2008	Dengue virus 1	HM488256	10459	Human	Viet Nam
DENV-1/VN/BID-V4036/2008(1)	Dengue virus 1	GU131793	10472	Human	Viet Nam
DENV-1/VN/BID-V4037/2008(1)	Dengue virus 1	GU131794	10472	Human	Viet Nam
DENV-1/VN/BID-V4038/2008(1)	Dengue virus 1	GU131795	10404	Human	Viet Nam
DENV-1/VN/BID-V4039/2008(1)	Dengue virus 1	GU131796	10467	Human	Viet Nam
DENV-1/VN/BID-V4040/2008(1)	Dengue virus 1	GU131797	10465	Human	Viet Nam
DENV-1/VN/BID-V4041/2008(1)	Dengue virus 1	GU131798	10471	Human	Viet Nam
DENV-1/VN/BID-V4042/2008(1)	Dengue virus 1	GU131799	10404	Human	Viet Nam
DENV-1/VN/BID-V4043/2008(1)	Dengue virus 1	GU131800	10477	Human	Viet Nam

DENV-1/VN/BID-V4044/2008(1)	Dengue virus 1	GU131801	10467	Human	Viet Nam
DENV-1/VN/BID-V4045/2008-1	Dengue virus 1	JF937613	10386	Human	Viet Nam
DENV-1/VN/BID-V4047/2008-1	Dengue virus 1	JF937614	10444	Human	Viet Nam
DENV-1/VN/BID-V4048/2008(1)	Dengue virus 1	GU131802	10486	Human	Viet Nam
DENV-1/VN/BID-V4049/2008(1)	Dengue virus 1	GU131803	10463	Human	Viet Nam
DENV-1/VN/BID-V4051/2008(1)	Dengue virus 1	GU131804	10464	Human	Viet Nam
DENV-1/VN/BID-V4052/2008(1)	Dengue virus 1	GU131805	10451	Human	Viet Nam
DENV-1/VN/BID-V4053/2008	Dengue virus 1	HM631851	10385	Human	Viet Nam
DENV-1/VN/BID-V4054/2008(1)	Dengue virus 1	GU131806	10404	Human	Viet Nam
DENV-1/VN/BID-V4055/2008(1)	Dengue virus 1	GU131807	10444	Human	Viet Nam
DENV-1/VN/BID-V4056/2008(1)	Dengue virus 1	GU131808	10476	Human	Viet Nam
DENV-1/VN/BID-V4057/2008(1)	Dengue virus 1	GU131809	10474	Human	Viet Nam
DENV-1/VN/BID-V4058/2008(1)	Dengue virus 1	GU131810	10474	Human	Viet Nam
DENV-1/VN/BID-V4059/2008(1)	Dengue virus 1	GU131811	10465	Human	Viet Nam
DENV-1/VN/BID-V4062/2008-1	Dengue virus 1	JF937615	10448	Human	Viet Nam
DENV-1/VN/BID-V4063/2008(1)	Dengue virus 1	GU131812	10519	Human	Viet Nam
DENV-1/VN/BID-V4064/2008(1)	Dengue virus 1	GU131813	10474	Human	Viet Nam
DENV-1/VN/BID-V4065/2008(1)	Dengue virus 1	GU131814	10468	Human	Viet Nam
DENV-1/VN/BID-V4066/2008(1)	Dengue virus 1	GU131815	10385	Human	Viet Nam
DENV-1/VN/BID-V4067/2008(1)	Dengue virus 1	GU131816	10513	Human	Viet Nam
DENV-1/VN/BID-V4068/2008(1)	Dengue virus 1	GU131817	10418	Human	Viet Nam
DENV-1/VN/BID-V4069/2008(1)	Dengue virus 1	GU131818	10467	Human	Viet Nam
DENV-1/VN/BID-V4070/2008(1)	Dengue virus 1	GU131819	10462	Human	Viet Nam
DENV-1/VN/BID-V4071/2008(1)	Dengue virus 1	GU131820	10405	Human	Viet Nam
DENV-1/VN/BID-V4072/2008	Dengue virus 1	HM181970	10472	Human	Viet Nam
DENV-1/VN/BID-V4073/2008(1)	Dengue virus 1	GU131821	10482	Human	Viet Nam
DENV-1/VN/BID-V4074/2008(1)	Dengue virus 1	GU131822	10479	Human	Viet Nam
DENV-1/VN/BID-V4075/2008(1)	Dengue virus 1	GU131823	10486	Human	Viet Nam
DENV-1/VN/BID-V4076/2008(1)	Dengue virus 1	GU131824	10475	Human	Viet Nam
DENV-1/VN/BID-V4077/2008(1)	Dengue virus 1	GU131825	10462	Human	Viet Nam
DENV-1/VN/BID-V4078/2008(1)	Dengue virus 1	GU131826	10471	Human	Viet Nam
DENV-1/VN/BID-V4079/2008-1	Dengue virus 1	JF937616	10431	Human	Viet Nam
DENV-1/VN/BID-V4080/2008-1	Dengue virus 1	JF937617	10454	Human	Viet Nam
DENV-1/VN/BID-V4081/2008-1	Dengue virus 1	JF937618	10444	Human	Viet Nam
DENV-1/VN/BID-V4082/2008-1	Dengue virus 1	JF937619	10444	Human	Viet Nam
DENV-1/VN/BID-V4083/2008(1)	Dengue virus 1	GU131827	10418	Human	Viet Nam
DENV-1/VN/BID-V4084/2008(1)	Dengue virus 1	GU131828	10676	Human	Viet Nam
DENV-1/VN/BID-V4085/2008(1)	Dengue virus 1	GU131829	10476	Human	Viet Nam
DENV-1/VN/BID-V4087/2008(1)	Dengue virus 1	GU131830	10453	Human	Viet Nam
DENV-1/VN/BID-V4088/2008(1)	Dengue virus 1	GU131831	10473	Human	Viet Nam
DENV-1/VN/BID-V767/2003(1)	Dengue virus 1	EU482789	10690	Human	Viet Nam
DENV-1/VN/BID-V768/2003(1)	Dengue virus 1	EU482790	10690	Human	Viet Nam
DENV-1/VN/BID-V769/2003(1)	Dengue virus 1	EU482791	10690	Human	Viet Nam
DENV-1/VN/BID-V770/2003(1)	Dengue virus 1	EU482792	10690	Human	Viet Nam
DENV-1/VN/BID-V780/2006(1)	Dengue virus 1	EU482706	10690	Human	Viet Nam
DENV-1/VN/BID-V781/2006(1)	Dengue virus 1	EU482707	10690	Human	Viet Nam
DENV-1/VN/BID-V782/2007(1)	Dengue virus 1	EU482708	10690	Human	Viet Nam
DENV-1/VN/BID-V783/2007(1)	Dengue virus 1	EU482709	10690	Human	Viet Nam
DENV-1/VN/BID-V784/2007(1)	Dengue virus 1	EU482710	10690	Human	Viet Nam
DENV-1/VN/BID-V785/2007(1)	Dengue virus 1	EU482711	10690	Human	Viet Nam
DENV-1/VN/BID-V786/2007(1)	Dengue virus 1	EU482712	10690	Human	Viet Nam
DENV-1/VN/BID-V787/2007(1)	Dengue virus 1	EU482713	10690	Human	Viet Nam
DENV-1/VN/BID-V788/2007(1)	Dengue virus 1	EU482714	10690	Human	Viet Nam
DENV-1/VN/BID-V789/2007(1)	Dengue virus 1	EU482715	10690	Human	Viet Nam
DENV-1/VN/BID-V790/2007(1)	Dengue virus 1	EU482716	10690	Human	Viet Nam
DENV-1/VN/BID-V791/2007(1)	Dengue virus 1	EU482717	10690	Human	Viet Nam
DENV-1/VN/BID-V792/2007(1)	Dengue virus 1	EU482718	10690	Human	Viet Nam
DENV-1/VN/BID-V793/2006(1)	Dengue virus 1	EU249490	10690	Human	Viet Nam
DENV-1/VN/BID-V794/2006(1)	Dengue virus 1	EU249491	10690	Human	Viet Nam
DENV-1/VN/BID-V795/2006(1)	Dengue virus 1	EU249492	10690	Human	Viet Nam
DENV-1/VN/BID-V796/2006(1)	Dengue virus 1	EU249493	10690	Human	Viet Nam
DENV-1/VN/BID-V797/2006(1)	Dengue virus 1	EU249494	10690	Human	Viet Nam
DENV-1/VN/BID-V798/2006(1)	Dengue virus 1	EU249495	10690	Human	Viet Nam
DENV-1/VN/BID-V799/2006(1)	Dengue virus 1	EU482793	10690	Human	Viet Nam
DENV-1/VN/BID-V800/2006(1)	Dengue virus 1	EU482794	10689	Human	Viet Nam
DENV-1/VN/BID-V801/2006(1)	Dengue virus 1	EU482795	10690	Human	Viet Nam
DENV-1/VN/BID-V802/2006(1)	Dengue virus 1	EU482796	10690	Human	Viet Nam
DENV-1/VN/BID-V803/2006(1)	Dengue virus 1	EU482797	10690	Human	Viet Nam
DENV-1/VN/BID-V804/2006(1)	Dengue virus 1	EU482798	10690	Human	Viet Nam
DENV-1/VN/BID-V805/2006(1)	Dengue virus 1	EU482799	10690	Human	Viet Nam
DENV-1/VN/BID-V806/2006(1)	Dengue virus 1	EU482800	10690	Human	Viet Nam
DENV-1/VN/BID-V807/2006(1)	Dengue virus 1	EU482801	10690	Human	Viet Nam
DENV-1/VN/BID-V808/2006(1)	Dengue virus 1	EU482802	10690	Human	Viet Nam
DENV-1/VN/BID-V809/2006(1)	Dengue virus 1	EU482803	10690	Human	Viet Nam
DENV-1/VN/BID-V810/2006(1)	Dengue virus 1	EU482804	10690	Human	Viet Nam
DENV-1/VN/BID-V811/2006(1)	Dengue virus 1	EU482805	10690	Human	Viet Nam
DENV-1/VN/BID-V812/2006(1)	Dengue virus 1	EU482806	10690	Human	Viet Nam
DENV-1/VN/BID-V813/2006(1)	Dengue virus 1	EU482807	10690	Human	Viet Nam
DENV-1/VN/BID-V814/2006(1)	Dengue virus 1	EU482808	10690	Human	Viet Nam
DENV-1/VN/BID-V815/2006(1)	Dengue virus 1	EU482809	10690	Human	Viet Nam
DENV-1/VN/BID-V816/2006(1)	Dengue virus 1	EU482810	10690	Human	Viet Nam
DENV-1/VN/BID-V817/2006(1)	Dengue virus 1	EU482811	10690	Human	Viet Nam
DENV-1/VN/BID-V818/2006(1)	Dengue virus 1	EU482812	10690	Human	Viet Nam
DENV-1/VN/BID-V819/2006(1)	Dengue virus 1	EU482813	10690	Human	Viet Nam
DENV-1/VN/BID-V820/2006(1)	Dengue virus 1	EU482814	10690	Human	Viet Nam
DENV-1/VN/BID-V821/2006(1)	Dengue virus 1	EU482815	10690	Human	Viet Nam
DENV-1/VN/BID-V822/2006(1)	Dengue virus 1	EU482816	10690	Human	Viet Nam
DENV-1/VN/BID-V823/2006(1)	Dengue virus 1	EU482817	10690	Human	Viet Nam
DENV-1/VN/BID-V824/2006(1)	Dengue virus 1	EU482818	10690	Human	Viet Nam
DENV-1/VN/BID-V825/2006(1)	Dengue virus 1	EU482819	10688	Human	Viet Nam
DENV-1/VN/BID-V826/2006(1)	Dengue virus 1	EU482820	10690	Human	Viet Nam
DENV-1/VN/BID-V827/2006(1)	Dengue virus 1	EU482821	10690	Human	Viet Nam
DENV-1/VN/BID-V828/2006(1)	Dengue virus 1	EU482822	10690	Human	Viet Nam
DENV-1/VN/BID-V829/2006(1)	Dengue virus 1	EU482823	10690	Human	Viet Nam
DENV-1/VN/BID-V830/2006(1)	Dengue virus 1	EU482824	10690	Human	Viet Nam
DENV-1/VN/BID-V831/2006(1)	Dengue virus 1	EU482825	10690	Human	Viet Nam
DENV-1/VN/BID-V832/2006(1)	Dengue virus 1	EU482826	10690	Human	Viet Nam
DENV-1/VN/BID-V833/2006(1)	Dengue virus 1	EU482827	10690	Human	Viet Nam
DENV-1/VN/BID-V834/2006(1)	Dengue virus 1	EU482828	10690	Human	Viet Nam
DENV-1/VN/BID-V931/2003(1)	Dengue virus 1	EU482476	10690	Human	Viet Nam
DENV-1/VN/BID-V932/2007(1)	Dengue virus 1	EU482477	10690	Human	Viet Nam
DENV-1/VN/BID-V933/2007(1)	Dengue virus 1	EU482478	10690	Human	Viet Nam
DENV-1/VN/BID-V934/2007(1)	Dengue virus 1	EU482479	10690	Human	Viet Nam
DENV-1/VN/BID-V935/2006(1)	Dengue virus 1	EU482480	10690	Human	Viet Nam
DENV-1/VN/BID-V936/2007(1)	Dengue virus 1	EU482481	10690	Human	Viet Nam
DENV-1/VN/BID-V937/2007(1)	Dengue virus 1	EU482482	10690	Human	Viet Nam
DENV-1/VN/BID-V938/2007(1)	Dengue virus 1	EU482483	10690	Human	Viet Nam
DENV-1/VN/BID-V939/2007(1)	Dengue virus 1	EU482484	10690	Human	Viet Nam
DENV-1/VN/BID-V940/2007(1)	Dengue virus 1	EU482485	10690	Human	Viet Nam
DENV-1/VN/BID-V941/2007(1)	Dengue virus 1	EU482486	10690	Human	Viet Nam
DENV-1/VN/BID-V942/2007(1)	Dengue virus 1	EU482487	10690	Human	Viet Nam
DENV-1/VN/BID-V943/2007(1)	Dengue virus 1	EU482488	10690	Human	Viet Nam
DENV-1/VN/BID-V944/2007(1)	Dengue virus 1	EU482489	10690	Human	Viet Nam

DENV-1/VN/BID-V945/2007(1)	Dengue virus 1	EU482490	10675	Human	Viet Nam
DENV-1/VN/BID-V946/2007(1)	Dengue virus 1	EU482491	10684	Human	Viet Nam
DENV-1/VN/BID-V947/2007(1)	Dengue virus 1	EU482492	10688	Human	Viet Nam
DENV-1/VN/BID-V948/2007(1)	Dengue virus 1	EU482493	10690	Human	Viet Nam
DENV-1/VN/BID-V949/2007(1)	Dengue virus 1	EU482494	10684	Human	Viet Nam
DENV-1/VN/BID-V950/2007(1)	Dengue virus 1	EU482495	10690	Human	Viet Nam
DENV-1/VN/BID-V951/2007(1)	Dengue virus 1	EU482496	10690	Human	Viet Nam
DENV-1/VN/BID-V952/2007(1)	Dengue virus 1	EU482497	10690	Human	Viet Nam
DENV-1/VN/BID-V953/2007(1)	Dengue virus 1	EU482498	10690	Human	Viet Nam
DENV-1/VN/BID-V954/2007(1)	Dengue virus 1	EU482499	10690	Human	Viet Nam
DENV-1/VN/BID-V955/2007(1)	Dengue virus 1	EU482500	10685	Human	Viet Nam
DENV-1/VN/BID-V956/2007(1)	Dengue virus 1	EU482501	10690	Human	Viet Nam
DENV-1/VN/BID-V957/2007(1)	Dengue virus 1	EU482502	10686	Human	Viet Nam
DENV-1/VN/BID-V958/2007(1)	Dengue virus 1	EU482503	10690	Human	Viet Nam
DENV-1/VN/BID-V959/2007(1)	Dengue virus 1	EU482504	10690	Human	Viet Nam
DENV-1/VN/BID-V960/2007(1)	Dengue virus 1	EU482505	10690	Human	Viet Nam
DENV-1/VN/BID-V961/2007(1)	Dengue virus 1	EU482506	10690	Human	Viet Nam
DENV-1/VN/BID-V962/2007(1)	Dengue virus 1	EU482507	10690	Human	Viet Nam
DENV-1/VN/BID-V963/2007(1)	Dengue virus 1	EU482508	10690	Human	Viet Nam
DENV-1/VN/BID-V964/2007(1)	Dengue virus 1	EU482509	10690	Human	Viet Nam
DENV-1/VN/BID-V965/2007(1)	Dengue virus 1	EU482510	10690	Human	Viet Nam
DENV-1/VN/BID-V966/2007(1)	Dengue virus 1	EU482511	10690	Human	Viet Nam
DENV-1/VN/BID-V967/2007(1)	Dengue virus 1	EU482512	10684	Human	Viet Nam
DENV-1/VN/BID-V968/2007(1)	Dengue virus 1	EU482513	10690	Human	Viet Nam
DENV-1/VN/BID-V969/2007(1)	Dengue virus 1	EU482514	10690	Human	Viet Nam
DENV-1/VN/BID-V970/2007(1)	Dengue virus 1	EU482515	10690	Human	Viet Nam
DENV-1/VN/BID-V971/2007(1)	Dengue virus 1	FJ461303	10691	Human	Viet Nam
DENV-1/VN/BID-V972/2007(1)	Dengue virus 1	EU482516	10690	Human	Viet Nam
DENV-1/VN/BID-V973/2007(1)	Dengue virus 1	EU482517	10690	Human	Viet Nam
DENV-1/VN/BID-V974/2006(1)	Dengue virus 1	EU482518	10690	Human	Viet Nam
DENV-1/VN/BID-V975/2006(1)	Dengue virus 1	EU482519	10690	Human	Viet Nam
DENV-1/VN/BID-V976/2006(1)	Dengue virus 1	EU482520	10690	Human	Viet Nam
DENV-1/VN/BID-V977/2006(1)	Dengue virus 1	EU482521	10688	Human	Viet Nam
DENV-1/VN/BID-V978/2006(1)	Dengue virus 1	EU482522	10690	Human	Viet Nam
DENV-1/VN/BID-V979/2006(1)	Dengue virus 1	EU482523	10690	Human	Viet Nam
DENV-1/VN/BID-V980/2006(1)	Dengue virus 1	EU482524	10690	Human	Viet Nam
DENV-1/VN/BID-V981/2006(1)	Dengue virus 1	EU482525	10690	Human	Viet Nam
DENV-1/VN/BID-V982/2006(1)	Dengue virus 1	EU482526	10690	Human	Viet Nam
DENV-1/VN/BID-V983/2006(1)	Dengue virus 1	EU482527	10688	Human	Viet Nam
DENV-1/VN/BID-V984/2006(1)	Dengue virus 1	EU482528	10689	Human	Viet Nam
DENV-1/VN/BID-V985/2006(1)	Dengue virus 1	EU482529	10690	Human	Viet Nam
DENV-1/VN/BID-V986/2006(1)	Dengue virus 1	EU482530	10690	Human	Viet Nam
DENV-1/VN/BID-V987/2006(1)	Dengue virus 1	EU482531	10690	Human	Viet Nam
DENV-1/VN/BID-V988/2006(1)	Dengue virus 1	EU482532	10690	Human	Viet Nam
DENV-1/VN/BID-V989/2006(1)	Dengue virus 1	EU482533	10690	Human	Viet Nam
DENV-1/VN/BID-V990/2006(1)	Dengue virus 1	EU482534	10690	Human	Viet Nam
DENV-1/VN/BID-V991/2006(1)	Dengue virus 1	EU482535	10690	Human	Viet Nam
DENV-1/VN/BID-V992/2006(1)	Dengue virus 1	EU482536	10690	Human	Viet Nam
DENV-1/VN/BID-V993/2006(1)	Dengue virus 1	EU482537	10690	Human	Viet Nam
DENV-1/VN/BID-V994/2006(1)	Dengue virus 1	EU482538	10690	Human	Viet Nam
DENV-1/VN/BID-V995/2006(1)	Dengue virus 1	EU482539	10690	Human	Viet Nam
DENV-1/VN/BID-V996/2006(1)	Dengue virus 1	EU482540	10690	Human	Viet Nam
DENV1/CN/GZ27/2014-1	Dengue virus 1	KP723473	10630	Human	China
DENV1/CN/GZ35/2014-1	Dengue virus 1	KP723476	10628	Human	China
DENV1/CN/GZDF696/2014-1	Dengue virus 1	KT187563	10714	Human	China
DENV1/CN/GZDF716/2014-1	Dengue virus 1	KT187564	10735	Human	China
DENV1/CN/GZDF780/2014-1	Dengue virus 1	KT187559	10714	Human	China
DENV1/CN/GZDF874/2014-1	Dengue virus 1	KT187560	10714	Human	China
DENV1/CN/GZDF876/2014-1	Dengue virus 1	KT187561	10714	Human	China
DENV1/CN/GZDF982/2014-1	Dengue virus 1	KT187562	10714	Human	China
DENV1/Vietnam/10dx-111-801-Placebo-48hrs-1	Dengue virus 1	JQ045626	10674	Human	Viet Nam
DENV1/Vietnam/10dx-111-802-Placebo-48hrs-1	Dengue virus 1	JQ045627	10651	Human	Viet Nam
DENV1/Vietnam/10dx-114-801-Placebo-0hrs-1	Dengue virus 1	JQ045628	10689	Human	Viet Nam
DENV1/Vietnam/10dx-116-801-1500mg-0hrs-1	Dengue virus 1	JQ045629	10699	Human	Viet Nam
DENV1/Vietnam/10dx-118-801-Placebo-72hrs-1	Dengue virus 1	JQ045630	10670	Human	Viet Nam
DENV1/Vietnam/10dx-118-802-Placebo-72hrs-1	Dengue virus 1	JQ045631	10697	Human	Viet Nam
DENV1/Vietnam/10dx-120-801-1500mg-0hrs-1	Dengue virus 1	JQ045632	10671	Human	Viet Nam
DENV1/Vietnam/10dx-201-801-Placebo-12hrs-1	Dengue virus 1	JQ045633	10671	Human	Viet Nam
DENV1/Vietnam/10dx-201-802-Placebo-12hrs-1	Dengue virus 1	JQ045634	10699	Human	Viet Nam
DENV1/Vietnam/10dx-203-801-3000mg-12hrs-1	Dengue virus 1	JQ045636	10671	Human	Viet Nam
DENV1/Vietnam/10dx-203-802-3000mg-12hrs-1	Dengue virus 1	JQ045635	10692	Human	Viet Nam
DENV1/Vietnam/10dx-204-801-Placebo-36hrs-1	Dengue virus 1	JQ045637	10674	Human	Viet Nam
DENV1/Vietnam/10dx-204-802-Placebo-36hrs-1	Dengue virus 1	JQ045638	10671	Human	Viet Nam
DENV1/Vietnam/10dx-205-801-3000mg-24hrs-1	Dengue virus 1	JQ045639	10671	Human	Viet Nam
DENV1/Vietnam/10dx-205-802-3000mg-24hrs-1	Dengue virus 1	JQ045640	10672	Human	Viet Nam
DENV1/Vietnam/10dx-209-801-3000mg-12hrs-1	Dengue virus 1	JQ045641	10670	Human	Viet Nam
DENV1/Vietnam/10dx-209-802-3000mg-12hrs-1	Dengue virus 1	JQ045645	10652	Human	Viet Nam
DENV1/Vietnam/10dx-210-801-Placebo-24hrs-1	Dengue virus 1	JQ045642	10672	Human	Viet Nam
DENV1/Vietnam/10dx-210-802-Placebo-24hrs-1	Dengue virus 1	JQ045643	10669	Human	Viet Nam
DENV1/Vietnam/10dx-218-801-Placebo-0hrs-1	Dengue virus 1	JQ045644	10672	Human	Viet Nam
DENV1/Vietnam/10dx-219-801-3000mg-0hrs-1	Dengue virus 1	JQ045646	10672	Human	Viet Nam
DENV1/Vietnam/10dx-221-801-Placebo-72hrs-1	Dengue virus 1	JQ045647	10672	Human	Viet Nam
DENV1/Vietnam/10dx-221-802-Placebo-72hrs-1	Dengue virus 1	JQ045648	10672	Human	Viet Nam
DENV1/Vietnam/10dx-222-801-3000mg-60hrs-1	Dengue virus 1	JQ045649	10670	Human	Viet Nam
DENV1/Vietnam/10dx-222-802-3000mg-60hrs-1	Dengue virus 1	JQ045650	10669	Human	Viet Nam
DENV1/Vietnam/10dx-225-801-3000mg-36hrs-1	Dengue virus 1	JQ045651	10672	Human	Viet Nam
DENV1/Vietnam/10dx-225-802-3000mg-36hrs-1	Dengue virus 1	JQ045652	10669	Human	Viet Nam
DENV1/Vietnam/10dx-226-801-Placebo-48hrs-1	Dengue virus 1	JQ045653	10672	Human	Viet Nam
DENV1/Vietnam/10dx-226-802-Placebo-48hrs-1	Dengue virus 1	JQ045654	10669	Human	Viet Nam
DENV1/Vietnam/10dx-227-801-Placebo-24hrs-1	Dengue virus 1	JQ045655	10673	Human	Viet Nam
DENV1/Vietnam/10dx-227-802-Placebo-24hrs-1	Dengue virus 1	JQ045656	10669	Human	Viet Nam
DENV1/Vietnam/10dx-228-801-3000mg-72hrs-1	Dengue virus 1	JQ045657	10672	Human	Viet Nam
DENV1/Vietnam/10dx-228-802-3000mg-72hrs-1	Dengue virus 1	JQ045658	10669	Human	Viet Nam
DENV1/Vietnam/10dx-229-801-Placebo-36hrs-1	Dengue virus 1	JQ045659	10672	Human	Viet Nam
DENV1/Vietnam/10dx-229-802-Placebo-36hrs-1	Dengue virus 1	JQ045660	10672	Human	Viet Nam
DENV1/Vietnam/10dx-231-801-3000mg-48hrs-1	Dengue virus 1	JQ045661	10672	Human	Viet Nam
DENV1/Vietnam/10dx-231-802-3000mg-48hrs-1	Dengue virus 1	JQ045662	10671	Human	Viet Nam
DENV1/Vietnam/10dx-238-801-Placebo-60hrs-1	Dengue virus 1	JQ045663	10672	Human	Viet Nam
DENV1/Vietnam/10dx-238-802-Placebo-60hrs-1	Dengue virus 1	JQ045664	10670	Human	Viet Nam
DENV1/Vietnam/10dx-241-801-Placebo-24hrs-1	Dengue virus 1	JQ045665	10672	Human	Viet Nam
DENV1/Vietnam/10dx-241-802-Placebo-24hrs-1	Dengue virus 1	JQ045666	10669	Human	Viet Nam
DENV1/Vietnam/10dx-244-801-3000mg-48hrs-1	Dengue virus 1	JQ045667	10671	Human	Viet Nam
DENV1/Vietnam/10dx-244-802-3000mg-48hrs-1	Dengue virus 1	JQ045668	10687	Human	Viet Nam
DF-1	Dengue virus 1	HQ624983	10562	Human	Cambodia
DF01-HUB01021093(1)	Dengue virus 1	FJ384655	10735	Human	Brazil
DF1203-type 1	Dengue virus 1	KC759167	10735	Human	China
DG14-1	Dengue virus 1	JQ048541	10718	Human	China
DGVgz01(1)	Dengue virus 1	EU280167	10735	Human	China
DH/S1/05/04	Dengue virus 1	JN697056	10735	Human	Malaysia
DH/S1/05/152	Dengue virus 1	JN697058	10735	Human	Malaysia
DH/S1/05/154	Dengue virus 1	JN697057	10735	Human	Malaysia
DHF-1	Dengue virus 1	HQ624984	10539	Human	Cambodia
DK87	Dengue virus 1	KP398852	10735	Human	Sri Lanka

DS06-210505(1)	Dengue virus 1	EU179860	10628	Unknown	Brunei
DS212-110306(1)	Dengue virus 1	EU179861	10672	Unknown	Brunei
DV1_SL_2009a-1	Dengue virus 1	HQ891313	10734	Human	Sri Lanka
DV1_SL_2009b-1	Dengue virus 1	HQ891314	10735	Human	Sri Lanka
DV1_SL_2009c-1	Dengue virus 1	HQ891315	10735	Human	Sri Lanka
DV1_SL_2009d-1	Dengue virus 1	HQ891316	10735	Human	Sri Lanka
DV1_SL_2009e-1	Dengue virus 1	JN054256	10735	Human	Sri Lanka
DV1_SL_2010b-1	Dengue virus 1	JN054255	10735	Human	Sri Lanka
FGA/89(1)	Dengue virus 1	AF226687	10735	Unknown	-N/A-
FGA/NA a5c(1)	Dengue virus 1	EF122232	10735	Unknown	French Guiana
FGA/NA d1d(1)	Dengue virus 1	AF226686	10735	Unknown	-N/A-
FGA/NA P6(1)	Dengue virus 1	EF122231	10735	Unknown	French Guiana
Fj231/04(1)	Dengue virus 1	DQ193572	10735	Unknown	China
FP0203(1)	Dengue virus 1	DQ672556	10735	Unknown	French Polynesia
FP0705(1)	Dengue virus 1	DQ672557	10735	Unknown	French Polynesia
FP0908(1)	Dengue virus 1	DQ672558	10735	Unknown	French Polynesia
FP1104(1)	Dengue virus 1	DQ672559	10735	Unknown	French Polynesia
GD01/06	Dengue virus 1	FJ196843	10735	Unknown	China
GD01/97	Dengue virus 1	FJ196847	10735	Unknown	China
GD02/06	Dengue virus 1	FJ196844	10735	Unknown	China
GD03/91	Dengue virus 1	FJ196845	10735	Unknown	China
GD54/03	Dengue virus 1	FJ196841	10735	Unknown	China
GD66/03	Dengue virus 1	FJ196842	10735	Unknown	China
GD95/95	Dengue virus 1	FJ196846	10735	Unknown	China
GD99/99	Dengue virus 1	FJ196848	10735	Unknown	China
GZ/80(1)	Dengue virus 1	AF350498	10735	Unknown	-N/A-
GZ01/95-1	Dengue virus 1	EF032590	10735	Unknown	-N/A-
GZ10-1	Dengue virus 1	KJ438293	10724	Human	China
GZ2002-1	Dengue virus 1	JN205310	10735	Human	China
GZ27-1	Dengue virus 1	KJ438296	10735	Human	China
Haiti/1207/2014-1	Dengue virus 1	KT279761	10720	Human	Haiti
Hawaii-1	Dengue virus 1	KM204119	10736	Human	USA
HawM2516(1)	Dengue virus 1	DQ672560	10735	Unknown	French Polynesia
HawM2540(1)	Dengue virus 1	DQ672562	10735	Unknown	USA
HawM3430(1)	Dengue virus 1	DQ672561	10735	Unknown	USA
HawO3663(1)	Dengue virus 1	DQ672564	10735	Unknown	USA
HawO3758(1)	Dengue virus 1	DQ672563	10735	Unknown	USA
HNRG12188-1	Dengue virus 1	KC692495	10735	Human	Argentina
HNRG12447-1	Dengue virus 1	KC692496	10735	Human	Argentina
HNRG12560-1	Dengue virus 1	KC692497	10735	Human	Argentina
HNRG12589-1	Dengue virus 1	KC692498	10735	Human	Argentina
HNRG13154-1	Dengue virus 1	KC692499	10735	Human	Argentina
HNRG13188-1	Dengue virus 1	KC692500	10735	Human	Argentina
HNRG13301-1	Dengue virus 1	KC692501	10735	Human	Argentina
HNRG13405-1	Dengue virus 1	KC692502	10735	Human	Argentina
HNRG13561-1	Dengue virus 1	KC692503	10735	Human	Argentina
HNRG13707-1	Dengue virus 1	KC692504	10735	Human	Argentina
HNRG13708-1	Dengue virus 1	KC692505	10735	Human	Argentina
HNRG14043-1	Dengue virus 1	KC692506	10735	Human	Argentina
HNRG14076-1	Dengue virus 1	KC692507	10735	Human	Argentina
HNRG14194-1	Dengue virus 1	KC692508	10735	Human	Argentina
HNRG14635-1	Dengue virus 1	KC692509	10735	Human	Argentina
HNRG15417-1	Dengue virus 1	KC692510	10735	Human	Argentina
HNRG24827-1	Dengue virus 1	KC692511	10735	Human	Argentina
HNRG25001-1	Dengue virus 1	KC692512	10735	Human	Argentina
HNRG27213-1	Dengue virus 1	KC692513	10735	Human	Argentina
HNRG27486-1	Dengue virus 1	KC692514	10735	Human	Argentina
HNRG28226-1	Dengue virus 1	KC692515	10735	Human	Argentina
HNRG28425-1	Dengue virus 1	KC692516	10735	Human	Argentina
HNRG37945-1	Dengue virus 1	KC692517	10735	Human	Argentina
KD86-035-1	Dengue virus 1	JN638336	10735	Unknown	Thailand
KD90-157-1	Dengue virus 1	JN638337	10735	Unknown	Thailand
KD92-080-1	Dengue virus 1	JN638338	10735	Unknown	Thailand
KDH0026A	Dengue virus 1	HG316481	10735	Unknown	Thailand
KDH0030A	Dengue virus 1	HG316482	10735	Unknown	Thailand
MKS-0055-1	Dengue virus 1	KC762649	10735	Human	Indonesia
MKS-0056-1	Dengue virus 1	KC762625	10735	Human	Indonesia
MKS-0077-1	Dengue virus 1	KC762654	10735	Human	Indonesia
MKS-0080-1	Dengue virus 1	KC762636	10735	Human	Indonesia
MKS-0088-1	Dengue virus 1	KC762635	10735	Human	Indonesia
MKS-0109-1	Dengue virus 1	KC762650	10735	Human	Indonesia
MKS-0206-1	Dengue virus 1	KC762623	10735	Human	Indonesia
MKS-0248-1	Dengue virus 1	KC762632	10735	Human	Indonesia
MKS-0344-1	Dengue virus 1	KC762646	10735	Human	Indonesia
MKS-0352-1	Dengue virus 1	KC762637	10735	Human	Indonesia
MKS-0390-1	Dengue virus 1	KC762620	10735	Human	Indonesia
MKS-0395-1	Dengue virus 1	KC762631	10735	Human	Indonesia
MKS-0397-1	Dengue virus 1	KC762621	10735	Human	Indonesia
MKS-0398-1	Dengue virus 1	KC762643	10735	Human	Indonesia
MKS-0483-1	Dengue virus 1	KC762651	10735	Human	Indonesia
MKS-0486-1	Dengue virus 1	KC762653	10735	Human	Indonesia
MKS-1001-1	Dengue virus 1	KC762652	10735	Human	Indonesia
MKS-2004-1	Dengue virus 1	KC762638	10735	Human	Indonesia
MKS-2029-1	Dengue virus 1	KC762640	10735	Human	Indonesia
MKS-2040-1	Dengue virus 1	KC762645	10735	Human	Indonesia
MKS-2058-1	Dengue virus 1	KC762628	10735	Human	Indonesia
MKS-2082-1	Dengue virus 1	KC762624	10735	Human	Indonesia
MKS-2094-1	Dengue virus 1	KC762644	10735	Human	Indonesia
MKS-2097-1	Dengue virus 1	KC762630	10735	Human	Indonesia
MKS-2138-1	Dengue virus 1	KC762626	10735	Human	Indonesia
MKS-2147-1	Dengue virus 1	KC762641	10735	Human	Indonesia
MKS-2194-1	Dengue virus 1	KC762648	10735	Human	Indonesia
MKS-2200-1	Dengue virus 1	KC762622	10735	Human	Indonesia
MKS-2201-1	Dengue virus 1	KC762634	10735	Human	Indonesia
MKS-2216-1	Dengue virus 1	KC762633	10735	Human	Indonesia
MKS-IF039-1	Dengue virus 1	KC762629	10735	Human	Indonesia
MKS-IF062-1	Dengue virus 1	KC762627	10735	Human	Indonesia
MKS-WS72-1	Dengue virus 1	KC762647	10735	Human	Indonesia
MKS-WS81-1	Dengue virus 1	KC762639	10735	Human	Indonesia
MKS-WS88-1	Dengue virus 1	KC762642	10735	Human	Indonesia
Mochizuki(type 1)	Dengue virus 1	AB074760	10735	Unknown	-N/A-
Nauru Island Western Pacific 45AZ5(1)	Dengue virus 1	NC_001477 *	10735	Unknown	-N/A-
Nauru Island, Western Pacific-1	Dengue virus 1	U88535	10735	Unknown	-N/A-
Nauru Island, Western Pacific-1	Dengue virus 1	U88536	10735	Unknown	-N/A-
Nauru Island, Western Pacific-1	Dengue virus 1	U88537	10735	Unknown	-N/A-
NC02/030602-585-1	Dengue virus 1	JQ915077	10699	Human	New Caledonia
NC09/300509-13639-1	Dengue virus 1	JQ915079	10699	Human	New Caledonia
NC10/080810-1138-1	Dengue virus 1	JQ915080	10699	Human	New Caledonia
NDF30	Dengue virus 1	AB608786	10749	Human	Taiwan
P23086-1	Dengue virus 1	KF289073	10690	Human	India
P72-1244-1	Dengue virus 1	EF457905	10735	Unknown	Malaysia
PF07/051107-164-1	Dengue virus 1	JQ915072	10699	Human	French Polynesia
PF07/230407-201-1	Dengue virus 1	JQ915071	10699	Human	French Polynesia

	PF08/070308-138-1	Dengue virus 1	JQ915073	10699	Human	French Polynesia
	PF08/180908-01-1	Dengue virus 1	JQ915074	10699	Human	French Polynesia
	PF09/060209-120-1	Dengue virus 1	JQ915075	10699	Human	French Polynesia
	PF09/090609-76-1	Dengue virus 1	JQ915076	10699	Human	French Polynesia
	Reunion 185/04(1)	Dengue virus 1	DQ285558	10689	Human	Reunion
	Reunion 191/04(1)	Dengue virus 1	DQ285559	10717	Human	Reunion
	Reunion 257/04(1)	Dengue virus 1	DQ285560	10696	Human	Reunion
	RGCB294-1	Dengue virus 1	JN903578	10715	Human	India
	RGCB419-1	Dengue virus 1	JN903579	10694	Human	India
	RGCB585-1	Dengue virus 1	JN903580	10694	Human	India
	RGCB592-1	Dengue virus 1	JN903581	10694	Human	India
	RR107-1	Dengue virus 1	KF289072	10669	Human	India
	RR121-1	Dengue virus 1	JQ692085	10669	Human	India
	RR57-1	Dengue virus 1	JQ917404	10669	Human	India
	S275/90(1)	Dengue virus 1	A75711	10718	Unknown	-N/A-
	SB 01057805 (1)	Dengue virus 1	AB519681	10735	Unknown	Brazil
	SDDF1543	Dengue virus 1	AB608787	10749	Human	Taiwan
	Seychelles 1480_04(1)	Dengue virus 1	DQ285561	10662	Human	Seychelles
	SG(EHI)D1209Y03(1)	Dengue virus 1	FJ469907	10642	Unknown	Singapore
	SG(EHI)D1210Y03(1)	Dengue virus 1	FJ469908	10642	Unknown	Singapore
	SG(EHI)D1227Y03(1)	Dengue virus 1	FJ469909	10642	Unknown	Singapore
	SGEHI(D1)0266Y08(1)	Dengue virus 1	GU370048	10542	Human	Singapore
	SGEHI(D1)1494Y08(1)	Dengue virus 1	GU370049	10564	Human	Singapore
	Singapore 8114/93(1)	Dengue virus 1	AY762084	10733	Unknown	-N/A-
	Singapore S275/90(1)	Dengue virus 1	M87512	10717	Unknown	-N/A-
	SL_2012_GS0289-1	Dengue virus 1	KJ726663	10659	Unknown	Sri Lanka
	SL_2012_GS0308-1	Dengue virus 1	KJ726664	10684	Unknown	Sri Lanka
	SL_2012_GS0319-1	Dengue virus 1	KJ726662	10690	Unknown	Sri Lanka
	Su1	Dengue virus 1	KJ933413	10689	Human	China
	SV2951/07-1	Dengue virus 1	HM469968	10735	Human	Thailand
	ThD1_0008_81-1	Dengue virus 1	AY732483	10735	Unknown	-N/A-
	ThD1_0049_01-1	Dengue virus 1	AY732482	10735	Unknown	Thailand
	ThD1_0081_82-1	Dengue virus 1	AY732481	10735	Unknown	Thailand
	ThD1_0097_94-1	Dengue virus 1	AY732480	10735	Unknown	Thailand
	ThD1_0102_01-1	Dengue virus 1	AY732479	10735	Unknown	Thailand
	ThD1_0323_91-1	Dengue virus 1	AY732478	10735	Unknown	Thailand
	ThD1_0336_91-1	Dengue virus 1	AY732477	10735	Unknown	Thailand
	ThD1_0442_80-1	Dengue virus 1	AY732476	10735	Unknown	Thailand
	ThD1_0488_94-1	Dengue virus 1	AY732475	10735	Unknown	Thailand
	ThD1_0673_80-1	Dengue virus 1	AY732474	10735	Unknown	Thailand
	UNKNOWN-DI195805	Dengue virus 1	DI195805	10735	Unknown	-N/A-
	UNKNOWN-FV537254	Dengue virus 1	FV537254	10735	Unknown	-N/A-
	UNKNOWN-FV537255	Dengue virus 1	FV537255	10735	Unknown	-N/A-
	UNKNOWN-FV537256	Dengue virus 1	FV537256	10735	Unknown	-N/A-
	UNKNOWN-FV537257	Dengue virus 1	FV537257	10735	Unknown	-N/A-
	UNKNOWN-FW503158(1)	Dengue virus 1	FW503158	10735	Unknown	-N/A-
	UNKNOWN-HH961660(1)	Dengue virus 1	HH961660	10735	Unknown	-N/A-
	UNKNOWN-HI553375(1)	Dengue virus 1	HI553375	10735	Unknown	-N/A-
	UNKNOWN-HZ038019	Dengue virus 1	HZ038019	10735	Unknown	-N/A-
	UNKNOWN-JC562941	Dengue virus 1	JC562941	10735	Unknown	-N/A-
	UNKNOWN-JE963510	Dengue virus 1	JE963510	10735	Unknown	-N/A-
	VE_61006_2006-1	Dengue virus 1	HQ332182	10735	Human	Venezuela
	VE_61059_2006-1	Dengue virus 1	HQ332177	10735	Human	Venezuela
	VE_61060_2006-1	Dengue virus 1	HQ332178	10735	Human	Venezuela
	VE_61063_2006-1	Dengue virus 1	HQ332181	10735	Human	Venezuela
	VE_61068_2006-1	Dengue virus 1	HQ332180	10735	Human	Venezuela
	VE_61081_2007-1	Dengue virus 1	HQ332183	10735	Human	Venezuela
	VE_61084_2007-1	Dengue virus 1	HQ332179	10735	Human	Venezuela
	VR-1254(1)	Dengue virus 1	EU848545	10734	Unknown	USA
	Western Pacific(1)	Dengue virus 1	AY145121	10735	Unknown	-N/A-
	Western Pacific(1)	Dengue virus 1	AY145122	10733	Unknown	-N/A-
	Western Pacific(1)	Dengue virus 1	AY145123	10705	Unknown	-N/A-
	ww2014	Dengue virus 1	KP686070	10670	Human	China
	ZH1067(1)	Dengue virus 1	EU359008	10735	Human	China
	ZH398-1	Dengue virus 1	KC131140	10668	Human	China
	ZH415-1	Dengue virus 1	KC131141	10671	Human	China
	ZJ01/2004(1)	Dengue virus 1	AY835999	10727	Human	China
**DENV2**	00-St-002	Dengue virus 2	KF744400	10176	Human	Philippines
	00-St-022	Dengue virus 2	KF744401	10176	Human	Philippines
	00-St-68B	Dengue virus 2	KF744402	10176	Human	Philippines
	01-St-206	Dengue virus 2	KF744397	10176	Human	Philippines
	05-RBD-204	Dengue virus 2	KF744408	10176	Human	Philippines
	05-Sa-018	Dengue virus 2	KF744398	10176	Human	Philippines
	1018-DF-12/03/2001(2)	Dengue virus 2	DQ645543	10671	Unknown	Taiwan
	1024-DHF-12/07/2001(2)	Dengue virus 2	DQ645544	10671	Unknown	Taiwan
	1183-DF-06/17/2002(2)	Dengue virus 2	DQ645545	10616	Unknown	Taiwan
	1222-DF-06/24/2002(2)	Dengue virus 2	DQ645546	10671	Unknown	Taiwan
	131-2	Dengue virus 2	AF100469	10674	Unknown	-N/A-
	1328(2)	Dengue virus 2	EU056812	10714	Human	Puerto Rico
	1349-2	Dengue virus 2	EU056810	10723	Human	Burkina Faso
	13858/BR-PE/10	Dengue virus 2	JX669476	10723	Human	Brazil
	1392-2	Dengue virus 2	JX475906	10670	Human	India
	1421-DF-07/16/2002(2)	Dengue virus 2	DQ645547	10671	Unknown	Taiwan
	1464-DHF-07/20/2002(2)	Dengue virus 2	DQ645548	10644	Unknown	Taiwan
	14905/BR-PE/10	Dengue virus 2	JX669477	10723	Human	Brazil
	16681	Dengue virus 2	NC_001474 *	10723	Unknown	Thailand
	16681-2	Dengue virus 2	M84727	10723	Unknown	-N/A-
	16681-PDK53(2)	Dengue virus 2	M84728	10723	Unknown	-N/A-
	19190/BR-PE/10	Dengue virus 2	JX669478	10723	Human	Brazil
	1945-DHF-08/18/2002(2)	Dengue virus 2	DQ645549	10671	Unknown	Taiwan
	1949-DF-08/19/2002(2)	Dengue virus 2	DQ645550	10671	Unknown	Taiwan
	2191-DF-09/12/2002(2)	Dengue virus 2	DQ645551	10671	Unknown	Taiwan
	2208-DHF-09/13/2002(2)	Dengue virus 2	DQ645552	10671	Unknown	Taiwan
	2559-DHF-10/23/2002(2)	Dengue virus 2	DQ645553	10671	Unknown	Taiwan
	2587-DF-10/26/2002(2)	Dengue virus 2	DQ645554	10616	Unknown	Taiwan
	2659-DHF-11/01/2002(2)	Dengue virus 2	DQ645555	10616	Unknown	Taiwan
	2784-DF-11/18/2002(2)	Dengue virus 2	DQ645556	10671	Unknown	Taiwan
	3275/BR-PE/95	Dengue virus 2	JX669480	10723	Human	Brazil
	3311/BR-PE/95	Dengue virus 2	JX669481	10723	Human	Brazil
	3337/BR-PE/95	Dengue virus 2	JX669482	10726	Human	Brazil
	37473/BR-PE/97	Dengue virus 2	JX669483	10725	Human	Brazil
	43(2)	Dengue virus 2	AF204178	10723	Unknown	China
	44(2)	Dengue virus 2	AF204177	10723	Unknown	China
	47913/BR-PE/98	Dengue virus 2	JX669484	10723	Human	Brazil
	51347/BR-PE/98	Dengue virus 2	JX669485	10723	Human	Brazil
	57135/BR-PE/99	Dengue virus 2	JX669486	10724	Human	Brazil
	72144/BR-PE/00	Dengue virus 2	JX669487	10723	Human	Brazil
	87086/BR-PE/02	Dengue virus 2	JX669488	10724	Human	Brazil
	904-DHF-10/31/2001(2)	Dengue virus 2	DQ645540	10671	Unknown	Taiwan
	915-DF-11/03/2001(2)	Dengue virus 2	DQ645541	10616	Unknown	Taiwan
	9479/BR-PE/10	Dengue virus 2	JX669479	10726	Human	Brazil
	95-SLMC-125	Dengue virus 2	KF744403	10176	Human	Philippines
	95-SLMC-148	Dengue virus 2	KF744406	10176	Human	Philippines

950-DF-11/12/2001(2)	Dengue virus 2	DQ645542	10616	Unknown	Taiwan
96-BRL-12	Dengue virus 2	KF744404	10176	Human	Philippines
96-CSMC-007	Dengue virus 2	KF744407	10176	Human	Philippines
96-SLMC-179	Dengue virus 2	KF744405	10176	Human	Philippines
98-CI-15	Dengue virus 2	KF744399	10176	Human	Philippines
98900663 DHF DV-2(2)	Dengue virus 2	AB189122	10723	Human	Indonesia
98900665 DF DV-2(2)	Dengue virus 2	AB189123	10723	Human	Indonesia
98900666 DSS DV-2(2)	Dengue virus 2	AB189124	10723	Human	Indonesia
ACS380-2	Dengue virus 2	JX286526	10630	Human	Brazil
ACS46-2	Dengue virus 2	JX286516	10542	Human	Brazil
ACS46_II-2	Dengue virus 2	JX286517	10539	Human	Brazil
ACS538-2	Dengue virus 2	JX286518	10544	Human	Brazil
ACS542-2	Dengue virus 2	JX286519	10540	Human	Brazil
ACS721-2	Dengue virus 2	JX286521	10597	Human	Brazil
BA05i(2)	Dengue virus 2	AY858035	10723	Human	Indonesia
BR DEN2 01-01-2	Dengue virus 2	JX073928	10723	Unknown	Brazil
BR0690/RJ/2008(2)	Dengue virus 2	HQ026763	10176	Human	Brazil
BR64022(2)	Dengue virus 2	AF489932	10722	Unknown	-N/A-
C0166(2)	Dengue virus 2	AF100463	10685	Unknown	-N/A-
C0167(2)	Dengue virus 2	AF100464	10685	Unknown	-N/A-
C0371(2)	Dengue virus 2	AF100461	10685	Unknown	-N/A-
C0390(2)	Dengue virus 2	AF100462	10684	Unknown	-N/A-
CAM7786(2)	Dengue virus 2	GU369819	10521	Human	Mexico
China 04(2)	Dengue virus 2	AF119661	10723	Unknown	China
CNS36	Dengue virus 2	JX649147	10723	Human	Viet Nam
CNS36	Dengue virus 2	JX649148	10723	Human	Viet Nam
CSF381(2)	Dengue virus 2	FM210236	10659	Unknown	Viet Nam
CSF63(2)	Dengue virus 2	FM210214	10685	Unknown	Viet Nam
Cuba115/97-2	Dengue virus 2	AY702036	10722	Unknown	Cuba
Cuba13/97-2	Dengue virus 2	AY702034	10722	Unknown	Cuba
Cuba165/97-2	Dengue virus 2	AY702038	10722	Unknown	Cuba
Cuba205/97-2	Dengue virus 2	AY702039	10722	Unknown	Cuba
Cuba58/97(2)	Dengue virus 2	AY702035	10722	Unknown	Cuba
Cuba89/97-2	Dengue virus 2	AY702037	10722	Unknown	Cuba
Cuba_A115_1981	Dengue virus 2	KF704356	10176	Human	Cuba
Cuba_A132_1981	Dengue virus 2	KF704355	10176	Human	Cuba
Cuba_A15_1981	Dengue virus 2	KF704354	10176	Human	Cuba
Cuba_A169_1981	Dengue virus 2	KF704357	10176	Human	Cuba
Cuba_A35_1981	Dengue virus 2	KF704358	10176	Human	Cuba
D2/AS/UH73/1972(2)	Dengue virus 2	HM582107	10713	Unknown	American Samoa
D2/AS/UH77/1972(2)	Dengue virus 2	HM582104	10713	Unknown	American Samoa
D2/AS/UH79/1972(2)	Dengue virus 2	HM582105	10713	Unknown	American Samoa
D2/AS/UH85/1972(2)	Dengue virus 2	HM582106	10714	Unknown	American Samoa
D2/FJ/UH21/1971(2)	Dengue virus 2	HM582099	10714	Unknown	Fiji
D2/FJ/UH22/1971(2)	Dengue virus 2	HM582101	10694	Unknown	Fiji
D2/FJ/UH40/1971(2)	Dengue virus 2	HM582100	10713	Unknown	Fiji
D2/NC/UH37/1971(2)	Dengue virus 2	HM582102	10713	Unknown	New Caledonia
D2/NC/UH97/1972(2)	Dengue virus 2	HM582103	10713	Unknown	New Caledonia
D2/Pakistan/2011-23/2011-2	Dengue virus 2	KF041232	10723	Human	Pakistan
D2/Pakistan/2011-3/2011-2	Dengue virus 2	KF041233	10723	Human	Pakistan
D2/Pakistan/2011-4/2011-2	Dengue virus 2	KF041234	10723	Human	Pakistan
D2/Pakistan/209/2009-2	Dengue virus 2	KF041235	10723	Human	Pakistan
D2/Pakistan/51/2008-2	Dengue virus 2	KF041236	10723	Human	Pakistan
D2/Pakistan/78/2009-2	Dengue virus 2	KF041237	10723	Human	Pakistan
D2/PF/UH00/1973(2)	Dengue virus 2	HM582110	10713	Unknown	French Polynesia
D2/PF/UH50/1972(2)	Dengue virus 2	HM582108	10713	Unknown	French Polynesia
D2/PF/UH57/1971(2)	Dengue virus 2	HM582109	10681	Unknown	French Polynesia
D2/Pk/A1/2011-2	Dengue virus 2	KM217157	10656	Human	Pakistan
D2/Pk/A2/2011-2	Dengue virus 2	KM217156	10656	Human	Pakistan
D2/Pk/Swat-01-2	Dengue virus 2	KM217158	10656	Human	Pakistan
D2/SG/05K3295DK1/2005(2)	Dengue virus 2	EU081177	10723	Unknown	Singapore
D2/SG/05K3330DK1/2005(2)	Dengue virus 2	EU081178	10723	Unknown	Singapore
D2/SG/05K4137DK1/2005(2)	Dengue virus 2	EU081179	10723	Unknown	Singapore
D2/SG/05K4155DK1/2005(2)	Dengue virus 2	EU081180	10723	Unknown	Singapore
D2/TO/UH04/1974(2)	Dengue virus 2	HM582117	10714	Unknown	Tonga
D2/TO/UH16/1974(2)	Dengue virus 2	HM582111	10713	Unknown	Tonga
D2/TO/UH19/1974(2)	Dengue virus 2	HM582112	10714	Unknown	Tonga
D2/TO/UH20/1974(2)	Dengue virus 2	HM582113	10713	Unknown	Tonga
D2/TO/UH39/1974(2)	Dengue virus 2	HM582114	10652	Unknown	Tonga
D2/TO/UH44/1974(2)	Dengue virus 2	HM582115	10713	Unknown	Tonga
D2/TO/UH94/1974(2)	Dengue virus 2	HM582116	10713	Unknown	Tonga
Dak Ar 141069(2)	Dengue virus 2	EF105389	10723	Unknown	Senegal
Dak Ar 141070(2)	Dengue virus 2	EF105390	10717	Unknown	Senegal
Dak Ar 2039(2)	Dengue virus 2	EF105382	10722	Unknown	Burkina Faso
Dak Ar 510(2)	Dengue virus 2	EF105381	10722	Unknown	Cote dIvoire
Dak Ar 578(2)	Dengue virus 2	EF105380	10722	Unknown	Cote dIvoire
Dak Ar A1247(2)	Dengue virus 2	EF105383	10700	Unknown	Cote dIvoire
Dak Ar A2022(2)	Dengue virus 2	EF105386	10721	Unknown	Burkina Faso
Dak Ar D20761(2)	Dengue virus 2	EF105385	10724	Unknown	Senegal
Dak Ar D75505(2)	Dengue virus 2	EF457904	10724	Unknown	Senegal
Dak HD 10674(2)	Dengue virus 2	EF105384	10724	Unknown	Senegal
DC331Y11-2	Dengue virus 2	KM279515	10723	Human	Singapore
DC353Y11-2	Dengue virus 2	KM279517	10723	Human	Singapore
DC357Y11-2	Dengue virus 2	KM279518	10723	Human	Singapore
DC367Y11-2	Dengue virus 2	KM279519	10723	Human	Singapore
DC378Y11-2	Dengue virus 2	KM279520	10723	Human	Singapore
DC380Y11-2	Dengue virus 2	KM279581	10723	Human	Singapore
DC389Y11-2	Dengue virus 2	KM279582	10723	Human	Singapore
DC391Y11-2	Dengue virus 2	KM279521	10723	Human	Singapore
DC395Y11-2	Dengue virus 2	KM279522	10723	Human	Singapore
DC403Y11-2	Dengue virus 2	KM279523	10723	Human	Singapore
DC415Y11-2	Dengue virus 2	KM279524	10723	Human	Singapore
DC427Y11-2	Dengue virus 2	KM279586	10723	Human	Singapore
DC429Y11-2	Dengue virus 2	KM279525	10723	Human	Singapore
DC430Y11-2	Dengue virus 2	KM279526	10723	Human	Singapore
DC589Y12-2	Dengue virus 2	KM279528	10723	Human	Singapore
DC596Y12-2	Dengue virus 2	KM279587	10723	Human	Singapore
DC597Y12-2	Dengue virus 2	KM279530	10723	Human	Singapore
DC618Y12-2	Dengue virus 2	KM279532	10723	Human	Singapore
DC619Y12-2	Dengue virus 2	KM279533	10723	Human	Singapore
DC620Y12-2	Dengue virus 2	KM279534	10723	Human	Singapore
DC621Y12-2	Dengue virus 2	KM279535	10723	Human	Singapore
DC629Y12-2	Dengue virus 2	KM279536	10723	Human	Singapore
DC635Y12-2	Dengue virus 2	KM279537	10723	Human	Singapore
DC636Y12-2	Dengue virus 2	KM279588	10723	Human	Singapore
DC639Y12-2	Dengue virus 2	KM279538	10723	Human	Singapore
DC641Y12-2	Dengue virus 2	KM279539	10723	Human	Singapore
DC642Y12-2	Dengue virus 2	KM279540	10723	Human	Singapore
DC643Y12-2	Dengue virus 2	KM279541	10723	Human	Singapore
DC644Y12-2	Dengue virus 2	KM279542	10723	Human	Singapore
DC645Y12-2	Dengue virus 2	KM279543	10723	Human	Singapore
DC648Y12-2	Dengue virus 2	KM279571	10723	Human	Singapore
DC649Y12-2	Dengue virus 2	KM279544	10723	Human	Singapore

DC652Y12-2	Dengue virus 2	KM279545	10723	Human	Singapore
DC654Y12-2	Dengue virus 2	KM279546	10723	Human	Singapore
DC657Y12-2	Dengue virus 2	KM279548	10723	Human	Singapore
DC661Y12-2	Dengue virus 2	KM279549	10723	Human	Singapore
DC663Y12-2	Dengue virus 2	KM279550	10723	Human	Singapore
DC669Y12-2	Dengue virus 2	KM279551	10723	Human	Singapore
DC673Y12-2	Dengue virus 2	KM279552	10723	Human	Singapore
DC677Y12-2	Dengue virus 2	KM279553	10723	Human	Singapore
DC687Y12-2	Dengue virus 2	KM279554	10723	Human	Singapore
DC688Y12-2	Dengue virus 2	KM279590	10723	Human	Singapore
DC694Y12-2	Dengue virus 2	KM279555	10723	Human	Singapore
DC704Y12-2	Dengue virus 2	KM279556	10723	Human	Singapore
DC710Y12-2	Dengue virus 2	KM279557	10724	Human	Singapore
DC716Y12-2	Dengue virus 2	KM279591	10723	Human	Singapore
DC719Y12-2	Dengue virus 2	KM279597	10723	Human	Singapore
DC720Y12-2	Dengue virus 2	KM279592	10723	Human	Singapore
DC730Y12-2	Dengue virus 2	KM279558	10723	Human	Singapore
DC735Y12-2	Dengue virus 2	KM279559	10723	Human	Singapore
DC740Y12-2	Dengue virus 2	KM279560	10723	Human	Singapore
DC756Y12-2	Dengue virus 2	KM279561	10723	Human	Singapore
DC759Y12-2	Dengue virus 2	KM279593	10723	Human	Singapore
DC763Y12-2	Dengue virus 2	KM279562	10723	Human	Singapore
DC766Y12-2	Dengue virus 2	KM279563	10723	Human	Singapore
DC771Y12-2	Dengue virus 2	KM279594	10723	Human	Singapore
DC786Y12-2	Dengue virus 2	KM279564	10723	Human	Singapore
DC790Y12-2	Dengue virus 2	KM279565	10723	Human	Singapore
DC792Y12-2	Dengue virus 2	KM279595	10723	Human	Singapore
DC793Y12-2	Dengue virus 2	KM279566	10723	Human	Singapore
DC795Y12-2	Dengue virus 2	KM279572	10723	Human	Singapore
DC796Y12-2	Dengue virus 2	KM279573	10723	Human	Singapore
DC811Y12-2	Dengue virus 2	KM279567	10723	Human	Singapore
DC812Y12-2	Dengue virus 2	KM279568	10723	Human	Singapore
DC814Y12-2	Dengue virus 2	KM279569	10723	Human	Singapore
DC827Y12-2	Dengue virus 2	KM279575	10723	Human	Singapore
DC848Y12-2	Dengue virus 2	KM279570	10723	Human	Singapore
DEN2/H/IMTSSA-MART/98-703(2)	Dengue virus 2	AF208496	10722	Human	Martinique
DENV-2/BF/BID-V3502/1986(2)	Dengue virus 2	GU131843	10657	Mosquito	Burkina Faso
DENV-2/BR/BID-V2376/2000(2)	Dengue virus 2	FJ850072	10677	Human	Brazil
DENV-2/BR/BID-V2377/2000	Dengue virus 2	JN819419	10601	Human	Brazil
DENV-2/BR/BID-V2379/2001(2)	Dengue virus 2	FJ850074	10678	Human	Brazil
DENV-2/BR/BID-V2382/2002(2)	Dengue virus 2	FJ850076	10663	Human	Brazil
DENV-2/BR/BID-V2385/2003(2)	Dengue virus 2	GQ868640	10667	Human	Brazil
DENV-2/BR/BID-V2386/2003(2)	Dengue virus 2	FJ850078	10667	Human	Brazil
DENV-2/BR/BID-V2390/2004(2)	Dengue virus 2	FJ850082	10679	Human	Brazil
DENV-2/BR/BID-V2393/2005(2)	Dengue virus 2	FJ850085	10677	Human	Brazil
DENV-2/BR/BID-V2396/2006(2)	Dengue virus 2	FJ850088	10667	Human	Brazil
DENV-2/BR/BID-V2399/2007(2)	Dengue virus 2	FJ850091	10678	Human	Brazil
DENV-2/BR/BID-V2402/2008(2)	Dengue virus 2	GQ199890	10679	Human	Brazil
DENV-2/BR/BID-V3481/2008(2)	Dengue virus 2	GQ868549	10678	Human	Brazil
DENV-2/BR/BID-V3483/2008(2)	Dengue virus 2	GQ868550	10669	Human	Brazil
DENV-2/BR/BID-V3486/2008(2)	Dengue virus 2	GQ868551	10639	Human	Brazil
DENV-2/BR/BID-V3495/2008(2)	Dengue virus 2	GU131864	10531	Human	Brazil
DENV-2/BR/BID-V3637/2008	Dengue virus 2	HM181971	10531	Human	Brazil
DENV-2/BR/BID-V3638/2008(2)	Dengue virus 2	GU131879	10639	Human	Brazil
DENV-2/BR/BID-V3640/2008(2)	Dengue virus 2	GU131880	10531	Human	Brazil
DENV-2/BR/BID-V3644/2008(2)	Dengue virus 2	GU131881	10531	Human	Brazil
DENV-2/BR/BID-V3645/2008(2)	Dengue virus 2	GU131882	10644	Human	Brazil
DENV-2/BR/BID-V3648/2008(2)	Dengue virus 2	GU131883	10678	Human	Brazil
DENV-2/BR/BID-V3650/2008(2)	Dengue virus 2	GU131884	10665	Human	Brazil
DENV-2/BR/BID-V3653/2008(2)	Dengue virus 2	GU131885	10677	Human	Brazil
DENV-2/BZ/BID-V2952/2002(2)	Dengue virus 2	FJ898461	10679	Human	Belize
DENV-2/CO/BID-V1594/2005(2)	Dengue virus 2	FJ024473	10647	Human	Colombia
DENV-2/CO/BID-V1595/2005(2)	Dengue virus 2	FJ024474	10640	Human	Colombia
DENV-2/CO/BID-V1596/2005(2)	Dengue virus 2	FJ024475	10640	Human	Colombia
DENV-2/CO/BID-V1597/2005(2)	Dengue virus 2	FJ182012	10637	Human	Colombia
DENV-2/CO/BID-V1599/1944(2)	Dengue virus 2	EU854293	10633	Human	Colombia
DENV-2/CO/BID-V1601/2005(2)	Dengue virus 2	EU854294	10636	Human	Colombia
DENV-2/CO/BID-V1603/2004(2)	Dengue virus 2	FJ024477	10647	Human	Colombia
DENV-2/CO/BID-V3358/1986(2)	Dengue virus 2	GQ868592	10667	Human	Colombia
DENV-2/CO/BID-V3368/1998(2)	Dengue virus 2	GQ868552	10663	Human	Colombia
DENV-2/CO/BID-V3369/1999(2)	Dengue virus 2	GQ868553	10625	Human	Colombia
DENV-2/CO/BID-V3370/2004(2)	Dengue virus 2	GQ868554	10628	Human	Colombia
DENV-2/CO/BID-V3371/2005(2)	Dengue virus 2	GQ868555	10668	Human	Colombia
DENV-2/CO/BID-V3372/2005(2)	Dengue virus 2	GQ868556	10679	Human	Colombia
DENV-2/CO/BID-V3373/2005(2)	Dengue virus 2	GQ868557	10668	Human	Colombia
DENV-2/CO/BID-V3374/2007(2)	Dengue virus 2	GU131947	10623	Human	Colombia
DENV-2/CO/BID-V3375/2007(2)	Dengue virus 2	GQ868558	10679	Human	Colombia
DENV-2/CO/BID-V7294/2000	Dengue virus 2	KJ189305	10478	Human	Colombia
DENV-2/DO/BID-V2955/2003(2)	Dengue virus 2	FJ898451	10678	Human	Dominican Republic
DENV-2/GU/BID-V2950/2001	Dengue virus 2	HM488257	10967	Human	Guam
DENV-2/GU/FDA-GUA09/2009-2	Dengue virus 2	HQ999999	10725	Human	Guatemala
DENV-2/Harvard/BID-V2990/2009(2)	Dengue virus 2	FJ906966	10678	Human	-N/A-
DENV-2/Harvard/BID-V2991/2009(2)	Dengue virus 2	FJ906967	10678	Human	-N/A-
DENV-2/Harvard/BID-V2992/2009(2)	Dengue virus 2	FJ906968	10678	Human	-N/A-
DENV-2/Harvard/BID-V2993/2009(2)	Dengue virus 2	FJ906969	10678	Human	-N/A-
DENV-2/HN/BID-V2945/1984(2)	Dengue virus 2	FJ898449	10668	Human	Honduras
DENV-2/ID/1016DN/1975(2)	Dengue virus 2	GQ398258	10723	Human	Indonesia
DENV-2/ID/1017DN/1976(2)	Dengue virus 2	GQ398259	10723	Human	Indonesia
DENV-2/ID/1022DN/1975(2)	Dengue virus 2	GQ398268	10724	Human	Indonesia
DENV-2/ID/1023DN/1975(2)	Dengue virus 2	GQ398263	10723	Human	Indonesia
DENV-2/ID/1046DN/1976(2)	Dengue virus 2	GQ398264	10723	Human	Indonesia
DENV-2/ID/1070DN/1976(2)	Dengue virus 2	GQ398260	10723	Human	Indonesia
DENV-2/ID/1127DN/1976(2)	Dengue virus 2	GQ398262	10723	Human	Indonesia
DENV-2/ID/1172DN/1976(2)	Dengue virus 2	GQ398261	10723	Human	Indonesia
DENV-2/ID/1183DN/1977(2)	Dengue virus 2	GQ398257	10713	Human	Indonesia
DENV-2/IN/BID-V2961/2006(2)	Dengue virus 2	FJ898454	10669	Human	India
DENV-2/IND/053598/2005-2	Dengue virus 2	JQ922551	10639	Human	India
DENV-2/IND/715394/1971-2	Dengue virus 2	JQ922550	10619	Human	India
DENV-2/IND/803347/1980-2	Dengue virus 2	JQ922553	10591	Human	India
DENV-2/IND/969201/1996-2	Dengue virus 2	JQ922549	10648	Human	India
DENV-2/IND/P23085/1960-2	Dengue virus 2	JQ922552	10675	Human	India
DENV-2/IPC/BID-V3788/2007(2)	Dengue virus 2	GU131896	10411	Human	Cambodia
DENV-2/IPC/BID-V3789/2007(2)	Dengue virus 2	GU131897	10428	Human	Cambodia
DENV-2/IPC/BID-V3791/2008(2)	Dengue virus 2	GQ868631	10669	Human	Cambodia
DENV-2/IPC/BID-V3795/2008(2)	Dengue virus 2	GU131898	10643	Human	Cambodia
DENV-2/IPC/BID-V3796/2008(2)	Dengue virus 2	GU131899	10646	Human	Cambodia
DENV-2/IPC/BID-V3797/2008(2)	Dengue virus 2	GU131900	10658	Human	Cambodia
DENV-2/IPC/BID-V3798/2008(2)	Dengue virus 2	GU131901	10630	Human	Cambodia
DENV-2/IPC/BID-V3799/2008(2)	Dengue virus 2	GU131902	10679	Human	Cambodia
DENV-2/IPC/BID-V3922/2008(2)	Dengue virus 2	GU131924	10557	Human	Cambodia
DENV-2/IPC/BID-V3924/2008(2)	Dengue virus 2	GQ868638	10667	Human	Cambodia
DENV-2/IPC/BID-V4265/2007(2)	Dengue virus 2	GU131927	10531	Human	Cambodia
DENV-2/IPC/BID-V4266/2008(2)	Dengue virus 2	GU131928	10494	Human	Cambodia

DENV-2/IPC/BID-V4268/2008(2)	Dengue virus 2	GU131929	10603	Human	Cambodia
DENV-2/IPC/BID-V4270/2008(2)	Dengue virus 2	GU131930	10532	Human	Cambodia
DENV-2/IPC/BID-V4271/2008(2)	Dengue virus 2	GU131931	10531	Human	Cambodia
DENV-2/IPC/BID-V4277/2008(2)	Dengue virus 2	GU131932	10531	Human	Cambodia
DENV-2/JM/BID-V2963/2007(2)	Dengue virus 2	GQ199892	10750	Human	Jamaica
DENV-2/KBPV-VR-29-2	Dengue virus 2	KP406804	10712	Unknown	South Korea
DENV-2/KH/BID-V2019/2001-2	Dengue virus 2	JF730044	10475	Human	Cambodia
DENV-2/KH/BID-V2020/2001(2)	Dengue virus 2	FJ639697	10667	Human	Cambodia
DENV-2/KH/BID-V2021/2002(2)	Dengue virus 2	FJ639698	10679	Human	Cambodia
DENV-2/KH/BID-V2022/2002(2)	Dengue virus 2	FJ639699	10680	Human	Cambodia
DENV-2/KH/BID-V2023/2002(2)	Dengue virus 2	FJ639700	10679	Human	Cambodia
DENV-2/KH/BID-V2024/2002(2)	Dengue virus 2	FJ639701	10681	Human	Cambodia
DENV-2/KH/BID-V2028/2003(2)	Dengue virus 2	GQ868620	10663	Human	Cambodia
DENV-2/KH/BID-V2030/2003(2)	Dengue virus 2	FJ639702	10667	Human	Cambodia
DENV-2/KH/BID-V2033/2003(2)	Dengue virus 2	FJ639703	10663	Human	Cambodia
DENV-2/KH/BID-V2034/2003(2)	Dengue virus 2	GQ868621	10678	Human	Cambodia
DENV-2/KH/BID-V2036/2003(2)	Dengue virus 2	FJ639704	10672	Human	Cambodia
DENV-2/KH/BID-V2037/2003(2)	Dengue virus 2	GQ868622	10679	Human	Cambodia
DENV-2/KH/BID-V2039/2003(2)	Dengue virus 2	FJ639705	10678	Human	Cambodia
DENV-2/KH/BID-V2040/2004(2)	Dengue virus 2	FJ639706	10677	Human	Cambodia
DENV-2/KH/BID-V2041/2004(2)	Dengue virus 2	FJ639707	10677	Human	Cambodia
DENV-2/KH/BID-V2042/2005(2)	Dengue virus 2	FJ639708	10677	Human	Cambodia
DENV-2/KH/BID-V2043/2005(2)	Dengue virus 2	FJ639709	10683	Human	Cambodia
DENV-2/KH/BID-V2044/2005(2)	Dengue virus 2	FJ639710	10658	Human	Cambodia
DENV-2/KH/BID-V2045/2005(2)	Dengue virus 2	FJ639711	10666	Human	Cambodia
DENV-2/KH/BID-V2047/2005(2)	Dengue virus 2	GQ868623	10677	Human	Cambodia
DENV-2/KH/BID-V2062/2007(2)	Dengue virus 2	GQ868624	10679	Human	Cambodia
DENV-2/KH/BID-V2066/2007(2)	Dengue virus 2	FJ639717	10678	Human	Cambodia
DENV-2/KH/BID-V2068/2008(2)	Dengue virus 2	FJ639718	10703	Human	Cambodia
DENV-2/KH/BID-V2069/2008(2)	Dengue virus 2	GQ868625	10675	Human	Cambodia
DENV-2/KH/BID-V4267/2008-2	Dengue virus 2	JF730045	10489	Human	Cambodia
DENV-2/KH/BID-V4269/2008	Dengue virus 2	KF955401	10558	Human	Cambodia
DENV-2/KH/BID-V4272/2008-2	Dengue virus 2	JF730046	10477	Human	Cambodia
DENV-2/KH/BID-V4273/2008-2	Dengue virus 2	JF730047	10477	Human	Cambodia
DENV-2/KH/BID-V4274/2008-2	Dengue virus 2	JF730048	10475	Human	Cambodia
DENV-2/KH/BID-V4276/2008	Dengue virus 2	KF955402	10505	Human	Cambodia
DENV-2/KN/BID-V2951/2001(2)	Dengue virus 2	FJ898460	10678	Human	Saint Kitts and Nevis
DENV-2/LK/BID-V2416/1996(2)	Dengue virus 2	FJ882602	10677	Human	Sri Lanka
DENV-2/LK/BID-V2421/2003(2)	Dengue virus 2	GQ252676	10629	Human	Sri Lanka
DENV-2/LK/BID-V2422/2004(2)	Dengue virus 2	GQ252677	10628	Human	Sri Lanka
DENV-2/MX/BID-V2953/2002(2)	Dengue virus 2	FJ898438	10665	Human	Mexico
DENV-2/MX/BID-V2954/2002(2)	Dengue virus 2	GQ199893	10679	Human	Mexico
DENV-2/MX/BID-V2959/2005(2)	Dengue virus 2	GQ199894	10669	Human	Mexico
DENV-2/MX/BID-V2964/2008(2)	Dengue virus 2	FJ898439	10680	Human	Mexico
DENV-2/MX/BID-V3354/1983(2)	Dengue virus 2	GQ868588	10667	Human	Mexico
DENV-2/MX/BID-V3355/1983(2)	Dengue virus 2	GQ868589	10668	Human	Mexico
DENV-2/MX/BID-V3356/1992(2)	Dengue virus 2	GQ868590	10668	Human	Mexico
DENV-2/MX/BID-V3654/2006(2)	Dengue virus 2	GQ868497	10664	Human	Mexico
DENV-2/MX/BID-V3661/2006(2)	Dengue virus 2	GU131959	10628	Human	Mexico
DENV-2/MX/BID-V3713/2007(2)	Dengue virus 2	GQ868515	10671	Human	Mexico
DENV-2/MX/BID-V3714/2007(2)	Dengue virus 2	GQ868516	10673	Human	Mexico
DENV-2/MX/BID-V3715/2007(2)	Dengue virus 2	GU131974	10667	Human	Mexico
DENV-2/MX/BID-V3717/2007(2)	Dengue virus 2	GU131975	10668	Human	Mexico
DENV-2/MX/BID-V3719/2007	Dengue virus 2	KF955395	10649	Human	Mexico
DENV-2/MX/BID-V3763/2008	Dengue virus 2	JN819422	10519	Human	Mexico
DENV-2/MX/BID-V3768/2004(2)	Dengue virus 2	GU131955	10523	Human	Mexico
DENV-2/MX/BID-V7535/2009	Dengue virus 2	KJ189308	10477	Mosquito	Mexico
DENV-2/MX/BID-V7537/2010	Dengue virus 2	KJ189309	10300	Mosquito	Mexico
DENV-2/MX/BID-V7540/2007	Dengue virus 2	KJ189310	10520	Human	Mexico
DENV-2/MX/BID-V7542/2007	Dengue virus 2	KJ189311	10715	Human	Mexico
DENV-2/MX/BID-V8199/2011	Dengue virus 2	KJ189370	10481	Human	Mexico
DENV-2/NI/BID-V1074/2006(2)	Dengue virus 2	EU482620	10680	Human	Nicaragua
DENV-2/NI/BID-V1191/2007(2)	Dengue virus 2	EU482621	10680	Human	Nicaragua
DENV-2/NI/BID-V1192/2007(2)	Dengue virus 2	EU569692	10674	Human	Nicaragua
DENV-2/NI/BID-V1193/2007(2)	Dengue virus 2	EU569693	10680	Human	Nicaragua
DENV-2/NI/BID-V1194/2007(2)	Dengue virus 2	FJ373300	10679	Human	Nicaragua
DENV-2/NI/BID-V1195/2007(2)	Dengue virus 2	EU596495	10679	Human	Nicaragua
DENV-2/NI/BID-V1196/2007(2)	Dengue virus 2	EU660404	10709	Human	Nicaragua
DENV-2/NI/BID-V1197/2007(2)	Dengue virus 2	FJ850116	10660	Human	Nicaragua
DENV-2/NI/BID-V1198/2007(2)	Dengue virus 2	EU482622	10679	Human	Nicaragua
DENV-2/NI/BID-V1199/2007(2)	Dengue virus 2	EU569694	10680	Human	Nicaragua
DENV-2/NI/BID-V1200/2007(2)	Dengue virus 2	EU596496	10681	Human	Nicaragua
DENV-2/NI/BID-V1201/2007(2)	Dengue virus 2	EU660405	10680	Human	Nicaragua
DENV-2/NI/BID-V1202/2007(2)	Dengue virus 2	EU596497	10680	Human	Nicaragua
DENV-2/NI/BID-V1203/2007(2)	Dengue virus 2	EU569695	10680	Human	Nicaragua
DENV-2/NI/BID-V1205/2007(2)	Dengue virus 2	FJ373301	10706	Human	Nicaragua
DENV-2/NI/BID-V1206/2007(2)	Dengue virus 2	EU569696	10664	Human	Nicaragua
DENV-2/NI/BID-V1207/2007(2)	Dengue virus 2	EU660406	10679	Human	Nicaragua
DENV-2/NI/BID-V1208/2007(2)	Dengue virus 2	EU569697	10680	Human	Nicaragua
DENV-2/NI/BID-V1210/2007(2)	Dengue virus 2	EU482623	10681	Human	Nicaragua
DENV-2/NI/BID-V1211/2007(2)	Dengue virus 2	EU596498	10680	Human	Nicaragua
DENV-2/NI/BID-V1212/2007(2)	Dengue virus 2	EU569698	10680	Human	Nicaragua
DENV-2/NI/BID-V1214/2007(2)	Dengue virus 2	FJ478459	10680	Human	Nicaragua
DENV-2/NI/BID-V1215/2007(2)	Dengue virus 2	EU596499	10663	Human	Nicaragua
DENV-2/NI/BID-V1217/2007(2)	Dengue virus 2	EU569699	10678	Human	Nicaragua
DENV-2/NI/BID-V1218/2007	Dengue virus 2	KF955366	10658	Human	Nicaragua
DENV-2/NI/BID-V1219/2007(2)	Dengue virus 2	EU569700	10679	Human	Nicaragua
DENV-2/NI/BID-V1224/2007(2)	Dengue virus 2	EU569701	10680	Human	Nicaragua
DENV-2/NI/BID-V1225/2007(2)	Dengue virus 2	EU621672	10711	Human	Nicaragua
DENV-2/NI/BID-V1226/2007	Dengue virus 2	KF955369	10651	Human	Nicaragua
DENV-2/NI/BID-V1228/2007(2)	Dengue virus 2	EU569702	10679	Human	Nicaragua
DENV-2/NI/BID-V1229/2007(2)	Dengue virus 2	EU482624	10680	Human	Nicaragua
DENV-2/NI/BID-V1230/2007(2)	Dengue virus 2	EU596500	10680	Human	Nicaragua
DENV-2/NI/BID-V1232/2007(2)	Dengue virus 2	EU482625	10680	Human	Nicaragua
DENV-2/NI/BID-V1233/2007(2)	Dengue virus 2	FJ410291	10679	Human	Nicaragua
DENV-2/NI/BID-V1234/2007(2)	Dengue virus 2	EU482626	10679	Human	Nicaragua
DENV-2/NI/BID-V1235/2007(2)	Dengue virus 2	EU482627	10680	Human	Nicaragua
DENV-2/NI/BID-V1236/2007(2)	Dengue virus 2	EU482628	10680	Human	Nicaragua
DENV-2/NI/BID-V1237/2007(2)	Dengue virus 2	FJ639833	10679	Human	Nicaragua
DENV-2/NI/BID-V1297/2007(2)	Dengue virus 2	EU482629	10679	Human	Nicaragua
DENV-2/NI/BID-V1298/2007(2)	Dengue virus 2	FJ390390	10679	Human	Nicaragua
DENV-2/NI/BID-V1300/2007(2)	Dengue virus 2	FJ390391	10680	Human	Nicaragua
DENV-2/NI/BID-V1302/2007(2)	Dengue virus 2	FJ882593	10680	Human	Nicaragua
DENV-2/NI/BID-V1303/2007(2)	Dengue virus 2	FJ182014	10680	Human	Nicaragua
DENV-2/NI/BID-V1304/2007(2)	Dengue virus 2	FJ898432	10443	Human	Nicaragua
DENV-2/NI/BID-V1311/2007(2)	Dengue virus 2	FJ547090	10641	Human	Nicaragua
DENV-2/NI/BID-V1312/2007(2)	Dengue virus 2	EU482630	10680	Human	Nicaragua
DENV-2/NI/BID-V1313/2007(2)	Dengue virus 2	FJ882594	10648	Human	Nicaragua
DENV-2/NI/BID-V1721/2008(2)	Dengue virus 2	FJ205885	10680	Human	Nicaragua
DENV-2/NI/BID-V1755/2007(2)	Dengue virus 2	FJ744743	10727	Human	Nicaragua
DENV-2/NI/BID-V1761/2006	Dengue virus 2	JN819424	10679	Human	Nicaragua
DENV-2/NI/BID-V1762/2006(2)	Dengue virus 2	FJ744741	10679	Human	Nicaragua
DENV-2/NI/BID-V1763/2006(2)	Dengue virus 2	FJ744742	10679	Human	Nicaragua

DENV-2/NI/BID-V1764/2007(2)	Dengue virus 2	FJ744703	10673	Human	Nicaragua
DENV-2/NI/BID-V2331/2006(2)	Dengue virus 2	FJ850067	10679	Human	Nicaragua
DENV-2/NI/BID-V2344/2000(2)	Dengue virus 2	FJ850060	10729	Human	Nicaragua
DENV-2/NI/BID-V2346/2000(2)	Dengue virus 2	FJ850061	10690	Human	Nicaragua
DENV-2/NI/BID-V2351/2008(2)	Dengue virus 2	FJ744709	10640	Human	Nicaragua
DENV-2/NI/BID-V2352/2007(2)	Dengue virus 2	FJ744708	10689	Human	Nicaragua
DENV-2/NI/BID-V2353/2008(2)	Dengue virus 2	FJ810418	10677	Human	Nicaragua
DENV-2/NI/BID-V2354/2007(2)	Dengue virus 2	FJ639834	10665	Human	Nicaragua
DENV-2/NI/BID-V2355/2007(2)	Dengue virus 2	FJ639835	10680	Human	Nicaragua
DENV-2/NI/BID-V2356/2007(2)	Dengue virus 2	FJ850050	10660	Human	Nicaragua
DENV-2/NI/BID-V2357/2007(2)	Dengue virus 2	FJ639836	10680	Human	Nicaragua
DENV-2/NI/BID-V2358/2007(2)	Dengue virus 2	FJ639837	10679	Human	Nicaragua
DENV-2/NI/BID-V2359/2007(2)	Dengue virus 2	FJ744706	10679	Human	Nicaragua
DENV-2/NI/BID-V2360/2007(2)	Dengue virus 2	FJ744707	10678	Human	Nicaragua
DENV-2/NI/BID-V2361/2001(2)	Dengue virus 2	FJ744704	10664	Human	Nicaragua
DENV-2/NI/BID-V2362/2000(2)	Dengue virus 2	FJ744745	10679	Human	Nicaragua
DENV-2/NI/BID-V2363/2000(2)	Dengue virus 2	FJ744705	10669	Human	Nicaragua
DENV-2/NI/BID-V2364/2000(2)	Dengue virus 2	FJ744744	10664	Human	Nicaragua
DENV-2/NI/BID-V2427/2007(2)	Dengue virus 2	GQ199866	10670	Human	Nicaragua
DENV-2/NI/BID-V2428/2007(2)	Dengue virus 2	GQ868646	10678	Human	Nicaragua
DENV-2/NI/BID-V2569/2007(2)	Dengue virus 2	FJ906960	10660	Human	Nicaragua
DENV-2/NI/BID-V2572/2005(2)	Dengue virus 2	FJ906961	10661	Human	Nicaragua
DENV-2/NI/BID-V2573/2005(2)	Dengue virus 2	FJ906962	10678	Human	Nicaragua
DENV-2/NI/BID-V2574/2005(2)	Dengue virus 2	FJ850053	10679	Human	Nicaragua
DENV-2/NI/BID-V2576/2005(2)	Dengue virus 2	FJ850054	10679	Human	Nicaragua
DENV-2/NI/BID-V2596/2006	Dengue virus 2	HQ541786	10694	Human	Nicaragua
DENV-2/NI/BID-V2599/2006	Dengue virus 2	HQ733861	10731	Human	Nicaragua
DENV-2/NI/BID-V2605/2007	Dengue virus 2	JF357905	10669	Human	Nicaragua
DENV-2/NI/BID-V2635/2007(2)	Dengue virus 2	FJ850051	10679	Human	Nicaragua
DENV-2/NI/BID-V2639/2007	Dengue virus 2	JN819420	10487	Human	Nicaragua
DENV-2/NI/BID-V2657/2000(2)	Dengue virus 2	FJ850117	10679	Human	Nicaragua
DENV-2/NI/BID-V2658/2000(2)	Dengue virus 2	FJ850118	10679	Human	Nicaragua
DENV-2/NI/BID-V2659/2000(2)	Dengue virus 2	FJ850062	10679	Human	Nicaragua
DENV-2/NI/BID-V2660/2000(2)	Dengue virus 2	FJ850063	10679	Human	Nicaragua
DENV-2/NI/BID-V2661/2000	Dengue virus 2	JN819416	10667	Human	Nicaragua
DENV-2/NI/BID-V2662/2000(2)	Dengue virus 2	FJ850119	10678	Human	Nicaragua
DENV-2/NI/BID-V2663/2000(2)	Dengue virus 2	FJ850064	10679	Human	Nicaragua
DENV-2/NI/BID-V2664/2000(2)	Dengue virus 2	FJ850065	10679	Human	Nicaragua
DENV-2/NI/BID-V2665/2000(2)	Dengue virus 2	FJ850066	10679	Human	Nicaragua
DENV-2/NI/BID-V2666/2000(2)	Dengue virus 2	FJ873808	10741	Human	Nicaragua
DENV-2/NI/BID-V2673/2001(2)	Dengue virus 2	FJ850120	10679	Human	Nicaragua
DENV-2/NI/BID-V2674/2001(2)	Dengue virus 2	FJ850121	10679	Human	Nicaragua
DENV-2/NI/BID-V2675/2002(2)	Dengue virus 2	GQ199897	10668	Human	Nicaragua
DENV-2/NI/BID-V2680/2006(2)	Dengue virus 2	GQ199896	10679	Human	Nicaragua
DENV-2/NI/BID-V2683/1999(2)	Dengue virus 2	GQ199895	10678	Human	Nicaragua
DENV-2/NI/BID-V2920/2007(2)	Dengue virus 2	FJ898434	10680	Human	Nicaragua
DENV-2/NI/BID-V2922/2007(2)	Dengue virus 2	FJ898479	10680	Human	Nicaragua
DENV-2/NI/BID-V2923/2000(2)	Dengue virus 2	FJ898477	10678	Human	Nicaragua
DENV-2/NI/BID-V2924/2000(2)	Dengue virus 2	FJ898478	10679	Human	Nicaragua
DENV-2/NI/BID-V2996/2007(2)	Dengue virus 2	GQ199868	10679	Human	Nicaragua
DENV-2/NI/BID-V2997/2007	Dengue virus 2	JN819421	10403	Human	Nicaragua
DENV-2/NI/BID-V3002/2007(2)	Dengue virus 2	GQ199869	10680	Human	Nicaragua
DENV-2/NI/BID-V3076/2001(2)	Dengue virus 2	GQ199898	10721	Human	Nicaragua
DENV-2/NI/BID-V3136/2007	Dengue virus 2	HQ541787	10498	Human	Nicaragua
DENV-2/NI/BID-V3143/2007	Dengue virus 2	HQ634199	10479	Human	Nicaragua
DENV-2/NI/BID-V3149/2007	Dengue virus 2	HQ541788	10487	Human	Nicaragua
DENV-2/NI/BID-V3152/2008	Dengue virus 2	JX079688	10477	Human	Nicaragua
DENV-2/NI/BID-V3223/2008	Dengue virus 2	HQ541792	10477	Human	Nicaragua
DENV-2/NI/BID-V3227/2008	Dengue virus 2	JF357906	10783	Human	Nicaragua
DENV-2/NI/BID-V3232/2007	Dengue virus 2	JX079690	10342	Human	Nicaragua
DENV-2/NI/BID-V4156/2007	Dengue virus 2	HM631865	10679	Human	Nicaragua
DENV-2/NI/BID-V4157/2006	Dengue virus 2	HM631866	10680	Human	Nicaragua
DENV-2/NI/BID-V4158/2006	Dengue virus 2	HM631867	10680	Human	Nicaragua
DENV-2/NI/BID-V4159/2005	Dengue virus 2	HM631868	10679	Human	Nicaragua
DENV-2/NI/BID-V4636/2005	Dengue virus 2	HQ541793	10485	Human	Nicaragua
DENV-2/NI/BID-V4637/2005	Dengue virus 2	JX079694	10478	Human	Nicaragua
DENV-2/NI/BID-V4639/2005	Dengue virus 2	HQ541794	10476	Human	Nicaragua
DENV-2/NI/BID-V4650/2007	Dengue virus 2	JF357907	10477	Human	Nicaragua
DENV-2/NI/BID-V4914/2009	Dengue virus 2	HQ705624	10479	Human	Nicaragua
DENV-2/NI/BID-V4915/2009	Dengue virus 2	HQ705625	10494	Human	Nicaragua
DENV-2/NI/BID-V5072/2009-2	Dengue virus 2	JF730051	10477	Human	Nicaragua
DENV-2/NI/BID-V5073/2009-2	Dengue virus 2	JF730052	10477	Human	Nicaragua
DENV-2/NI/BID-V513/2005(2)	Dengue virus 2	EU482748	10681	Human	Nicaragua
DENV-2/NI/BID-V514/2005(2)	Dengue virus 2	EU482749	10679	Human	Nicaragua
DENV-2/NI/BID-V515/2005(2)	Dengue virus 2	EU482750	10679	Human	Nicaragua
DENV-2/NI/BID-V517/2005(2)	Dengue virus 2	EU482751	10680	Human	Nicaragua
DENV-2/NI/BID-V518/2005(2)	Dengue virus 2	GQ868604	10679	Human	Nicaragua
DENV-2/NI/BID-V520/2005(2)	Dengue virus 2	EU482752	10679	Human	Nicaragua
DENV-2/NI/BID-V524/2005(2)	Dengue virus 2	EU482753	10677	Human	Nicaragua
DENV-2/NI/BID-V527/2005(2)	Dengue virus 2	EU482770	10678	Human	Nicaragua
DENV-2/NI/BID-V528/2005(2)	Dengue virus 2	FJ226066	10684	Human	Nicaragua
DENV-2/NI/BID-V529/2005(2)	Dengue virus 2	EU482680	10680	Human	Nicaragua
DENV-2/NI/BID-V530/2005(2)	Dengue virus 2	EU482754	10668	Human	Nicaragua
DENV-2/NI/BID-V531/2005(2)	Dengue virus 2	EU482755	10679	Human	Nicaragua
DENV-2/NI/BID-V532/2005(2)	Dengue virus 2	FJ478455	10678	Human	Nicaragua
DENV-2/NI/BID-V533/2005(2)	Dengue virus 2	EU482756	10679	Human	Nicaragua
DENV-2/NI/BID-V535/2005(2)	Dengue virus 2	EU482757	10675	Human	Nicaragua
DENV-2/NI/BID-V539/2005(2)	Dengue virus 2	EU482758	10679	Human	Nicaragua
DENV-2/NI/BID-V543/2005(2)	Dengue virus 2	FJ906956	10679	Human	Nicaragua
DENV-2/NI/BID-V544/2005(2)	Dengue virus 2	EU482759	10679	Human	Nicaragua
DENV-2/NI/BID-V548/2005(2)	Dengue virus 2	EU482760	10679	Human	Nicaragua
DENV-2/NI/BID-V549/2005(2)	Dengue virus 2	EU482681	10678	Human	Nicaragua
DENV-2/NI/BID-V553/2005(2)	Dengue virus 2	EU482761	10679	Human	Nicaragua
DENV-2/NI/BID-V554/2005(2)	Dengue virus 2	EU482762	10678	Human	Nicaragua
DENV-2/NI/BID-V556/2005(2)	Dengue virus 2	EU482763	10679	Human	Nicaragua
DENV-2/NI/BID-V557/2005(2)	Dengue virus 2	EU482771	10679	Human	Nicaragua
DENV-2/NI/BID-V559/2006(2)	Dengue virus 2	EU482682	10680	Human	Nicaragua
DENV-2/NI/BID-V563/2006(2)	Dengue virus 2	EU482444	10679	Human	Nicaragua
DENV-2/NI/BID-V565/2006(2)	Dengue virus 2	EU482772	10680	Human	Nicaragua
DENV-2/NI/BID-V566/2006(2)	Dengue virus 2	EU482773	10680	Human	Nicaragua
DENV-2/NI/BID-V570/2006(2)	Dengue virus 2	EU482683	10680	Human	Nicaragua
DENV-2/NI/BID-V571/2006(2)	Dengue virus 2	EU482684	10679	Human	Nicaragua
DENV-2/NI/BID-V572/2006(2)	Dengue virus 2	EU482685	10678	Human	Nicaragua
DENV-2/NI/BID-V573/2006(2)	Dengue virus 2	EU482686	10680	Human	Nicaragua
DENV-2/NI/BID-V574/2006(2)	Dengue virus 2	EU482687	10679	Human	Nicaragua
DENV-2/NI/BID-V575/2006(2)	Dengue virus 2	EU482688	10678	Human	Nicaragua
DENV-2/NI/BID-V576/2006(2)	Dengue virus 2	EU482689	10680	Human	Nicaragua
DENV-2/NI/BID-V578/2006(2)	Dengue virus 2	EU482690	10680	Human	Nicaragua
DENV-2/NI/BID-V579/2006(2)	Dengue virus 2	EU482691	10680	Human	Nicaragua
DENV-2/NI/BID-V580/2006(2)	Dengue virus 2	EU482692	10680	Human	Nicaragua
DENV-2/NI/BID-V581/2006(2)	Dengue virus 2	EU482693	10673	Human	Nicaragua
DENV-2/NI/BID-V582/2007(2)	Dengue virus 2	EU482694	10680	Human	Nicaragua
DENV-2/NI/BID-V608/2006(2)	Dengue virus 2	EU596483	10680	Human	Nicaragua

DENV-2/NI/BID-V609/2005(2)	Dengue virus 2	EU482597	10679	Human	Nicaragua
DENV-2/NI/BID-V613/2005(2)	Dengue virus 2	EU482598	10679	Human	Nicaragua
DENV-2/NI/BID-V615/2007(2)	Dengue virus 2	EU482603	10679	Human	Nicaragua
DENV-2/NI/BID-V618/2005(2)	Dengue virus 2	EU482599	10679	Human	Nicaragua
DENV-2/NI/BID-V620/2005(2)	Dengue virus 2	EU482632	10679	Human	Nicaragua
DENV-2/NI/BID-V623/2005(2)	Dengue virus 2	EU482600	10679	Human	Nicaragua
DENV-2/NI/BID-V627/2004(2)	Dengue virus 2	FJ898436	10679	Human	Nicaragua
DENV-2/NI/BID-V631/2006(2)	Dengue virus 2	EU482633	10678	Human	Nicaragua
DENV-2/NI/BID-V633/2006(2)	Dengue virus 2	EU482634	10680	Human	Nicaragua
DENV-2/NI/BID-V634/2005(2)	Dengue virus 2	FJ850115	10678	Human	Nicaragua
DENV-2/NI/BID-V640/2005(2)	Dengue virus 2	EU482635	10679	Human	Nicaragua
DENV-2/NI/BID-V645/2005	Dengue virus 2	KF955362	10740	Human	Nicaragua
DENV-2/NI/BID-V648/2005(2)	Dengue virus 2	FJ898435	10679	Human	Nicaragua
DENV-2/NI/BID-V654/2005(2)	Dengue virus 2	EU482636	10679	Human	Nicaragua
DENV-2/NI/BID-V658/2005(2)	Dengue virus 2	EU482637	10678	Human	Nicaragua
DENV-2/NI/BID-V660/2005(2)	Dengue virus 2	EU596484	10678	Human	Nicaragua
DENV-2/NI/BID-V662/2007(2)	Dengue virus 2	EU482638	10680	Human	Nicaragua
DENV-2/NI/BID-V663/2007(2)	Dengue virus 2	EU482601	10680	Human	Nicaragua
DENV-2/NI/BID-V668/2004(2)	Dengue virus 2	GQ199874	10667	Human	Nicaragua
DENV-2/NI/BID-V670/2005(2)	Dengue virus 2	EU482631	10679	Human	Nicaragua
DENV-2/NI/BID-V672/2005(2)	Dengue virus 2	EU482602	10679	Human	Nicaragua
DENV-2/NI/BID-V673/2006(2)	Dengue virus 2	EU482639	10679	Human	Nicaragua
DENV-2/NI/BID-V693/2005(2)	Dengue virus 2	EU482766	10679	Human	Nicaragua
DENV-2/NI/BID-V743/2005(2)	Dengue virus 2	EU482695	10670	Human	Nicaragua
DENV-2/NI/BID-V744/2005(2)	Dengue virus 2	EU482769	10679	Human	Nicaragua
DENV-2/NI/BID-V747/2006(2)	Dengue virus 2	EU482696	10666	Human	Nicaragua
DENV-2/PE/FMD1337/2007-2	Dengue virus 2	KC294200	10724	Human	Peru
DENV-2/PE/FMD2303/2009-2	Dengue virus 2	KC294201	10723	Human	Peru
DENV-2/PE/FPI00073/2010-2	Dengue virus 2	KC294202	10723	Human	Peru
DENV-2/PE/FPI00154/2010-2	Dengue virus 2	KC294203	10723	Human	Peru
DENV-2/PE/FPI00174/2011-2	Dengue virus 2	KC294204	10723	Human	Peru
DENV-2/PE/FPI01202/2011-2	Dengue virus 2	KC294207	10723	Human	Peru
DENV-2/PE/FPI01345/2011-2	Dengue virus 2	KC294208	10723	Human	Peru
DENV-2/PE/FPI01399/2011-2	Dengue virus 2	KC294209	10723	Human	Peru
DENV-2/PE/FPI01695/2011-2	Dengue virus 2	KC294211	10723	Human	Peru
DENV-2/PE/FPI01777/2011-2	Dengue virus 2	KC294212	10723	Human	Peru
DENV-2/PE/FPI01851/2011-2	Dengue virus 2	KC294213	10723	Human	Peru
DENV-2/PE/FPI01915/2011-2	Dengue virus 2	KC294214	10723	Human	Peru
DENV-2/PE/FPI0492/2011-2	Dengue virus 2	KC294205	10723	Human	Peru
DENV-2/PE/FPI1049/2011-2	Dengue virus 2	KC294206	10723	Human	Peru
DENV-2/PE/FPI1617/2011-2	Dengue virus 2	KC294210	10723	Human	Peru
DENV-2/PE/FPY00016/2010-2	Dengue virus 2	KC294215	10723	Human	Peru
DENV-2/PE/FSL 4862/2010-2	Dengue virus 2	KC294217	10723	Human	Peru
DENV-2/PE/FSL699/2002-2	Dengue virus 2	KC294216	10723	Human	Peru
DENV-2/PE/IDA 1081/2011-2	Dengue virus 2	KC294218	10723	Human	Peru
DENV-2/PE/IQA 1995/2010-2	Dengue virus 2	KC294219	10723	Human	Peru
DENV-2/PE/IQA 2042/2010-2	Dengue virus 2	KC294220	10723	Human	Peru
DENV-2/PE/IQA 2080/2010-2	Dengue virus 2	KC294221	10723	Human	Peru
DENV-2/PE/NFI 52/2002-2	Dengue virus 2	KC294222	10723	Human	Peru
DENV-2/PE/NFI1159/2010-2	Dengue virus 2	KC294223	10723	Human	Peru
DENV-2/PG/BID-V2618/2008(2)	Dengue virus 2	FJ906959	10678	Human	Papua New Guinea
DENV-2/PK-2	Dengue virus 2	KF360005	10629	Human	Pakistan
DENV-2/PK/2011-2	Dengue virus 2	KJ010185	10629	Human	Pakistan
DENV-2/PK/2013-2	Dengue virus 2	KJ010186	10629	Human	Pakistan
DENV-2/Pk/Swat-02-2	Dengue virus 2	KJ701507	10656	Human	Pakistan
DENV-2/PR/10DN/1994(2)	Dengue virus 2	GQ398288	10722	Human	Puerto Rico
DENV-2/PR/11DN/1994(2)	Dengue virus 2	GQ398313	10723	Human	Puerto Rico
DENV-2/PR/12DN/1994(2)	Dengue virus 2	GQ398285	10723	Human	Puerto Rico
DENV-2/PR/13DN/1994(2)	Dengue virus 2	GQ398314	10723	Human	Puerto Rico
DENV-2/PR/14DN/1994(2)	Dengue virus 2	GQ398295	10723	Human	Puerto Rico
DENV-2/PR/15DN/1994(2)	Dengue virus 2	GQ398271	10723	Human	Puerto Rico
DENV-2/PR/16DN/1995(2)	Dengue virus 2	GQ398298	10723	Human	Puerto Rico
DENV-2/PR/17DN/1995(2)	Dengue virus 2	GQ398301	10723	Human	Puerto Rico
DENV-2/PR/19DN/1994(2)	Dengue virus 2	GQ398307	10723	Human	Puerto Rico
DENV-2/PR/1DN/1994(2)	Dengue virus 2	GQ398309	10724	Human	Puerto Rico
DENV-2/PR/21DN/1994(2)	Dengue virus 2	GQ398297	10723	Human	Puerto Rico
DENV-2/PR/22DN/1994(2)	Dengue virus 2	GQ398306	10723	Human	Puerto Rico
DENV-2/PR/23DN/1994(2)	Dengue virus 2	GQ398274	10723	Human	Puerto Rico
DENV-2/PR/24DN/1994(2)	Dengue virus 2	GQ398279	10723	Human	Puerto Rico
DENV-2/PR/25DN/1994(2)	Dengue virus 2	GQ398299	10723	Human	Puerto Rico
DENV-2/PR/26DN/1994(2)	Dengue virus 2	GQ398276	10723	Human	Puerto Rico
DENV-2/PR/27DN/1994(2)	Dengue virus 2	GQ398281	10723	Human	Puerto Rico
DENV-2/PR/28DN/1994(2)	Dengue virus 2	GQ398312	10724	Human	Puerto Rico
DENV-2/PR/29DN/1994(2)	Dengue virus 2	GQ398291	10722	Human	Puerto Rico
DENV-2/PR/2DN/1994(2)	Dengue virus 2	GQ398270	10724	Human	Puerto Rico
DENV-2/PR/30DN/1994(2)	Dengue virus 2	GQ398289	10722	Human	Puerto Rico
DENV-2/PR/31DN/1994(2)	Dengue virus 2	GQ398290	10722	Human	Puerto Rico
DENV-2/PR/33DN/1994(2)	Dengue virus 2	GQ398275	10723	Human	Puerto Rico
DENV-2/PR/34DN/1994(2)	Dengue virus 2	GQ398303	10723	Human	Puerto Rico
DENV-2/PR/35DN/1994(2)	Dengue virus 2	GQ398282	10722	Human	Puerto Rico
DENV-2/PR/36DN/1994(2)	Dengue virus 2	GQ398284	10723	Human	Puerto Rico
DENV-2/PR/37DN/1994(2)	Dengue virus 2	GQ398283	10722	Human	Puerto Rico
DENV-2/PR/38DN/1994(2)	Dengue virus 2	GQ398277	10723	Human	Puerto Rico
DENV-2/PR/39DN/1994(2)	Dengue virus 2	GQ398280	10723	Human	Puerto Rico
DENV-2/PR/3DN/1994(2)	Dengue virus 2	GQ398308	10724	Human	Puerto Rico
DENV-2/PR/40DN/1994(2)	Dengue virus 2	GQ398278	10723	Human	Puerto Rico
DENV-2/PR/42DN/1994(2)	Dengue virus 2	GQ398286	10722	Human	Puerto Rico
DENV-2/PR/43DN/1994(2)	Dengue virus 2	GQ398293	10723	Human	Puerto Rico
DENV-2/PR/44DN/1994(2)	Dengue virus 2	GQ398296	10723	Human	Puerto Rico
DENV-2/PR/45DN/1994(2)	Dengue virus 2	GQ398294	10723	Human	Puerto Rico
DENV-2/PR/46DN/1994(2)	Dengue virus 2	GQ398287	10722	Human	Puerto Rico
DENV-2/PR/47DN/1994(2)	Dengue virus 2	GQ398302	10723	Human	Puerto Rico
DENV-2/PR/48DN/1994(2)	Dengue virus 2	GQ398310	10724	Human	Puerto Rico
DENV-2/PR/49DN/1994(2)	Dengue virus 2	GQ398272	10723	Human	Puerto Rico
DENV-2/PR/4DN/1994(2)	Dengue virus 2	GQ398304	10723	Human	Puerto Rico
DENV-2/PR/50DN/1994(2)	Dengue virus 2	GQ398269	10723	Human	Puerto Rico
DENV-2/PR/5DN/1994(2)	Dengue virus 2	GQ398305	10723	Human	Puerto Rico
DENV-2/PR/6780DN/1994(2)	Dengue virus 2	GQ398311	10724	Human	Puerto Rico
DENV-2/PR/6DN/1994(2)	Dengue virus 2	GQ398292	10723	Human	Puerto Rico
DENV-2/PR/8DN/1994(2)	Dengue virus 2	GQ398273	10723	Human	Puerto Rico
DENV-2/PR/9DN/1994(2)	Dengue virus 2	GQ398300	10723	Human	Puerto Rico
DENV-2/PR/BID-V1037/2006	Dengue virus 2	KF955364	10521	Human	Puerto Rico
DENV-2/PR/BID-V1406/1997	Dengue virus 2	KF955372	10646	Human	Puerto Rico
DENV-2/PR/BID-V1408/1997	Dengue virus 2	KF955373	10991	Human	Puerto Rico
DENV-2/PR/BID-V3367/1969(2)	Dengue virus 2	GQ868600	10636	Human	Puerto Rico
DENV-2/PR/BID-V586/2006	Dengue virus 2	KF955359	10531	Human	Puerto Rico
DENV-2/PR/BID-V590/2006	Dengue virus 2	KF955360	10531	Human	Puerto Rico
DENV-2/PR/BID-V856/1986	Dengue virus 2	KF955363	10531	Human	Puerto Rico
DENV-2/SG/07K3588DK1/2007(2)	Dengue virus 2	GQ398267	10723	Human	Singapore
DENV-2/SG/07K3598DK2/2007(2)	Dengue virus 2	GQ398266	10723	Human	Singapore
DENV-2/SG/07K3608DK1/2008(2)	Dengue virus 2	GQ398265	10723	Human	Singapore
DENV-2/SG/D2Y98P-PP1/2009-DENV-2	Dengue virus 2	JF327392	10723	Unknown	Singapore
DENV-2/TH/BID-V1458/1994(2)	Dengue virus 2	EU726767	10678	Human	Thailand

DENV-2/TH/BID-V1498/1994(2)	Dengue virus 2	EU687246	10679	Human	Thailand
DENV-2/TH/BID-V2153/2001(2)	Dengue virus 2	FJ639828	10670	Human	Thailand
DENV-2/TH/BID-V2154/2001(2)	Dengue virus 2	FJ639829	10679	Human	Thailand
DENV-2/TH/BID-V2155/2001(2)	Dengue virus 2	FJ639830	10679	Human	Thailand
DENV-2/TH/BID-V2156/2001(2)	Dengue virus 2	FJ639831	10660	Human	Thailand
DENV-2/TH/BID-V2157/2001(2)	Dengue virus 2	FJ639832	10658	Human	Thailand
DENV-2/TH/BID-V2278/2001(2)	Dengue virus 2	FJ687434	10679	Human	Thailand
DENV-2/TH/BID-V2279/2001(2)	Dengue virus 2	FJ687435	10679	Human	Thailand
DENV-2/TH/BID-V2280/2001(2)	Dengue virus 2	FJ687436	10679	Human	Thailand
DENV-2/TH/BID-V2281/2001(2)	Dengue virus 2	FJ687437	10678	Human	Thailand
DENV-2/TH/BID-V2282/2001(2)	Dengue virus 2	FJ687438	10678	Human	Thailand
DENV-2/TH/BID-V2283/2001(2)	Dengue virus 2	FJ687439	10677	Human	Thailand
DENV-2/TH/BID-V2284/2001(2)	Dengue virus 2	FJ687440	10679	Human	Thailand
DENV-2/TH/BID-V2285/2001(2)	Dengue virus 2	FJ810409	10679	Human	Thailand
DENV-2/TH/BID-V2286/2001(2)	Dengue virus 2	FJ687441	10679	Human	Thailand
DENV-2/TH/BID-V2287/2001(2)	Dengue virus 2	FJ687442	10679	Human	Thailand
DENV-2/TH/BID-V2288/2001(2)	Dengue virus 2	FJ687443	10679	Human	Thailand
DENV-2/TH/BID-V2289/2001(2)	Dengue virus 2	FJ687444	10661	Human	Thailand
DENV-2/TH/BID-V2290/2001(2)	Dengue virus 2	FJ687445	10679	Human	Thailand
DENV-2/TH/BID-V2291/2001(2)	Dengue virus 2	FJ810410	10703	Human	Thailand
DENV-2/TH/BID-V2292/2001(2)	Dengue virus 2	FJ687446	10679	Human	Thailand
DENV-2/TH/BID-V2293/2001(2)	Dengue virus 2	FJ687447	10678	Human	Thailand
DENV-2/TH/BID-V2294/2001(2)	Dengue virus 2	FJ744710	10678	Human	Thailand
DENV-2/TH/BID-V2295/2001(2)	Dengue virus 2	FJ744711	10678	Human	Thailand
DENV-2/TH/BID-V2296/2001(2)	Dengue virus 2	FJ744712	10678	Human	Thailand
DENV-2/TH/BID-V2297/2001(2)	Dengue virus 2	FJ810411	10678	Human	Thailand
DENV-2/TH/BID-V2298/2001(2)	Dengue virus 2	FJ744713	10678	Human	Thailand
DENV-2/TH/BID-V2299/2001(2)	Dengue virus 2	FJ810412	10737	Human	Thailand
DENV-2/TH/BID-V2300/2001(2)	Dengue virus 2	FJ744714	10678	Human	Thailand
DENV-2/TH/BID-V2301/2001(2)	Dengue virus 2	FJ744715	10678	Human	Thailand
DENV-2/TH/BID-V2302/2001(2)	Dengue virus 2	FJ744716	10678	Human	Thailand
DENV-2/TH/BID-V2303/2001(2)	Dengue virus 2	FJ744717	10678	Human	Thailand
DENV-2/TH/BID-V2304/2001(2)	Dengue virus 2	FJ744718	10678	Human	Thailand
DENV-2/TH/BID-V2305/2001(2)	Dengue virus 2	FJ744719	10719	Human	Thailand
DENV-2/TH/BID-V2306/2001(2)	Dengue virus 2	FJ744720	10678	Human	Thailand
DENV-2/TH/BID-V2307/2001(2)	Dengue virus 2	FJ744721	10679	Human	Thailand
DENV-2/TH/BID-V2308/2001(2)	Dengue virus 2	FJ744722	10679	Human	Thailand
DENV-2/TH/BID-V2309/2001(2)	Dengue virus 2	FJ744723	10671	Human	Thailand
DENV-2/TH/BID-V2310/2001(2)	Dengue virus 2	FJ744724	10659	Human	Thailand
DENV-2/TH/BID-V2311/2001(2)	Dengue virus 2	FJ744725	10678	Human	Thailand
DENV-2/TH/BID-V2616/1996(2)	Dengue virus 2	FJ906957	10678	Human	Thailand
DENV-2/TH/BID-V2617/1996(2)	Dengue virus 2	FJ906958	10679	Human	Thailand
DENV-2/TH/BID-V2957/2003(2)	Dengue virus 2	FJ898452	10679	Human	Thailand
DENV-2/TH/BID-V3357/1964(2)	Dengue virus 2	GQ868591	10678	Human	Thailand
DENV-2/TH/BID-V3498/1994(2)	Dengue virus 2	GQ868542	10678	Human	Thailand
DENV-2/TH/BID-V3499/1996(2)	Dengue virus 2	GQ868544	10681	Human	Thailand
DENV-2/TH/BID-V3500/1996(2)	Dengue virus 2	GQ868545	10679	Human	Thailand
DENV-2/TH/BID-V3501/1995(2)	Dengue virus 2	GQ868543	10678	Human	Thailand
DENV-2/TH/BID-V4325/2001(2)	Dengue virus 2	GU131886	10678	Human	Thailand
DENV-2/TW/BID-V5054/2008	Dengue virus 2	HQ891023	10497	Human	Taiwan
DENV-2/TW/BID-V5056/2008	Dengue virus 2	HQ891024	10615	Mouse	Taiwan
DENV-2/UMASS-Medical/BID-V2619/2008(2)	Dengue virus 2	GQ199899	10570	Mouse	-N/A-
DENV-2/UMASS-Medical/BID-V2620/2008(2)	Dengue virus 2	GQ199900	10538	Mouse	-N/A-
DENV-2/UMASS-Medical/BID-V2621/2008(2)	Dengue virus 2	GQ199901	10569	Mouse	-N/A-
DENV-2/US/BID-V1031/2006(2)	Dengue virus 2	EU482544	10678	Human	USA
DENV-2/US/BID-V1032/1998(2)	Dengue virus 2	EU482545	10678	Human	USA
DENV-2/US/BID-V1033/1998(2)	Dengue virus 2	EU482546	10678	Human	USA
DENV-2/US/BID-V1034/1998(2)	Dengue virus 2	EU482547	10678	Human	USA
DENV-2/US/BID-V1035/2006(2)	Dengue virus 2	EU482548	10620	Human	USA
DENV-2/US/BID-V1036/2006(2)	Dengue virus 2	EU482549	10679	Human	USA
DENV-2/US/BID-V1038/1998(2)	Dengue virus 2	EU482550	10678	Human	USA
DENV-2/US/BID-V1039/2006(2)	Dengue virus 2	EU482551	10678	Human	USA
DENV-2/US/BID-V1040/2006(2)	Dengue virus 2	EU482552	10678	Human	USA
DENV-2/US/BID-V1041/2006(2)	Dengue virus 2	EU482553	10678	Human	USA
DENV-2/US/BID-V1042/1998(2)	Dengue virus 2	EU482554	10639	Human	USA
DENV-2/US/BID-V1045/2005(2)	Dengue virus 2	EU482556	10679	Human	USA
DENV-2/US/BID-V1046/2004(2)	Dengue virus 2	EU529693	10639	Human	USA
DENV-2/US/BID-V1048/1999(2)	Dengue virus 2	EU482557	10639	Human	USA
DENV-2/US/BID-V1052/1998(2)	Dengue virus 2	EU482560	10678	Human	USA
DENV-2/US/BID-V1054/1996(2)	Dengue virus 2	EU482561	10677	Human	USA
DENV-2/US/BID-V1055/1996(2)	Dengue virus 2	EU569703	10663	Human	USA
DENV-2/US/BID-V1057/1994(2)	Dengue virus 2	EU529694	10678	Human	USA
DENV-2/US/BID-V1058/1994(2)	Dengue virus 2	EU529695	10666	Human	USA
DENV-2/US/BID-V1060/1989(2)	Dengue virus 2	EU482562	10680	Human	USA
DENV-2/US/BID-V1061/1989(2)	Dengue virus 2	EU660398	10639	Human	USA
DENV-2/US/BID-V1084/1998(2)	Dengue virus 2	EU482565	10678	Human	USA
DENV-2/US/BID-V1085/1994(2)	Dengue virus 2	EU529700	10678	Human	USA
DENV-2/US/BID-V1087/1991(2)	Dengue virus 2	EU529701	10678	Human	USA
DENV-2/US/BID-V1163/1986(2)	Dengue virus 2	EU569704	10668	Human	USA
DENV-2/US/BID-V1164/1986(2)	Dengue virus 2	EU482568	10678	Human	USA
DENV-2/US/BID-V1165/1987(2)	Dengue virus 2	EU482569	10678	Human	USA
DENV-2/US/BID-V1166/1987(2)	Dengue virus 2	EU482570	10678	Human	USA
DENV-2/US/BID-V1167/1987(2)	Dengue virus 2	EU482571	10678	Human	USA
DENV-2/US/BID-V1168/1987(2)	Dengue virus 2	EU482572	10649	Human	USA
DENV-2/US/BID-V1169/1987(2)	Dengue virus 2	EU482573	10660	Human	USA
DENV-2/US/BID-V1170/1987(2)	Dengue virus 2	EU482574	10678	Human	USA
DENV-2/US/BID-V1171/1987(2)	Dengue virus 2	EU482575	10667	Human	USA
DENV-2/US/BID-V1172/1987(2)	Dengue virus 2	EU482576	10678	Human	USA
DENV-2/US/BID-V1174/1987(2)	Dengue virus 2	EU482577	10678	Human	USA
DENV-2/US/BID-V1175/1988(2)	Dengue virus 2	EU482578	10678	Human	USA
DENV-2/US/BID-V1176/1988(2)	Dengue virus 2	EU482579	10678	Human	USA
DENV-2/US/BID-V1177/1989(2)	Dengue virus 2	EU482580	10678	Human	USA
DENV-2/US/BID-V1178/1989(2)	Dengue virus 2	EU482581	10679	Human	USA
DENV-2/US/BID-V1179/1989(2)	Dengue virus 2	EU482582	10679	Human	USA
DENV-2/US/BID-V1180/1989(2)	Dengue virus 2	EU482583	10678	Human	USA
DENV-2/US/BID-V1181/1989(2)	Dengue virus 2	EU482584	10662	Human	USA
DENV-2/US/BID-V1182/1989(2)	Dengue virus 2	EU482585	10678	Human	USA
DENV-2/US/BID-V1183/1990(2)	Dengue virus 2	EU482586	10678	Human	USA
DENV-2/US/BID-V1188/1990(2)	Dengue virus 2	EU482587	10678	Human	USA
DENV-2/US/BID-V1189/1990(2)	Dengue virus 2	EU482588	10678	Human	USA
DENV-2/US/BID-V1356/1993(2)	Dengue virus 2	EU569705	10663	Human	USA
DENV-2/US/BID-V1360/1993(2)	Dengue virus 2	EU482589	10678	Human	USA
DENV-2/US/BID-V1367/1995(2)	Dengue virus 2	EU569706	10678	Human	USA
DENV-2/US/BID-V1368/1995(2)	Dengue virus 2	EU569707	10678	Human	USA
DENV-2/US/BID-V1372/1995(2)	Dengue virus 2	EU660399	10678	Human	USA
DENV-2/US/BID-V1373/1995(2)	Dengue virus 2	EU569708	10678	Human	USA
DENV-2/US/BID-V1376/1996(2)	Dengue virus 2	EU569709	10678	Human	USA
DENV-2/US/BID-V1378/1996(2)	Dengue virus 2	EU596485	10678	Human	USA
DENV-2/US/BID-V1383/1996(2)	Dengue virus 2	EU569710	10678	Human	USA
DENV-2/US/BID-V1385/1996(2)	Dengue virus 2	EU596486	10678	Human	USA
DENV-2/US/BID-V1387/1998(2)	Dengue virus 2	EU687212	10668	Human	USA
DENV-2/US/BID-V1388/1998(2)	Dengue virus 2	EU660400	10677	Human	USA
DENV-2/US/BID-V1392/1998(2)	Dengue virus 2	EU569711	10678	Human	USA

DENV-2/US/BID-V1393/1998(2)	Dengue virus 2	EU569712	10677	Human	USA
DENV-2/US/BID-V1394/1998(2)	Dengue virus 2	EU596487	10666	Human	USA
DENV-2/US/BID-V1395/1997(2)	Dengue virus 2	EU569713	10677	Human	USA
DENV-2/US/BID-V1396/1997(2)	Dengue virus 2	EU569714	10678	Human	USA
DENV-2/US/BID-V1397/1997(2)	Dengue virus 2	EU569715	10678	Human	USA
DENV-2/US/BID-V1398/1997(2)	Dengue virus 2	EU569716	10678	Human	USA
DENV-2/US/BID-V1399/1997(2)	Dengue virus 2	EU569717	10678	Human	USA
DENV-2/US/BID-V1401/1997(2)	Dengue virus 2	EU569718	10663	Human	USA
DENV-2/US/BID-V1404/1997(2)	Dengue virus 2	EU569719	10677	Human	USA
DENV-2/US/BID-V1409/1997(2)	Dengue virus 2	EU569720	10723	Human	USA
DENV-2/US/BID-V1410/2007(2)	Dengue virus 2	EU596488	10678	Human	USA
DENV-2/US/BID-V1411/2007(2)	Dengue virus 2	EU596489	10678	Human	USA
DENV-2/US/BID-V1412/2007(2)	Dengue virus 2	EU596490	10678	Human	USA
DENV-2/US/BID-V1413/2007(2)	Dengue virus 2	EU596491	10678	Human	USA
DENV-2/US/BID-V1424/1996(2)	Dengue virus 2	EU677141	10677	Human	USA
DENV-2/US/BID-V1425/1999(2)	Dengue virus 2	EU677142	10678	Human	USA
DENV-2/US/BID-V1426/1999(2)	Dengue virus 2	EU677143	10678	Human	USA
DENV-2/US/BID-V1427/1999(2)	Dengue virus 2	EU677144	10678	Human	USA
DENV-2/US/BID-V1428/1999(2)	Dengue virus 2	EU677145	10678	Human	USA
DENV-2/US/BID-V1431/2004(2)	Dengue virus 2	EU677146	10678	Human	USA
DENV-2/US/BID-V1432/2004(2)	Dengue virus 2	EU677147	10678	Human	USA
DENV-2/US/BID-V1434/2004(2)	Dengue virus 2	EU687213	10678	Human	USA
DENV-2/US/BID-V1435/2004(2)	Dengue virus 2	EU687214	10678	Human	USA
DENV-2/US/BID-V1436/2004(2)	Dengue virus 2	EU687215	10678	Human	USA
DENV-2/US/BID-V1439/2005(2)	Dengue virus 2	EU687216	10678	Human	USA
DENV-2/US/BID-V1440/2005(2)	Dengue virus 2	EU726770	10679	Human	USA
DENV-2/US/BID-V1441/2005(2)	Dengue virus 2	EU687217	10678	Human	USA
DENV-2/US/BID-V1442/2005(2)	Dengue virus 2	EU781135	10678	Human	USA
DENV-2/US/BID-V1461/2000(2)	Dengue virus 2	EU687222	10678	Human	USA
DENV-2/US/BID-V1462/2000(2)	Dengue virus 2	EU687223	10678	Human	USA
DENV-2/US/BID-V1463/2000(2)	Dengue virus 2	EU687224	10678	Human	USA
DENV-2/US/BID-V1464/2000(2)	Dengue virus 2	EU687225	10678	Human	USA
DENV-2/US/BID-V1467/2001(2)	Dengue virus 2	EU687227	10678	Human	USA
DENV-2/US/BID-V1468/2001(2)	Dengue virus 2	EU687228	10678	Human	USA
DENV-2/US/BID-V1469/2001(2)	Dengue virus 2	EU687229	10678	Human	USA
DENV-2/US/BID-V1470/2001(2)	Dengue virus 2	EU687230	10674	Human	USA
DENV-2/US/BID-V1471/2001(2)	Dengue virus 2	EU687231	10678	Human	USA
DENV-2/US/BID-V1472/2001(2)	Dengue virus 2	EU687232	10662	Human	USA
DENV-2/US/BID-V1482/2003(2)	Dengue virus 2	EU687199	10678	Human	USA
DENV-2/US/BID-V1483/2003(2)	Dengue virus 2	EU687235	10677	Human	USA
DENV-2/US/BID-V1484/2003(2)	Dengue virus 2	EU687236	10678	Human	USA
DENV-2/US/BID-V1486/2003(2)	Dengue virus 2	EU687237	10678	Human	USA
DENV-2/US/BID-V1487/2003(2)	Dengue virus 2	EU687238	10678	Human	USA
DENV-2/US/BID-V1492/2003(2)	Dengue virus 2	EU687240	10678	Human	USA
DENV-2/US/BID-V1493/2003(2)	Dengue virus 2	EU687241	10678	Human	USA
DENV-2/US/BID-V1494/2004(2)	Dengue virus 2	EU687242	10678	Human	USA
DENV-2/US/BID-V1495/2004(2)	Dengue virus 2	EU687243	10678	Human	USA
DENV-2/US/BID-V1496/2004(2)	Dengue virus 2	EU687244	10678	Human	USA
DENV-2/US/BID-V1497/2005(2)	Dengue virus 2	EU687245	10678	Human	USA
DENV-2/US/BID-V4824/2009	Dengue virus 2	HQ541798	10507	Human	USA
DENV-2/US/BID-V4825/2010	Dengue virus 2	HQ541799	10508	Mouse	USA
DENV-2/US/BID-V5055/2008	Dengue virus 2	JN796245	10488	Mouse	USA
DENV-2/US/BID-V5411/2006	Dengue virus 2	JF730053	10476	Human	USA
DENV-2/US/BID-V5412/2007	Dengue virus 2	JF730050	10484	Human	USA
DENV-2/US/BID-V5413/2009	Dengue virus 2	JF730054	10696	Mouse	USA
DENV-2/US/BID-V5414/2009	Dengue virus 2	JF730055	10659	Mouse	USA
DENV-2/US/BID-V585/2006(2)	Dengue virus 2	EU529706	10668	Human	USA
DENV-2/US/BID-V587/2006(2)	Dengue virus 2	EU482719	10678	Human	USA
DENV-2/US/BID-V588/2006(2)	Dengue virus 2	EU482720	10678	Human	USA
DENV-2/US/BID-V589/2006(2)	Dengue virus 2	EU482721	10678	Human	USA
DENV-2/US/BID-V591/2002(2)	Dengue virus 2	EU482722	10668	Human	USA
DENV-2/US/BID-V592/2002(2)	Dengue virus 2	EU482723	10678	Human	USA
DENV-2/US/BID-V593/2005(2)	Dengue virus 2	EU482724	10678	Human	USA
DENV-2/US/BID-V594/2006(2)	Dengue virus 2	EU482725	10678	Human	USA
DENV-2/US/BID-V595/2006(2)	Dengue virus 2	EU482726	10678	Human	USA
DENV-2/US/BID-V596/1998(2)	Dengue virus 2	EU482727	10678	Human	USA
DENV-2/US/BID-V597/1998(2)	Dengue virus 2	EU482728	10675	Human	USA
DENV-2/US/BID-V598/1999(2)	Dengue virus 2	EU482729	10678	Human	USA
DENV-2/US/BID-V599/1999(2)	Dengue virus 2	EU482730	10629	Human	USA
DENV-2/US/BID-V600/2005(2)	Dengue virus 2	EU482731	10678	Human	USA
DENV-2/US/BID-V675/1998(2)	Dengue virus 2	EU482732	10678	Human	USA
DENV-2/US/BID-V676/1998(2)	Dengue virus 2	EU482733	10676	Human	USA
DENV-2/US/BID-V677/1998(2)	Dengue virus 2	EU482734	10678	Human	USA
DENV-2/US/BID-V678/1998(2)	Dengue virus 2	EU482735	10678	Human	USA
DENV-2/US/BID-V679/1994(2)	Dengue virus 2	EU482736	10678	Human	USA
DENV-2/US/BID-V680/1994(2)	Dengue virus 2	EU482737	10678	Human	USA
DENV-2/US/BID-V681/1998(2)	Dengue virus 2	EU482738	10678	Human	USA
DENV-2/US/BID-V682/1994(2)	Dengue virus 2	EU482739	10678	Human	USA
DENV-2/US/BID-V683/1994(2)	Dengue virus 2	EU482740	10678	Human	USA
DENV-2/US/BID-V684/1994(2)	Dengue virus 2	EU482741	10678	Human	USA
DENV-2/US/BID-V685/1988(2)	Dengue virus 2	EU482742	10678	Human	USA
DENV-2/US/BID-V686/1989(2)	Dengue virus 2	EU482743	10679	Human	USA
DENV-2/US/BID-V687/1989(2)	Dengue virus 2	EU482744	10677	Human	USA
DENV-2/US/BID-V688/1989(2)	Dengue virus 2	EU482745	10678	Human	USA
DENV-2/US/BID-V689/1989(2)	Dengue virus 2	EU482746	10678	Human	USA
DENV-2/US/BID-V690/1988(2)	Dengue virus 2	EU482747	10678	Human	USA
DENV-2/US/BID-V851/1990(2)	Dengue virus 2	EU482590	10678	Human	USA
DENV-2/US/BID-V854/2001(2)	Dengue virus 2	EU482593	10678	Human	USA
DENV-2/US/BID-V855/1992(2)	Dengue virus 2	EU482594	10678	Human	USA
DENV-2/VE/BID-V1095/2007(2)	Dengue virus 2	EU482604	10678	Human	Venezuela
DENV-2/VE/BID-V1104/2007(2)	Dengue virus 2	EU482605	10679	Human	Venezuela
DENV-2/VE/BID-V1105/2007(2)	Dengue virus 2	EU482606	10668	Human	Venezuela
DENV-2/VE/BID-V1107/2007(2)	Dengue virus 2	EU482607	10679	Human	Venezuela
DENV-2/VE/BID-V1111/2007(2)	Dengue virus 2	EU482608	10662	Human	Venezuela
DENV-2/VE/BID-V1142/39083.5	Dengue virus 2	KF955365	10780	Human	Venezuela
DENV-2/VE/BID-V1144/2007(2)	Dengue virus 2	GQ868641	10678	Human	Venezuela
DENV-2/VE/BID-V1456/1996(2)	Dengue virus 2	EU687220	10678	Human	Venezuela
DENV-2/VE/BID-V1457/1996(2)	Dengue virus 2	EU726775	10679	Human	Venezuela
DENV-2/VE/BID-V2158/2005(2)	Dengue virus 2	FJ639732	10630	Human	Venezuela
DENV-2/VE/BID-V2159/2005(2)	Dengue virus 2	FJ639733	10670	Human	Venezuela
DENV-2/VE/BID-V2160/2003(2)	Dengue virus 2	FJ639734	10647	Human	Venezuela
DENV-2/VE/BID-V2161/2001	Dengue virus 2	JN819408	10620	Human	Venezuela
DENV-2/VE/BID-V2216/2003(2)	Dengue virus 2	FJ639783	10679	Human	Venezuela
DENV-2/VE/BID-V2221/2004(2)	Dengue virus 2	FJ639788	10678	Human	Venezuela
DENV-2/VE/BID-V2246/2005(2)	Dengue virus 2	FJ639809	10678	Human	Venezuela
DENV-2/VE/BID-V2262/2006(2)	Dengue virus 2	FJ639822	10679	Human	Venezuela
DENV-2/VE/BID-V2424/2004(2)	Dengue virus 2	FJ850112	10702	Human	Venezuela
DENV-2/VE/BID-V2470/2007(2)	Dengue virus 2	FJ850105	10678	Human	Venezuela
DENV-2/VE/BID-V2476/2008(2)	Dengue virus 2	FJ850106	10677	Human	Venezuela
DENV-2/VE/BID-V2477/2008(2)	Dengue virus 2	FJ850107	10667	Human	Venezuela
DENV-2/VE/BID-V2478/2008(2)	Dengue virus 2	FJ850108	10664	Human	Venezuela
DENV-2/VE/BID-V2613/2007	Dengue virus 2	JN819407	10651	Human	Venezuela
DENV-2/VE/BID-V2941/1998(2)	Dengue virus 2	FJ898465	10677	Human	Venezuela

DENV-2/VE/BID-V2942/2000(2)	Dengue virus 2	FJ898466	10679	Human	Venezuela
DENV-2/VE/BID-V2944/2005(2)	Dengue virus 2	FJ898467	10678	Human	Venezuela
DENV-2/VE/BID-V3362/1991(2)	Dengue virus 2	GQ868595	10678	Human	Venezuela
DENV-2/VE/BID-V3363/1991(2)	Dengue virus 2	GQ868596	10667	Human	Venezuela
DENV-2/VE/BID-V3364/1991(2)	Dengue virus 2	GQ868597	10678	Human	Venezuela
DENV-2/VE/BID-V3365/1991(2)	Dengue virus 2	GQ868598	10678	Human	Venezuela
DENV-2/VE/BID-V3366/1987(2)	Dengue virus 2	GQ868599	10668	Human	Venezuela
DENV-2/VE/BID-V3496/1990(2)	Dengue virus 2	GQ868540	10678	Human	Venezuela
DENV-2/VE/BID-V3497/1991(2)	Dengue virus 2	GQ868541	10678	Human	Venezuela
DENV-2/VI/BID-V2946/1987(2)	Dengue virus 2	GQ868603	10666	Human	Virgin Islands
DENV-2/VI/BID-V2948/1990(2)	Dengue virus 2	FJ898450	10678	Human	Virgin Islands
DENV-2/VI/BID-V2960/2005(2)	Dengue virus 2	FJ898453	10678	Human	Virgin Islands
DENV-2/VN/BID-V1000/2006(2)	Dengue virus 2	EU482445	10669	Human	Viet Nam
DENV-2/VN/BID-V1001/2006(2)	Dengue virus 2	EU482446	10678	Human	Viet Nam
DENV-2/VN/BID-V1002/2006(2)	Dengue virus 2	EU482447	10678	Human	Viet Nam
DENV-2/VN/BID-V1003/2006(2)	Dengue virus 2	EU482448	10678	Human	Viet Nam
DENV-2/VN/BID-V1004/2006(2)	Dengue virus 2	EU482449	10678	Human	Viet Nam
DENV-2/VN/BID-V1005/2006(2)	Dengue virus 2	EU482450	10678	Human	Viet Nam
DENV-2/VN/BID-V1006/2006(2)	Dengue virus 2	EU569721	10678	Human	Viet Nam
DENV-2/VN/BID-V1007/2006(2)	Dengue virus 2	EU482451	10678	Human	Viet Nam
DENV-2/VN/BID-V1507/2007(2)	Dengue virus 2	EU660413	10678	Human	Viet Nam
DENV-2/VN/BID-V1508/2007(2)	Dengue virus 2	EU660414	10678	Human	Viet Nam
DENV-2/VN/BID-V1509/2007(2)	Dengue virus 2	EU687248	10678	Human	Viet Nam
DENV-2/VN/BID-V1511/2007(2)	Dengue virus 2	EU660415	10711	Human	Viet Nam
DENV-2/VN/BID-V1512/2007(2)	Dengue virus 2	EU687249	10678	Human	Viet Nam
DENV-2/VN/BID-V1513/2007(2)	Dengue virus 2	EU660416	10678	Human	Viet Nam
DENV-2/VN/BID-V1514/2007(2)	Dengue virus 2	EU726776	10663	Human	Viet Nam
DENV-2/VN/BID-V1515/2007(2)	Dengue virus 2	EU660417	10678	Human	Viet Nam
DENV-2/VN/BID-V1517/2007(2)	Dengue virus 2	EU677137	10661	Human	Viet Nam
DENV-2/VN/BID-V1518/2007(2)	Dengue virus 2	EU677138	10678	Human	Viet Nam
DENV-2/VN/BID-V1519/2007(2)	Dengue virus 2	EU677148	10678	Human	Viet Nam
DENV-2/VN/BID-V1520/2007(2)	Dengue virus 2	EU677149	10661	Human	Viet Nam
DENV-2/VN/BID-V1522/2007(2)	Dengue virus 2	EU687250	10668	Human	Viet Nam
DENV-2/VN/BID-V1654/2007(2)	Dengue virus 2	FJ410288	10678	Human	Viet Nam
DENV-2/VN/BID-V1669/2007(2)	Dengue virus 2	FJ024452	10678	Human	Viet Nam
DENV-2/VN/BID-V1674/2007(2)	Dengue virus 2	FJ205877	10677	Human	Viet Nam
DENV-2/VN/BID-V1675/2007(2)	Dengue virus 2	FJ024454	10678	Human	Viet Nam
DENV-2/VN/BID-V1679/2007(2)	Dengue virus 2	FJ390384	10678	Human	Viet Nam
DENV-2/VN/BID-V1680/2007(2)	Dengue virus 2	FJ205878	10678	Human	Viet Nam
DENV-2/VN/BID-V1682/2007(2)	Dengue virus 2	FJ205879	10678	Human	Viet Nam
DENV-2/VN/BID-V1684/2007(2)	Dengue virus 2	FJ390385	10678	Human	Viet Nam
DENV-2/VN/BID-V1685/2007(2)	Dengue virus 2	FJ024458	10678	Human	Viet Nam
DENV-2/VN/BID-V1690/2007(2)	Dengue virus 2	FJ205880	10678	Human	Viet Nam
DENV-2/VN/BID-V1696/2007(2)	Dengue virus 2	FJ390387	10668	Human	Viet Nam
DENV-2/VN/BID-V1697/2007(2)	Dengue virus 2	FJ024461	10678	Human	Viet Nam
DENV-2/VN/BID-V1699/2007(2)	Dengue virus 2	FJ373299	10678	Human	Viet Nam
DENV-2/VN/BID-V1773/2007(2)	Dengue virus 2	FJ410193	10666	Human	Viet Nam
DENV-2/VN/BID-V1776/2007(2)	Dengue virus 2	FJ410195	10678	Human	Viet Nam
DENV-2/VN/BID-V1777/2007(2)	Dengue virus 2	FJ432724	10663	Human	Viet Nam
DENV-2/VN/BID-V1780/2007(2)	Dengue virus 2	FJ432726	10678	Human	Viet Nam
DENV-2/VN/BID-V1781/2007-2	Dengue virus 2	JF730049	10308	Human	Viet Nam
DENV-2/VN/BID-V1794/2007(2)	Dengue virus 2	FJ562098	10665	Human	Viet Nam
DENV-2/VN/BID-V1796/2007(2)	Dengue virus 2	FJ859028	10663	Human	Viet Nam
DENV-2/VN/BID-V1801/2007(2)	Dengue virus 2	FJ410200	10673	Human	Viet Nam
DENV-2/VN/BID-V1807/2007(2)	Dengue virus 2	FJ410202	10668	Human	Viet Nam
DENV-2/VN/BID-V1818/2007(2)	Dengue virus 2	FJ410208	10674	Human	Viet Nam
DENV-2/VN/BID-V1837/2007(2)	Dengue virus 2	FJ461305	10664	Human	Viet Nam
DENV-2/VN/BID-V1844/2008(2)	Dengue virus 2	FJ410215	10678	Human	Viet Nam
DENV-2/VN/BID-V1845/2008(2)	Dengue virus 2	FJ461309	10678	Human	Viet Nam
DENV-2/VN/BID-V1848/2008(2)	Dengue virus 2	FJ461311	10678	Human	Viet Nam
DENV-2/VN/BID-V1853/2007(2)	Dengue virus 2	FJ410217	10678	Human	Viet Nam
DENV-2/VN/BID-V1857/2007(2)	Dengue virus 2	FJ410219	10668	Human	Viet Nam
DENV-2/VN/BID-V1858/2007(2)	Dengue virus 2	FJ461314	10678	Human	Viet Nam
DENV-2/VN/BID-V1864/2007(2)	Dengue virus 2	FJ410221	10678	Human	Viet Nam
DENV-2/VN/BID-V1868/2007(2)	Dengue virus 2	FJ547064	10679	Human	Viet Nam
DENV-2/VN/BID-V1869/2007(2)	Dengue virus 2	FJ410223	10677	Human	Viet Nam
DENV-2/VN/BID-V1872/2007(2)	Dengue virus 2	FJ410224	10678	Human	Viet Nam
DENV-2/VN/BID-V1873/2007(2)	Dengue virus 2	FJ461321	10694	Human	Viet Nam
DENV-2/VN/BID-V1881/2007(2)	Dengue virus 2	FJ410228	10678	Human	Viet Nam
DENV-2/VN/BID-V1895/2008(2)	Dengue virus 2	FJ410233	10663	Human	Viet Nam
DENV-2/VN/BID-V1900/2008(2)	Dengue virus 2	FJ410237	10678	Human	Viet Nam
DENV-2/VN/BID-V1905/2008(2)	Dengue virus 2	FJ410241	10678	Human	Viet Nam
DENV-2/VN/BID-V1928/2008(2)	Dengue virus 2	FJ410259	10678	Human	Viet Nam
DENV-2/VN/BID-V1930/2007(2)	Dengue virus 2	FJ547067	10675	Human	Viet Nam
DENV-2/VN/BID-V2947/1988	Dengue virus 2	JN819418	10576	Human	Viet Nam
DENV-2/VN/BID-V703/2006(2)	Dengue virus 2	EU482640	10676	Human	Viet Nam
DENV-2/VN/BID-V704/2006(2)	Dengue virus 2	EU482641	10677	Human	Viet Nam
DENV-2/VN/BID-V705/2006(2)	Dengue virus 2	EU482642	10678	Human	Viet Nam
DENV-2/VN/BID-V706/2006(2)	Dengue virus 2	EU482643	10678	Human	Viet Nam
DENV-2/VN/BID-V707/2006(2)	Dengue virus 2	EU482644	10678	Human	Viet Nam
DENV-2/VN/BID-V708/2006(2)	Dengue virus 2	EU482645	10678	Human	Viet Nam
DENV-2/VN/BID-V709/2006(2)	Dengue virus 2	EU482646	10660	Human	Viet Nam
DENV-2/VN/BID-V710/2006(2)	Dengue virus 2	EU482647	10678	Human	Viet Nam
DENV-2/VN/BID-V711/2006(2)	Dengue virus 2	EU482648	10678	Human	Viet Nam
DENV-2/VN/BID-V712/2006(2)	Dengue virus 2	EU482649	10678	Human	Viet Nam
DENV-2/VN/BID-V713/2006(2)	Dengue virus 2	EU482650	10678	Human	Viet Nam
DENV-2/VN/BID-V714/2006(2)	Dengue virus 2	EU482651	10678	Human	Viet Nam
DENV-2/VN/BID-V715/2006(2)	Dengue virus 2	EU482652	10678	Human	Viet Nam
DENV-2/VN/BID-V716/2006(2)	Dengue virus 2	EU482653	10678	Human	Viet Nam
DENV-2/VN/BID-V717/2006(2)	Dengue virus 2	EU482654	10678	Human	Viet Nam
DENV-2/VN/BID-V718/2006(2)	Dengue virus 2	EU482655	10678	Human	Viet Nam
DENV-2/VN/BID-V719/2006(2)	Dengue virus 2	EU482656	10673	Human	Viet Nam
DENV-2/VN/BID-V720/2006(2)	Dengue virus 2	EU482657	10678	Human	Viet Nam
DENV-2/VN/BID-V721/2006(2)	Dengue virus 2	EU482658	10678	Human	Viet Nam
DENV-2/VN/BID-V722/2006(2)	Dengue virus 2	EU482659	10678	Human	Viet Nam
DENV-2/VN/BID-V723/2006(2)	Dengue virus 2	EU482660	10666	Human	Viet Nam
DENV-2/VN/BID-V724/2006(2)	Dengue virus 2	EU482661	10678	Human	Viet Nam
DENV-2/VN/BID-V725/2006(2)	Dengue virus 2	EU482662	10669	Human	Viet Nam
DENV-2/VN/BID-V726/2006(2)	Dengue virus 2	EU482663	10678	Human	Viet Nam
DENV-2/VN/BID-V727/2006(2)	Dengue virus 2	EU482664	10678	Human	Viet Nam
DENV-2/VN/BID-V728/2006(2)	Dengue virus 2	EU482665	10671	Human	Viet Nam
DENV-2/VN/BID-V729/2006(2)	Dengue virus 2	EU482666	10678	Human	Viet Nam
DENV-2/VN/BID-V730/2006(2)	Dengue virus 2	EU482667	10661	Human	Viet Nam
DENV-2/VN/BID-V731/2006(2)	Dengue virus 2	EU482668	10678	Human	Viet Nam
DENV-2/VN/BID-V732/2006(2)	Dengue virus 2	EU482669	10665	Human	Viet Nam
DENV-2/VN/BID-V733/2006(2)	Dengue virus 2	EU482670	10678	Human	Viet Nam
DENV-2/VN/BID-V734/2006(2)	Dengue virus 2	EU482671	10660	Human	Viet Nam
DENV-2/VN/BID-V735/2006(2)	Dengue virus 2	EU482672	10678	Human	Viet Nam
DENV-2/VN/BID-V736/2006(2)	Dengue virus 2	EU482673	10677	Human	Viet Nam
DENV-2/VN/BID-V737/2006(2)	Dengue virus 2	EU482674	10678	Human	Viet Nam
DENV-2/VN/BID-V738/2006(2)	Dengue virus 2	EU482675	10678	Human	Viet Nam
DENV-2/VN/BID-V739/2006(2)	Dengue virus 2	EU482676	10678	Human	Viet Nam
DENV-2/VN/BID-V740/2006(2)	Dengue virus 2	EU482677	10678	Human	Viet Nam

DENV-2/VN/BID-V741/2006(2)	Dengue virus 2	EU482678	10678	Human	Viet Nam
DENV-2/VN/BID-V742/2007(2)	Dengue virus 2	EU482679	10678	Human	Viet Nam
DENV-2/VN/BID-V752/2004(2)	Dengue virus 2	EU482774	10678	Human	Viet Nam
DENV-2/VN/BID-V753/2004(2)	Dengue virus 2	EU482775	10678	Human	Viet Nam
DENV-2/VN/BID-V754/2005(2)	Dengue virus 2	EU482776	10678	Human	Viet Nam
DENV-2/VN/BID-V755/2005(2)	Dengue virus 2	EU482777	10678	Human	Viet Nam
DENV-2/VN/BID-V756/2003(2)	Dengue virus 2	EU482778	10677	Human	Viet Nam
DENV-2/VN/BID-V757/2003(2)	Dengue virus 2	EU482779	10678	Human	Viet Nam
DENV-2/VN/BID-V758/2003(2)	Dengue virus 2	EU482780	10678	Human	Viet Nam
DENV-2/VN/BID-V759/2003(2)	Dengue virus 2	EU482781	10678	Human	Viet Nam
DENV-2/VN/BID-V760/2003(2)	Dengue virus 2	EU482782	10678	Human	Viet Nam
DENV-2/VN/BID-V761/2003(2)	Dengue virus 2	EU482783	10677	Human	Viet Nam
DENV-2/VN/BID-V762/2003(2)	Dengue virus 2	EU482784	10679	Human	Viet Nam
DENV-2/VN/BID-V763/2003(2)	Dengue virus 2	EU482785	10678	Human	Viet Nam
DENV-2/VN/BID-V764/2003(2)	Dengue virus 2	EU482786	10678	Human	Viet Nam
DENV-2/VN/BID-V765/2003(2)	Dengue virus 2	EU482787	10678	Human	Viet Nam
DENV-2/VN/BID-V766/2003(2)	Dengue virus 2	EU482788	10678	Human	Viet Nam
DENV-2/VN/BID-V771/2007(2)	Dengue virus 2	EU482697	10678	Human	Viet Nam
DENV-2/VN/BID-V772/2007(2)	Dengue virus 2	EU482698	10678	Human	Viet Nam
DENV-2/VN/BID-V773/2007(2)	Dengue virus 2	EU482699	10678	Human	Viet Nam
DENV-2/VN/BID-V774/2007(2)	Dengue virus 2	EU482700	10678	Human	Viet Nam
DENV-2/VN/BID-V775/2007(2)	Dengue virus 2	EU482701	10678	Human	Viet Nam
DENV-2/VN/BID-V776/2007(2)	Dengue virus 2	EU482702	10678	Human	Viet Nam
DENV-2/VN/BID-V777/2007(2)	Dengue virus 2	EU482703	10678	Human	Viet Nam
DENV-2/VN/BID-V778/2007(2)	Dengue virus 2	EU482704	10678	Human	Viet Nam
DENV-2/VN/BID-V779/2007(2)	Dengue virus 2	EU482705	10678	Human	Viet Nam
DENV-2/VN/BID-V917/2006(2)	Dengue virus 2	EU482463	10678	Human	Viet Nam
DENV-2/VN/BID-V918/2006(2)	Dengue virus 2	EU482464	10678	Human	Viet Nam
DENV-2/VN/BID-V919/2006(2)	Dengue virus 2	EU482465	10678	Human	Viet Nam
DENV-2/VN/BID-V920/2006(2)	Dengue virus 2	EU482466	10678	Human	Viet Nam
DENV-2/VN/BID-V921/2006(2)	Dengue virus 2	EU482467	10678	Human	Viet Nam
DENV-2/VN/BID-V922/2006(2)	Dengue virus 2	FJ873811	10678	Human	Viet Nam
DENV-2/VN/BID-V923/2006(2)	Dengue virus 2	EU482468	10678	Human	Viet Nam
DENV-2/VN/BID-V924/2006(2)	Dengue virus 2	EU482469	10668	Human	Viet Nam
DENV-2/VN/BID-V925/2006(2)	Dengue virus 2	EU482470	10678	Human	Viet Nam
DENV-2/VN/BID-V926/2006(2)	Dengue virus 2	EU482471	10678	Human	Viet Nam
DENV-2/VN/BID-V927/2004(2)	Dengue virus 2	EU482472	10678	Human	Viet Nam
DENV-2/VN/BID-V928/2006(2)	Dengue virus 2	EU482473	10678	Human	Viet Nam
DENV-2/VN/BID-V929/2007(2)	Dengue virus 2	EU482474	10678	Human	Viet Nam
DENV-2/VN/BID-V930/2007(2)	Dengue virus 2	EU482475	10678	Human	Viet Nam
DENV-2/VN/BID-V997/2006(2)	Dengue virus 2	EU482541	10678	Human	Viet Nam
DENV-2/VN/BID-V998/2006(2)	Dengue virus 2	EU482542	10678	Human	Viet Nam
DENV-2/VN/BID-V999/2006(2)	Dengue virus 2	EU482543	10668	Human	Viet Nam
DENV2-PR159	Dengue virus 2	JX966380	10712	Human	Puerto Rico
DENV2-QR94	Dengue virus 2	JX966379	10713	Human	Mexico
DENV2/CN/GZ05/2014-2	Dengue virus 2	KP012546	10723	Human	China
DENV2/CN/GZ1118/2014-2	Dengue virus 2	KT187558	10723	Human	China
DENV2/CN/GZ25/2014-2	Dengue virus 2	KP723478	10583	Human	China
DENV2/CN/GZ32/2010-2	Dengue virus 2	KP723479	10723	Human	China
DENV2/CN/GZDF31241/2014-2	Dengue virus 2	KT187553	10723	Human	China
DENV2/CN/GZDF571/2014-2	Dengue virus 2	KT187554	10723	Human	China
DENV2/CN/GZDF574/2014-2	Dengue virus 2	KT187555	10723	Human	China
DENV2/CN/GZDF595/2014-2	Dengue virus 2	KT187556	10723	Human	China
DENV2/CN/GZDF615/2014-2	Dengue virus 2	KT187557	10723	Human	China
DENV2/Vietnam/10dx-117-801-1500mg-0hrs-2	Dengue virus 2	JQ045669	10685	Human	Viet Nam
DENV2/Vietnam/10dx-206-801-Placebo-0hrs-2	Dengue virus 2	JQ045670	10702	Human	Viet Nam
DENV2/Vietnam/10dx-207-801-3000mg-36hrs-2	Dengue virus 2	JQ045671	10691	Human	Viet Nam
DENV2/Vietnam/10dx-207-802-3000mg-36hrs-2	Dengue virus 2	JQ045672	10631	Human	Viet Nam
DENV2/Vietnam/10dx-211-801-3000mg-12hrs-2	Dengue virus 2	JQ045673	10559	Human	Viet Nam
DENV2/Vietnam/10dx-211-802-3000mg-12hrs-2	Dengue virus 2	JQ045674	10700	Human	Viet Nam
DENV2/Vietnam/10dx-217-801-3000mg-48hrs-2	Dengue virus 2	JQ045675	10691	Human	Viet Nam
DENV2/Vietnam/10dx-217-802-3000mg-48hrs-2	Dengue virus 2	JQ045676	10684	Human	Viet Nam
DENV2/Vietnam/10dx-220-801-Placebo-0hrs-2	Dengue virus 2	JQ045677	10617	Human	Viet Nam
DENV2/Vietnam/10dx-224-801-3000mg-24hrs-2	Dengue virus 2	JQ045678	10690	Human	Viet Nam
DENV2/Vietnam/10dx-224-802-3000mg-24hrs-2	Dengue virus 2	JQ045679	10691	Human	Viet Nam
DENV2/Vietnam/10dx-230-801-3000mg-0hrs-2	Dengue virus 2	JQ045680	10688	Human	Viet Nam
DENV2/Vietnam/10dx-233-801-Placebo-60hrs-2	Dengue virus 2	JQ045681	10691	Human	Viet Nam
DENV2/Vietnam/10dx-233-802-Placebo-60hrs-2	Dengue virus 2	JQ045682	10698	Human	Viet Nam
DENV2/Vietnam/10dx-234-801-3000mg-36hrs-2	Dengue virus 2	JQ045683	10688	Human	Viet Nam
DENV2/Vietnam/10dx-234-802-3000mg-36hrs-2	Dengue virus 2	JQ045684	10702	Human	Viet Nam
DENV2/Vietnam/10dx-236-801-Placebo-24hrs-2	Dengue virus 2	JQ045685	10691	Human	Viet Nam
DENV2/Vietnam/10dx-236-802-Placebo-24hrs-2	Dengue virus 2	JQ045686	10702	Human	Viet Nam
DF380(2)	Dengue virus 2	FM210215	10685	Human	Viet Nam
DF401(2)	Dengue virus 2	FM210216	10685	Human	Viet Nam
DF404(2)	Dengue virus 2	FM210217	10685	Human	Viet Nam
DF593(2)	Dengue virus 2	FM210218	10385	Unknown	Viet Nam
DF657(2)	Dengue virus 2	FM210237	10684	Unknown	Viet Nam
DF670(2)	Dengue virus 2	FM210219	10685	Unknown	Viet Nam
DF674(2)	Dengue virus 2	FM210220	10616	Unknown	Viet Nam
DF699(2)	Dengue virus 2	FM210221	10685	Unknown	Viet Nam
DF707(2)	Dengue virus 2	FM210208	10595	Unknown	Viet Nam
DF726(2)	Dengue virus 2	FM210209	10692	Unknown	Viet Nam
DF727(2)	Dengue virus 2	FM210210	10693	Unknown	Viet Nam
DF755(2)	Dengue virus 2	FM210222	10685	Unknown	Viet Nam
DF768(2)	Dengue virus 2	FM210202	10692	Unknown	Viet Nam
DF897(2)	Dengue virus 2	FM210223	10687	Unknown	Viet Nam
DF900(2)	Dengue virus 2	FM210203	10690	Unknown	Viet Nam
DF907(2)	Dengue virus 2	FM210211	10691	Unknown	Viet Nam
DGV106-2	Dengue virus 2	JX286524	10587	Human	Brazil
DGV34-2	Dengue virus 2	JX286522	10599	Human	Brazil
DGV37-2	Dengue virus 2	JX286520	10598	Human	Brazil
DGV69-2	Dengue virus 2	JX286525	10596	Human	Brazil
DGV91-2	Dengue virus 2	JX286523	10611	Human	Brazil
DKD811(2)	Dengue virus 2	FJ467493	10719	Unknown	Malaysia
DR23/01(2)	Dengue virus 2	AB122020	10723	Human	Dominican Republic
DR31/01(2)	Dengue virus 2	AB122021	10723	Human	Dominican Republic
DR59/01(2)	Dengue virus 2	AB122022	10723	Human	Dominican Republic
DS04-221205(2)	Dengue virus 2	EU179858	10709	Unknown	Brunei
DS09-280106(2)	Dengue virus 2	EU179859	10709	Unknown	Brunei
DS31-291005(2)	Dengue virus 2	EU179857	10709	Unknown	Brunei
E1379Y12-2	Dengue virus 2	KM279598	10723	Human	Singapore
E1429Y12-2	Dengue virus 2	KM279599	10723	Human	Singapore
E1432Y12-2	Dengue virus 2	KM279600	10723	Human	Singapore
E1433Y12-2	Dengue virus 2	KM279576	10723	Human	Singapore
E1434Y12-2	Dengue virus 2	KM279577	10723	Human	Singapore
E1436Y12-2	Dengue virus 2	KM279578	10723	Human	Singapore
E1439Y12-2	Dengue virus 2	KM279601	10723	Human	Singapore
E1502Y12-2	Dengue virus 2	KM279579	10723	Human	Singapore
FGU-Apr-02-DENV-2	Dengue virus 2	EU920835	10724	Unknown	French Guiana
FGU-Apr1-06-DENV-2	Dengue virus 2	EU920847	10644	Unknown	French Guiana
FGU-Apr2-06-DENV-2	Dengue virus 2	EU920848	10724	Unknown	French Guiana
FGU-Aug-98-DENV-2	Dengue virus 2	EU920831	10694	Unknown	French Guiana
FGU-Dec-01-DENV-2	Dengue virus 2	EU920834	10676	Unknown	French Guiana

FGU-Feb-93-DENV-2	Dengue virus 2	EU920828	10663	Unknown	French Guiana
FGU-Fev-98-DENV-2	Dengue virus 2	EU920830	10717	Unknown	French Guiana
FGU-Jan-00-DENV-2	Dengue virus 2	EU920833	10649	Unknown	French Guiana
FGU-Jan1-06-DENV-2	Dengue virus 2	EU920844	10724	Unknown	French Guiana
FGU-Jan2-06-DENV-2	Dengue virus 2	EU920845	10666	Unknown	French Guiana
FGU-Jun-97-DENV-2	Dengue virus 2	EU920829	10724	Unknown	French Guiana
FGU-Mar-06-DENV-2	Dengue virus 2	EU920846	10687	Unknown	French Guiana
FGU-May-99-DENV-2	Dengue virus 2	EU920832	10698	Unknown	French Guiana
FGU-Oct-05-DENV-2	Dengue virus 2	EU920840	10688	Unknown	French Guiana
FJ-10(2)	Dengue virus 2	AF276619	10723	Unknown	China
FJ11/99(2)	Dengue virus 2	AF359579	10723	Unknown	-N/A-
GD01/01	Dengue virus 2	FJ196852	10723	Unknown	China
GD01/03	Dengue virus 2	FJ196853	10723	Unknown	China
GD05/98	Dengue virus 2	KC964095	10723	Human	China
GD06/93	Dengue virus 2	FJ196854	10723	Unknown	China
GD08/98	Dengue virus 2	FJ196851	10723	Unknown	China
GD09/93	Dengue virus 2	KC964094	10723	Human	China
GD19/2001	Dengue virus 2	KC964093	10723	Human	China
GUA-Oct-06-DENV-2	Dengue virus 2	EU920850	10613	Unknown	Guadeloupe
GUA-Sep-06-DENV-2	Dengue virus 2	EU920849	10690	Unknown	Guadeloupe
GW6	Dengue virus 2	KM587709	10713	Unknown	USA
GWL18 INDI-01(2)	Dengue virus 2	DQ448231	10670	Human	India
GZ40-2	Dengue virus 2	JX470186	10723	Human	China
I348600(2)	Dengue virus 2	AY702040	10675	Unknown	Colombia
IBH11208-2	Dengue virus 2	EF105387	10709	Unknown	Nigeria
IBH11234(2)	Dengue virus 2	EU003591	10501	Unknown	Nigeria
IBH11664(2)	Dengue virus 2	EF105388	10711	Unknown	Nigeria
IQT-1950(2)	Dengue virus 2	EU056811	10713	Human	Peru
IQT1797(2)	Dengue virus 2	AF100467	10674	Unknown	-N/A-
IQT2913-2	Dengue virus 2	AF100468	10674	Unknown	-N/A-
Jamaica/N.1409(2)	Dengue virus 2	M20558	10723	Unknown	-N/A-
Jeddah-2014	Dengue virus 2	KJ830750	10718	Human	Saudi Arabia
K0008(2)	Dengue virus 2	AF100459	10685	Unknown	-N/A-
K0010(2)	Dengue virus 2	AF100460	10685	Unknown	-N/A-
MAR-Aug-05-DENV-2	Dengue virus 2	EU920837	10717	Unknown	Martinique
MAR-Jun-05-DENV-2	Dengue virus 2	EU920836	10724	Unknown	Martinique
MAR-Nov-05-DENV-2	Dengue virus 2	EU920843	10647	Unknown	Martinique
MAR-Oct-05-DENV-2	Dengue virus 2	EU920841	10665	Unknown	Martinique
MAR-Sep-05-DENV-2	Dengue virus 2	EU920839	10711	Unknown	Martinique
Mara4(2)	Dengue virus 2	AF100466	10682	Unknown	-N/A-
MD1240(2)	Dengue virus 2	FM210231	10685	Human	Viet Nam
MD1244(2)	Dengue virus 2	FM210239	10672	Human	Viet Nam
MD1270(2)	Dengue virus 2	FM210232	10685	Human	Viet Nam
MD1272-2	Dengue virus 2	FM210233	10685	Human	Viet Nam
MD1273(2)	Dengue virus 2	FM210240	10602	Unknown	Viet Nam
MD1275(2)	Dengue virus 2	FM210241	10611	Human	Viet Nam
MD1279(2)	Dengue virus 2	FM210242	10690	Human	Viet Nam
MD1280-2	Dengue virus 2	FM210243	10604	Human	Viet Nam
MD1284-2	Dengue virus 2	FM210244	10683	Human	Viet Nam
MD1366(2)	Dengue virus 2	FM210234	10685	Human	Viet Nam
MD1504-2	Dengue virus 2	FM210213	10694	Human	Viet Nam
MD1515-2	Dengue virus 2	FM210235	10652	Human	Viet Nam
MD1520-2	Dengue virus 2	FM210245	10673	Human	Viet Nam
MD1533-2	Dengue virus 2	FM210205	10691	Human	Viet Nam
MD1600(2)	Dengue virus 2	FM210206	10691	Human	Viet Nam
MD1618(2)	Dengue virus 2	FM210246	10614	Human	Viet Nam
MD1619(2)	Dengue virus 2	FM210207	10691	Human	Viet Nam
MD510(2)	Dengue virus 2	FM210238	10659	Human	Viet Nam
MD518(2)	Dengue virus 2	FM210224	10689	Human	Viet Nam
MD594(2)	Dengue virus 2	FM210225	10684	Human	Viet Nam
MD861(2)	Dengue virus 2	FM210226	10685	Human	Viet Nam
MD863-2	Dengue virus 2	FM210227	10685	Human	Viet Nam
MD902(2)	Dengue virus 2	FM210212	10486	Human	Viet Nam
MD903(2)	Dengue virus 2	FM210204	10559	Human	Viet Nam
MD917-2	Dengue virus 2	FM210228	10685	Human	Viet Nam
MD919(2)	Dengue virus 2	FM210229	10392	Human	Viet Nam
MD944(2)	Dengue virus 2	FM210230	10687	Human	Viet Nam
MKS-0068-2	Dengue virus 2	KC762665	10723	Human	Indonesia
MKS-0071-2	Dengue virus 2	KC762669	10723	Human	Indonesia
MKS-0082-2	Dengue virus 2	KC762658	10723	Human	Indonesia
MKS-0084-2	Dengue virus 2	KC762660	10723	Human	Indonesia
MKS-0091-2	Dengue virus 2	KC762670	10723	Human	Indonesia
MKS-0099-2	Dengue virus 2	KC762676	10723	Human	Indonesia
MKS-0297-2	Dengue virus 2	KC762662	10723	Human	Indonesia
MKS-0412-2	Dengue virus 2	KC762661	10723	Human	Indonesia
MKS-0417-2	Dengue virus 2	KC762655	10723	Human	Indonesia
MKS-0502-2	Dengue virus 2	KC762656	10723	Human	Indonesia
MKS-2018-2	Dengue virus 2	KC762673	10723	Human	Indonesia
MKS-2024-2	Dengue virus 2	KC762663	10723	Human	Indonesia
MKS-2032-2	Dengue virus 2	KC762675	10723	Human	Indonesia
MKS-2108-2	Dengue virus 2	KC762671	10723	Human	Indonesia
MKS-2145-2	Dengue virus 2	KC762664	10723	Human	Indonesia
MKS-2167-2	Dengue virus 2	KC762674	10723	Human	Indonesia
MKS-2198-2	Dengue virus 2	KC762672	10723	Human	Indonesia
MKS-2204-2	Dengue virus 2	KC762667	10723	Human	Indonesia
MKS-2210-2	Dengue virus 2	KC762659	10723	Human	Indonesia
MKS-2234-2	Dengue virus 2	KC762657	10723	Human	Indonesia
MKS-3007-2	Dengue virus 2	KC762666	10723	Human	Indonesia
MKS-IF011-2	Dengue virus 2	KC762677	10723	Human	Indonesia
MKS-IF014-2	Dengue virus 2	KC762668	10723	Human	Indonesia
MKS-WS73-2	Dengue virus 2	KC762678	10723	Human	Indonesia
MKS-WS79a-2	Dengue virus 2	KC762679	10723	Human	Indonesia
MKS-WS80-2	Dengue virus 2	KC762680	10723	Human	Indonesia
New Guinea C derivative	Dengue virus 2	FJ390389	10632	Human	Papua New Guinea
New Guinea C-2	Dengue virus 2	AF038403	10724	Unknown	-N/A-
New Guinea C-2	Dengue virus 2	KM204118	10723	Human	Papua New Guinea
New Guinea C/PUO-218 hybrid(2)	Dengue virus 2	AF038402	10724	Unknown	-N/A-
New Guinea-C(2)	Dengue virus 2	M29095	10723	Unknown	-N/A-
Od2112-2	Dengue virus 2	JQ955624	10670	Human	India
P23085 INDI-60-2	Dengue virus 2	KJ918750	10723	Human	India
P8-1407-2	Dengue virus 2	EF105379	10719	Unknown	Malaysia
PDK-53(2)	Dengue virus 2	U87412	10723	Unknown	-N/A-
PL046(type 2)	Dengue virus 2	AJ968413	10723	Human	Taiwan
PL046(type 2)	Dengue virus 2	KJ734727	10723	Unknown	Taiwan
PM33974-2	Dengue virus 2	EF105378	10722	Unknown	Guinea
QHD13CAIQ	Dengue virus 2	KF479233	10723	Human	China
RR44-2	Dengue virus 2	JQ955623	10670	Human	India
S1 vaccine(2)	Dengue virus 2	M19197	10703	Unknown	-N/A-
S16803-dengue 2	Dengue virus 2	GU289914	10723	Human	Thailand
SG(EHI)D2/0466Y07-2	Dengue virus 2	KR779782	10723	Human	Singapore
SGEHI(D2)0017Y06-2	Dengue virus 2	JN851113	10176	Human	Singapore
SGEHI(D2)0099Y07(2)	Dengue virus 2	GU370050	10200	Human	Singapore
SGEHI(D2)0194Y08-2	Dengue virus 2	JN851119	10176	Human	Singapore

	SGEHI(D2)0204Y06-2	Dengue virus 2	JN851129	10176	Human	Singapore
	SGEHI(D2)0232Y06-2	Dengue virus 2	JN851128	10176	Human	Singapore
	SGEHI(D2)0270Y05-2	Dengue virus 2	JN851130	10176	Human	Singapore
	SGEHI(D2)0345Y05-2	Dengue virus 2	JN851125	10176	Human	Singapore
	SGEHI(D2)0377Y04-2	Dengue virus 2	JN851123	10176	Human	Singapore
	SGEHI(D2)0391Y08-2	Dengue virus 2	JN851118	10176	Human	Singapore
	SGEHI(D2)0431Y07-2	Dengue virus 2	JN851117	10176	Human	Singapore
	SGEHI(D2)0462Y05-2	Dengue virus 2	JN851124	10176	Human	Singapore
	SGEHI(D2)0522Y07-2	Dengue virus 2	JN851114	10176	Human	Singapore
	SGEHI(D2)0578Y05-2	Dengue virus 2	JN851126	10176	Human	Singapore
	SGEHI(D2)0615Y08-2	Dengue virus 2	JN851121	10176	Human	Singapore
	SGEHI(D2)0642Y07-2	Dengue virus 2	JN851115	10176	Human	Singapore
	SGEHI(D2)0685Y04-2	Dengue virus 2	JN851127	10176	Human	Singapore
	SGEHI(D2)0722Y07-2	Dengue virus 2	JN851120	10176	Human	Singapore
	SGEHI(D2)0762Y05-2	Dengue virus 2	JN851131	10176	Human	Singapore
	SGEHI(D2)0950Y08-2	Dengue virus 2	JN851122	10176	Human	Singapore
	SGEHI(D2)1079Y07-2	Dengue virus 2	JN851116	10176	Human	Singapore
	SGEHI(D2)1158Y08(2)	Dengue virus 2	GU370051	10527	Human	Singapore
	SUR-Aug-05-DENV-2	Dengue virus 2	EU920838	10659	Unknown	Suriname
	SUR-Oct-05-DENV-2	Dengue virus 2	EU920842	10726	Unknown	Suriname
	Taiwan-1008DHF(2)	Dengue virus 2	AY776328	10485	Unknown	Taiwan
	TB16i(2)	Dengue virus 2	AY858036	10723	Human	Indonesia
	ThD2_0017_98(2)	Dengue virus 2	DQ181799	10723	Unknown	Thailand
	ThD2_0026_88(2)	Dengue virus 2	DQ181802	10723	Unknown	Thailand
	ThD2_0038_74(2)	Dengue virus 2	DQ181806	10723	Unknown	Thailand
	ThD2_0055_99(2)	Dengue virus 2	DQ181798	10723	Unknown	Thailand
	ThD2_0078_01(2)	Dengue virus 2	DQ181797	10723	Unknown	Thailand
	ThD2_0168_79(2)	Dengue virus 2	DQ181805	10723	Unknown	Thailand
	ThD2_0263_95(2)	Dengue virus 2	DQ181800	10723	Unknown	Thailand
	ThD2_0284_90(2)	Dengue virus 2	DQ181801	10723	Unknown	Thailand
	ThD2_0433_85(2)	Dengue virus 2	DQ181803	10723	Unknown	Thailand
	ThD2_0498_84(2)	Dengue virus 2	DQ181804	10723	Unknown	Thailand
	ThNH-28/93(2)	Dengue virus 2	AF022435	10723	Unknown	-N/A-
	ThNH-52/93(2)	Dengue virus 2	AF022436	10723	Unknown	-N/A-
	ThNH-7/93-2	Dengue virus 2	AF022434	10724	Unknown	-N/A-
	ThNH-p11/93(2)	Dengue virus 2	AF022437	10723	Unknown	-N/A-
	ThNH-p12/93(2)	Dengue virus 2	AF022438	10723	Unknown	-N/A-
	ThNH-p14/93(2)	Dengue virus 2	AF022439	10723	Unknown	-N/A-
	ThNH-p16/93(2)	Dengue virus 2	AF022440	10723	Unknown	-N/A-
	ThNH-p36/93(2)	Dengue virus 2	AF022441	10723	Unknown	-N/A-
	ThNH29/93(2)	Dengue virus 2	AF169678	10723	Unknown	-N/A-
	ThNH36/93(2)	Dengue virus 2	AF169679	10723	Unknown	-N/A-
	ThNH45/93(2)	Dengue virus 2	AF169680	10723	Unknown	-N/A-
	ThNH54/93(2)	Dengue virus 2	AF169682	10723	Unknown	-N/A-
	ThNH55/93(2)	Dengue virus 2	AF169681	10723	Unknown	-N/A-
	ThNH62/93(2)	Dengue virus 2	AF169683	10723	Unknown	-N/A-
	ThNH63/93(2)	Dengue virus 2	AF169684	10723	Unknown	-N/A-
	ThNH69/93(2)	Dengue virus 2	AF169685	10723	Unknown	-N/A-
	ThNH73/93(2)	Dengue virus 2	AF169686	10723	Unknown	-N/A-
	ThNH76/93(2)	Dengue virus 2	AF169687	10723	Unknown	-N/A-
	ThNH81/93(2)	Dengue virus 2	AF169688	10723	Unknown	-N/A-
	Tonga/74(2)	Dengue virus 2	AY744147	10713	Unknown	Tonga
	TSV01-2	Dengue virus 2	AY037116	10723	Unknown	Australia
	UNKNOWN-AB543624	Dengue virus 2	AB543624	10731	Human	-N/A-
	UNKNOWN-FV537258	Dengue virus 2	FV537258	10703	Unknown	-N/A-
	UNKNOWN-FV537259	Dengue virus 2	FV537259	10703	Unknown	-N/A-
	UNKNOWN-FV537260	Dengue virus 2	FV537260	10703	Unknown	-N/A-
	UNKNOWN-FV537261	Dengue virus 2	FV537261	10703	Unknown	-N/A-
	UNKNOWN-HH961661(2)	Dengue virus 2	HH961661	10724	Unknown	-N/A-
	UNKNOWN-JC562942	Dengue virus 2	JC562942	10723	Unknown	-N/A-
	UNKNOWN-JE963511	Dengue virus 2	JE963511	10723	Unknown	-N/A-
	UNKNOWN-JN368476-2	Dengue virus 2	JN368476	10651	Human	Cambodia
	VE_61069_2006-2	Dengue virus 2	HQ332184	10722	Human	Venezuela
	VE_61082_2007-2	Dengue virus 2	HQ332185	10722	Human	Venezuela
	VE_61095_2007-2	Dengue virus 2	HQ332190	10722	Human	Venezuela
	VE_61115_2007-2	Dengue virus 2	HQ332186	10722	Human	Venezuela
	VE_61133_2007-2	Dengue virus 2	HQ332187	10722	Human	Venezuela
	VE_61136_2007-2	Dengue virus 2	HQ332188	10722	Human	Venezuela
	VE_61154_2007-2	Dengue virus 2	HQ332189	10722	Human	Venezuela
	Ven2-2	Dengue virus 2	AF100465	10674	Unknown	-N/A-
	VNHCM18-C/02	Dengue virus 2	AB479041	10647	Unknown	Viet Nam
	VNHCM18-K/02	Dengue virus 2	AB479042	10647	Unknown	Viet Nam
	ZH1340(2)	Dengue virus 2	EU359009	10723	Human	China
	ZH413-2-2	Dengue virus 2	KC131142	10673	Human	China
	ZS01/01	Dengue virus 2	KR920365	10723	Unknown	-N/A-
	ZS01/01(2)	Dengue virus 2	EF051521	10723	Unknown	China
	16681-2	Dengue virus 2 Thailand/16681/84	U87411	10723	Unknown	
**DENV3**	07CHLS001(3)	Dengue virus 3	EU367962	10707	Human	China
	101905/BR-PE/03	Dengue virus 3	JX669489	10709	Human	Brazil
	129/BR-PE/04	Dengue virus 3	JX669498	10707	Human	Brazil
	13GDZDVS30B-III	Dengue virus 3	KF954946	10686	Human	China
	13GDZDVS30C-III	Dengue virus 3	KF954947	10675	Human	China
	13GDZDVS30D-III	Dengue virus 3	KF954948	10678	Human	China
	13GDZDVS30E-III	Dengue virus 3	KF954949	10677	Human	China
	13GDZSDV30A-III	Dengue virus 3	KF954945	10686	Human	China
	145/BR-PE/04	Dengue virus 3	JX669495	10707	Human	Brazil
	161/BR-PE/04	Dengue virus 3	JX669499	10707	Human	Brazil
	206/BR-PE/05	Dengue virus 3	JX669492	10709	Human	Brazil
	249/BR-PE/05	Dengue virus 3	JX669500	10707	Human	Brazil
	255/BR-PE/05	Dengue virus 3	JX669497	10709	Human	Brazil
	263/BR-PE/05	Dengue virus 3	JX669501	10707	Human	Brazil
	277/BR-PE/05	Dengue virus 3	JX669494	10710	Human	Brazil
	283/BR-PE/05	Dengue virus 3	JX669502	10705	Human	Brazil
	314/BR-PE/06	Dengue virus 3	JX669503	10707	Human	Brazil
	339/BR-PE/05	Dengue virus 3	JX669493	10709	Human	Brazil
	411/BR-PE/06	Dengue virus 3	JX669504	10707	Human	Brazil
	418/BR-PE/06	Dengue virus 3	JX669505	10707	Human	Brazil
	420/BR-PE/06	Dengue virus 3	JX669506	10708	Human	Brazil
	423/BR-PE/06	Dengue virus 3	JX669507	10707	Human	Brazil
	424/BR-PE/06	Dengue virus 3	JX669508	10708	Human	Brazil
	603/BR-PE/06	Dengue virus 3	JX669496	10709	Human	Brazil
	80-2(3)	Dengue virus 3	AF317645	10696	Unknown	China
	81257/BR-PE/02	Dengue virus 3	JX669491	10708	Human	Brazil
	85469/BR-PE/02	Dengue virus 3	JX669490	10707	Human	Brazil
	95016/BR-PE/02	Dengue virus 3	KC425219	10707	Human	Brazil
	95TW466(3)	Dengue virus 3	DQ675519	10707	Unknown	Taiwan
	98901403 DSS DV-3(3)	Dengue virus 3	AB189125	10707	Human	Indonesia
	98901437 DSS DV-3(3)	Dengue virus 3	AB189126	10707	Human	Indonesia
	98901517 DHF DV-3(3)	Dengue virus 3	AB189127	10707	Human	Indonesia
	98902890 DF DV-3(3)	Dengue virus 3	AB189128	10707	Human	Indonesia
	98TW182(3)	Dengue virus 3	DQ675520	10706	Unknown	Indonesia
	98TW349(3)	Dengue virus 3	DQ675521	10707	Unknown	Taiwan
	98TW358(3)	Dengue virus 3	DQ675522	10707	Unknown	Taiwan

98TW360(3)	Dengue virus 3	DQ675523	10707	Unknown	Taiwan
98TW364(3)	Dengue virus 3	DQ675524	10707	Unknown	Taiwan
98TW368(3)	Dengue virus 3	DQ675525	10707	Unknown	Taiwan
98TW388(3)	Dengue virus 3	DQ675526	10707	Unknown	Taiwan
98TW390(3)	Dengue virus 3	DQ675527	10707	Unknown	Taiwan
98TW407(3)	Dengue virus 3	DQ675528	10707	Unknown	Taiwan
98TW414(3)	Dengue virus 3	DQ675529	10707	Unknown	Taiwan
98TW434(3)	Dengue virus 3	DQ675530	10707	Unknown	Taiwan
98TW503(3)	Dengue virus 3	DQ675531	10707	Unknown	Taiwan
98TWmosq(3)	Dengue virus 3	DQ675532	10707	Unknown	Taiwan
99TW628(3)	Dengue virus 3	DQ675533	10707	Unknown	Taiwan
BA51(3)	Dengue virus 3	AY858037	10707	Human	Indonesia
BDH02-1(3)	Dengue virus 3	AY496871	10707	Unknown	Bangladesh
BDH02-3(3)	Dengue virus 3	AY496873	10707	Unknown	Bangladesh
BDH02-4(3)	Dengue virus 3	AY496874	10707	Unknown	Bangladesh
BDH02-7(3)	Dengue virus 3	AY496877	10707	Unknown	Bangladesh
BR DEN3 290-02(3)	Dengue virus 3	EF629369	10707	Unknown	Brazil
BR DEN3 95-04(3)	Dengue virus 3	EF629366	10707	Unknown	Brazil
BR DEN3 97-04(3)	Dengue virus 3	EF629367	10707	Unknown	Brazil
BR DEN3 98-04(3)	Dengue virus 3	EF629368	10603	Unknown	Brazil
BR DEN3 RO1-02(3)	Dengue virus 3	EF629370	10696	Unknown	Brazil
BR73354mosq/01(3)	Dengue virus 3	FJ177308	10652	Unknown	Brazil
BR74886/02(3)	Dengue virus 3	AY679147	10703	Unknown	Brazil
C0331/94(3)	Dengue virus 3	AY876494	10707	Unknown	Thailand
C0360/94	Dengue virus 3	KJ737429	10707	Human	Thailand
C0360/94(3)	Dengue virus 3	AY923865	10707	Unknown	Thailand
CH53489-3	Dengue virus 3	DQ863638	10707	Human	Thailand
D3/H/IMTSSA-MART/1999/1243(3)	Dengue virus 3	AY099337	10707	Unknown	Martinique
D3/H/IMTSSA-SRI/2000/1266	Dengue virus 3	NC_001475 *	10707	Unknown	Sri Lanka
D3/H/IMTSSA-SRI/2000/1266-3	Dengue virus 3	AY099336	10707	Unknown	Sri Lanka
D3/Hu/TL018NIID/2005(3)	Dengue virus 3	AB214879	10707	Human	East Timor
D3/Hu/TL029NIID/2005(3)	Dengue virus 3	AB214880	10707	Human	East Timor
D3/Hu/TL109NIID/2005(3)	Dengue virus 3	AB214881	10707	Human	East Timor
D3/Hu/TL129NIID/2005(3)	Dengue virus 3	AB214882	10707	Human	East Timor
D3/Pakistan/43298/2006-3	Dengue virus 3	KF041259	10675	Human	Pakistan
D3/Pakistan/45251/2009-3	Dengue virus 3	KF041258	10675	Human	Pakistan
D3/Pakistan/52440/2006-3	Dengue virus 3	KF041257	10675	Human	Pakistan
D3/Pakistan/55505/2007-3	Dengue virus 3	KF041255	10675	Human	Pakistan
D3/Pakistan/55709/2006-3	Dengue virus 3	KF041256	10675	Human	Pakistan
D3/Pakistan/56/2008-3	Dengue virus 3	KF041254	10675	Human	Pakistan
D3/SG/05K2400DK1/2005(3)	Dengue virus 3	EU081193	10707	Unknown	Singapore
D3/SG/05K2406DK1/2005(3)	Dengue virus 3	EU081194	10707	Unknown	-N/A-
D3/SG/05K2418DK1/2005(3)	Dengue virus 3	EU081195	10707	Unknown	Singapore
D3/SG/05K2899DK1/2005(3)	Dengue virus 3	EU081196	10675	Unknown	Singapore
D3/SG/05K2918DK1/2005(3)	Dengue virus 3	EU081197	10707	Unknown	Singapore
D3/SG/05K2933DK1/2005(3)	Dengue virus 3	EU081198	10707	Unknown	Singapore
D3/SG/05K3305DK1/2005(3)	Dengue virus 3	EU081199	10707	Unknown	Singapore
D3/SG/05K3312DK1/2005(3)	Dengue virus 3	EU081200	10707	Unknown	Singapore
D3/SG/05K3314DK1/2005(3)	Dengue virus 3	EU081201	10707	Unknown	Singapore
D3/SG/05K3316DK1/2005(3)	Dengue virus 3	EU081202	10671	Unknown	Singapore
D3/SG/05K3324DK1/2005(3)	Dengue virus 3	EU081203	10671	Unknown	Singapore
D3/SG/05K3325DK1/2005(3)	Dengue virus 3	EU081204	10671	Unknown	Singapore
D3/SG/05K3329DK1/2005(3)	Dengue virus 3	EU081205	10675	Unknown	Singapore
D3/SG/05K3887DK1/2005(3)	Dengue virus 3	EU081206	10673	Unknown	Singapore
D3/SG/05K3897DK1/2005(3)	Dengue virus 3	EU081207	10707	Unknown	Singapore
D3/SG/05K3900DK1/2005(3)	Dengue virus 3	EU081208	10707	Unknown	Singapore
D3/SG/05K3912DK1/2005(3)	Dengue virus 3	EU081209	10673	Unknown	Singapore
D3/SG/05K3913DK1/2005(3)	Dengue virus 3	EU081210	10707	Unknown	Singapore
D3/SG/05K3923DK1/2005(3)	Dengue virus 3	EU081211	10675	Unknown	Singapore
D3/SG/05K3927DK1/2005(3)	Dengue virus 3	EU081212	10707	Unknown	Singapore
D3/SG/05K3928DK1/2005(3)	Dengue virus 3	EU081213	10707	Unknown	Singapore
D3/SG/05K4141DK1/2005(3)	Dengue virus 3	EU081214	10707	Unknown	Singapore
D3/SG/05K4144DK1/2005(3)	Dengue virus 3	EU081215	10675	Unknown	Singapore
D3/SG/05K4157DK1/2005(3)	Dengue virus 3	EU081216	10675	Unknown	Singapore
D3/SG/05K4159DK1/2005(3)	Dengue virus 3	EU081217	10707	Unknown	Singapore
D3/SG/05K4168DK1/2005(3)	Dengue virus 3	EU081218	10707	Unknown	Singapore
D3/SG/05K4176DK1/2005(3)	Dengue virus 3	EU081219	10707	Unknown	Singapore
D3/SG/05K4182DK1/2005(3)	Dengue virus 3	EU081220	10707	Unknown	Singapore
D3/SG/05K4440DK1/2005(3)	Dengue virus 3	EU081221	10706	Unknown	Singapore
D3/SG/05K4454DK1/2005(3)	Dengue virus 3	EU081222	10707	Unknown	Singapore
D3/SG/05K4477DK1/2005(3)	Dengue virus 3	EU081223	10706	Unknown	Singapore
D3/SG/05K4647DK1/2005(3)	Dengue virus 3	EU081224	10675	Unknown	Singapore
D3/SG/05K4648DK1/2005(3)	Dengue virus 3	EU081225	10707	Unknown	Singapore
D3/SG/05K791DK1/2005(3)	Dengue virus 3	EU081182	10673	Unknown	Singapore
D3/SG/05K797DK1/2005(3)	Dengue virus 3	EU081183	10705	Unknown	Singapore
D3/SG/05K802DK1/2005(3)	Dengue virus 3	EU081184	10673	Unknown	Singapore
D3/SG/05K805DK1/2005(3)	Dengue virus 3	EU081185	10707	Unknown	Singapore
D3/SG/05K827DK1/2005(3)	Dengue virus 3	EU081186	10695	Unknown	Singapore
D3/SG/05K843DK1/2005(3)	Dengue virus 3	EU081187	10673	Unknown	Singapore
D3/SG/05K845DK1/2005(3)	Dengue virus 3	EU081188	10705	Unknown	Singapore
D3/SG/05K852DK1/2005(3)	Dengue virus 3	EU081189	10707	Unknown	Singapore
D3/SG/05K863DK1/2005(3)	Dengue virus 3	EU081190	10706	Unknown	Singapore
D3/SG/05K868DK1/2005(3)	Dengue virus 3	EU081191	10707	Unknown	Singapore
D3/SG/05K871DK1/2005(3)	Dengue virus 3	EU081192	10673	Unknown	Singapore
D3/SG/SS710/2004(3)	Dengue virus 3	EU081181	10707	Unknown	Singapore
D3BR/ACN/2007	Dengue virus 3	JF808121	10707	Human	Brazil
D3BR/AL95/2009	Dengue virus 3	JF808120	10707	Human	Brazil
D3BR/BR8/04	Dengue virus 3	JF808119	10707	Human	Brazil
D3BR/BV4/02	Dengue virus 3	JF808118	10707	Human	Brazil
D3BR/CU6/02	Dengue virus 3	JF808127	10707	Human	Brazil
D3BR/MR9/03	Dengue virus 3	JF808124	10699	Human	Brazil
D3BR/PV1/03	Dengue virus 3	JF808126	10707	Human	Brazil
D3BR/RP1/2003(3)	Dengue virus 3	EF643017	10707	Human	Brazil
D3BR/SL3/02	Dengue virus 3	JF808125	10707	Human	Brazil
D3PY/AS10/03	Dengue virus 3	JF808129	10707	Human	Paraguay
D3PY/AS12/02	Dengue virus 3	JF808123	10707	Human	Paraguay
D3PY/PJ4/03	Dengue virus 3	JF808128	10707	Human	Paraguay
D3PY/SUS/2003	Dengue virus 3	JF808122	10707	Human	Paraguay
D83-144	Dengue virus 3	KJ737430	10707	Human	Thailand
DEL-72-Dengue 3	Dengue virus 3	GQ466079	10680	Human	India
den3_88(3)	Dengue virus 3	AY858038	10707	Human	Indonesia
den3_98(3)	Dengue virus 3	AY858039	10707	Human	Indonesia
DENV-3/AI/BID-V2976/2001(3)	Dengue virus 3	FJ898462	10663	Human	Anguilla
DENV-3/BR/BID-V2380/2001(3)	Dengue virus 3	FJ913015	10663	Human	Brazil
DENV-3/BR/BID-V2383/2002	Dengue virus 3	KF955473	10459	Human	Brazil
DENV-3/BR/BID-V2387/2003(3)	Dengue virus 3	FJ850079	10663	Human	Brazil
DENV-3/BR/BID-V2388/2003(3)	Dengue virus 3	FJ850080	10663	Human	Brazil
DENV-3/BR/BID-V2391/2004(3)	Dengue virus 3	FJ850083	10663	Human	Brazil
DENV-3/BR/BID-V2394/2005(3)	Dengue virus 3	FJ850086	10652	Human	Brazil
DENV-3/BR/BID-V2397/2006(3)	Dengue virus 3	FJ850089	10652	Human	Brazil
DENV-3/BR/BID-V2400/2007(3)	Dengue virus 3	FJ850092	10648	Human	Brazil
DENV-3/BR/BID-V2403/2008(3)	Dengue virus 3	FJ850094	10662	Human	Brazil
DENV-3/BR/BID-V2977/2001(3)	Dengue virus 3	FJ898446	10661	Human	Brazil

DENV-3/BR/BID-V2983/2003(3)	Dengue virus 3	FJ898447	10663	Human	Brazil
DENV-3/BR/BID-V3417/2006(3)	Dengue virus 3	GU131844	10594	Human	Brazil
DENV-3/BR/BID-V3423/2006(3)	Dengue virus 3	GU131845	10525	Human	Brazil
DENV-3/BR/BID-V3424/2006(3)	Dengue virus 3	GU131846	10616	Human	Brazil
DENV-3/BR/BID-V3427/2006(3)	Dengue virus 3	GU131847	10525	Human	Brazil
DENV-3/BR/BID-V3429/2006(3)	Dengue virus 3	GU131848	10525	Human	Brazil
DENV-3/BR/BID-V3430/2006(3)	Dengue virus 3	GU131849	10525	Human	Brazil
DENV-3/BR/BID-V3431/2006(3)	Dengue virus 3	GQ868546	10663	Human	Brazil
DENV-3/BR/BID-V3434/2006(3)	Dengue virus 3	GU131850	10525	Human	Brazil
DENV-3/BR/BID-V3435/2006(3)	Dengue virus 3	GU131851	10525	Human	Brazil
DENV-3/BR/BID-V3441/2006(3)	Dengue virus 3	GU131852	10525	Human	Brazil
DENV-3/BR/BID-V3442/2006(3)	Dengue virus 3	GU131853	10525	Human	Brazil
DENV-3/BR/BID-V3444/2006(3)	Dengue virus 3	GQ868547	10655	Human	Brazil
DENV-3/BR/BID-V3446/2006(3)	Dengue virus 3	GU131854	10517	Human	Brazil
DENV-3/BR/BID-V3451/2006(3)	Dengue virus 3	GU131855	10616	Human	Brazil
DENV-3/BR/BID-V3456/2006(3)	Dengue virus 3	GU131856	10616	Human	Brazil
DENV-3/BR/BID-V3457/2006(3)	Dengue virus 3	GU131857	10616	Human	Brazil
DENV-3/BR/BID-V3460/2006(3)	Dengue virus 3	GU131858	10525	Human	Brazil
DENV-3/BR/BID-V3463/2006(3)	Dengue virus 3	GQ868548	10663	Human	Brazil
DENV-3/BR/BID-V3464/2006(3)	Dengue virus 3	GU131859	10525	Human	Brazil
DENV-3/BR/BID-V3465/2006(3)	Dengue virus 3	GU131860	10517	Human	Brazil
DENV-3/BR/BID-V3469/2007(3)	Dengue virus 3	GU131861	10526	Human	Brazil
DENV-3/BR/BID-V3470/2007(3)	Dengue virus 3	GU131862	10525	Human	Brazil
DENV-3/BR/BID-V3584/2006(3)	Dengue virus 3	GU131865	10501	Human	Brazil
DENV-3/BR/BID-V3585/2007(3)	Dengue virus 3	GU131866	10526	Human	Brazil
DENV-3/BR/BID-V3588/2007(3)	Dengue virus 3	GU131867	10525	Human	Brazil
DENV-3/BR/BID-V3589/2007(3)	Dengue virus 3	GU131868	10517	Human	Brazil
DENV-3/BR/BID-V3590/2007(3)	Dengue virus 3	GU131869	10525	Human	Brazil
DENV-3/BR/BID-V3591/2007(3)	Dengue virus 3	GU131870	10525	Human	Brazil
DENV-3/BR/BID-V3593/2007(3)	Dengue virus 3	GU131871	10510	Human	Brazil
DENV-3/BR/BID-V3597/2007(3)	Dengue virus 3	GU131872	10608	Human	Brazil
DENV-3/BR/BID-V3598/2007(3)	Dengue virus 3	GU131873	10402	Human	Brazil
DENV-3/BR/BID-V3601/2007(3)	Dengue virus 3	GU131874	10525	Human	Brazil
DENV-3/BR/BID-V3605/2007(3)	Dengue virus 3	GU131875	10525	Human	Brazil
DENV-3/BR/BID-V3606/2007(3)	Dengue virus 3	GU131876	10516	Human	Brazil
DENV-3/BR/BID-V3609/2007(3)	Dengue virus 3	GU131877	10616	Human	Brazil
DENV-3/BR/BID-V3615/2007(3)	Dengue virus 3	GU131878	10638	Human	Brazil
DENV-3/CK/BID-V2972/1991(3)	Dengue virus 3	FJ898455	10663	Human	Cook Islands
DENV-3/CO/BID-V2978/2001(3)	Dengue virus 3	GQ199891	10663	Human	Colombia
DENV-3/CO/BID-V2984/2003(3)	Dengue virus 3	FJ898443	10663	Human	Colombia
DENV-3/CO/BID-V2986/2005(3)	Dengue virus 3	FJ898444	10663	Human	Colombia
DENV-3/CO/BID-V2988/2007(3)	Dengue virus 3	FJ898445	10664	Human	Colombia
DENV-3/CO/BID-V3392/2001(3)	Dengue virus 3	GU131950	10616	Human	Colombia
DENV-3/CO/BID-V3393/2002(3)	Dengue virus 3	GQ868571	10663	Human	Colombia
DENV-3/CO/BID-V3394/2003(3)	Dengue virus 3	GU131951	10638	Human	Colombia
DENV-3/CO/BID-V3395/2003(3)	Dengue virus 3	GQ868572	10652	Human	Colombia
DENV-3/CO/BID-V3397/2003(3)	Dengue virus 3	GQ868573	10644	Human	Colombia
DENV-3/CO/BID-V3398/2003(3)	Dengue virus 3	GQ868574	10663	Human	Colombia
DENV-3/CO/BID-V3399/2003(3)	Dengue virus 3	GU131952	10638	Human	Colombia
DENV-3/CO/BID-V3400/2004(3)	Dengue virus 3	GQ868575	10663	Human	Colombia
DENV-3/CO/BID-V3401/2004(3)	Dengue virus 3	GU131953	10588	Human	Colombia
DENV-3/CO/BID-V3402/2005(3)	Dengue virus 3	GQ868576	10663	Human	Colombia
DENV-3/CO/BID-V3403/2005(3)	Dengue virus 3	GQ868577	10646	Human	Colombia
DENV-3/CO/BID-V3404/2006(3)	Dengue virus 3	GU131954	10525	Human	Colombia
DENV-3/CO/BID-V3405/2007(3)	Dengue virus 3	GQ868578	10659	Human	Colombia
DENV-3/EC/BID-V2975/2000(3)	Dengue virus 3	FJ898457	10663	Human	Ecuador
DENV-3/GD/BID-V3930/2002	Dengue virus 3	KF955505	10653	Human	Grenada
DENV-3/GY/BID-V2980/2002(3)	Dengue virus 3	FJ898464	10663	Human	Guyana
DENV-3/IN/BID-V2417/1984	Dengue virus 3	KF955477	10525	Human	India
DENV-3/IND/58760/2005-3	Dengue virus 3	JQ922556	10652	Human	India
DENV-3/IND/59826/2005-3	Dengue virus 3	JQ922557	10657	Human	India
DENV-3/IND/664481/1966-3	Dengue virus 3	JQ922555	10663	Human	India
DENV-3/IPC/BID-V3804/2008(3)	Dengue virus 3	GU131903	10509	Human	Cambodia
DENV-3/IPC/BID-V3807/2005(3)	Dengue virus 3	GU131904	10638	Human	Cambodia
DENV-3/IPC/BID-V3808/2008(3)	Dengue virus 3	GU131905	10638	Human	Cambodia
DENV-3/IPC/BID-V3809/2003(3)	Dengue virus 3	GU131906	10625	Human	Cambodia
DENV-3/IPC/BID-V3815/2006(3)	Dengue virus 3	GU131907	10525	Human	Cambodia
DENV-3/IPC/BID-V3819/2006(3)	Dengue virus 3	GQ868634	10663	Human	Cambodia
DENV-3/IPC/BID-V3820/2006(3)	Dengue virus 3	GU131908	10511	Human	Cambodia
DENV-3/IPC/BID-V3822/2006(3)	Dengue virus 3	GU131909	10509	Human	Cambodia
DENV-3/IPC/BID-V3824/2006(3)	Dengue virus 3	GU131910	10623	Human	Cambodia
DENV-3/IPC/BID-V3825/2006(3)	Dengue virus 3	GU131911	10524	Human	Cambodia
DENV-3/IPC/BID-V3826/2007(3)	Dengue virus 3	GU131912	10522	Human	Cambodia
DENV-3/IPC/BID-V3827/2007(3)	Dengue virus 3	GU131913	10648	Human	Cambodia
DENV-3/IPC/BID-V3828/2007(3)	Dengue virus 3	GU131914	10619	Human	Cambodia
DENV-3/IPC/BID-V3830/2007(3)	Dengue virus 3	GU131915	10523	Human	Cambodia
DENV-3/IPC/BID-V3831/2007(3)	Dengue virus 3	GU131916	10625	Human	Cambodia
DENV-3/IPC/BID-V3832/2007(3)	Dengue virus 3	GU131917	10616	Human	Cambodia
DENV-3/IPC/BID-V3833/2007(3)	Dengue virus 3	GU131918	10497	Human	Cambodia
DENV-3/IPC/BID-V4280/2006(3)	Dengue virus 3	GU131933	10487	Human	Cambodia
DENV-3/IPC/BID-V4282/2007(3)	Dengue virus 3	GU131934	10659	Human	Cambodia
DENV-3/IPC/BID-V4283/2007(3)	Dengue virus 3	GU131935	10637	Human	Cambodia
DENV-3/IPC/BID-V4284/2007(3)	Dengue virus 3	GU131936	10660	Human	Cambodia
DENV-3/IPC/BID-V4286/2007(3)	Dengue virus 3	GU131937	10657	Human	Cambodia
DENV-3/IPC/BID-V4290/2007(3)	Dengue virus 3	GU131938	10647	Human	Cambodia
DENV-3/IPC/BID-V4294/2007(3)	Dengue virus 3	GU131939	10644	Human	Cambodia
DENV-3/IPC/BID-V4297/2007(3)	Dengue virus 3	GU131940	10632	Human	Cambodia
DENV-3/IPC/BID-V4298/2007(3)	Dengue virus 3	GU131941	10638	Human	Cambodia
DENV-3/IPC/BID-V4300/2007(3)	Dengue virus 3	GU131942	10633	Human	Cambodia
DENV-3/IPC/BID-V4302/2007(3)	Dengue virus 3	GU131943	10649	Human	Cambodia
DENV-3/IPC/BID-V4306/2007(3)	Dengue virus 3	GU131944	10656	Human	Cambodia
DENV-3/IPC/BID-V4308/2007(3)	Dengue virus 3	GU131945	10507	Human	Cambodia
DENV-3/IPC/BID-V4314/2008(3)	Dengue virus 3	GU131946	10631	Human	Cambodia
DENV-3/KBPV-VR-30-3	Dengue virus 3	KP406805	10696	Unknown	South Korea
DENV-3/KH/BID-V2050/2007(3)	Dengue virus 3	FJ639712	10643	Human	Cambodia
DENV-3/KH/BID-V2051/2007(3)	Dengue virus 3	FJ639713	10648	Human	Cambodia
DENV-3/KH/BID-V2052/2007(3)	Dengue virus 3	FJ639714	10649	Human	Cambodia
DENV-3/KH/BID-V2053/2008(3)	Dengue virus 3	FJ639715	10648	Human	Cambodia
DENV-3/KH/BID-V2054/2008(3)	Dengue virus 3	FJ639716	10648	Human	Cambodia
DENV-3/KH/BID-V2070/1999	Dengue virus 3	KF955461	10668	Human	Cambodia
DENV-3/KH/BID-V2071/2000(3)	Dengue virus 3	FJ639719	10648	Human	Cambodia
DENV-3/KH/BID-V2072/2000	Dengue virus 3	KF955332	10688	Human	Cambodia
DENV-3/KH/BID-V2073/2001(3)	Dengue virus 3	FJ639720	10648	Human	Cambodia
DENV-3/KH/BID-V2074/2001	Dengue virus 3	KF955462	10810	Human	Cambodia
DENV-3/KH/BID-V2075/2001(3)	Dengue virus 3	GQ868626	10663	Human	Cambodia
DENV-3/KH/BID-V2076/2001	Dengue virus 3	KF955463	10525	Human	Cambodia
DENV-3/KH/BID-V2077/2002(3)	Dengue virus 3	GQ868627	10663	Human	Cambodia
DENV-3/KH/BID-V2078/2002(3)	Dengue virus 3	FJ639721	10648	Human	Cambodia
DENV-3/KH/BID-V2079/2002(3)	Dengue virus 3	FJ639722	10648	Human	Cambodia
DENV-3/KH/BID-V2080/2003(3)	Dengue virus 3	FJ639723	10648	Human	Cambodia
DENV-3/KH/BID-V2081/2003(3)	Dengue virus 3	FJ639724	10648	Human	Cambodia
DENV-3/KH/BID-V2082/2003(3)	Dengue virus 3	FJ639725	10648	Human	Cambodia
DENV-3/KH/BID-V2083/2004(3)	Dengue virus 3	FJ639726	10648	Human	Cambodia

DENV-3/KH/BID-V2084/2005	Dengue virus 3	KF955333	10502	Human	Cambodia
DENV-3/KH/BID-V2085/2005(3)	Dengue virus 3	GQ868628	10659	Human	Cambodia
DENV-3/KH/BID-V2086/2005(3)	Dengue virus 3	FJ639727	10648	Human	Cambodia
DENV-3/KH/BID-V2087/2005(3)	Dengue virus 3	GQ868629	10660	Human	Cambodia
DENV-3/KH/BID-V2088/2005(3)	Dengue virus 3	FJ639728	10648	Human	Cambodia
DENV-3/KH/BID-V2089/2006(3)	Dengue virus 3	FJ639729	10648	Human	Cambodia
DENV-3/KH/BID-V2090/2006(3)	Dengue virus 3	FJ639730	10648	Human	Cambodia
DENV-3/KH/BID-V2091/2007	Dengue virus 3	KF955464	10525	Human	Cambodia
DENV-3/KH/BID-V2092/2007(3)	Dengue virus 3	FJ639731	10648	Human	Cambodia
DENV-3/KH/BID-V3816/2006	Dengue virus 3	HM181933	10525	Human	Cambodia
DENV-3/KH/BID-V3823/2006	Dengue virus 3	HM181934	10525	Human	Cambodia
DENV-3/KH/BID-V3829/2007	Dengue virus 3	HM181935	10525	Human	Cambodia
DENV-3/KH/BID-V4292/2007	Dengue virus 3	KF955507	10642	Human	Cambodia
DENV-3/KH/BID-V4307/2007-3	Dengue virus 3	JF295012	10510	Human	Cambodia
DENV-3/KH/BID-V4315/2008	Dengue virus 3	HM631854	10665	Human	Cambodia
DENV-3/LC/BID-V2979/2001(3)	Dengue virus 3	FJ898463	10660	Human	Saint Lucia
DENV-3/LC/BID-V3929/2001(3)	Dengue virus 3	GQ868616	10663	Human	Saint Lucia
DENV-3/LK/BID-V2404/1989	Dengue virus 3	KF955474	10663	Human	Sri Lanka
DENV-3/LK/BID-V2405/1983(3)	Dengue virus 3	GQ199887	10663	Human	Sri Lanka
DENV-3/LK/BID-V2407/1983(3)	Dengue virus 3	GQ199888	10663	Human	Sri Lanka
DENV-3/LK/BID-V2409/1997(3)	Dengue virus 3	GQ252674	10682	Human	Sri Lanka
DENV-3/LK/BID-V2410/1983(3)	Dengue virus 3	GQ199889	10645	Human	Sri Lanka
DENV-3/LK/BID-V2411/1989(3)	Dengue virus 3	FJ882571	10644	Human	Sri Lanka
DENV-3/LK/BID-V2412/1989(3)	Dengue virus 3	FJ882572	10663	Human	Sri Lanka
DENV-3/LK/BID-V2413/1993(3)	Dengue virus 3	FJ882573	10663	Human	Sri Lanka
DENV-3/LK/BID-V2414/1985(3)	Dengue virus 3	FJ882574	10651	Human	Sri Lanka
DENV-3/LK/BID-V2415/1983	Dengue virus 3	KF955476	10462	Human	Sri Lanka
DENV-3/MX/BID-V2985/2003(3)	Dengue virus 3	FJ898440	10663	Human	Mexico
DENV-3/MX/BID-V2987/2006(3)	Dengue virus 3	FJ898441	10663	Human	Mexico
DENV-3/MX/BID-V2989/2007(3)	Dengue virus 3	FJ898442	10663	Human	Mexico
DENV-3/MZ/BID-V2418/1985(3)	Dengue virus 3	FJ882575	10663	Human	Mozambique
DENV-3/NI/BID-V2419/1998(3)	Dengue virus 3	GQ199886	10646	Human	Nicaragua
DENV-3/NI/BID-V2420/1994(3)	Dengue virus 3	FJ882576	10663	Human	Nicaragua
DENV-3/NI/BID-V2644/2008(3)	Dengue virus 3	FJ850052	10652	Human	Nicaragua
DENV-3/NI/BID-V2647/2008(3)	Dengue virus 3	FJ873812	10638	Human	Nicaragua
DENV-3/NI/BID-V2649/2008(3)	Dengue virus 3	FJ873813	10637	Human	Nicaragua
DENV-3/NI/BID-V2653/2008(3)	Dengue virus 3	FJ850048	10652	Human	Nicaragua
DENV-3/NI/BID-V2654/2008(3)	Dengue virus 3	FJ850049	10652	Human	Nicaragua
DENV-3/NI/BID-V2934/2008(3)	Dengue virus 3	FJ898476	10639	Human	Nicaragua
DENV-3/NI/BID-V2935/2008(3)	Dengue virus 3	FJ898475	10652	Human	Nicaragua
DENV-3/NI/BID-V3055/2008(3)	Dengue virus 3	GQ199860	10652	Human	Nicaragua
DENV-3/NI/BID-V3057/2008(3)	Dengue virus 3	GQ199861	10652	Human	Nicaragua
DENV-3/NI/BID-V3059/2008(3)	Dengue virus 3	GQ199862	10652	Human	Nicaragua
DENV-3/NI/BID-V3066/2008(3)	Dengue virus 3	GQ199863	10652	Human	Nicaragua
DENV-3/NI/BID-V3068/2008(3)	Dengue virus 3	GQ199864	10652	Human	Nicaragua
DENV-3/NI/BID-V3069/2009(3)	Dengue virus 3	GQ199865	10652	Human	Nicaragua
DENV-3/NI/BID-V3072/2008(3)	Dengue virus 3	GQ199870	10652	Human	Nicaragua
DENV-3/NI/BID-V3073/2008(3)	Dengue virus 3	GQ199871	10637	Human	Nicaragua
DENV-3/NI/BID-V3163/2008	Dengue virus 3	HQ541789	10718	Human	Nicaragua
DENV-3/NI/BID-V3169/2008	Dengue virus 3	HQ541790	10694	Human	Nicaragua
DENV-3/NI/BID-V3175/2008	Dengue virus 3	HQ541791	10708	Human	Nicaragua
DENV-3/NI/BID-V4732/2009(3)	Dengue virus 3	HQ166031	10539	Human	Nicaragua
DENV-3/NI/BID-V4733/2009(3)	Dengue virus 3	HQ166032	10538	Human	Nicaragua
DENV-3/NI/BID-V4734/2009(3)	Dengue virus 3	HQ166033	10539	Human	Nicaragua
DENV-3/NI/BID-V4735/2009-3	Dengue virus 3	JF937630	10381	Human	Nicaragua
DENV-3/NI/BID-V4738/2009(3)	Dengue virus 3	HQ166030	10540	Human	Nicaragua
DENV-3/NI/BID-V4739/2009-3	Dengue virus 3	JN000936	10537	Human	Nicaragua
DENV-3/NI/BID-V4740/2009	Dengue virus 3	HQ541795	10539	Human	Nicaragua
DENV-3/NI/BID-V4742/2009	Dengue virus 3	HQ541796	10539	Human	Nicaragua
DENV-3/NI/BID-V4743/2009(3)	Dengue virus 3	HQ166034	10538	Human	Nicaragua
DENV-3/NI/BID-V4744/2009	Dengue virus 3	JN093517	10310	Human	Nicaragua
DENV-3/NI/BID-V4746/2009-3	Dengue virus 3	JF937631	10583	Human	Nicaragua
DENV-3/NI/BID-V4747/2009	Dengue virus 3	HM756274	10537	Human	Nicaragua
DENV-3/NI/BID-V4748/2009	Dengue virus 3	HM756275	10531	Human	Nicaragua
DENV-3/NI/BID-V4750/2009-3	Dengue virus 3	JF937632	10581	Human	Nicaragua
DENV-3/NI/BID-V4751/2009	Dengue virus 3	HQ671178	10583	Human	Nicaragua
DENV-3/NI/BID-V4752/2009	Dengue virus 3	HM756281	10540	Human	Nicaragua
DENV-3/NI/BID-V4753/2009	Dengue virus 3	HQ541806	10627	Human	Nicaragua
DENV-3/NI/BID-V4754/2009	Dengue virus 3	JN093514	10580	Human	Nicaragua
DENV-3/NI/BID-V4755/2009-3	Dengue virus 3	JF937643	10538	Human	Nicaragua
DENV-3/NI/BID-V4757/2009	Dengue virus 3	HM631869	10539	Human	Nicaragua
DENV-3/NI/BID-V4759/2009	Dengue virus 3	HM756282	10539	Human	Nicaragua
DENV-3/NI/BID-V4761/2009	Dengue virus 3	HM181972	10539	Human	Nicaragua
DENV-3/NI/BID-V4762/2009	Dengue virus 3	HQ541797	10565	Human	Nicaragua
DENV-3/NI/BID-V4763/2009	Dengue virus 3	HM631857	10539	Human	Nicaragua
DENV-3/NI/BID-V4765/2009	Dengue virus 3	HM631860	10538	Human	Nicaragua
DENV-3/NI/BID-V4766/2009	Dengue virus 3	HM181976	10539	Human	Nicaragua
DENV-3/NI/BID-V4767/2009	Dengue virus 3	HM631862	10539	Human	Nicaragua
DENV-3/NI/BID-V4768/2009	Dengue virus 3	HM631863	10540	Human	Nicaragua
DENV-3/NI/BID-V4774/2009	Dengue virus 3	HM181977	10536	Human	Nicaragua
DENV-3/NI/BID-V4776/2009	Dengue virus 3	HM631858	10539	Human	Nicaragua
DENV-3/NI/BID-V4778/2009	Dengue virus 3	HM181973	10538	Human	Nicaragua
DENV-3/NI/BID-V4780/2009	Dengue virus 3	HM181978	10566	Human	Nicaragua
DENV-3/NI/BID-V4782/2009	Dengue virus 3	JN183884	10539	Human	Nicaragua
DENV-3/NI/BID-V4783/2009	Dengue virus 3	HM181974	10540	Human	Nicaragua
DENV-3/NI/BID-V4786/2009	Dengue virus 3	HQ541785	10539	Human	Nicaragua
DENV-3/NI/BID-V4787/2009	Dengue virus 3	KF921916	10534	Human	Nicaragua
DENV-3/NI/BID-V4788/2009	Dengue virus 3	HM181975	10539	Human	Nicaragua
DENV-3/NI/BID-V4790/2009	Dengue virus 3	HM631864	10539	Human	Nicaragua
DENV-3/NI/BID-V4793/2009	Dengue virus 3	HM631861	10534	Human	Nicaragua
DENV-3/NI/BID-V4795/2009	Dengue virus 3	HM631859	10539	Human	Nicaragua
DENV-3/NI/BID-V4796/2009	Dengue virus 3	HM631856	10495	Human	Nicaragua
DENV-3/NI/BID-V4809/2009	Dengue virus 3	HM756276	10539	Human	Nicaragua
DENV-3/NI/BID-V4811/2009	Dengue virus 3	HM756277	10540	Human	Nicaragua
DENV-3/NI/BID-V4813/2009	Dengue virus 3	HM756278	10541	Human	Nicaragua
DENV-3/NI/BID-V4816/2009	Dengue virus 3	HM756279	10539	Human	Nicaragua
DENV-3/NI/BID-V4820/2009	Dengue virus 3	HM756280	10540	Human	Nicaragua
DENV-3/NI/BID-V4829/2009-3	Dengue virus 3	HQ891025	10287	Human	Nicaragua
DENV-3/NI/BID-V4830/2009-3	Dengue virus 3	HQ671177	10681	Human	Nicaragua
DENV-3/NI/BID-V4831/2009	Dengue virus 3	HQ705617	10682	Human	Nicaragua
DENV-3/NI/BID-V4836/2009	Dengue virus 3	HQ705618	10680	Human	Nicaragua
DENV-3/NI/BID-V4838/2009	Dengue virus 3	HQ705619	10680	Human	Nicaragua
DENV-3/NI/BID-V4845/2009	Dengue virus 3	HQ705620	10681	Human	Nicaragua
DENV-3/NI/BID-V4848/2009	Dengue virus 3	HQ705621	10681	Human	Nicaragua
DENV-3/NI/BID-V4849/2009	Dengue virus 3	HQ705622	10681	Human	Nicaragua
DENV-3/NI/BID-V4850/2009	Dengue virus 3	HQ705623	10681	Human	Nicaragua
DENV-3/NI/BID-V4856/2009	Dengue virus 3	HQ671176	10681	Human	Nicaragua
DENV-3/NI/BID-V4859/2009	Dengue virus 3	HQ705609	10681	Human	Nicaragua
DENV-3/NI/BID-V4860/2009	Dengue virus 3	HQ705610	10681	Human	Nicaragua
DENV-3/NI/BID-V4861/2009	Dengue virus 3	HQ705611	10685	Human	Nicaragua
DENV-3/NI/BID-V4862/2009	Dengue virus 3	HQ705612	10681	Human	Nicaragua
DENV-3/NI/BID-V4866/2009	Dengue virus 3	HQ705613	10681	Human	Nicaragua
DENV-3/NI/BID-V4867/2009	Dengue virus 3	HQ705614	10681	Human	Nicaragua

DENV-3/NI/BID-V4871/2009	Dengue virus 3	HQ705615	10431	Human	Nicaragua
DENV-3/NI/BID-V4872/2009	Dengue virus 3	HQ705616	10682	Human	Nicaragua
DENV-3/NI/BID-V5095/2009	Dengue virus 3	JF920398	10522	Human	Nicaragua
DENV-3/NI/BID-V5097/2009	Dengue virus 3	JF920399	10681	Human	Nicaragua
DENV-3/NI/BID-V5099/2009	Dengue virus 3	JF920400	10681	Human	Nicaragua
DENV-3/NI/BID-V5101/2009	Dengue virus 3	JF920407	10680	Human	Nicaragua
DENV-3/NI/BID-V5441/2009	Dengue virus 3	JF920402	10680	Human	Nicaragua
DENV-3/NI/BID-V5442/2009	Dengue virus 3	JF920393	10681	Human	Nicaragua
DENV-3/NI/BID-V5443/2009-3	Dengue virus 3	JF937652	10477	Human	Nicaragua
DENV-3/NI/BID-V5445/2009	Dengue virus 3	JF920403	10535	Human	Nicaragua
DENV-3/NI/BID-V5449/2009	Dengue virus 3	JF920404	10681	Human	Nicaragua
DENV-3/NI/BID-V5452/2009	Dengue virus 3	JF920394	10682	Human	Nicaragua
DENV-3/NI/BID-V5453/2009	Dengue virus 3	KF971709	10678	Human	Nicaragua
DENV-3/NI/BID-V5455/2009-3	Dengue virus 3	JF937641	10681	Human	Nicaragua
DENV-3/NI/BID-V5457/2009	Dengue virus 3	JF920395	10605	Human	Nicaragua
DENV-3/NI/BID-V5461/2009	Dengue virus 3	JF920396	10675	Human	Nicaragua
DENV-3/NI/BID-V5464/2009-3	Dengue virus 3	JF937642	10681	Human	Nicaragua
DENV-3/NI/BID-V5467/2010	Dengue virus 3	JF920405	10681	Human	Nicaragua
DENV-3/NI/BID-V5468/2010	Dengue virus 3	JF920406	10681	Human	Nicaragua
DENV-3/NI/BID-V5471/2010	Dengue virus 3	JF920409	10680	Human	Nicaragua
DENV-3/NI/BID-V5474/2010	Dengue virus 3	JF920408	10681	Human	Nicaragua
DENV-3/NI/BID-V5477/2010-3	Dengue virus 3	JF937620	10681	Human	Nicaragua
DENV-3/NI/BID-V5478/2010	Dengue virus 3	JF920397	10676	Human	Nicaragua
DENV-3/NI/BID-V5482/2010	Dengue virus 3	JF920401	10681	Human	Nicaragua
DENV-3/NI/BID-V5486/2010-3	Dengue virus 3	JF937629	10683	Human	Nicaragua
DENV-3/NI/BID-V5488/2010	Dengue virus 3	JN093513	10690	Human	Nicaragua
DENV-3/NI/BID-V5489/2010-3	Dengue virus 3	JF937624	10681	Human	Nicaragua
DENV-3/NI/BID-V5491/2010-3	Dengue virus 3	JN000938	10685	Human	Nicaragua
DENV-3/NI/BID-V5492/2010	Dengue virus 3	JN093515	10679	Human	Nicaragua
DENV-3/NI/BID-V5494/2010-3	Dengue virus 3	JF937646	10681	Human	Nicaragua
DENV-3/NI/BID-V5495/2009-3	Dengue virus 3	JF937647	10682	Human	Nicaragua
DENV-3/NI/BID-V5496/2010-3	Dengue virus 3	JF937625	10683	Human	Nicaragua
DENV-3/NI/BID-V5498/2010-3	Dengue virus 3	JF937633	10683	Human	Nicaragua
DENV-3/NI/BID-V5499/2010-3	Dengue virus 3	JF937634	10681	Human	Nicaragua
DENV-3/NI/BID-V5506/2009-3	Dengue virus 3	JF937636	10670	Human	Nicaragua
DENV-3/NI/BID-V5507/2010-3	Dengue virus 3	JF937648	10680	Human	Nicaragua
DENV-3/NI/BID-V5508/2010-3	Dengue virus 3	JF937626	10691	Human	Nicaragua
DENV-3/NI/BID-V5511/2010-3	Dengue virus 3	JF937621	10676	Human	Nicaragua
DENV-3/NI/BID-V5512/2010-3	Dengue virus 3	JF937622	10680	Human	Nicaragua
DENV-3/NI/BID-V5514/2010-3	Dengue virus 3	JF937623	10681	Human	Nicaragua
DENV-3/NI/BID-V5516/2010-3	Dengue virus 3	JF937637	10689	Human	Nicaragua
DENV-3/NI/BID-V5517/2010-3	Dengue virus 3	JF937638	10681	Human	Nicaragua
DENV-3/NI/BID-V5680/2010-3	Dengue virus 3	JF937639	10644	Human	Nicaragua
DENV-3/NI/BID-V5681/2010-3	Dengue virus 3	JF937640	10678	Human	Nicaragua
DENV-3/NI/BID-V5684/2010-3	Dengue virus 3	JN000937	10398	Human	Nicaragua
DENV-3/NI/BID-V5685/2010-3	Dengue virus 3	JF937627	10527	Human	Nicaragua
DENV-3/NI/BID-V5686/2010-3	Dengue virus 3	JF937628	10684	Human	Nicaragua
DENV-3/NI/BID-V7634/2011	Dengue virus 3	KF973476	10696	Human	Nicaragua
DENV-3/NI/BID-V7637/2011	Dengue virus 3	KF973477	10512	Human	Nicaragua
DENV-3/NI/BID-V7646/2012	Dengue virus 3	KF973478	10584	Human	Nicaragua
DENV-3/NI/BID-V7657/2012	Dengue virus 3	KF973479	10569	Human	Nicaragua
DENV-3/NI/BID-V7658/2012	Dengue virus 3	KF973480	10569	Human	Nicaragua
DENV-3/NI/BID-V7662/2011	Dengue virus 3	KF973481	10644	Human	Nicaragua
DENV-3/NI/BID-V7663/2011	Dengue virus 3	KF973482	10584	Human	Nicaragua
DENV-3/NI/BID-V7665/2011	Dengue virus 3	KF973483	10509	Human	Nicaragua
DENV-3/NI/BID-V7668/2011	Dengue virus 3	KF973484	10578	Human	Nicaragua
DENV-3/NI/BID-V7674/2011	Dengue virus 3	KF973485	10604	Human	Nicaragua
DENV-3/NI/BID-V7694/2012	Dengue virus 3	KF973486	10576	Human	Nicaragua
DENV-3/NI/BID-V7699/2011	Dengue virus 3	KF973487	10569	Human	Nicaragua
DENV-3/PE/BID-V2981/2002(3)	Dengue virus 3	FJ898458	10663	Human	Peru
DENV-3/PE/BID-V6158/2002	Dengue virus 3	KJ189256	10693	Human	Peru
DENV-3/PE/BID-V6159/2004	Dengue virus 3	KJ189257	10693	Human	Peru
DENV-3/PE/BID-V6167/2002	Dengue virus 3	KJ189258	10692	Human	Peru
DENV-3/PE/BID-V6168/2002	Dengue virus 3	KJ189259	10692	Human	Peru
DENV-3/PE/BID-V6170/2002	Dengue virus 3	KJ189260	10692	Human	Peru
DENV-3/PE/BID-V6262/2007	Dengue virus 3	KJ643590	10692	Human	Peru
DENV-3/PE/BID-V6263/2008	Dengue virus 3	KJ189261	10685	Human	Peru
DENV-3/PE/BID-V7041/2004	Dengue virus 3	KJ189262	10692	Human	Peru
DENV-3/PE/BID-V7042/2004	Dengue virus 3	KJ189263	10693	Human	Peru
DENV-3/PE/BID-V7043/2004	Dengue virus 3	KJ189264	10693	Human	Peru
DENV-3/PE/BID-V7046/2004	Dengue virus 3	KJ189265	10693	Human	Peru
DENV-3/PE/BID-V7048/2004	Dengue virus 3	KJ189266	10696	Human	Peru
DENV-3/PE/BID-V7050/2007	Dengue virus 3	KJ189267	10693	Human	Peru
DENV-3/PE/BID-V7051/2007	Dengue virus 3	KJ189268	10692	Human	Peru
DENV-3/PE/BID-V7052/2007	Dengue virus 3	KJ189269	10693	Human	Peru
DENV-3/PE/BID-V7053/2007	Dengue virus 3	KJ189270	10693	Human	Peru
DENV-3/PE/BID-V7055/2007	Dengue virus 3	KJ189271	10692	Human	Peru
DENV-3/PE/BID-V7056/2007	Dengue virus 3	KJ189272	10693	Human	Peru
DENV-3/PE/BID-V7057/2007	Dengue virus 3	KJ189273	10693	Human	Peru
DENV-3/PE/BID-V7058/2007	Dengue virus 3	KJ189274	10695	Human	Peru
DENV-3/PE/BID-V7059/2007	Dengue virus 3	KJ189275	10698	Human	Peru
DENV-3/PE/BID-V7060/2007	Dengue virus 3	KJ189276	10693	Human	Peru
DENV-3/PE/BID-V7061/2007	Dengue virus 3	KJ189277	10693	Human	Peru
DENV-3/PE/BID-V7062/2007	Dengue virus 3	KJ189278	10694	Human	Peru
DENV-3/PE/BID-V7063/2007	Dengue virus 3	KJ189279	10692	Human	Peru
DENV-3/PE/BID-V7064/2007	Dengue virus 3	KJ189280	10692	Human	Peru
DENV-3/PE/BID-V7065/2007	Dengue virus 3	KJ189281	10692	Human	Peru
DENV-3/PE/BID-V7066/2007	Dengue virus 3	KJ189282	10695	Human	Peru
DENV-3/PE/BID-V7067/2007	Dengue virus 3	KJ189283	10693	Human	Peru
DENV-3/PE/BID-V7068/2008	Dengue virus 3	KJ189284	10693	Human	Peru
DENV-3/PE/BID-V7069/2008	Dengue virus 3	KJ189285	10692	Human	Peru
DENV-3/PE/BID-V7070/2008	Dengue virus 3	KJ189286	10697	Human	Peru
DENV-3/PE/BID-V7071/2008	Dengue virus 3	KJ189287	10694	Human	Peru
DENV-3/PE/BID-V7073/2008	Dengue virus 3	KJ189288	10697	Human	Peru
DENV-3/PE/BID-V7074/2008	Dengue virus 3	KJ189289	10692	Human	Peru
DENV-3/PE/BID-V7078/2008	Dengue virus 3	KJ189290	10692	Human	Peru
DENV-3/PE/BID-V7079/2008	Dengue virus 3	KJ189291	10692	Human	Peru
DENV-3/PE/BID-V7081/2009	Dengue virus 3	KJ189292	10696	Human	Peru
DENV-3/PE/BID-V7082/2005	Dengue virus 3	KJ189293	10693	Human	Peru
DENV-3/PE/BID-V7083/2006	Dengue virus 3	KJ189294	10692	Human	Peru
DENV-3/PE/BID-V7084/2006	Dengue virus 3	KJ189295	10694	Human	Peru
DENV-3/PE/BID-V7085/2006	Dengue virus 3	KJ189296	10697	Human	Peru
DENV-3/PE/BID-V7086/2006	Dengue virus 3	KJ189297	10692	Human	Peru
DENV-3/PE/BID-V7087/2008	Dengue virus 3	KJ189298	10692	Human	Peru
DENV-3/PE/BID-V7088/2005	Dengue virus 3	KJ189299	10692	Human	Peru
DENV-3/PE/BID-V7090/2008	Dengue virus 3	KJ189300	10692	Human	Peru
DENV-3/PE/BID-V7289/2008	Dengue virus 3	KJ189301	10693	Human	Peru
DENV-3/PR/BID-V1728/2006	Dengue virus 3	KF955456	10645	Human	Puerto Rico
DENV-3/PR/BID-V2101/2000	Dengue virus 3	KF955465	10614	Human	Puerto Rico
DENV-3/PR/BID-V2102/2000	Dengue virus 3	KF955466	10596	Human	Puerto Rico
DENV-3/PR/BID-V2116/2001	Dengue virus 3	KF955468	10640	Human	Puerto Rico
DENV-3/TH/BID-V2312/2001(3)	Dengue virus 3	FJ744726	10649	Human	Thailand
DENV-3/TH/BID-V2313/2001(3)	Dengue virus 3	FJ744727	10665	Human	Thailand

DENV-3/TH/BID-V2314/2001(3)	Dengue virus 3	FJ744728	10664	Human	Thailand
DENV-3/TH/BID-V2315/2001(3)	Dengue virus 3	FJ744729	10663	Human	Thailand
DENV-3/TH/BID-V2316/2001(3)	Dengue virus 3	FJ744730	10663	Human	Thailand
DENV-3/TH/BID-V2317/2001(3)	Dengue virus 3	FJ744731	10663	Human	Thailand
DENV-3/TH/BID-V2318/2001(3)	Dengue virus 3	FJ687448	10629	Human	Thailand
DENV-3/TH/BID-V2319/2001(3)	Dengue virus 3	FJ810413	10664	Human	Thailand
DENV-3/TH/BID-V2320/2001(3)	Dengue virus 3	FJ744732	10648	Human	Thailand
DENV-3/TH/BID-V2321/2001(3)	Dengue virus 3	FJ744733	10663	Human	Thailand
DENV-3/TH/BID-V2322/2001(3)	Dengue virus 3	FJ810414	10663	Human	Thailand
DENV-3/TH/BID-V2323/2001(3)	Dengue virus 3	FJ744734	10663	Human	Thailand
DENV-3/TH/BID-V2324/2001(3)	Dengue virus 3	FJ744735	10645	Human	Thailand
DENV-3/TH/BID-V2325/2001(3)	Dengue virus 3	FJ744736	10663	Human	Thailand
DENV-3/TH/BID-V2326/2001(3)	Dengue virus 3	FJ744737	10661	Human	Thailand
DENV-3/TH/BID-V2327/2001(3)	Dengue virus 3	FJ744738	10651	Human	Thailand
DENV-3/TH/BID-V2328/2001(3)	Dengue virus 3	FJ744739	10630	Human	Thailand
DENV-3/TH/BID-V2329/2001(3)	Dengue virus 3	FJ744740	10648	Human	Thailand
DENV-3/TH/BID-V3360/1973(3)	Dengue virus 3	GQ868593	10663	Human	Thailand
DENV-3/TT/BID-V2982/2002(3)	Dengue virus 3	FJ898459	10663	Human	Trinidad and Tobago
DENV-3/TT/BID-V3928/2002(3)	Dengue virus 3	GQ868617	10662	Human	Trinidad and Tobago
DENV-3/US/BID-V1043/2006(3)	Dengue virus 3	EU482555	10648	Human	USA
DENV-3/US/BID-V1044/2006(3)	Dengue virus 3	EU529692	10648	Human	USA
DENV-3/US/BID-V1049/1998(3)	Dengue virus 3	EU482558	10648	Human	USA
DENV-3/US/BID-V1050/1998(3)	Dengue virus 3	EU482559	10648	Human	USA
DENV-3/US/BID-V1075/1998(3)	Dengue virus 3	EU482563	10648	Human	USA
DENV-3/US/BID-V1076/1999(3)	Dengue virus 3	EU529696	10651	Human	USA
DENV-3/US/BID-V1077/2000(3)	Dengue virus 3	EU529697	10648	Human	USA
DENV-3/US/BID-V1078/2003(3)	Dengue virus 3	EU482564	10648	Human	USA
DENV-3/US/BID-V1079/2006(3)	Dengue virus 3	EU529698	10648	Human	USA
DENV-3/US/BID-V1080/2006(3)	Dengue virus 3	EU529699	10648	Human	USA
DENV-3/US/BID-V1088/1998(3)	Dengue virus 3	EU482566	10648	Human	USA
DENV-3/US/BID-V1089/2003(3)	Dengue virus 3	EU529702	10649	Human	USA
DENV-3/US/BID-V1090/1998(3)	Dengue virus 3	EU529703	10649	Human	USA
DENV-3/US/BID-V1091/2004(3)	Dengue virus 3	EU529704	10649	Human	USA
DENV-3/US/BID-V1092/2004(3)	Dengue virus 3	EU529705	10648	Human	USA
DENV-3/US/BID-V1415/2007(3)	Dengue virus 3	EU596492	10648	Human	USA
DENV-3/US/BID-V1416/2007(3)	Dengue virus 3	EU596493	10648	Human	USA
DENV-3/US/BID-V1417/2007(3)	Dengue virus 3	EU596494	10648	Human	USA
DENV-3/US/BID-V1447/1998(3)	Dengue virus 3	EU687218	10648	Human	USA
DENV-3/US/BID-V1448/1998(3)	Dengue virus 3	EU726771	10648	Human	USA
DENV-3/US/BID-V1449/1998(3)	Dengue virus 3	EU726772	10648	Human	USA
DENV-3/US/BID-V1450/1998(3)	Dengue virus 3	FJ182013	10648	Human	USA
DENV-3/US/BID-V1451/1999(3)	Dengue virus 3	EU781136	10648	Human	USA
DENV-3/US/BID-V1452/1999(3)	Dengue virus 3	EU781137	10648	Human	USA
DENV-3/US/BID-V1453/1999(3)	Dengue virus 3	EU726773	10650	Human	USA
DENV-3/US/BID-V1454/1999(3)	Dengue virus 3	EU726774	10648	Human	USA
DENV-3/US/BID-V1455/1999(3)	Dengue virus 3	EU687219	10655	Human	USA
DENV-3/US/BID-V1460/2000(3)	Dengue virus 3	EU687221	10648	Human	USA
DENV-3/US/BID-V1465/2000(3)	Dengue virus 3	EU726768	10648	Human	USA
DENV-3/US/BID-V1466/1999(3)	Dengue virus 3	EU687226	10645	Human	USA
DENV-3/US/BID-V1473/2002(3)	Dengue virus 3	EU687233	10653	Human	USA
DENV-3/US/BID-V1475/2002(3)	Dengue virus 3	EU687234	10645	Human	USA
DENV-3/US/BID-V1476/2002(3)	Dengue virus 3	EU687196	10653	Human	USA
DENV-3/US/BID-V1477/2002(3)	Dengue virus 3	EU854298	10640	Human	USA
DENV-3/US/BID-V1478/2002(3)	Dengue virus 3	FJ373306	10648	Human	USA
DENV-3/US/BID-V1480/2003(3)	Dengue virus 3	EU687197	10659	Human	USA
DENV-3/US/BID-V1481/2003(3)	Dengue virus 3	EU687198	10675	Human	USA
DENV-3/US/BID-V1490/2003(3)	Dengue virus 3	EU726769	10648	Human	USA
DENV-3/US/BID-V1491/2003(3)	Dengue virus 3	EU687239	10648	Human	USA
DENV-3/US/BID-V1604/2004(3)	Dengue virus 3	FJ182004	10648	Human	USA
DENV-3/US/BID-V1605/2004(3)	Dengue virus 3	FJ850055	10648	Human	USA
DENV-3/US/BID-V1606/2004(3)	Dengue virus 3	FJ024465	10648	Human	USA
DENV-3/US/BID-V1607/2004(3)	Dengue virus 3	FJ024466	10650	Human	USA
DENV-3/US/BID-V1608/2004(3)	Dengue virus 3	FJ024467	10645	Human	USA
DENV-3/US/BID-V1609/2004(3)	Dengue virus 3	FJ024468	10648	Human	USA
DENV-3/US/BID-V1610/2004(3)	Dengue virus 3	FJ024469	10648	Human	USA
DENV-3/US/BID-V1611/2004(3)	Dengue virus 3	FJ850056	10649	Human	USA
DENV-3/US/BID-V1612/2004(3)	Dengue virus 3	FJ024470	10626	Human	USA
DENV-3/US/BID-V1613/2004(3)	Dengue virus 3	FJ024471	10649	Human	USA
DENV-3/US/BID-V1614/2004(3)	Dengue virus 3	FJ182005	10650	Human	USA
DENV-3/US/BID-V1615/2004(3)	Dengue virus 3	FJ373302	10652	Human	USA
DENV-3/US/BID-V1616/2004(3)	Dengue virus 3	FJ182006	10648	Human	USA
DENV-3/US/BID-V1617/2005(3)	Dengue virus 3	FJ182007	10648	Human	USA
DENV-3/US/BID-V1618/2005(3)	Dengue virus 3	FJ182008	10648	Human	USA
DENV-3/US/BID-V1619/2005(3)	Dengue virus 3	FJ182009	10648	Human	USA
DENV-3/US/BID-V1620/2005(3)	Dengue virus 3	FJ182010	10648	Human	USA
DENV-3/US/BID-V1621/2005(3)	Dengue virus 3	FJ182011	10650	Human	USA
DENV-3/US/BID-V1622/2005(3)	Dengue virus 3	FJ182037	10648	Human	USA
DENV-3/US/BID-V1623/2005(3)	Dengue virus 3	FJ182038	10648	Human	USA
DENV-3/US/BID-V1624/2005(3)	Dengue virus 3	FJ182039	10648	Human	USA
DENV-3/US/BID-V1625/2005(3)	Dengue virus 3	FJ182040	10652	Human	USA
DENV-3/US/BID-V1626/2005(3)	Dengue virus 3	FJ182041	10648	Human	USA
DENV-3/US/BID-V1729/2003(3)	Dengue virus 3	FJ390371	10648	Human	USA
DENV-3/US/BID-V1730/2003(3)	Dengue virus 3	FJ390372	10655	Human	USA
DENV-3/US/BID-V1731/2003(3)	Dengue virus 3	FJ205870	10648	Human	USA
DENV-3/US/BID-V1732/2002(3)	Dengue virus 3	FJ390373	10648	Human	USA
DENV-3/US/BID-V1733/1999(3)	Dengue virus 3	FJ205871	10651	Human	USA
DENV-3/US/BID-V1735/1999(3)	Dengue virus 3	FJ390375	10650	Human	USA
DENV-3/US/BID-V1736/1999(3)	Dengue virus 3	FJ390376	10669	Human	USA
DENV-3/US/BID-V1737/1999(3)	Dengue virus 3	FJ390377	10650	Human	USA
DENV-3/US/BID-V2098/1999(3)	Dengue virus 3	FJ547069	10648	Human	USA
DENV-3/US/BID-V2099/1998(3)	Dengue virus 3	FJ547070	10648	Human	USA
DENV-3/US/BID-V2100/2000(3)	Dengue virus 3	FJ562107	10648	Human	USA
DENV-3/US/BID-V2103/2000(3)	Dengue virus 3	FJ547071	10648	Human	USA
DENV-3/US/BID-V2104/2000(3)	Dengue virus 3	FJ410176	10645	Human	USA
DENV-3/US/BID-V2105/2000(3)	Dengue virus 3	FJ410177	10648	Human	USA
DENV-3/US/BID-V2106/2000(3)	Dengue virus 3	FJ547072	10649	Human	USA
DENV-3/US/BID-V2107/2000(3)	Dengue virus 3	FJ547073	10648	Human	USA
DENV-3/US/BID-V2108/2000(3)	Dengue virus 3	FJ547074	10648	Human	USA
DENV-3/US/BID-V2110/2000(3)	Dengue virus 3	FJ547075	10648	Human	USA
DENV-3/US/BID-V2111/2000(3)	Dengue virus 3	FJ547076	10648	Human	USA
DENV-3/US/BID-V2112/2000(3)	Dengue virus 3	FJ547077	10648	Human	USA
DENV-3/US/BID-V2113/2000(3)	Dengue virus 3	FJ547078	10648	Human	USA
DENV-3/US/BID-V2114/2001(3)	Dengue virus 3	FJ547079	10648	Human	USA
DENV-3/US/BID-V2115/2001(3)	Dengue virus 3	FJ547080	10648	Human	USA
DENV-3/US/BID-V2117/2001(3)	Dengue virus 3	FJ547081	10648	Human	USA
DENV-3/US/BID-V2118/2001(3)	Dengue virus 3	FJ547082	10648	Human	USA
DENV-3/US/BID-V2119/2002(3)	Dengue virus 3	FJ547083	10649	Human	USA
DENV-3/US/BID-V2120/2002(3)	Dengue virus 3	FJ410178	10647	Human	USA
DENV-3/US/BID-V2122/2002(3)	Dengue virus 3	FJ547084	10648	Human	USA
DENV-3/US/BID-V2123/2002(3)	Dengue virus 3	FJ478456	10648	Human	USA
DENV-3/US/BID-V2126/2006(3)	Dengue virus 3	FJ547085	10648	Human	USA
DENV-3/US/BID-V858/2003(3)	Dengue virus 3	EU482595	10663	Human	USA
DENV-3/US/BID-V859/1998(3)	Dengue virus 3	EU482596	10663	Human	USA

DENV-3/USA/633798/1963-3	Dengue virus 3	JQ922554	10654	Human	USA
DENV-3/VE/BID-V1102/2007(3)	Dengue virus 3	EU529683	10649	Human	Venezuela
DENV-3/VE/BID-V1113/2001(3)	Dengue virus 3	EU529684	10662	Human	Venezuela
DENV-3/VE/BID-V1114/2001(3)	Dengue virus 3	FJ182015	10648	Human	Venezuela
DENV-3/VE/BID-V1115/2001(3)	Dengue virus 3	EU569688	10648	Human	Venezuela
DENV-3/VE/BID-V1116/2001(3)	Dengue virus 3	EU529685	10648	Human	Venezuela
DENV-3/VE/BID-V1117/2001(3)	Dengue virus 3	EU529686	10648	Human	Venezuela
DENV-3/VE/BID-V1118/2001(3)	Dengue virus 3	EU529687	10648	Human	Venezuela
DENV-3/VE/BID-V1121/36892.5	Dengue virus 3	KF955449	10421	Human	Venezuela
DENV-3/VE/BID-V1149/2007(3)	Dengue virus 3	EU932687	10664	Human	Venezuela
DENV-3/VE/BID-V1150/2007(3)	Dengue virus 3	EU932688	10663	Human	Venezuela
DENV-3/VE/BID-V1585/2001(3)	Dengue virus 3	FJ373303	10648	Human	Venezuela
DENV-3/VE/BID-V1586/37622.5	Dengue virus 3	KF955451	10507	Human	Venezuela
DENV-3/VE/BID-V1589/37987.5	Dengue virus 3	KF955453	10511	Human	Venezuela
DENV-3/VE/BID-V1590/2004(3)	Dengue virus 3	FJ373304	10653	Human	Venezuela
DENV-3/VE/BID-V1591/2004(3)	Dengue virus 3	EU854291	10648	Human	Venezuela
DENV-3/VE/BID-V1592/37987.5	Dengue virus 3	KF955454	10510	Human	Venezuela
DENV-3/VE/BID-V1593/2005(3)	Dengue virus 3	EU854292	10648	Human	Venezuela
DENV-3/VE/BID-V2174/2000(3)	Dengue virus 3	FJ639746	10648	Human	Venezuela
DENV-3/VE/BID-V2175/2000(3)	Dengue virus 3	FJ639747	10648	Human	Venezuela
DENV-3/VE/BID-V2178/2000(3)	Dengue virus 3	FJ639749	10647	Human	Venezuela
DENV-3/VE/BID-V2179/2000(3)	Dengue virus 3	FJ639750	10648	Human	Venezuela
DENV-3/VE/BID-V2180/2001(3)	Dengue virus 3	FJ639751	10632	Human	Venezuela
DENV-3/VE/BID-V2181/2001(3)	Dengue virus 3	FJ639752	10648	Human	Venezuela
DENV-3/VE/BID-V2182/2001(3)	Dengue virus 3	FJ639753	10647	Human	Venezuela
DENV-3/VE/BID-V2183/2001(3)	Dengue virus 3	FJ639754	10648	Human	Venezuela
DENV-3/VE/BID-V2184/2001(3)	Dengue virus 3	FJ639755	10635	Human	Venezuela
DENV-3/VE/BID-V2185/2001(3)	Dengue virus 3	FJ639756	10648	Human	Venezuela
DENV-3/VE/BID-V2186/2001(3)	Dengue virus 3	FJ744700	10648	Human	Venezuela
DENV-3/VE/BID-V2187/2001(3)	Dengue virus 3	FJ639757	10627	Human	Venezuela
DENV-3/VE/BID-V2188/2001(3)	Dengue virus 3	FJ639758	10648	Human	Venezuela
DENV-3/VE/BID-V2189/2001(3)	Dengue virus 3	FJ639759	10648	Human	Venezuela
DENV-3/VE/BID-V2190/2001(3)	Dengue virus 3	FJ639760	10649	Human	Venezuela
DENV-3/VE/BID-V2191/2001(3)	Dengue virus 3	FJ639761	10648	Human	Venezuela
DENV-3/VE/BID-V2192/2001(3)	Dengue virus 3	FJ639762	10651	Human	Venezuela
DENV-3/VE/BID-V2193/2001(3)	Dengue virus 3	FJ639763	10648	Human	Venezuela
DENV-3/VE/BID-V2195/2001(3)	Dengue virus 3	FJ639765	10648	Human	Venezuela
DENV-3/VE/BID-V2196/2001(3)	Dengue virus 3	FJ639766	10648	Human	Venezuela
DENV-3/VE/BID-V2197/2001(3)	Dengue virus 3	FJ639767	10628	Human	Venezuela
DENV-3/VE/BID-V2198/2001(3)	Dengue virus 3	FJ639768	10648	Human	Venezuela
DENV-3/VE/BID-V2199/2001(3)	Dengue virus 3	FJ639769	10647	Human	Venezuela
DENV-3/VE/BID-V2200/2001(3)	Dengue virus 3	FJ882577	10661	Human	Venezuela
DENV-3/VE/BID-V2201/2001(3)	Dengue virus 3	FJ850096	10648	Human	Venezuela
DENV-3/VE/BID-V2202/2001(3)	Dengue virus 3	FJ882578	10663	Human	Venezuela
DENV-3/VE/BID-V2203/2001(3)	Dengue virus 3	FJ639770	10648	Human	Venezuela
DENV-3/VE/BID-V2204/2001(3)	Dengue virus 3	FJ639771	10647	Human	Venezuela
DENV-3/VE/BID-V2205/2007(3)	Dengue virus 3	FJ639772	10648	Human	Venezuela
DENV-3/VE/BID-V2207/2001(3)	Dengue virus 3	FJ639774	10647	Human	Venezuela
DENV-3/VE/BID-V2208/2002(3)	Dengue virus 3	FJ639775	10629	Human	Venezuela
DENV-3/VE/BID-V2209/2002(3)	Dengue virus 3	FJ639776	10648	Human	Venezuela
DENV-3/VE/BID-V2210/2002(3)	Dengue virus 3	FJ639777	10638	Human	Venezuela
DENV-3/VE/BID-V2211/2002(3)	Dengue virus 3	FJ639778	10649	Human	Venezuela
DENV-3/VE/BID-V2212/2003(3)	Dengue virus 3	FJ639779	10633	Human	Venezuela
DENV-3/VE/BID-V2213/2003(3)	Dengue virus 3	FJ639780	10649	Human	Venezuela
DENV-3/VE/BID-V2214/2003(3)	Dengue virus 3	FJ639781	10648	Human	Venezuela
DENV-3/VE/BID-V2215/2003(3)	Dengue virus 3	FJ639782	10654	Human	Venezuela
DENV-3/VE/BID-V2217/2003(3)	Dengue virus 3	FJ639784	10648	Human	Venezuela
DENV-3/VE/BID-V2218/2003(3)	Dengue virus 3	FJ639785	10648	Human	Venezuela
DENV-3/VE/BID-V2219/2003(3)	Dengue virus 3	FJ639786	10654	Human	Venezuela
DENV-3/VE/BID-V2220/2004(3)	Dengue virus 3	FJ639787	10648	Human	Venezuela
DENV-3/VE/BID-V2222/2004(3)	Dengue virus 3	FJ639789	10650	Human	Venezuela
DENV-3/VE/BID-V2223/2004(3)	Dengue virus 3	FJ639790	10654	Human	Venezuela
DENV-3/VE/BID-V2224/2004(3)	Dengue virus 3	FJ639791	10647	Human	Venezuela
DENV-3/VE/BID-V2225/2004(3)	Dengue virus 3	FJ639792	10636	Human	Venezuela
DENV-3/VE/BID-V2226/2004(3)	Dengue virus 3	FJ639793	10648	Human	Venezuela
DENV-3/VE/BID-V2228/2004(3)	Dengue virus 3	FJ639795	10647	Human	Venezuela
DENV-3/VE/BID-V2231/2004(3)	Dengue virus 3	FJ639798	10648	Human	Venezuela
DENV-3/VE/BID-V2232/2004(3)	Dengue virus 3	FJ639799	10653	Human	Venezuela
DENV-3/VE/BID-V2233/2004(3)	Dengue virus 3	FJ639800	10648	Human	Venezuela
DENV-3/VE/BID-V2234/2004(3)	Dengue virus 3	FJ639801	10648	Human	Venezuela
DENV-3/VE/BID-V2236/2004	Dengue virus 3	KF955471	10525	Human	Venezuela
DENV-3/VE/BID-V2238/2004	Dengue virus 3	KF955472	10639	Human	Venezuela
DENV-3/VE/BID-V2239/2005(3)	Dengue virus 3	FJ639803	10648	Human	Venezuela
DENV-3/VE/BID-V2240/2005(3)	Dengue virus 3	FJ639804	10648	Human	Venezuela
DENV-3/VE/BID-V2242/2005(3)	Dengue virus 3	FJ639805	10626	Human	Venezuela
DENV-3/VE/BID-V2244/2005(3)	Dengue virus 3	FJ639807	10648	Human	Venezuela
DENV-3/VE/BID-V2247/2005(3)	Dengue virus 3	FJ639810	10648	Human	Venezuela
DENV-3/VE/BID-V2256/2005(3)	Dengue virus 3	FJ639816	10648	Human	Venezuela
DENV-3/VE/BID-V2257/2006(3)	Dengue virus 3	FJ639817	10649	Human	Venezuela
DENV-3/VE/BID-V2266/2006(3)	Dengue virus 3	FJ639825	10648	Human	Venezuela
DENV-3/VE/BID-V2267/2008(3)	Dengue virus 3	FJ639826	10654	Human	Venezuela
DENV-3/VE/BID-V2268/2008(3)	Dengue virus 3	FJ639827	10635	Human	Venezuela
DENV-3/VE/BID-V2452/2001(3)	Dengue virus 3	FJ850097	10663	Human	Venezuela
DENV-3/VE/BID-V2453/2001(3)	Dengue virus 3	FJ810416	10649	Human	Venezuela
DENV-3/VE/BID-V2454/2001(3)	Dengue virus 3	GQ252678	10641	Human	Venezuela
DENV-3/VE/BID-V2455/2001(3)	Dengue virus 3	FJ850098	10650	Human	Venezuela
DENV-3/VE/BID-V2456/2001	Dengue virus 3	KF955479	10638	Human	Venezuela
DENV-3/VE/BID-V2480/2007(3)	Dengue virus 3	FJ850109	10645	Human	Venezuela
DENV-3/VE/BID-V2481/2007(3)	Dengue virus 3	GQ868586	10663	Human	Venezuela
DENV-3/VE/BID-V2482/2007(3)	Dengue virus 3	FJ850110	10663	Human	Venezuela
DENV-3/VE/BID-V2483/2007(3)	Dengue virus 3	GQ868587	10663	Human	Venezuela
DENV-3/VE/BID-V2484/2007(3)	Dengue virus 3	FJ850111	10663	Human	Venezuela
DENV-3/VE/BID-V2579/2001	Dengue virus 3	KF955486	10525	Human	Venezuela
DENV-3/VE/BID-V2580/2001	Dengue virus 3	KF955487	10638	Human	Venezuela
DENV-3/VE/BID-V2965/2000(3)	Dengue virus 3	FJ898468	10663	Human	Venezuela
DENV-3/VE/BID-V2966/2001(3)	Dengue virus 3	FJ898469	10663	Human	Venezuela
DENV-3/VE/BID-V2967/2001(3)	Dengue virus 3	FJ898470	10663	Human	Venezuela
DENV-3/VE/BID-V2968/2002(3)	Dengue virus 3	FJ898471	10663	Human	Venezuela
DENV-3/VE/BID-V2969/2003(3)	Dengue virus 3	FJ898472	10664	Human	Venezuela
DENV-3/VE/BID-V2970/2003(3)	Dengue virus 3	FJ898473	10663	Human	Venezuela
DENV-3/VE/BID-V2971/2007(3)	Dengue virus 3	FJ898474	10664	Human	Venezuela
DENV-3/VE/BID-V903/2001(3)	Dengue virus 3	EU529688	10648	Human	Venezuela
DENV-3/VE/BID-V904/2001(3)	Dengue virus 3	EU482612	10649	Human	Venezuela
DENV-3/VE/BID-V905/2001(3)	Dengue virus 3	EU660420	10663	Human	Venezuela
DENV-3/VE/BID-V906/2001(3)	Dengue virus 3	EU482613	10648	Human	Venezuela
DENV-3/VE/BID-V907/2001(3)	Dengue virus 3	EU529689	10648	Human	Venezuela
DENV-3/VE/BID-V908/2001(3)	Dengue virus 3	EU529690	10648	Human	Venezuela
DENV-3/VE/BID-V911/2001(3)	Dengue virus 3	EU529691	10648	Human	Venezuela
DENV-3/VE/BID-V912/2001(3)	Dengue virus 3	EU569689	10648	Human	Venezuela
DENV-3/VE/BID-V913/2001(3)	Dengue virus 3	EU482614	10648	Human	Venezuela
DENV-3/VE/BID-V915/2001(3)	Dengue virus 3	EU569690	10648	Human	Venezuela
DENV-3/VE/BID-V916/2001(3)	Dengue virus 3	EU569691	10651	Human	Venezuela
DENV-3/VN/BID-V1008/2006(3)	Dengue virus 3	EU482452	10664	Human	Viet Nam

DENV-3/VN/BID-V1009/2006(3)	Dengue virus 3	EU482453	10653	Human	Viet Nam
DENV-3/VN/BID-V1010/2006(3)	Dengue virus 3	EU482454	10663	Human	Viet Nam
DENV-3/VN/BID-V1011/2006(3)	Dengue virus 3	EU482455	10653	Human	Viet Nam
DENV-3/VN/BID-V1012/2006(3)	Dengue virus 3	EU482456	10648	Human	Viet Nam
DENV-3/VN/BID-V1013/2006(3)	Dengue virus 3	EU482457	10663	Human	Viet Nam
DENV-3/VN/BID-V1014/2006(3)	Dengue virus 3	EU482458	10653	Human	Viet Nam
DENV-3/VN/BID-V1015/2006(3)	Dengue virus 3	EU482459	10653	Human	Viet Nam
DENV-3/VN/BID-V1016/2006(3)	Dengue virus 3	EU482460	10636	Human	Viet Nam
DENV-3/VN/BID-V1017/2007(3)	Dengue virus 3	EU482461	10649	Human	Viet Nam
DENV-3/VN/BID-V1018/2007(3)	Dengue virus 3	EU482462	10653	Human	Viet Nam
DENV-3/VN/BID-V1326/2006(3)	Dengue virus 3	EU660407	10663	Human	Viet Nam
DENV-3/VN/BID-V1327/2006(3)	Dengue virus 3	EU660408	10653	Human	Viet Nam
DENV-3/VN/BID-V1329/2006(3)	Dengue virus 3	EU660409	10663	Human	Viet Nam
DENV-3/VN/BID-V1330/2006(3)	Dengue virus 3	EU660410	10653	Human	Viet Nam
DENV-3/VN/BID-V1331/2006(3)	Dengue virus 3	EU660411	10663	Human	Viet Nam
DENV-3/VN/BID-V1769/2007(3)	Dengue virus 3	FJ432722	10648	Human	Viet Nam
DENV-3/VN/BID-V1783/2007(3)	Dengue virus 3	FJ432728	10638	Human	Viet Nam
DENV-3/VN/BID-V1786/2007(3)	Dengue virus 3	FJ432731	10638	Human	Viet Nam
DENV-3/VN/BID-V1790/2007(3)	Dengue virus 3	FJ562097	10648	Human	Viet Nam
DENV-3/VN/BID-V1810/2007(3)	Dengue virus 3	FJ432741	10638	Human	Viet Nam
DENV-3/VN/BID-V1815/2007(3)	Dengue virus 3	FJ562099	10651	Human	Viet Nam
DENV-3/VN/BID-V1817/2007(3)	Dengue virus 3	FJ432743	10648	Human	Viet Nam
DENV-3/VN/BID-V1821/2007	Dengue virus 3	KF955457	10636	Human	Viet Nam
DENV-3/VN/BID-V1824/2007(3)	Dengue virus 3	FJ562100	10638	Human	Viet Nam
DENV-3/VN/BID-V1831/2007(3)	Dengue virus 3	FJ547061	10638	Human	Viet Nam
DENV-3/VN/BID-V1833/2007(3)	Dengue virus 3	FJ547062	10638	Human	Viet Nam
DENV-3/VN/BID-V1874/2008(3)	Dengue virus 3	FJ461322	10638	Human	Viet Nam
DENV-3/VN/BID-V1876/2007	Dengue virus 3	KF955458	10640	Human	Viet Nam
DENV-3/VN/BID-V1877/2007(3)	Dengue virus 3	FJ562102	10638	Human	Viet Nam
DENV-3/VN/BID-V1882/2007(3)	Dengue virus 3	FJ410229	10638	Human	Viet Nam
DENV-3/VN/BID-V1891/2007(3)	Dengue virus 3	FJ461326	10638	Human	Viet Nam
DENV-3/VN/BID-V1897/2007(3)	Dengue virus 3	FJ461329	10638	Human	Viet Nam
DENV-3/VN/BID-V1903/2008	Dengue virus 3	KF955459	10637	Human	Viet Nam
DENV-3/VN/BID-V1911/2008(3)	Dengue virus 3	FJ547066	10637	Human	Viet Nam
DENV-3/VN/BID-V1933/2008	Dengue virus 3	KF955460	10640	Human	Viet Nam
DENV-3/VN/BID-V1936/2008(3)	Dengue virus 3	FJ461334	10638	Human	Viet Nam
DENV-3/VN/BID-V1946/2008(3)	Dengue virus 3	FJ461337	10623	Human	Viet Nam
DENV-3/VN/BID-V1953/2008(3)	Dengue virus 3	FJ461338	10638	Human	Viet Nam
DENV-3/VN/BID-V1957/2008(3)	Dengue virus 3	FJ562103	10644	Human	Viet Nam
DENV-3/WS/BID-V2973/1995(3)	Dengue virus 3	FJ898456	10663	Human	Samoa
DENV3/BR/D3LIMHO/2006-3	Dengue virus 3	JN697379	10696	Human	Brazil
DENV3/Vietnam/10dx-223-801-Placebo-60hrs-3	Dengue virus 3	JQ045687	10665	Human	Viet Nam
DENV3/Vietnam/10dx-223-802-Placebo-60hrs-3	Dengue virus 3	JQ045688	10665	Human	Viet Nam
DENV3/Vietnam/10dx-232-801-Placebo-36hrs-3	Dengue virus 3	JQ045689	10665	Human	Viet Nam
DENV3/Vietnam/10dx-232-802-Placebo-36hrs-3	Dengue virus 3	JQ045690	10665	Human	Viet Nam
DENV3/Vietnam/10dx-235-801-3000mg-0hrs-3	Dengue virus 3	JQ045691	10665	Human	Viet Nam
DENV3/Vietnam/10dx-240-801-3000mg-24hrs-3	Dengue virus 3	JQ045692	10665	Human	Viet Nam
DENV3/Vietnam/10dx-240-802-3000mg-24hrs-3	Dengue virus 3	JQ045693	10638	Human	Viet Nam
DENV3/Vietnam/10dx-242-801-3000mg-12hrs-3	Dengue virus 3	JQ045694	10665	Human	Viet Nam
DENV3/Vietnam/10dx-242-802-3000mg-12hrs-3	Dengue virus 3	JQ045695	10665	Human	Viet Nam
DTID-ZJU04(3)	Dengue virus 3	GU189648	10173	Human	China
FW01(3)	Dengue virus 3	AY858040	10706	Human	Indonesia
FW06(3)	Dengue virus 3	AY858041	10707	Human	Indonesia
GWL-25(3)	Dengue virus 3	AY770511	10707	Unknown	India
GZ/10476/2012	Dengue virus 3	KC261634	10630	Human	China
GZ1D3(3)	Dengue virus 3	GU363549	10707	Human	China
GZ2D3-3	Dengue virus 3	JN662391	10707	Human	China
H87-3	Dengue virus 3	KU050695	10696	Human	Philippines
H87-3	Dengue virus 3	M93130	10696	Unknown	-N/A-
HN/2013/107-3	Dengue virus 3	KJ622197	10710	Human	China
HN/2013/108-3	Dengue virus 3	KJ622198	10710	Human	China
HN/2013/11-3	Dengue virus 3	KJ622191	10710	Human	China
HN/2013/110-3	Dengue virus 3	KJ622199	10710	Human	China
HN/2013/20-3	Dengue virus 3	KJ622192	10710	Human	China
HN/2013/21-3	Dengue virus 3	KJ622193	10706	Human	China
HN/2013/22-3	Dengue virus 3	KJ622194	10711	Human	China
HN/2013/50-3	Dengue virus 3	KJ622195	10711	Human	China
HN/2013/92-3	Dengue virus 3	KJ622196	10710	Human	China
InJ-16-82(3)	Dengue virus 3	DQ401690	10707	Unknown	Indonesia
Jeddah-2014	Dengue virus 3	KJ830751	10635	Human	Saudi Arabia
KDH0010A	Dengue virus 3	HG316483	10707	Unknown	Thailand
KDH0014A	Dengue virus 3	HG316484	10707	Unknown	Thailand
KJ30i(3)	Dengue virus 3	AY858042	10707	Human	Indonesia
KJ46(3)	Dengue virus 3	AY858043	10706	Human	Indonesia
KJ71(3)	Dengue virus 3	AY858044	10707	Human	Indonesia
MKS-0057-3	Dengue virus 3	KC762681	10707	Human	Indonesia
MKS-0076-3	Dengue virus 3	KC762682	10707	Human	Indonesia
MKS-0079-3	Dengue virus 3	KC762683	10707	Human	Indonesia
MKS-0098-3	Dengue virus 3	KC762684	10698	Human	Indonesia
MKS-0172-3	Dengue virus 3	KC762686	10707	Human	Indonesia
MKS-0388-3	Dengue virus 3	KC762685	10707	Human	Indonesia
MKS-0396-3	Dengue virus 3	KC762687	10707	Human	Indonesia
MKS-2006-3	Dengue virus 3	KC762688	10707	Human	Indonesia
MKS-2065-3	Dengue virus 3	KC762689	10707	Human	Indonesia
MKS-3000-3	Dengue virus 3	KC762690	10709	Human	Indonesia
MKS-IF058-3	Dengue virus 3	KC762691	10707	Human	Indonesia
MKS-WS78-3	Dengue virus 3	KC762692	10707	Human	Indonesia
MKS-WS79b-3	Dengue virus 3	KC762693	10707	Human	Indonesia
mutant BDH02-01(3)	Dengue virus 3	DQ401689	10707	Unknown	Bangladesh
mutant BDH02-03(3)	Dengue virus 3	DQ401691	10707	Unknown	Bangladesh
mutant BDH02-04(3)	Dengue virus 3	DQ401692	10707	Unknown	Bangladesh
mutant BDH02-07(3)	Dengue virus 3	DQ401693	10707	Unknown	Bangladesh
mutant InJ-l6-82(3)	Dengue virus 3	DQ401694	10707	Unknown	Indonesia
mutant PhMH-J1-97(3)	Dengue virus 3	DQ401695	10707	Unknown	Philippines
NC89/060289-283-3	Dengue virus 3	JQ920481	10671	Human	New Caledonia
NC95/170895-3750-3	Dengue virus 3	JQ920483	10671	Human	New Caledonia
NC95/240295-418-3	Dengue virus 3	JQ920482	10671	Human	New Caledonia
NC95/271295-4197-3	Dengue virus 3	JQ920484	10671	Human	New Caledonia
NC96/181196-4690-3	Dengue virus 3	JQ920486	10671	Human	New Caledonia
NC96/211096-4631-3	Dengue virus 3	JQ920485	10663	Human	New Caledonia
ND143	Dengue virus 3	FJ644564	10707	Human	India
PAR DENV3 5532-07-3	Dengue virus 3	HQ235027	10707	Human	Paraguay
PF89/27643(3)	Dengue virus 3	AY744677	10707	Human	French Polynesia
PF89/320219(3)	Dengue virus 3	AY744678	10707	Human	French Polynesia
PF90/3050(3)	Dengue virus 3	AY744679	10707	Human	French Polynesia
PF90/3056(3)	Dengue virus 3	AY744680	10707	Human	French Polynesia
PF90/6056(3)	Dengue virus 3	AY744681	10707	Human	French Polynesia
PF91/090891-22130-3	Dengue virus 3	JQ920476	10671	Human	French Polynesia
PF92/2956(3)	Dengue virus 3	AY744682	10707	Human	French Polynesia
PF92/2986(3)	Dengue virus 3	AY744683	10707	Human	-N/A-
PF92/4190(3)	Dengue virus 3	AY744684	10707	Human	French Polynesia
PF93/200493-110185-3	Dengue virus 3	JQ920477	10671	Human	French Polynesia
PF94/136116(3)	Dengue virus 3	AY744685	10707	Human	French Polynesia

	PF95/190795-20064-3	Dengue virus 3	JQ920478	10671	Human	French Polynesia
	PF96/040996-24881-3	Dengue virus 3	JQ920480	10671	Human	French Polynesia
	PF96/150296-46183-3	Dengue virus 3	JQ920479	10671	Human	French Polynesia
	PH86(3)	Dengue virus 3	AY858045	10707	Human	Indonesia
	PhMH-J1-97(3)	Dengue virus 3	AY496879	10707	Unknown	Philippines
	PI64(3)	Dengue virus 3	AY858046	10707	Human	Indonesia
	rDENV3-4	Dengue virus 3	KJ160505	10707	Unknown	-N/A-
	SGEHI(D3)0040Y09(3)	Dengue virus 3	GU370052	10250	Human	Singapore
	SGEHI(D3)0235Y07(3)	Dengue virus 3	GU370053	10248	Human	Singapore
	Singapore 8120/95(3)	Dengue virus 3	AY766104	10696	Unknown	-N/A-
	Singapore(3)	Dengue virus 3	AY662691	10707	Unknown	Singapore
	Sleman/78(3)	Dengue virus 3	AY648961	10707	Unknown	Indonesia
	Taiwan-739079A(3)	Dengue virus 3	AY776329	10490	Unknown	Taiwan
	TB16(3)	Dengue virus 3	AY858047	10688	Human	Indonesia
	TB55i(3)	Dengue virus 3	AY858048	10707	Human	Indonesia
	ThD3_0007_87-3	Dengue virus 3	AY676353	10707	Unknown	Thailand
	ThD3_0010_87-3	Dengue virus 3	AY676352	10709	Unknown	Thailand
	ThD3_0055_93-3	Dengue virus 3	AY676351	10707	Unknown	Thailand
	ThD3_0104_93-3	Dengue virus 3	AY676350	10707	Unknown	Thailand
	ThD3_1283_98-3	Dengue virus 3	AY676349	10707	Unknown	Thailand
	ThD3_1687_98-3	Dengue virus 3	AY676348	10702	Unknown	Thailand
	UNC3001	Dengue virus 3	JQ411814	10707	Human	Sri Lanka
	UNKNOWN-CS805342(3)	Dengue virus 3	CS805342	10707	Unknown	-N/A-
	UNKNOWN-FU267586(3)	Dengue virus 3	FU267586	10707	Unknown	-N/A-
	UNKNOWN-FV537262	Dengue virus 3	FV537262	10696	Unknown	-N/A-
	UNKNOWN-FV537263	Dengue virus 3	FV537263	10696	Unknown	-N/A-
	UNKNOWN-FV537264	Dengue virus 3	FV537264	10696	Unknown	-N/A-
	UNKNOWN-FV537265	Dengue virus 3	FV537265	10696	Unknown	-N/A-
	UNKNOWN-JC562943	Dengue virus 3	JC562943	10707	Unknown	-N/A-
	UNKNOWN-JE963512	Dengue virus 3	JE963512	10706	Unknown	-N/A-
	UNKNOWN-JN368477-3	Dengue virus 3	JN368477	10648	Human	Cambodia
	VE_61035_2006-3	Dengue virus 3	HQ332171	10707	Human	Venezuela
	VE_61051_2006-3	Dengue virus 3	HQ332170	10707	Human	Venezuela
	VIROAF7	Dengue virus 3	KM190937	10267	Human	Philippines
	WF89/140889-4838-3	Dengue virus 3	JQ920487	10671	Human	Wallis and Futuna
	WF95/050495-1650-3	Dengue virus 3	JQ920488	10671	Human	Wallis and Futuna
	WF95/090595-2448-3	Dengue virus 3	JQ920489	10671	Human	Wallis and Futuna
	YN01-3	Dengue virus 3	KF824902	10707	Human	China
	YN02-3	Dengue virus 3	KF824903	10707	Human	China
	YNSW1	Dengue virus 3	KR296743	10710	Unknown	China
	YNSW2	Dengue virus 3	KR296744	10702	Unknown	China
	ZJYW2009-3	Dengue virus 3	JF504679	10685	Human	China
**DENV4**	11/1666	Dengue virus 4	KR922405	10164	Human	Thailand
	2A(4)	Dengue virus 4	AF375822	10649	Unknown	-N/A-
	341750(dengue 4)	Dengue virus 4	GU289913	10649	Human	Colombia
	814669-4	Dengue virus 4	AF326573	10649	Unknown	-N/A-
	B5(4)	Dengue virus 4	AF289029	10665	Unknown	China
	Br246RR/10-4	Dengue virus 4	JN983813	10649	Human	Brazil
	D4/Pakistan/150/2009-4	Dengue virus 4	KF041260	10652	Human	Pakistan
	DENV-4/CO/BID-V1600/1997(4)	Dengue virus 4	FJ024476	10606	Human	Colombia
	DENV-4/CO/BID-V3406/2001(4)	Dengue virus 4	GQ868579	10593	Human	Colombia
	DENV-4/CO/BID-V3407/2001(4)	Dengue virus 4	GQ868580	10598	Human	Colombia
	DENV-4/CO/BID-V3408/2001(4)	Dengue virus 4	GQ868581	10592	Human	Colombia
	DENV-4/CO/BID-V3409/2001(4)	Dengue virus 4	GQ868582	10606	Human	Colombia
	DENV-4/CO/BID-V3410/2004(4)	Dengue virus 4	GQ868583	10551	Human	Colombia
	DENV-4/CO/BID-V3411/2004(4)	Dengue virus 4	GQ868584	10552	Human	Colombia
	DENV-4/CO/BID-V3412/2005(4)	Dengue virus 4	GQ868585	10606	Human	Colombia
	DENV-4/IND/0952326/2009-4	Dengue virus 4	JQ922560	10739	Human	India
	DENV-4/IND/624000/1962-4	Dengue virus 4	JQ922558	10626	Human	India
	DENV-4/IND/793679/1979-4	Dengue virus 4	JQ922559	10616	Human	India
	DENV-4/KBPV-VR-31-4	Dengue virus 4	KP406806	10664	Unknown	South Korea
	DENV-4/KH/BID-V2055/2002	Dengue virus 4	KF955510	10586	Human	Cambodia
	DENV-4/PH/BID-V3361/1956(4)	Dengue virus 4	GQ868594	10607	Human	Philippines
	DENV-4/SG/06K2270DK1/2005(4)	Dengue virus 4	GQ398256	10653	Human	Singapore
	DENV-4/US/BID-V1082/1998(4)	Dengue virus 4	FJ024424	10606	Human	USA
	DENV-4/US/BID-V1083/1986(4)	Dengue virus 4	EU854295	10566	Human	USA
	DENV-4/US/BID-V1093/1998(4)	Dengue virus 4	EU854296	10606	Human	USA
	DENV-4/US/BID-V1094/1998(4)	Dengue virus 4	EU854297	10606	Human	USA
	DENV-4/US/BID-V2429/1994(4)	Dengue virus 4	GQ199878	10592	Human	USA
	DENV-4/US/BID-V2430/1994(4)	Dengue virus 4	GQ199879	10593	Human	USA
	DENV-4/US/BID-V2431/1995(4)	Dengue virus 4	GQ199880	10590	Human	USA
	DENV-4/US/BID-V2432/1995(4)	Dengue virus 4	GQ252675	10591	Human	USA
	DENV-4/US/BID-V2433/1995(4)	Dengue virus 4	FJ810417	10606	Human	USA
	DENV-4/US/BID-V2434/1995(4)	Dengue virus 4	FJ850057	10606	Human	USA
	DENV-4/US/BID-V2435/1996(4)	Dengue virus 4	GQ199881	10590	Human	USA
	DENV-4/US/BID-V2436/1996(4)	Dengue virus 4	GQ199882	10592	Human	USA
	DENV-4/US/BID-V2437/1996(4)	Dengue virus 4	GQ199883	10606	Human	USA
	DENV-4/US/BID-V2438/1996(4)	Dengue virus 4	GQ199884	10606	Human	USA
	DENV-4/US/BID-V2439/1996(4)	Dengue virus 4	GQ199885	10590	Human	USA
	DENV-4/US/BID-V2440/1996(4)	Dengue virus 4	FJ850058	10606	Human	USA
	DENV-4/US/BID-V2441/1998(4)	Dengue virus 4	FJ882595	10606	Human	USA
	DENV-4/US/BID-V2442/1998(4)	Dengue virus 4	FJ882596	10606	Human	USA
	DENV-4/US/BID-V2443/1998(4)	Dengue virus 4	FJ850059	10606	Human	USA
	DENV-4/US/BID-V2444/1998(4)	Dengue virus 4	FJ882597	10606	Human	USA
	DENV-4/US/BID-V2445/1998(4)	Dengue virus 4	FJ882598	10606	Human	USA
	DENV-4/US/BID-V2446/1999(4)	Dengue virus 4	FJ882599	10595	Human	USA
	DENV-4/US/BID-V2447/1999(4)	Dengue virus 4	FJ882600	10606	Human	USA
	DENV-4/US/BID-V2448/1999(4)	Dengue virus 4	FJ882601	10606	Human	USA
	DENV-4/US/BID-V860/1994(4)	Dengue virus 4	FJ226067	10606	Human	USA
	DENV-4/VE/BID-V1153/2007(4)	Dengue virus 4	GQ868642	10595	Human	Venezuela
	DENV-4/VE/BID-V1154/2007(4)	Dengue virus 4	GQ868643	10593	Human	Venezuela
	DENV-4/VE/BID-V1155/2007(4)	Dengue virus 4	GQ868644	10637	Human	Venezuela
	DENV-4/VE/BID-V1156/2007(4)	Dengue virus 4	GQ868645	10606	Human	Venezuela
	DENV-4/VE/BID-V1157/2007(4)	Dengue virus 4	EU854299	10606	Human	Venezuela
	DENV-4/VE/BID-V1158/2007(4)	Dengue virus 4	FJ182016	10606	Human	Venezuela
	DENV-4/VE/BID-V1159/2007(4)	Dengue virus 4	EU854300	10606	Human	Venezuela
	DENV-4/VE/BID-V1160/2007(4)	Dengue virus 4	FJ182017	10606	Human	Venezuela
	DENV-4/VE/BID-V1161/2007(4)	Dengue virus 4	EU854301	10606	Human	Venezuela
	DENV-4/VE/BID-V2163/1998(4)	Dengue virus 4	FJ639736	10606	Human	Venezuela
	DENV-4/VE/BID-V2164/1998(4)	Dengue virus 4	FJ639737	10567	Human	Venezuela
	DENV-4/VE/BID-V2165/1998(4)	Dengue virus 4	FJ639738	10566	Human	Venezuela
	DENV-4/VE/BID-V2166/1998(4)	Dengue virus 4	FJ639739	10606	Human	Venezuela
	DENV-4/VE/BID-V2167/1998	Dengue virus 4	JN819409	10558	Human	Venezuela
	DENV-4/VE/BID-V2170/1999(4)	Dengue virus 4	FJ639742	10593	Human	Venezuela
	DENV-4/VE/BID-V2172/1999(4)	Dengue virus 4	FJ639744	10606	Human	Venezuela
	DENV-4/VE/BID-V2173/1999(4)	Dengue virus 4	FJ639745	10566	Human	Venezuela
	DENV-4/VE/BID-V2176/2000(4)	Dengue virus 4	FJ850095	10558	Human	Venezuela
	DENV-4/VE/BID-V2177/2000(4)	Dengue virus 4	FJ639748	10551	Human	Venezuela
	DENV-4/VE/BID-V2194/2001(4)	Dengue virus 4	FJ639764	10566	Human	Venezuela
	DENV-4/VE/BID-V2206/2001(4)	Dengue virus 4	FJ639773	10606	Human	Venezuela
	DENV-4/VE/BID-V2489/2007(4)	Dengue virus 4	FJ882580	10596	Human	Venezuela
	DENV-4/VE/BID-V2490/2007(4)	Dengue virus 4	FJ882581	10606	Human	Venezuela
	DENV-4/VE/BID-V2491/2007(4)	Dengue virus 4	FJ882582	10603	Human	Venezuela

	DENV-4/VE/BID-V2492/2007(4)	Dengue virus 4	FJ882583	10592	Human	Venezuela
	DENV-4/VE/BID-V2493/2007(4)	Dengue virus 4	FJ882584	10595	Human	Venezuela
	DENV-4/VE/BID-V2494/2007(4)	Dengue virus 4	FJ882585	10594	Human	Venezuela
	DENV-4/VE/BID-V2495/2007(4)	Dengue virus 4	FJ882586	10606	Human	Venezuela
	DENV-4/VE/BID-V2496/2007(4)	Dengue virus 4	FJ882587	10554	Human	Venezuela
	DENV-4/VE/BID-V2497/2007(4)	Dengue virus 4	FJ882588	10594	Human	Venezuela
	DENV-4/VE/BID-V2498/2007(4)	Dengue virus 4	FJ882589	10595	Human	Venezuela
	DENV-4/VE/BID-V2499/2007(4)	Dengue virus 4	FJ882590	10594	Human	Venezuela
	DENV-4/VE/BID-V2500/2007(4)	Dengue virus 4	FJ882591	10593	Human	Venezuela
	DENV-4/VE/BID-V2501/2008(4)	Dengue virus 4	FJ882592	10606	Human	Venezuela
	DENV-4/VE/BID-V2607/2006	Dengue virus 4	JN819406	10357	Human	Venezuela
	DENV-4/VE/BID-V2610/2007(4)	Dengue virus 4	GQ199876	10595	Human	Venezuela
	DENV4/CN/GZ29/2010-4	Dengue virus 4	KP723482	10653	Human	China
	DF patient-4	Dengue virus 4	JN638570	10650	Human	Cambodia
	DHF patient-4	Dengue virus 4	JN638571	10656	Human	Cambodia
	DSS patient-4	Dengue virus 4	JN638572	10656	Human	Cambodia
	EHI310A129CY10-4	Dengue virus 4	JX024757	10653	Human	Singapore
	EHI310A129SY10-4	Dengue virus 4	JX024758	10653	Human	Singapore
	GD07/78	Dengue virus 4	FJ196849	10649	Unknown	China
	GD09/90	Dengue virus 4	FJ196850	10649	Unknown	China
	GZ/9809/2012	Dengue virus 4	KC333651	10574	Human	China
	GZ1D4-4	Dengue virus 4	JF741967	10631	Human	China
	GZ30-4	Dengue virus 4	JQ822247	10653	Human	China
	H241-4	Dengue virus 4	AY947539	10643	Human	-N/A-
	H241-4	Dengue virus 4	KR011349	10664	Human	Philippines
	H402276-4	Dengue virus 4	JN559740	10604	Human	Brazil
	H772846-4	Dengue virus 4	JQ513330	10604	Human	Brazil
	H772852-4	Dengue virus 4	JQ513331	10604	Human	Brazil
	H772854-4	Dengue virus 4	JN559741	10604	Human	Brazil
	H773583-4	Dengue virus 4	JQ513332	10604	Human	Brazil
	H774846-4	Dengue virus 4	JQ513333	10604	Human	Brazil
	H775222-4	Dengue virus 4	JQ513334	10604	Human	Brazil
	H778494-4	Dengue virus 4	JQ513335	10604	Human	Brazil
	H778504-4	Dengue virus 4	JQ513336	10604	Human	Brazil
	H778887-4	Dengue virus 4	JQ513337	10604	Human	Brazil
	H779228-4	Dengue virus 4	JQ513338	10604	Human	Brazil
	H779652-4	Dengue virus 4	JQ513339	10604	Human	Brazil
	H780090-4	Dengue virus 4	JQ513340	10604	Human	Brazil
	H780120-4	Dengue virus 4	JQ513341	10604	Human	Brazil
	H780556-4	Dengue virus 4	JQ513342	10604	Human	Brazil
	H780563-4	Dengue virus 4	JQ513343	10604	Human	Brazil
	H780571-4	Dengue virus 4	JQ513344	10604	Human	Brazil
	H781363-4	Dengue virus 4	JQ513345	10604	Human	Brazil
	Haiti/0324/2014-4	Dengue virus 4	KT276273	10649	Human	Haiti
	Haiti73-4	Dengue virus 4	JF262782	10649	Human	Haiti
	INDIA G11337-4	Dengue virus 4	JF262783	10659	Human	India
	INH6412-4	Dengue virus 4	JF262781	10649	Human	Venezuela
	MKS-0033-4	Dengue virus 4	KC762694	10636	Human	Indonesia
	MKS-0070-4	Dengue virus 4	KC762695	10619	Human	Indonesia
	MKS-0252-4	Dengue virus 4	KC762696	10653	Human	Indonesia
	MKS-0706-4	Dengue virus 4	KC762697	10653	Human	Indonesia
	MKS-2007-4	Dengue virus 4	KC762698	10653	Human	Indonesia
	MKS-2139-4	Dengue virus 4	KC762699	10641	Human	Indonesia
	MRS 6169642904/2014	Dengue virus 4	KP140942	10565	Human	Haiti
	NC08/200208-409-4	Dengue virus 4	JQ915085	10572	Human	New Caledonia
	NC09/020409-9266-4	Dengue virus 4	JQ915088	10572	Human	New Caledonia
	NC09/050209-1413-4	Dengue virus 4	JQ915086	10572	Human	New Caledonia
	NC09/170309-6652-4	Dengue virus 4	JQ915087	10246	Human	New Caledonia
	NC09/230909-14518-4	Dengue virus 4	JQ915089	10572	Human	New Caledonia
	P73-1120-4	Dengue virus 4	JF262780	10667	Monkey	Malaysia
	P75-215-4	Dengue virus 4	EF457906	10666	Unknown	Malaysia
	P75-514-4	Dengue virus 4	JF262779	10667	Monkey	Malaysia
	PF09/080409-93-4	Dengue virus 4	JQ915083	10572	Human	French Polynesia
	PF09/230309-126-4	Dengue virus 4	JQ915082	10572	Human	French Polynesia
	PF09/290109-69-4	Dengue virus 4	JQ915081	10572	Human	French Polynesia
	PF10/150610-28-4	Dengue virus 4	JQ915084	10573	Human	French Polynesia
	rDEN4	Dengue virus 4	NC_002640 *	10649	Unknown	-N/A-
	rDENV4	Dengue virus 4	KJ160504	10650	Human	Sri Lanka
	recombinant clone 2Adel30(4)	Dengue virus 4	AF326826	10618	Unknown	-N/A-
	recombinant clone rDEN4del30(4)	Dengue virus 4	AF326827	10618	Unknown	-N/A-
	Singapore 8976/95(4)	Dengue virus 4	AY762085	10649	Unknown	-N/A-
	Taiwan-2K0713(4)	Dengue virus 4	AY776330	10353	Unknown	Taiwan
	ThD4_0017_97-4	Dengue virus 4	AY618989	10653	Unknown	Thailand
	ThD4_0087_77-4	Dengue virus 4	AY618991	10650	Unknown	Thailand
	ThD4_0348_91-4	Dengue virus 4	AY618990	10650	Unknown	Thailand
	ThD4_0476_97-4	Dengue virus 4	AY618988	10653	Unknown	Thailand
	ThD4_0485_01-4	Dengue virus 4	AY618992	10649	Unknown	Thailand
	ThD4_0734_00-4	Dengue virus 4	AY618993	10653	Unknown	Thailand
	UNKNOWN-AF326825(4)	Dengue virus 4	AF326825	10649	Unknown	-N/A-
	UNKNOWN-FV537266	Dengue virus 4	FV537266	10649	Unknown	-N/A-
	UNKNOWN-FV537267	Dengue virus 4	FV537267	10649	Unknown	-N/A-
	UNKNOWN-FV537268	Dengue virus 4	FV537268	10649	Unknown	-N/A-
	UNKNOWN-FV537269	Dengue virus 4	FV537269	10649	Unknown	-N/A-
	UNKNOWN-JC562944	Dengue virus 4	JC562944	10649	Unknown	-N/A-
	UNKNOWN-JE963513	Dengue virus 4	JE963513	10652	Unknown	-N/A-
	UNKNOWN-M14931(4)	Dengue virus 4	M14931	10648	Unknown	-N/A-
	VE_61013_2007-4	Dengue virus 4	HQ332175	10649	Human	Venezuela
	VE_61027_2007-4	Dengue virus 4	HQ332172	10649	Human	Venezuela
	VE_61054_2007-4	Dengue virus 4	HQ332176	10649	Human	Venezuela
	VE_61073_2007-4	Dengue virus 4	HQ332174	10649	Human	Venezuela
	VE_61110_2007-4	Dengue virus 4	HQ332173	10649	Human	Venezuela
	VIROAF8	Dengue virus 4	KM190936	10592	Human	Thailand
	WF09/010409-0001-4	Dengue virus 4	JQ915090	10246	Human	Wallis and Futuna
**WNV**	CH16532	West Nile virus	JX276662	10927	Unknown	-N/A-
	FLSDX	West Nile virus	AY274504	11022	Unknown	-N/A-
	MRM61C	West Nile virus	D00246	10664	Unknown	-N/A-
	pAKUN	West Nile virus	AY274505	11022	Unknown	-N/A-
	007WG-TX05EP	West Nile virus	GQ507468	10500	Human	USA
	009WG-NM05LC	West Nile virus	GQ507469	10500	Human	USA
	011WG-TX06EP	West Nile virus	GQ507470	10500	Human	USA
	013WG-TX07EP	West Nile virus	GQ507471	10825	Human	USA
	024WG-CA03OR	West Nile virus	GQ507472	10825	Human	USA
	03-104WI	West Nile virus	DQ431696	10520	Human	USA
	03-113FL	West Nile virus	DQ431697	10520	Human	USA
	03-120FL	West Nile virus	DQ431698	10520	Human	USA
	03-124FL	West Nile virus	DQ431699	10520	Human	USA
	03-20TX	West Nile virus	DQ431693	10520	Human	USA
	03-22TX	West Nile virus	DQ431694	10520	Human	-N/A-
	03-82IL	West Nile virus	DQ431695	10520	Human	USA
	04-213CA	West Nile virus	DQ431700	10480	Human	USA
	04-214CO	West Nile virus	DQ431701	10520	Human	USA
	04-216CO	West Nile virus	DQ431702	10520	Human	USA
	04-218CO	West Nile virus	DQ431703	10520	Human	USA

04-219CO	West Nile virus	DQ431704	10521	Human	USA
04-233ND	West Nile virus	DQ431705	10840	Human	USA
04-236NM	West Nile virus	DQ431706	10843	Human	USA
04-237NM	West Nile virus	DQ431707	10520	Human	USA
04-238CA	West Nile virus	DQ431708	10521	Human	USA
04-240CA	West Nile virus	DQ431709	10521	Human	USA
04-244CA	West Nile virus	DQ431710	10846	Human	USA
04-251AZ	West Nile virus	DQ431711	10520	Human	USA
04-252AZ	West Nile virus	DQ431712	10520	Human	USA
04.05	West Nile virus	AY701413	10945	Horse	Morocco
080WG-CA04LA	West Nile virus	GQ507473	10825	Human	USA
091WG-CA04SB	West Nile virus	GQ507474	10825	Human	USA
099WG-CA05SB	West Nile virus	GQ507475	10825	Human	USA
1	West Nile virus	DQ164196	11029	Human	USA
1	West Nile virus	DQ164198	11029	Human	USA
1	West Nile virus	DQ164204	11029	Hawk	USA
101_5-06-Uu	West Nile virus	FJ159129	10845	Mosquito	Russia
101WG-CA05SB	West Nile virus	GQ507476	10825	Human	USA
103WG-CA05LA	West Nile virus	GQ507477	10825	Human	USA
116WG-CA05LA	West Nile virus	GQ507478	10825	Human	USA
124WG-AZ05PI	West Nile virus	GQ507479	10820	Human	USA
1270/14	West Nile virus	KT207792	10995	Mosquito	Italy
132WG-CA05LA	West Nile virus	GQ507480	10825	Human	USA
142WG-NE06DO	West Nile virus	GQ507481	10825	Human	USA
144WG-AZ06PI	West Nile virus	GQ507482	10825	Human	USA
148WG-CA07LA	West Nile virus	GQ507483	10825	Human	USA
149WG-CA07LA	West Nile virus	GQ507484	10825	Human	USA
15217	West Nile virus	FJ483548	11030	Unknown	Italy
15803	West Nile virus	FJ483549	11030	Unknown	Italy
2	West Nile virus	DQ164197	11029	Human	USA
2	West Nile virus	DQ164203	11029	Magpie	USA
2	West Nile virus	DQ164205	11029	Human	USA
3356.2.1.1(JEV)	West Nile virus	EF530047	11029	Crow	USA
3356K VP2	West Nile virus	EF657887	11029	Crow	USA
349/77	West Nile virus	KM052152	11014	Horse	South Africa
385-99	West Nile virus	EF571854	11029	Unknown	USA
4	West Nile virus	DQ164206	11029	Blue Jay	USA
578/10	West Nile virus	KC496015	11028	Horse	Hungary
5_50-05-Uu	West Nile virus	FJ159130	10845	Mosquito	Russia
68856	West Nile virus	EU249803	11029	Bat	-N/A-
68856-ICDC-4	West Nile virus	KT163243	11029	Unknown	India
792/14	West Nile virus	KT207791	10983	Mosquito	Italy
804994	West Nile virus	DQ256376	11014	Human	India
8_1-05-Uu	West Nile virus	FJ159131	10845	Mosquito	Russia
956	West Nile virus	NC_001563 *	10962	Unknown	-N/A-
96-111	West Nile virus	AY701412	10945	Horse	Morocco
A-AZ-03-1623	West Nile virus	DQ080051	11029	Mosquito	USA
ABB-B13	West Nile virus	KC407667	11029	Mouse	Spain
ArB310/67	West Nile virus	GQ851608	10964	Unknown	Central African Republic
ArB3573/82	West Nile virus	DQ318020	11048	Unknown	Central African Republic
ARC10	West Nile virus	AY795965	11029	Unknown	USA
ARC10-06	West Nile virus	JF957161	11016	Unknown	USA
ARC13-06	West Nile virus	JF957162	11016	Unknown	USA
ARC140-07	West Nile virus	JF957168	11016	Unknown	USA
ARC17-06	West Nile virus	JF957163	11016	Unknown	USA
ARC23-06	West Nile virus	JF957164	11029	Unknown	USA
ARC27-06	West Nile virus	JF957165	11016	Unknown	USA
ARC33-06	West Nile virus	JF957166	11029	Unknown	USA
ArD27875	West Nile virus	GQ851606	10951	Unknown	Senegal
ArD76104	West Nile virus	DQ318019	11038	Unknown	Senegal
ArEq001	West Nile virus	GQ379160	11029	Horse	Argentina
ArEq003	West Nile virus	GQ379161	11029	Horse	Argentina
Ast-986	West Nile virus	JX041634	10998	Human	Russia
Ast01-182(Japanese encephalitis virus group)	West Nile virus	DQ411030	10811	Tick	Russia
Ast01-187(Japanese encephalitis virus group)	West Nile virus	DQ411031	10811	Crow	Russia
Ast01-66(Japanese encephalitis virus group)	West Nile virus	DQ411029	10811	Cormorant	Russia
Ast02-2-25(Japanese encephalitis virus group)	West Nile virus	DQ374653	10811	Crow	Russia
Ast02-2-26(Japanese encephalitis virus group)	West Nile virus	DQ377178	10811	Tick	Russia
Ast02-2-298(Japanese encephalitis virus group)	West Nile virus	DQ377179	10811	Rook	Russia
Ast02-2-691(Japanese encephalitis virus group)	West Nile virus	DQ411034	10811	Mosquito	Russia
Ast02-2-692(Japanese encephalitis virus group)	West Nile virus	DQ411035	10811	Mosquito	Russia
Ast02-3-146(Japanese encephalitis virus group)	West Nile virus	DQ411032	10811	Pigeon	Russia
Ast02-3-165(Japanese encephalitis virus group)	West Nile virus	DQ411033	10811	Cormorant	Russia
Ast02-3-208(Japanese encephalitis virus group)	West Nile virus	DQ377180	10811	Cormorant	Russia
Ast02-3-570(Japanese encephalitis virus group)	West Nile virus	DQ374651	10811	Magpie	Russia
Ast02-3-717(Japanese encephalitis virus group)	West Nile virus	DQ374650	10811	Cormorant	Russia
Ast04-2-824A(Japanese encephalitis virus group)	West Nile virus	DQ374652	10789	Crow	Russia
Ast99-901	West Nile virus	AY278441	10998	Unknown	Russia
Austria/2008_gh	West Nile virus	KF179640	10998	Goshawk	Austria
AVA1202598	West Nile virus	KC736486	11029	Mosquito	USA
AVA1202600	West Nile virus	KC736487	11029	Mosquito	USA
AVA1202606	West Nile virus	KC736488	11029	Mosquito	USA
AVA1202615	West Nile virus	KC736489	11029	Mosquito	USA
AVA1202621	West Nile virus	KC736490	11029	Mosquito	USA
AVA1202624	West Nile virus	KC736491	11029	Mosquito	USA
AVA1202689	West Nile virus	KC736492	11029	Mosquito	USA
AVA1202696	West Nile virus	KC736493	11029	Mosquito	USA
AVA1204250	West Nile virus	KC736494	11029	Mosquito	USA
AVA1204260	West Nile virus	KC736502	11029	Mosquito	USA
AVA1204331	West Nile virus	KC736495	11029	Mosquito	USA
AVA1204356	West Nile virus	KC736496	11029	Mosquito	USA
AVA1204485	West Nile virus	KC736497	11029	Mosquito	USA
AVA1204579	West Nile virus	KC736498	11029	Mosquito	USA
AVA1204580	West Nile virus	KC736499	11029	Mosquito	USA
AVA1204753	West Nile virus	KC736500	11029	Mosquito	USA
AVA1204895	West Nile virus	KC736501	11029	Mosquito	USA
AZ 2004	West Nile virus	DQ164201	11029	Human	USA
AZ10-581	West Nile virus	KF704153	11017	Mosquito	USA
AZ10-892	West Nile virus	KF704158	11024	Mosquito	USA
AZ10-91	West Nile virus	KF704147	11008	Mosquito	USA
B-AZ-03-1681	West Nile virus	DQ080052	11029	Mosquito	USA
B956	West Nile virus	AY532665	11038	Unknown	-N/A-
BC787	West Nile virus	AB185916	11029	Unknown	-N/A-
BC787	West Nile virus	AB185917	11029	Unknown	-N/A-
BD-AUT	West Nile virus	KM659876	11028	Human	Austria
Bird 1153	West Nile virus	AY712945	11029	Dove	USA
Bird 1171	West Nile virus	AY712946	11029	Blue Jay	USA
Bird 1461	West Nile virus	AY712947	11029	Unknown	USA
Bird114	West Nile virus	GU827998	10941	Avian	USA
Bird1175	West Nile virus	GU828000	10978	Avian	USA
Bird1519	West Nile virus	GU828004	10959	Avian	USA
Bird1576	West Nile virus	GU827999	10992	Avian	USA
Bird1881	West Nile virus	GU828003	10936	Avian	USA

blood donor/Vienna/2014	West Nile virus	KP109691	10988	Human	Austria
BSL106-06	West Nile virus	JF957167	11016	Unknown	USA
BSL11-09	West Nile virus	JF957178	11029	Unknown	USA
BSL13-2005	West Nile virus	DQ666451	11029	Unknown	USA
BSL173-08	West Nile virus	JF957173	11029	Unknown	USA
BSL176-08	West Nile virus	JF957174	11029	Unknown	USA
BSL18-09	West Nile virus	JF957179	11029	Unknown	USA
BSL2-09	West Nile virus	JF957175	11016	Unknown	USA
BSL2-10	West Nile virus	JF957185	11029	Unknown	USA
BSL2-2005	West Nile virus	DQ666452	11015	Unknown	USA
BSL20-09	West Nile virus	JF957180	11029	Unknown	USA
BSL22-09	West Nile virus	JF957181	11029	Unknown	USA
BSL23-11	West Nile virus	JQ700440	11029	Human	USA
BSL24-09	West Nile virus	JF957182	11029	Unknown	USA
BSL24-11	West Nile virus	JQ700441	11029	Human	USA
BSL26-11	West Nile virus	JQ700442	11029	Human	USA
BSL27-09	West Nile virus	JF957183	11029	Unknown	USA
BSL3-10	West Nile virus	JF957186	11029	Unknown	USA
BSL4-11	West Nile virus	JQ700438	11016	Human	USA
BSL5-09	West Nile virus	JF957176	11029	Unknown	USA
BSL5-2004	West Nile virus	DQ666448	11029	Unknown	USA
BSL6-09	West Nile virus	JF957177	11029	Unknown	USA
BSL6-11	West Nile virus	JQ700439	11029	Human	USA
BVI2013_Heart	West Nile virus	KF367469	10763	Flamingo	British Virgin Islands
C-AZ-03 03-1799	West Nile virus	DQ080053	11029	Mosquito	USA
CA-03 COAV997	West Nile virus	JF703162	11029	Mosquito	USA
CA-03 IMPR116	West Nile virus	JF703164	11029	Mosquito	USA
CA-04 COAV689	West Nile virus	JF703161	11029	Mosquito	USA
CA-05 COAV2900	West Nile virus	JF703163	11029	Mosquito	USA
Chin-01	West Nile virus	AY490240	11028	Unknown	China
CO4-07	West Nile virus	JF957169	11026	Unknown	USA
CO5-07	West Nile virus	JF957170	11029	Unknown	USA
CO7-09	West Nile virus	JF957184	11029	Unknown	USA
COL524/08	West Nile virus	JN716371	10754	American Flamingo	Colombia
COL9835/08	West Nile virus	JN716372	10754	American Flamingo	Colombia
Connecticut 1999	West Nile virus	AF206518	10975	Mosquito	USA
Cx. pipiens/Vienna/2014	West Nile virus	KP109692	10988	Mosquito	Austria
Cz 13-104	West Nile virus	KM203860	11018	Mosquito	Czech Republic
Cz 13-329	West Nile virus	KM203861	11018	Mosquito	Czech Republic
Cz 13-479	West Nile virus	KM203862	11018	Mosquito	Czech Republic
Cz 13-502	West Nile virus	KM203863	11018	Mosquito	Czech Republic
DB080718-14	West Nile virus	GQ379157	11029	Crow	USA
E-CA-03 GRLA-1260	West Nile virus	DQ080054	11029	Mosquito	USA
Eg101	West Nile virus	AF260968	11029	Unknown	-N/A-
Egypt 101	West Nile virus	EU081844	11029	Unknown	Egypt
EthAn4766	West Nile virus	AY603654	11029	Unknown	Ethiopia
F-CA-03 IMPR 102	West Nile virus	DQ080055	11018	Mosquito	USA
FDA-BSL5-2003	West Nile virus	DQ005530	11029	Unknown	USA
FL2001 crow 67030	West Nile virus	GQ379156	11029	Crow	USA
FL232	West Nile virus	DQ080072	11000	Bird	USA
FL234	West Nile virus	DQ080071	11000	Horse	Mexico
France 405/04	West Nile virus	DQ786572	10938	Avian	France
France 407/04	West Nile virus	DQ786573	10938	Avian	France
FtC-3699	West Nile virus	KR868734	11029	Mosquito	USA
G-CA-03 IMPR-1075	West Nile virus	DQ080056	11019	Mosquito	USA
G15578	West Nile virus	GQ851604	10489	Unknown	India
G16146	West Nile virus	GQ851605	10997	Mosquito	India
GCTX1-2005	West Nile virus	DQ666449	11029	Unknown	USA
GCTX2-2005	West Nile virus	DQ666450	11029	Unknown	USA
GE-1b/B	West Nile virus	FJ766331	10960	Eagle	Spain
GE-2o/V	West Nile virus	FJ766332	10961	Eagle	Spain
goose-Hungary/03(lineage 1a)	West Nile virus	DQ118127	10969	Goose	Hungary
goshawk-Hungary/04	West Nile virus	DQ116961	11028	Goshawk	Hungary
Greece/2012/Kavala/39.1	West Nile virus	KF179639	11000	Human	Greece
Greece/2013/C105	West Nile virus	KJ577738	10998	Human	Greece
Greece/2013/C147	West Nile virus	KJ577739	11003	Human	Greece
Greece/2013/Kavala_1	West Nile virus	KJ883342	11011	Human	Greece
Greece/2013/Kavala_2	West Nile virus	KJ883348	11002	Human	Greece
Greece/2013/Serres_1	West Nile virus	KJ883347	11013	Human	Greece
Greece/2013/Thessaloniki_3	West Nile virus	KJ883350	11013	Human	Greece
Greece/2013/Thessaloniki_4	West Nile virus	KJ883346	11000	Human	Greece
Greece/2013/Xanthi_1	West Nile virus	KJ883343	11002	Human	Greece
Greece/2013/Xanthi_2	West Nile virus	KJ883344	11003	Human	Greece
Greece/2013/Xanthi_3	West Nile virus	KJ883345	11008	Human	Greece
Greece/2013/Xanthi_4	West Nile virus	KJ883341	11013	Human	Greece
Greece/2013/Xanthi_5	West Nile virus	KJ883349	10967	Human	Greece
HNY1999	West Nile virus	AF202541	10945	Unknown	USA
HU6365/08	West Nile virus	JF707789	10900	Mosquito	Spain
Hyalomma/Romania/2013	West Nile virus	KJ934710	11013	Tick	Romania
I-CA-03 Arcadia-S0331532	West Nile virus	DQ080057	10956	Crow	USA
IBAN7019	West Nile virus	GQ851607	10962	Unknown	Nigeria
ID21bird-07	West Nile virus	JF957171	11016	Unknown	USA
ID28bird-07	West Nile virus	JF957172	11016	Unknown	USA
Ig2266	West Nile virus	JX041632	10900	Mosquito	India
IN 2002	West Nile virus	DQ164200	11029	Human	USA
IS-98 STD1	West Nile virus	AF481864	11029	Stork	-N/A-
Is98	West Nile virus	KR107956	10435	Unknown	Belgium
Ita09	West Nile virus	GU011992	11030	Human	Italy
Italy/2008/J-242853	West Nile virus	JF719065	10969	Jay	Italy
Italy/2008/M-203204	West Nile virus	JF719066	10953	Magpie	Italy
Italy/2009/FIN	West Nile virus	KF234080	11030	Human	Italy
Italy/2009/G-223184	West Nile virus	JF719067	10926	Gull	Italy
Italy/2009/J-225677	West Nile virus	JF719068	10926	Jay	Italy
Italy/2011/AN-1	West Nile virus	JN858069	10895	Human	Italy
Italy/2011/AN-2	West Nile virus	JN858070	10520	Human	Italy
Italy/2011/Livenza	West Nile virus	JQ928174	10714	Human	Italy
Italy/2011/Piave	West Nile virus	JQ928175	10477	Human	Italy
Italy/2012/Livenza/31.1	West Nile virus	JX556213	10736	Human	Italy
Italy/2012/Livenza/37.1	West Nile virus	KC954092	10781	Human	Italy
Italy/2013/Livenza/35.1	West Nile virus	KF647253	10483	Human	Italy
Italy/2013/Mantova/36.1	West Nile virus	KP789960	10949	Human	Italy
Italy/2013/Mantova/40.1	West Nile virus	KF823806	10975	Human	Italy
Italy/2013/Padova/34.1	West Nile virus	KF647251	10994	Human	Italy
Italy/2013/Rovigo/32.1	West Nile virus	KF588365	10998	Human	Italy
Italy/2013/Rovigo/33.1	West Nile virus	KF647250	11013	Human	Italy
Italy/2013/Rovigo/33.2	West Nile virus	KF647249	10986	Human	Italy
Italy/2013/Rovigo/34.1	West Nile virus	KF647248	11013	Human	Italy
Italy/2013/Rovigo/35.1	West Nile virus	KF647252	10974	Human	Italy
Italy/2013/Rovigo/35.2	West Nile virus	KF823805	11011	Human	Italy
Italy/2014/Cremona2	West Nile virus	KP789954	11000	Human	Italy
Italy/2014/Cremona4	West Nile virus	KP789957	10999	Human	Italy
Italy/2014/Pavia1	West Nile virus	KP789953	10994	Human	Italy
Italy/2014/Pavia4	West Nile virus	KP789959	10947	Human	Italy

Italy/2014/Pavia5	West Nile virus	KP789958	11000	Human	Italy
Italy/2014/Verona/35.1	West Nile virus	KP789955	10995	Human	Italy
Italy/2014/Verona/35.2	West Nile virus	KP789956	10938	Human	Italy
J-CA-03 Arcadia-S0334814	West Nile virus	DQ080058	11029	Crow	USA
JPW080813-01	West Nile virus	GQ379159	11029	Squirrel	USA
K6453	West Nile virus	GQ851603	10983	Mosquito	Australia
KN3829	West Nile virus	AY262283	10984	Mosquito	-N/A-
L-CA-04 SAC-04-7168	West Nile virus	DQ080059	11029	Magpie	USA
LEIV-1628Az	West Nile virus	JX041629	10898	Bird	Azerbaijan
LEIV-1640Az	West Nile virus	JX041630	10810	Bird	Azerbaijan
LEIV-3266Ukr	West Nile virus	JX041631	10820	Bird	Ukraine
LEIV-72Az	West Nile virus	JX041628	10942	Tick	Azerbaijan
LEIV-Krnd88-190	West Nile virus	AY277251	10741	Tick	Russia
LEIV-Vlg00-27924	West Nile virus	AY278442	10842	Unknown	Russia
LEIV-Vlg99-27889	West Nile virus	AY277252	10845	Unknown	Russia
LSU-AR01	West Nile virus	FJ527738	11029	Blue Jay	USA
M12214	West Nile virus	JF415914	11029	Mosquito	USA
M19433	West Nile virus	JF415919	11029	Mosquito	USA
M20122	West Nile virus	JF415928	11029	Mosquito	USA
M20140	West Nile virus	JF415926	11029	Mosquito	USA
M20141	West Nile virus	JF415927	11031	Mosquito	USA
M37012	West Nile virus	JF415922	11029	Mosquito	USA
M37906	West Nile virus	JF415923	11028	Mosquito	USA
M38488	West Nile virus	JF415925	11028	Mosquito	USA
M6019	West Nile virus	JF415930	11029	Mosquito	USA
Madagascar-AnMg798	West Nile virus	DQ176636	10866	Unknown	Madagascar
Mosquito v4369	West Nile virus	AY712948	11029	Mosquito	USA
MRM16	West Nile virus	GQ851602	10981	Mosquito	Australia
Nea Santa-Greece-2010-Lineage 2	West Nile virus	HQ537483	11028	Mosquito	Greece
New York 99	West Nile virus	HQ596519	11029	Crow	USA
Novi Sad-2010	West Nile virus	KC496016	11028	Mosquito	Serbia
NSW2011	West Nile virus	JN887352	10923	Horse	Australia
NY 2001 Suffolk	West Nile virus	DQ164194	11029	Crow	USA
NY 2002 Broome	West Nile virus	DQ164187	11029	Crow	USA
NY 2002 Clinton	West Nile virus	DQ164193	11029	Crow	USA
NY 2002 Nassau	West Nile virus	DQ164195	11029	Mosquito	USA
NY 2002 Queens	West Nile virus	DQ164186	11029	Crow	USA
NY 2003 Albany	West Nile virus	DQ164189	11029	Crow	USA
NY 2003 Chautauqua	West Nile virus	DQ164191	11029	Crow	USA
NY 2003 Rockland	West Nile virus	DQ164192	11029	Crow	USA
NY 2003 Suffolk	West Nile virus	DQ164190	11029	Crow	USA
NY 2003 Westchester	West Nile virus	DQ164188	11029	Crow	USA
NY-99	West Nile virus	KC407666	11029	Unknown	-N/A-
NY10-03	West Nile virus	JQ700437	11028	Mosquito	USA
NY2001-6263	West Nile virus	KJ786934	11026	Human	USA
NY2001-6263	West Nile virus	NC_027013 *	11026	Human	USA
NY99	West Nile virus	DQ211652	11029	Unknown	USA
NY99	West Nile virus	NC_009942 *	11029	Unknown	USA
NY99-6922	West Nile virus	AB185914	11029	Unknown	-N/A-
NY99-6922	West Nile virus	AB185915	11029	Unknown	-N/A-
NY99-6922	West Nile virus	FJ411043	11029	Unknown	-N/A-
NY99-crow-V76/1	West Nile virus	FJ151394	11029	Crow	USA
NY99-eqhs	West Nile virus	AF260967	11029	Horse	USA
NY99-flamingo382-99	West Nile virus	AF196835	11029	Unknown	USA
NY99P2	West Nile virus	KM083619	11029	Flamingo	USA
OH 2002	West Nile virus	DQ164202	11029	Human	USA
OK03	West Nile virus	EU155484	10822	Unknown	USA
ORCO0559-07	West Nile virus	GQ379158	11029	Mosquito	USA
PaAn001	West Nile virus	AY268132	10989	Unknown	-N/A-
PaH001	West Nile virus	AY268133	10989	Unknown	-N/A-
patent WO2006029300_1	West Nile virus	CS568914	11029	Unknown	-N/A-
patent WO2006029300_3	West Nile virus	CS568916	11029	Unknown	-N/A-
patent WO2006029300_4	West Nile virus	CS568917	11029	Unknown	-N/A-
patent WO2006029300_5	West Nile virus	CS568918	11029	Unknown	-N/A-
patent WO2006029300_6	West Nile virus	CS568919	11029	Unknown	-N/A-
patent WO2007015783_1	West Nile virus	CS543188	11029	Unknown	-N/A-
Q3574-5	West Nile virus	GQ903680	11062	Unknown	Cyprus
Rabensburg	West Nile virus	AY765264	10972	Mosquito	-N/A-
Reb_VLG_07_H	West Nile virus	FJ425721	10980	Human	Russia
RO97-50	West Nile virus	AF260969	11029	Mosquito	Romania
Sad/12	West Nile virus	KC407673	11028	Northern Goshawk	Serbia
Sarafend	West Nile virus	AY688948	11057	Unknown	Israel
Spain/2010/H-1b	West Nile virus	JF719069	10990	Horse	Spain
SPU116-89	West Nile virus	EU068667	10305	Unknown	South Africa
T-1304	West Nile virus	JX070655	10845	Unknown	Tajikistan
T2	West Nile virus	KJ958922	11026	Horse	Turkey
TM171-03	West Nile virus	AY660002	11029	Unknown	Mexico
TM171-03	West Nile virus	JN051152	10998	Raven	Mexico
TM171-03	West Nile virus	JN051153	10998	Raven	Mexico
TVP 8533	West Nile virus	AY289214	11029	Unknown	-N/A-
TVP9115	West Nile virus	DQ080070	11001	Grackle	Mexico
TVP9117	West Nile virus	DQ080069	10975	Horse	Mexico
TVP9218	West Nile virus	DQ080068	11001	Heron	Mexico
TVP9219	West Nile virus	DQ080067	11001	Heron	Mexico
TVP9220	West Nile virus	DQ080066	11001	Cormorant	Mexico
TVP9221	West Nile virus	DQ080065	10995	Grackle	Mexico
TVP9222	West Nile virus	DQ080064	11001	Coot	Mexico
TVP9223	West Nile virus	DQ080063	10975	Pigeon	Mexico
TWN165	West Nile virus	DQ080062	11001	Mosquito	USA
TWN496	West Nile virus	DQ080061	11000	Cardinal	USA
twn9	West Nile virus	HM051416	11029	Human	Israel
TX 2002-HC	West Nile virus	DQ176637	10678	Unknown	USA
TX 2003	West Nile virus	DQ164199	11029	Human	USA
TX5058	West Nile virus	JF415929	11023	Blue Jay	USA
TX5810	West Nile virus	JF415915	11029	Grackle	USA
TX6276	West Nile virus	JF415916	11029	Mockingbird	USA
TX6647	West Nile virus	JF415917	11028	Blue Jay	USA
TX6747	West Nile virus	JF415918	11029	Blue Jay	USA
TX7191	West Nile virus	JF415920	11029	Blue Jay	USA
TX7558	West Nile virus	JF415921	11028	Blue Jay	USA
TX7827	West Nile virus	JF415924	11028	Blue Jay	USA
TX8759	West Nile virus	KJ786936	10981	Blue Jay	USA
TX8779	West Nile virus	KJ786935	11028	Mockingbird	USA
TX_AR10-5718	West Nile virus	JX015522	10976	Mosquito	USA
TX_AR10-6572	West Nile virus	JX015523	10997	Mosquito	USA
TX_AR5-2686	West Nile virus	JX015515	10954	Mosquito	USA
TX_AR7-6745	West Nile virus	JX015516	11002	Mosquito	USA
TX_AR8-5947	West Nile virus	JX015517	10994	Mosquito	USA
TX_AR8-6866	West Nile virus	JX015518	11001	Mosquito	Mexico
TX_AR9-5282	West Nile virus	JX015519	11018	Mosquito	USA
TX_AR9-6115	West Nile virus	JX015520	10949	Mosquito	Mexico
TX_AR9-7465	West Nile virus	JX015521	10999	Mosquito	USA
UNKNOWN-AF533540	West Nile virus	AF533540	11029	Unknown	USA

UNKNOWN-FV537222	West Nile virus	FV537222	10962	Unknown	-N/A-
UNKNOWN-FV537223	West Nile virus	FV537223	10962	Unknown	-N/A-
UNKNOWN-FV537224	West Nile virus	FV537224	10962	Unknown	-N/A-
UNKNOWN-FV537225	West Nile virus	FV537225	10962	Unknown	-N/A-
UNKNOWN-HC467807	West Nile virus	HC467807	11029	Unknown	-N/A-
UNKNOWN-HH961658	West Nile virus	HH961658	10975	Unknown	-N/A-
UNKNOWN-HH961659	West Nile virus	HH961659	11029	Unknown	-N/A-
UNKNOWN-HM147822	West Nile virus	HM147822	11065	Unknown	South Africa
UNKNOWN-HM147823	West Nile virus	HM147823	11051	Unknown	Madagascar
UNKNOWN-HM147824	West Nile virus	HM147824	11050	Unknown	Democratic Republic of the Congo
UNKNOWN-HV572312	West Nile virus	HV572312	11029	Unknown	-N/A-
UNKNOWN-HW816192	West Nile virus	HW816192	11029	Unknown	-N/A-
UNKNOWN-M12294	West Nile virus	M12294	10962	Unknown	-N/A-
USA2002	West Nile virus	AY646354	11030	Human	USA
Uu-LN-AT-2013	West Nile virus	KJ831223	10666	Mosquito	Austria
V11-03	West Nile virus	JX123030	10983	Horse	Australia
V11-07	West Nile virus	JX123031	10983	Horse	Australia
v4095	West Nile virus	GU828002	10958	Mosquito	USA
v4380	West Nile virus	GU828001	10959	Mosquito	USA
VLG-4	West Nile virus	AF317203	10972	Human	Russia
WN Italy 1998-equine	West Nile virus	AF404757	11029	Horse	Italy
WN MD 2000-crow265	West Nile virus	AF404753	11029	Crow	USA
WN NJ 2000 MQ5488	West Nile virus	AF404754	11029	Mosquito	USA
WN NY 2000-crow3356	West Nile virus	AF404756	11029	Crow	USA
WN NY 2000-grouse3282	West Nile virus	AF404755	11029	Grouse	USA
WNV-1/BID-V5030	West Nile virus	JN819305	10692	Unknown	-N/A-
WNV-1/BID-V5031	West Nile virus	JN819306	10592	Unknown	-N/A-
WNV-1/BID-V5032	West Nile virus	JN819307	10584	Unknown	-N/A-
WNV-1/BID-V5036	West Nile virus	JN819309	10464	Unknown	-N/A-
WNV-1/BID-V5037	West Nile virus	JN819310	10581	Unknown	-N/A-
WNV-1/BID-V5038	West Nile virus	JN819311	10451	Mosquito	-N/A-
WNV-1/BID-V5039	West Nile virus	JN819312	10584	Mosquito	-N/A-
WNV-1/BID-V5040	West Nile virus	JN819313	10436	Mosquito	-N/A-
WNV-1/BID-V5042	West Nile virus	JN819315	10583	Bird	-N/A-
WNV-1/BID-V5043	West Nile virus	JN819316	10584	Unknown	-N/A-
WNV-1/BID-V5044	West Nile virus	JN819317	10594	Unknown	-N/A-
WNV-1/BID-V5045	West Nile virus	JN819318	10584	Bird	-N/A-
WNV-1/BID-V5047	West Nile virus	JN819319	10380	Bird	-N/A-
WNV-1/BID-V5048	West Nile virus	JN819320	10414	Bird	-N/A-
WNV-1/BID-V5050	West Nile virus	JN819323	10488	Unknown	-N/A-
WNV-1/BID-V5051	West Nile virus	JN819321	10584	Unknown	-N/A-
WNV-1/BID-V5052	West Nile virus	JN819324	10588	Unknown	-N/A-
WNV-1/CTFS/BID-V4111/2006	West Nile virus	HM488122	10617	Mosquito	-N/A-
WNV-1/CTFS/BID-V4112/2006	West Nile virus	HM488123	10449	Mosquito	-N/A-
WNV-1/CTFS/BID-V4113/2006	West Nile virus	HM488124	10622	Hamster	-N/A-
WNV-1/Culex/BID-V4161/legs	West Nile virus	HQ671687	10622	Mosquito	-N/A-
WNV-1/Culex/BID-V4163/legs	West Nile virus	HQ705670	10623	Mosquito	-N/A-
WNV-1/Culex/BID-V4165/legs	West Nile virus	HQ671688	10619	Mosquito	-N/A-
WNV-1/Culex/BID-V4166/midgut	West Nile virus	HQ705671	10620	Mosquito	-N/A-
WNV-1/Culex/BID-V4168/legs	West Nile virus	HQ705672	10623	Mosquito	-N/A-
WNV-1/Culex/BID-V4169/midgut	West Nile virus	HQ705673	10621	Mosquito	-N/A-
WNV-1/Culex/BID-V4171/midgut	West Nile virus	HQ671731	10620	Mosquito	-N/A-
WNV-1/Culex/BID-V4173/midgut	West Nile virus	HQ705674	10623	Mosquito	-N/A-
WNV-1/Culex/BID-V4174/legs	West Nile virus	HQ671689	10619	Mosquito	-N/A-
WNV-1/Culex/BID-V4180/midgut	West Nile virus	HQ671690	10604	Mosquito	-N/A-
WNV-1/Culex/BID-V4184/legs	West Nile virus	HQ705675	10620	Mosquito	-N/A-
WNV-1/Culex/BID-V5768/midgut	West Nile virus	JN183895	10634	Mosquito	-N/A-
WNV-1/Culex/BID-V5776/midgut	West Nile virus	JN183896	10622	Mosquito	-N/A-
WNV-1/Culex/BID-V5808/midgut	West Nile virus	JN183897	10622	Mosquito	-N/A-
WNV-1/Gallus/BID-V4954/kidney	West Nile virus	JN183893	10610	Chicken	-N/A-
WNV-1/Gallus/BID-V4955/kidney	West Nile virus	JN183894	10613	Chicken	-N/A-
WNV-1/Gallus/BID-V4958/kidney	West Nile virus	HQ671691	10642	Chicken	-N/A-
WNV-1/Gallus/BID-V4959/kidney	West Nile virus	HQ671692	10624	Chicken	-N/A-
WNV-1/Gallus/BID-V4960/kidney	West Nile virus	HQ705677	10613	Chicken	-N/A-
WNV-1/Gallus/BID-V4961/kidney	West Nile virus	HQ671693	10614	Chicken	-N/A-
WNV-1/Gallus/BID-V4962/kidney	West Nile virus	HQ705678	10609	Chicken	-N/A-
WNV-1/Gallus/BID-V4963/kidney	West Nile virus	HQ671694	10613	Chicken	-N/A-
WNV-1/Gallus/BID-V5109/skin	West Nile virus	JF357960	10621	Chicken	-N/A-
WNV-1/Gallus/BID-V5112/skin	West Nile virus	HQ671695	10612	Chicken	-N/A-
WNV-1/Mus/BID-V4728/spleen	West Nile virus	HQ671668	10623	Mouse	-N/A-
WNV-1/Mus/BID-V4729/spleen	West Nile virus	JF730041	10497	Mouse	-N/A-
WNV-1/Mus/BID-V4730/spleen	West Nile virus	HQ671669	10957	Mouse	-N/A-
WNV-1/Mus/BID-V4731/spleen	West Nile virus	HQ671670	10708	Mouse	-N/A-
WNV-1/Mus/BID-V4920/spleen	West Nile virus	HQ671671	10619	Mouse	-N/A-
WNV-1/Mus/BID-V4921/spleen	West Nile virus	HQ671672	10610	Mouse	-N/A-
WNV-1/Mus/BID-V4923/spleen	West Nile virus	JF899530	10613	Mouse	-N/A-
WNV-1/Mus/BID-V4924/spleen	West Nile virus	HQ671673	10614	Mouse	-N/A-
WNV-1/Mus/BID-V4925/spleen	West Nile virus	HQ671674	10613	Mouse	-N/A-
WNV-1/Mus/BID-V4926/spleen	West Nile virus	HQ671675	10613	Mouse	-N/A-
WNV-1/Mus/BID-V4936/spleen	West Nile virus	HQ705663	10516	Mouse	-N/A-
WNV-1/Mus/BID-V4937/spleen	West Nile virus	HQ671676	10504	Mouse	-N/A-
WNV-1/Mus/BID-V4938/spleen	West Nile virus	HQ671677	10621	Mouse	-N/A-
WNV-1/Mus/BID-V4940/spleen	West Nile virus	HQ671678	10462	Mouse	-N/A-
WNV-1/Mus/BID-V4969/serum	West Nile virus	HQ671679	10787	Mouse	-N/A-
WNV-1/Mus/BID-V4971/serum	West Nile virus	HQ705676	10583	Mouse	-N/A-
WNV-1/Mus/BID-V4974/serum	West Nile virus	JF784158	10621	Mouse	-N/A-
WNV-1/Mus/BID-V4979/serum	West Nile virus	JF899531	10623	Mouse	-N/A-
WNV-1/Mus/BID-V4980/serum	West Nile virus	HQ891009	10584	Mouse	-N/A-
WNV-1/Mus/BID-V4981/serum	West Nile virus	HQ671680	10584	Mouse	-N/A-
WNV-1/Mus/BID-V4982/serum	West Nile virus	JF730040	10584	Mouse	-N/A-
WNV-1/Mus/BID-V4985/spleen	West Nile virus	HQ671681	10632	Mouse	-N/A-
WNV-1/Mus/BID-V4986/spleen	West Nile virus	HQ671682	10599	Mouse	-N/A-
WNV-1/Mus/BID-V4987/spleen	West Nile virus	HQ891010	10584	Mouse	-N/A-
WNV-1/Mus/BID-V4992/brain	West Nile virus	JF899532	10622	Mouse	-N/A-
WNV-1/Mus/BID-V4993/brain	West Nile virus	HQ891011	10584	Mouse	-N/A-
WNV-1/Mus/BID-V4996/brain	West Nile virus	JF899533	10631	Mouse	-N/A-
WNV-1/Mus/BID-V4997/brain	West Nile virus	JF357958	10617	Mouse	-N/A-
WNV-1/Mus/BID-V4999/brain	West Nile virus	JF357959	10546	Mouse	-N/A-
WNV-1/Mus/BID-V5000/brain	West Nile virus	HQ671732	10590	Mouse	-N/A-
WNV-1/Mus/BID-V5002/brain	West Nile virus	JF899534	10434	Mouse	-N/A-
WNV-1/Mus/BID-V5004/brain	West Nile virus	HQ671733	10584	Mouse	-N/A-
WNV-1/Mus/BID-V5006/brain	West Nile virus	HQ671683	10785	Mouse	-N/A-
WNV-1/Mus/BID-V5007/brain	West Nile virus	HQ671684	10584	Mouse	-N/A-
WNV-1/Mus/BID-V5008/brain	West Nile virus	HQ671685	10584	Mouse	-N/A-
WNV-1/Mus/BID-V5017/brain	West Nile virus	HQ671686	10583	Mouse	-N/A-
WNV-1/Mus/BID-V5118/serum	West Nile virus	HQ891012	10585	Mouse	-N/A-
WNV-1/Mus/BID-V5122/serum	West Nile virus	HQ891013	10584	Mouse	-N/A-
WNV-1/Mus/BID-V5142/brain	West Nile virus	JF899535	10580	Mouse	-N/A-
WNV-1/Mus/BID-V5145/brain	West Nile virus	JF899536	10601	Mouse	-N/A-
WNV-1/Mus/BID-V5146/brain	West Nile virus	JF899537	10622	Mouse	-N/A-
WNV-1/US/BID-V4090/2007	West Nile virus	HM488199	10614	Avian	USA
WNV-1/US/BID-V4092/2007	West Nile virus	HM488200	10621	Avian	USA
WNV-1/US/BID-V4093/2007	West Nile virus	HM488201	10665	Avian	USA

WNV-1/US/BID-V4094/2007	West Nile virus	HM488202	10463	Blue Jay	USA
WNV-1/US/BID-V4095/2007	West Nile virus	HM756678	10621	Avian	USA
WNV-1/US/BID-V4096/2008	West Nile virus	HM488203	10620	Avian	USA
WNV-1/US/BID-V4097/2008	West Nile virus	HM756660	10547	Hawk	USA
WNV-1/US/BID-V4098/2008	West Nile virus	HM488204	10617	Avian	USA
WNV-1/US/BID-V4099/2008	West Nile virus	HM488205	10621	Avian	USA
WNV-1/US/BID-V4100/2008	West Nile virus	HM488206	10541	Avian	USA
WNV-1/US/BID-V4101/2008	West Nile virus	HM488207	10621	Blue Jay	USA
WNV-1/US/BID-V4102/2002	West Nile virus	HM488114	11020	Mosquito	USA
WNV-1/US/BID-V4103/2005	West Nile virus	HM488115	10621	Mosquito	USA
WNV-1/US/BID-V4104/2005	West Nile virus	HM488116	10904	Mosquito	USA
WNV-1/US/BID-V4105/2005	West Nile virus	HM488117	10621	Mosquito	USA
WNV-1/US/BID-V4106/2005	West Nile virus	KJ145799	10621	Mosquito	USA
WNV-1/US/BID-V4107/2005	West Nile virus	HM488118	10622	Mosquito	USA
WNV-1/US/BID-V4108/2005	West Nile virus	HM488119	10622	Mosquito	USA
WNV-1/US/BID-V4109/2005	West Nile virus	HM488120	10621	Mosquito	USA
WNV-1/US/BID-V4110/2005	West Nile virus	HM488121	10718	Mosquito	USA
WNV-1/US/BID-V4186/1999	West Nile virus	HM488125	10516	Crow	USA
WNV-1/US/BID-V4187/1999	West Nile virus	HM488126	10598	Crow	USA
WNV-1/US/BID-V4188/1999	West Nile virus	HM488127	10625	Crow	USA
WNV-1/US/BID-V4189/1999	West Nile virus	HM488128	10616	Crow	USA
WNV-1/US/BID-V4191/2000	West Nile virus	HM488129	10617	Mosquito	USA
WNV-1/US/BID-V4192/2000	West Nile virus	HM488130	10621	Mosquito	USA
WNV-1/US/BID-V4193/2000	West Nile virus	HM488131	10620	Mosquito	USA
WNV-1/US/BID-V4194/2000	West Nile virus	HM488132	10621	Mosquito	USA
WNV-1/US/BID-V4195/2001	West Nile virus	HM488133	10621	Mosquito	USA
WNV-1/US/BID-V4196/2001	West Nile virus	HQ671696	10618	Mosquito	USA
WNV-1/US/BID-V4198/2001	West Nile virus	HM488134	10513	Mosquito	USA
WNV-1/US/BID-V4199/2001	West Nile virus	HM488135	10621	Mosquito	USA
WNV-1/US/BID-V4200/2001	West Nile virus	HM488136	10621	Mosquito	USA
WNV-1/US/BID-V4202/2002	West Nile virus	HM488137	10621	Mosquito	USA
WNV-1/US/BID-V4203/2002	West Nile virus	HQ671698	10554	Mosquito	USA
WNV-1/US/BID-V4204/2002	West Nile virus	HM488208	10691	Mosquito	USA
WNV-1/US/BID-V4205/2002	West Nile virus	HM756648	10621	Mosquito	USA
WNV-1/US/BID-V4206/2002	West Nile virus	HQ671699	10667	Mosquito	USA
WNV-1/US/BID-V4207/2003	West Nile virus	HM488138	10621	Mosquito	USA
WNV-1/US/BID-V4208/2003	West Nile virus	HM488139	10445	Mosquito	USA
WNV-1/US/BID-V4209/2003	West Nile virus	HQ705659	10522	Mosquito	USA
WNV-1/US/BID-V4210/2003	West Nile virus	HM488140	10466	Mosquito	USA
WNV-1/US/BID-V4212/2003	West Nile virus	HM488141	10523	Mosquito	USA
WNV-1/US/BID-V4214/2004	West Nile virus	HM488142	10421	Mosquito	USA
WNV-1/US/BID-V4215/2004	West Nile virus	HM488143	10621	Mosquito	USA
WNV-1/US/BID-V4216/2004	West Nile virus	HM488144	11029	Mosquito	USA
WNV-1/US/BID-V4217/2004	West Nile virus	HM488145	10621	Mosquito	USA
WNV-1/US/BID-V4218/2004	West Nile virus	HM488146	10518	Mosquito	USA
WNV-1/US/BID-V4219/2004	West Nile virus	HM488147	10621	Mosquito	USA
WNV-1/US/BID-V4220/2004	West Nile virus	HM488148	10621	Mosquito	USA
WNV-1/US/BID-V4223/2005	West Nile virus	HM488149	10621	Mosquito	USA
WNV-1/US/BID-V4224/2005	West Nile virus	HM488150	10621	Mosquito	USA
WNV-1/US/BID-V4225/2005	West Nile virus	HM488151	10621	Mosquito	USA
WNV-1/US/BID-V4226/2005	West Nile virus	HM488152	10567	Mosquito	USA
WNV-1/US/BID-V4227/2005	West Nile virus	HM488153	10643	Mosquito	USA
WNV-1/US/BID-V4228/2005	West Nile virus	HM488154	10625	Mosquito	USA
WNV-1/US/BID-V4229/2006	West Nile virus	HM488155	10617	Mosquito	USA
WNV-1/US/BID-V4230/2006	West Nile virus	HM488156	10621	Mosquito	USA
WNV-1/US/BID-V4231/2006	West Nile virus	HM488157	10645	Mosquito	USA
WNV-1/US/BID-V4232/2006	West Nile virus	HM488158	10504	Mosquito	USA
WNV-1/US/BID-V4233/2006	West Nile virus	HM488159	10434	Mosquito	USA
WNV-1/US/BID-V4336/2002	West Nile virus	HM488177	10621	Avian	USA
WNV-1/US/BID-V4337/2002	West Nile virus	HM488178	10614	Avian	USA
WNV-1/US/BID-V4338/2002	West Nile virus	HM488179	10452	Avian	USA
WNV-1/US/BID-V4339/2002	West Nile virus	HM488180	10541	Avian	USA
WNV-1/US/BID-V4340/2002	West Nile virus	HM488181	10433	Avian	USA
WNV-1/US/BID-V4341/2002	West Nile virus	HM488182	10474	Avian	USA
WNV-1/US/BID-V4342/2002	West Nile virus	HQ705669	10451	Blue Jay	USA
WNV-1/US/BID-V4343/2002	West Nile virus	HQ671742	10622	Blue Jay	USA
WNV-1/US/BID-V4344/2002	West Nile virus	JN183891	10520	Blue Jay	USA
WNV-1/US/BID-V4345/2002	West Nile virus	HM488183	10621	Blue Jay	USA
WNV-1/US/BID-V4346/2002	West Nile virus	HM488184	10621	Blue Jay	USA
WNV-1/US/BID-V4347/2003	West Nile virus	HM488185	10621	Blue Jay	USA
WNV-1/US/BID-V4349/2003	West Nile virus	HM756676	10478	Avian	USA
WNV-1/US/BID-V4350/2003	West Nile virus	HM488186	10440	Avian	USA
WNV-1/US/BID-V4351/2003	West Nile virus	HM488187	10442	Avian	USA
WNV-1/US/BID-V4353/2004	West Nile virus	HM488188	10621	Avian	USA
WNV-1/US/BID-V4354/2006	West Nile virus	HM756649	10485	Mosquito	USA
WNV-1/US/BID-V4355/2006	West Nile virus	HM488160	10617	Mosquito	USA
WNV-1/US/BID-V4356/2007	West Nile virus	HM488161	10620	Mosquito	USA
WNV-1/US/BID-V4357/2007	West Nile virus	HM488162	10610	Mosquito	USA
WNV-1/US/BID-V4359/2007	West Nile virus	HM488163	10621	Mosquito	USA
WNV-1/US/BID-V4360/2007	West Nile virus	HM488164	10453	Mosquito	USA
WNV-1/US/BID-V4361/2007	West Nile virus	HM488165	10534	Mosquito	USA
WNV-1/US/BID-V4362/2008	West Nile virus	HM488166	10687	Mosquito	USA
WNV-1/US/BID-V4363/2008	West Nile virus	HM488167	10619	Mosquito	USA
WNV-1/US/BID-V4364/2008	West Nile virus	HM488168	10706	Mosquito	USA
WNV-1/US/BID-V4365/2008	West Nile virus	HM488169	10502	Mosquito	USA
WNV-1/US/BID-V4366/2008	West Nile virus	HM488170	10620	Mosquito	USA
WNV-1/US/BID-V4367/2004	West Nile virus	HM488189	10508	Blue Jay	USA
WNV-1/US/BID-V4368/2004	West Nile virus	HM488190	10402	Avian	USA
WNV-1/US/BID-V4369/2004	West Nile virus	HM488191	10621	Avian	USA
WNV-1/US/BID-V4371/2005	West Nile virus	HM488192	10610	Avian	USA
WNV-1/US/BID-V4373/2005	West Nile virus	HM488193	10409	Avian	USA
WNV-1/US/BID-V4374/2005	West Nile virus	HM488194	10663	Avian	USA
WNV-1/US/BID-V4375/2005	West Nile virus	HM488195	10488	Blue Jay	USA
WNV-1/US/BID-V4376/2005	West Nile virus	HM488196	10437	Avian	USA
WNV-1/US/BID-V4377/2005	West Nile virus	HM488197	10620	Avian	USA
WNV-1/US/BID-V4378/2005	West Nile virus	HM488198	10617	Mosquito	USA
WNV-1/US/BID-V4379/2005	West Nile virus	JN183892	10622	Mosquito	USA
WNV-1/US/BID-V4529/2007	West Nile virus	KJ145832	10621	Mammal	USA
WNV-1/US/BID-V4530/2005	West Nile virus	HM756677	10617	Avian	USA
WNV-1/US/BID-V4553/2006	West Nile virus	HM488253	10636	Mosquito	USA
WNV-1/US/BID-V4559/2007	West Nile virus	HM488254	10621	Mosquito	USA
WNV-1/US/BID-V4560/2003	West Nile virus	HM488171	10854	Mosquito	USA
WNV-1/US/BID-V4561/2003	West Nile virus	HM488172	10887	Mosquito	USA
WNV-1/US/BID-V4562/2003	West Nile virus	HM488173	10902	Mosquito	USA
WNV-1/US/BID-V4563/2003	West Nile virus	HM488174	10741	Mosquito	USA
WNV-1/US/BID-V4564/2003	West Nile virus	HM488209	10622	Mosquito	USA
WNV-1/US/BID-V4565/2003	West Nile virus	HM488210	10709	Mosquito	USA
WNV-1/US/BID-V4566/2003	West Nile virus	HM488211	10649	Mosquito	USA
WNV-1/US/BID-V4567/2003	West Nile virus	HM488212	10761	Mosquito	USA
WNV-1/US/BID-V4568/2003	West Nile virus	JF920728	10622	Mosquito	USA
WNV-1/US/BID-V4569/2003	West Nile virus	HM488175	10707	Mosquito	USA
WNV-1/US/BID-V4571/2003	West Nile virus	HM488213	10672	Mosquito	USA
WNV-1/US/BID-V4572/2003	West Nile virus	HM488214	10621	Mosquito	USA

WNV-1/US/BID-V4573/2003	West Nile virus	HM488215	10621	Mosquito	USA
WNV-1/US/BID-V4574/2003	West Nile virus	HM488216	10645	Mosquito	USA
WNV-1/US/BID-V4575/2003	West Nile virus	HM488176	11018	Mosquito	USA
WNV-1/US/BID-V4576/2003	West Nile virus	HQ671700	10645	Mosquito	USA
WNV-1/US/BID-V4579/2003	West Nile virus	JN183889	10521	Mosquito	USA
WNV-1/US/BID-V4581/2003	West Nile virus	HM488217	10467	Mosquito	USA
WNV-1/US/BID-V4582/2003	West Nile virus	HM756650	10809	Mosquito	USA
WNV-1/US/BID-V4583/2003	West Nile virus	HM488218	10491	Mosquito	USA
WNV-1/US/BID-V4584/2003	West Nile virus	HM756651	10637	Mosquito	USA
WNV-1/US/BID-V4585/2003	West Nile virus	HM488219	10985	Mosquito	USA
WNV-1/US/BID-V4586/2003	West Nile virus	HM488220	10621	Mosquito	USA
WNV-1/US/BID-V4587/2003	West Nile virus	HM756652	10619	Mosquito	USA
WNV-1/US/BID-V4588/2003	West Nile virus	HM756653	10621	Mosquito	USA
WNV-1/US/BID-V4590/2003	West Nile virus	HQ671701	10734	Mosquito	USA
WNV-1/US/BID-V4593/2003	West Nile virus	HM488221	10704	Mosquito	USA
WNV-1/US/BID-V4595/2003	West Nile virus	HQ671702	10662	Mosquito	USA
WNV-1/US/BID-V4597/2003	West Nile virus	JF920306	10700	Mosquito	USA
WNV-1/US/BID-V4598/2003	West Nile virus	HM756654	10509	Mosquito	USA
WNV-1/US/BID-V4599/2003	West Nile virus	HM488222	10649	Mosquito	USA
WNV-1/US/BID-V4603/2003	West Nile virus	HM488223	10619	Mosquito	USA
WNV-1/US/BID-V4604/2003	West Nile virus	HM488224	10439	Mosquito	USA
WNV-1/US/BID-V4605/2003	West Nile virus	HM488225	10620	Mosquito	USA
WNV-1/US/BID-V4607/2003	West Nile virus	HM488226	10621	Mosquito	USA
WNV-1/US/BID-V4608/2003	West Nile virus	HM488227	10655	Mosquito	USA
WNV-1/US/BID-V4609/2003	West Nile virus	HM488228	10621	Mosquito	USA
WNV-1/US/BID-V4610/2003	West Nile virus	HM488229	10622	Mosquito	USA
WNV-1/US/BID-V4611/2003	West Nile virus	HQ671703	10613	Mosquito	USA
WNV-1/US/BID-V4612/2003	West Nile virus	HM488230	10985	Mosquito	USA
WNV-1/US/BID-V4613/2003	West Nile virus	HM488231	10501	Mosquito	USA
WNV-1/US/BID-V4614/2003	West Nile virus	HM488232	10737	Mosquito	USA
WNV-1/US/BID-V4615/2003	West Nile virus	HM756656	10649	Mosquito	USA
WNV-1/US/BID-V4616/2003	West Nile virus	HM488233	10732	Mosquito	USA
WNV-1/US/BID-V4617/2003	West Nile virus	HM488234	10741	Mosquito	USA
WNV-1/US/BID-V4618/2003	West Nile virus	HQ671704	10804	Mosquito	USA
WNV-1/US/BID-V4619/2003	West Nile virus	HM488235	10908	Mosquito	USA
WNV-1/US/BID-V4620/2003	West Nile virus	HQ671705	10731	Mosquito	USA
WNV-1/US/BID-V4622/2008	West Nile virus	HM488237	10621	Avian	USA
WNV-1/US/BID-V4623/2008	West Nile virus	HM488238	10621	Avian	USA
WNV-1/US/BID-V4624/2008	West Nile virus	HM488239	10621	Avian	USA
WNV-1/US/BID-V4625/2008	West Nile virus	HQ671721	10622	Crow	USA
WNV-1/US/BID-V4626/2008	West Nile virus	JN183885	10521	Blue Jay	USA
WNV-1/US/BID-V4627/2008	West Nile virus	HM488240	10403	Blue Jay	USA
WNV-1/US/BID-V4628/2008	West Nile virus	HM488241	10623	Avian	USA
WNV-1/US/BID-V4629/2008	West Nile virus	JN183886	10671	Blue Jay	USA
WNV-1/US/BID-V4631/2008	West Nile virus	HM488242	10478	Avian	USA
WNV-1/US/BID-V4632/2008	West Nile virus	HM488243	10618	Avian	USA
WNV-1/US/BID-V4634/2008	West Nile virus	HM488244	10629	Avian	USA
WNV-1/US/BID-V4635/2008	West Nile virus	HM488245	10621	Avian	USA
WNV-1/US/BID-V4685/2003	West Nile virus	HM756657	11026	Mosquito	USA
WNV-1/US/BID-V4686/2003	West Nile virus	HM756658	10613	Mosquito	USA
WNV-1/US/BID-V4687/2003	West Nile virus	HM756659	10660	Mosquito	USA
WNV-1/US/BID-V4689/2001	West Nile virus	HM488246	10533	Avian	USA
WNV-1/US/BID-V4691/2001	West Nile virus	HM488247	10612	Avian	USA
WNV-1/US/BID-V4692/2001	West Nile virus	HM756661	10613	Avian	USA
WNV-1/US/BID-V4693/2001	West Nile virus	HM756662	10612	Avian	USA
WNV-1/US/BID-V4694/2001	West Nile virus	HM488248	10608	Avian	USA
WNV-1/US/BID-V4696/2001	West Nile virus	HM488249	10484	Avian	USA
WNV-1/US/BID-V4697/2001	West Nile virus	HM756663	10611	Avian	USA
WNV-1/US/BID-V4699/2003	West Nile virus	JN183890	10526	Mosquito	USA
WNV-1/US/BID-V4700/2003	West Nile virus	HM488236	10843	Mosquito	USA
WNV-1/US/BID-V4701/2002	West Nile virus	HM756664	10611	Avian	USA
WNV-1/US/BID-V4704/2002	West Nile virus	HQ671722	10454	Crow	USA
WNV-1/US/BID-V4706/2002	West Nile virus	JN183887	10685	Crow	USA
WNV-1/US/BID-V4709/2002	West Nile virus	HM756665	10488	Avian	USA
WNV-1/US/BID-V4711/2003	West Nile virus	HM756666	10611	Avian	USA
WNV-1/US/BID-V4712/2003	West Nile virus	HM756667	10613	Avian	USA
WNV-1/US/BID-V4714/2003	West Nile virus	HQ705660	10608	Crow	USA
WNV-1/US/BID-V4715/2003	West Nile virus	HQ671723	10608	Crow	USA
WNV-1/US/BID-V4716/2003	West Nile virus	HM756668	10608	Avian	USA
WNV-1/US/BID-V4717/2003	West Nile virus	HM488250	10611	Avian	USA
WNV-1/US/BID-V4718/2003	West Nile virus	HM756669	10413	Avian	USA
WNV-1/US/BID-V4719/2003	West Nile virus	HM488251	10612	Avian	USA
WNV-1/US/BID-V4720/2003	West Nile virus	HM756670	10612	Avian	USA
WNV-1/US/BID-V4798/2004	West Nile virus	HM756671	10612	Avian	USA
WNV-1/US/BID-V4799/2004	West Nile virus	HM756672	10454	Avian	USA
WNV-1/US/BID-V4800/2004	West Nile virus	JF899528	10610	Crow	USA
WNV-1/US/BID-V4801/2004	West Nile virus	HM756673	10612	Avian	USA
WNV-1/US/BID-V4803/2004	West Nile virus	JN367277	10612	Crow	USA
WNV-1/US/BID-V4805/2005	West Nile virus	HM488252	10611	Avian	USA
WNV-1/US/BID-V4806/2005	West Nile virus	HM756675	10612	Avian	USA
WNV-1/US/BID-V4808/2005	West Nile virus	JF899529	10613	Crow	USA
WNV-1/US/BID-V4883/2005	West Nile virus	HQ671724	10389	Crow	USA
WNV-1/US/BID-V4885/2005	West Nile virus	HQ671725	10613	Crow	USA
WNV-1/US/BID-V4887/2005	West Nile virus	HQ671726	10613	Crow	USA
WNV-1/US/BID-V4889/2006	West Nile virus	HQ671727	10553	Crow	USA
WNV-1/US/BID-V4891/2006	West Nile virus	HQ671728	10614	Crow	USA
WNV-1/US/BID-V4892/2006	West Nile virus	HQ671729	10613	Crow	USA
WNV-1/US/BID-V4896/2006	West Nile virus	JN183888	10635	Crow	USA
WNV-1/US/BID-V4897/2007	West Nile virus	HQ671730	10609	Crow	USA
WNV-1/US/BID-V4898/1999	West Nile virus	HQ671706	10614	Mosquito	USA
WNV-1/US/BID-V4899/1999	West Nile virus	HQ671707	10607	Mosquito	USA
WNV-1/US/BID-V4900/2000	West Nile virus	HQ671708	10613	Mosquito	USA
WNV-1/US/BID-V4901/2000	West Nile virus	HQ671709	10488	Mosquito	USA
WNV-1/US/BID-V4902/2000	West Nile virus	HQ671710	10612	Mosquito	USA
WNV-1/US/BID-V4903/2000	West Nile virus	HQ671711	10613	Mosquito	USA
WNV-1/US/BID-V4904/2000	West Nile virus	HQ671712	10613	Mosquito	USA
WNV-1/US/BID-V4905/2001	West Nile virus	HQ671713	10677	Crow	USA
WNV-1/US/BID-V4906/2001	West Nile virus	HQ671714	10613	Mosquito	USA
WNV-1/US/BID-V4907/2001	West Nile virus	JF920307	10613	Mosquito	USA
WNV-1/US/BID-V4908/2001	West Nile virus	HQ671715	10614	Mosquito	USA
WNV-1/US/BID-V4909/2001	West Nile virus	HQ671716	10610	Mosquito	USA
WNV-1/US/BID-V4910/2001	West Nile virus	HQ671717	10614	Mosquito	USA
WNV-1/US/BID-V4911/2001	West Nile virus	HQ671718	10614	Mosquito	USA
WNV-1/US/BID-V4912/2001	West Nile virus	HQ671719	10612	Mosquito	USA
WNV-1/US/BID-V4913/2002	West Nile virus	HQ671720	10737	Crow	USA
WNV-1/US/BID-V5147/2007	West Nile virus	JF730042	10414	Crow	USA
WNV-1/US/BID-V5148/2007	West Nile virus	JF488097	10619	Crow	USA
WNV-1/US/BID-V5150/2004	West Nile virus	JF488094	10622	Crow	USA
WNV-1/US/BID-V5157/2009	West Nile virus	JF488095	10622	Crow	USA
WNV-1/US/BID-V5159/2009	West Nile virus	JF488096	10579	Crow	USA
WNV-1/US/BID-V5170/2002	West Nile virus	JF730043	10743	Mosquito	USA
WNV-1/US/BID-V5176/2004	West Nile virus	JF488086	10981	Mosquito	USA
WNV-1/US/BID-V5177/2004	West Nile virus	JF488087	10828	Mosquito	USA

WNV-1/US/BID-V5178/2004	West Nile virus	JF488088	11355	Mosquito	USA
WNV-1/US/BID-V5179/2004	West Nile virus	JF488089	10620	Mosquito	USA
WNV-1/US/BID-V5180/2004	West Nile virus	JF488090	10783	Mosquito	USA
WNV-1/US/BID-V5181/2004	West Nile virus	JF488091	10841	Mosquito	USA
WNV-1/US/BID-V5182/2004	West Nile virus	JF488092	10769	Mosquito	USA
WNV-1/US/BID-V5188/2005	West Nile virus	JF488093	10990	Mosquito	USA
WNV-1/US/BID-V5196/2006	West Nile virus	JF920729	10622	Mosquito	USA
WNV-1/US/BID-V5197/2006	West Nile virus	JF920730	10695	Mosquito	USA
WNV-1/US/BID-V5201/2006	West Nile virus	JF920731	10778	Mosquito	USA
WNV-1/US/BID-V5202/2006	West Nile virus	JF920732	10811	Mosquito	USA
WNV-1/US/BID-V5203/2006	West Nile virus	JF920733	10622	Mosquito	USA
WNV-1/US/BID-V5204/2006	West Nile virus	JF920734	10492	Mosquito	USA
WNV-1/US/BID-V5205/2006	West Nile virus	JF920735	10625	Mosquito	USA
WNV-1/US/BID-V5206/2006	West Nile virus	JF920736	10796	Mosquito	USA
WNV-1/US/BID-V5207/2006	West Nile virus	JF920737	10513	Mosquito	USA
WNV-1/US/BID-V5208/2007	West Nile virus	JF920738	10621	Mosquito	USA
WNV-1/US/BID-V5209/2007	West Nile virus	JF920739	10791	Mosquito	USA
WNV-1/US/BID-V5210/2007	West Nile virus	JF920740	10512	Mosquito	USA
WNV-1/US/BID-V5212/2007	West Nile virus	JF920741	10622	Mosquito	USA
WNV-1/US/BID-V5213/2007	West Nile virus	JF920742	10622	Mosquito	USA
WNV-1/US/BID-V5214/2007	West Nile virus	JF920743	10466	Mosquito	USA
WNV-1/US/BID-V5215/2007	West Nile virus	JF920744	10623	Mosquito	USA
WNV-1/US/BID-V5216/2007	West Nile virus	JF920745	10622	Mosquito	USA
WNV-1/US/BID-V5217/2007	West Nile virus	JF920746	10511	Mosquito	USA
WNV-1/US/BID-V5218/2008	West Nile virus	JF920747	10968	Mosquito	USA
WNV-1/US/BID-V5219/2008	West Nile virus	JF920748	10497	Mosquito	USA
WNV-1/US/BID-V5220/2008	West Nile virus	JF920749	10622	Mosquito	USA
WNV-1/US/BID-V5222/2008	West Nile virus	JF920750	10622	Mosquito	USA
WNV-1/US/BID-V5223/2008	West Nile virus	JF920751	10622	Mosquito	USA
WNV-1/US/BID-V5224/2008	West Nile virus	JF920752	10569	Mosquito	USA
WNV-1/US/BID-V5225/2008	West Nile virus	JF920753	10622	Mosquito	USA
WNV-1/US/BID-V5226/2008	West Nile virus	JF920754	10620	Mosquito	USA
WNV-1/US/BID-V5227/2008	West Nile virus	JF920755	10622	Mosquito	USA
WNV-1/US/BID-V5228/2008	West Nile virus	JF972636	10623	Mosquito	USA
WNV-1/US/BID-V5229/2008	West Nile virus	JF920756	10622	Mosquito	USA
WNV-1/US/BID-V5230/2008	West Nile virus	JF920757	10622	Mosquito	USA
WNV-1/US/BID-V5233/2009	West Nile virus	JF920758	10621	Mosquito	USA
WNV-1/US/BID-V5234/2009	West Nile virus	JF920759	10616	Mosquito	USA
WNV-1/US/BID-V5235/2009	West Nile virus	JF920760	10497	Mosquito	USA
WNV-1/US/BID-V6056/2010	West Nile virus	KJ145827	10566	Crow	USA
WNV-1/US/BID-V6057/2010	West Nile virus	KJ145828	10616	Crow	USA
WNV-1/US/BID-V6058/2010	West Nile virus	KJ145829	10455	Crow	USA
WNV-1/US/BID-V6171/2010	West Nile virus	KJ501438	11028	Eagle	USA
WNV-1/US/BID-V6173/2001	West Nile virus	KJ501233	10957	Blue Jay	USA
WNV-1/US/BID-V6175/2001	West Nile virus	KJ501234	11028	Blue Jay	USA
WNV-1/US/BID-V6176/2001	West Nile virus	KJ501235	10913	Blue Jay	USA
WNV-1/US/BID-V6178/2008	West Nile virus	KJ501237	11028	Pelican	USA
WNV-1/US/BID-V6179/2008	West Nile virus	KJ501238	11026	Gull	USA
WNV-1/US/BID-V6182/2008	West Nile virus	KJ501239	10445	Cormorant	USA
WNV-1/US/BID-V6183/2008	West Nile virus	KJ501240	10979	Cormorant	USA
WNV-1/US/BID-V6184/2009	West Nile virus	KJ501241	10975	Pelican	USA
WNV-1/US/BID-V6185/2010	West Nile virus	KJ501242	10896	Pelican	USA
WNV-1/US/BID-V6187/2001	West Nile virus	KJ501243	10979	Crow	USA
WNV-1/US/BID-V6188/2001	West Nile virus	KJ501244	11028	Crow	USA
WNV-1/US/BID-V6189/2001	West Nile virus	KJ501245	10979	Crow	USA
WNV-1/US/BID-V6190/2001	West Nile virus	KJ501246	10962	Crow	USA
WNV-1/US/BID-V6191/2001	West Nile virus	KJ501247	10957	Crow	USA
WNV-1/US/BID-V6192/2001	West Nile virus	KJ501248	10966	Crow	USA
WNV-1/US/BID-V6193/2001	West Nile virus	KJ501249	10977	Crow	USA
WNV-1/US/BID-V6194/2001	West Nile virus	KJ501439	11028	Crow	USA
WNV-1/US/BID-V6196/2009	West Nile virus	KJ501250	10940	Pelican	USA
WNV-1/US/BID-V6197/2009	West Nile virus	KJ501251	11029	Crow	USA
WNV-1/US/BID-V6200/2008	West Nile virus	KJ501253	10979	Tern	USA
WNV-1/US/BID-V6203/2008	West Nile virus	KJ501254	10979	Goose	USA
WNV-1/US/BID-V6204/2008	West Nile virus	KJ501255	10929	Pelican	USA
WNV-1/US/BID-V6207/2009	West Nile virus	KJ501256	10979	Pelican	USA
WNV-1/US/BID-V6208/2002	West Nile virus	KJ501257	11032	Crow	USA
WNV-1/US/BID-V6209/2007	West Nile virus	KJ501258	10979	Tern	USA
WNV-1/US/BID-V6210/2008	West Nile virus	KJ501259	10951	Tern	USA
WNV-1/US/BID-V6211/2008	West Nile virus	KJ501260	10959	Pelican	USA
WNV-1/US/BID-V6212/2008	West Nile virus	KJ501440	11003	Pelican	USA
WNV-1/US/BID-V6213/2008	West Nile virus	KJ501441	10979	Hawk	USA
WNV-1/US/BID-V6215/2010	West Nile virus	KJ501262	10951	Duck	USA
WNV-1/US/BID-V6371/2010	West Nile virus	KJ501442	11029	Eagle	USA
WNV-1/US/BID-V6372/2002	West Nile virus	KJ501263	11029	Blue Jay	USA
WNV-1/US/BID-V6373/2001	West Nile virus	KJ501443	11028	Crow	USA
WNV-1/US/BID-V6374/2001	West Nile virus	KJ501264	10979	Blue Jay	USA
WNV-1/US/BID-V6375/2002	West Nile virus	KJ501265	11034	Blue Jay	USA
WNV-1/US/BID-V6378/2005	West Nile virus	KJ501267	10968	Pelican	USA
WNV-1/US/BID-V6379/2001	West Nile virus	KJ501268	10984	Falcon	USA
WNV-1/US/BID-V6380/2001	West Nile virus	KJ501269	10989	Merlin	USA
WNV-1/US/BID-V6381/2001	West Nile virus	KJ501445	10990	Crow	USA
WNV-1/US/BID-V6382/2002	West Nile virus	KJ501270	10979	Blue Jay	USA
WNV-1/US/BID-V6384/2003	West Nile virus	KJ501271	10978	Owl	USA
WNV-1/US/BID-V6386/2003	West Nile virus	KJ501446	10984	Owl	USA
WNV-1/US/BID-V6387/2003	West Nile virus	KJ501272	10957	Blue Jay	USA
WNV-1/US/BID-V6388/2003	West Nile virus	KJ501447	10984	Crow	USA
WNV-1/US/BID-V6389/2003	West Nile virus	KJ501273	10997	Blue Jay	USA
WNV-1/US/BID-V6390/2001	West Nile virus	KJ501274	10953	Crow	USA
WNV-1/US/BID-V6391/2001	West Nile virus	KJ501275	10989	Crow	USA
WNV-1/US/BID-V6392/2002	West Nile virus	KJ501276	10917	Owl	USA
WNV-1/US/BID-V6393/2002	West Nile virus	KJ501448	11029	Crow	USA
WNV-1/US/BID-V6394/2001	West Nile virus	KJ501277	10979	Crow	USA
WNV-1/US/BID-V6396/2001	West Nile virus	KJ501278	10978	Crow	USA
WNV-1/US/BID-V6398/2002	West Nile virus	KJ501449	10987	Blue Jay	USA
WNV-1/US/BID-V6399/2002	West Nile virus	KJ501450	10979	Crow	USA
WNV-1/US/BID-V6401/2002	West Nile virus	KJ501279	11029	Blue Jay	USA
WNV-1/US/BID-V6402/2003	West Nile virus	KJ501451	10980	Crow	USA
WNV-1/US/BID-V6403/2003	West Nile virus	KJ501280	10988	Crow	USA
WNV-1/US/BID-V6404/2002	West Nile virus	KJ501281	10976	Blue Jay	USA
WNV-1/US/BID-V6405/2001	West Nile virus	KJ501452	10979	Crow	USA
WNV-1/US/BID-V6406/2001	West Nile virus	KJ501282	10991	Owl	USA
WNV-1/US/BID-V6407/2001	West Nile virus	KJ501453	10980	Crow	USA
WNV-1/US/BID-V6408/2001	West Nile virus	KJ501454	10979	Crow	USA
WNV-1/US/BID-V6409/2001	West Nile virus	KJ501283	10962	Crow	USA
WNV-1/US/BID-V6410/2002	West Nile virus	KJ501284	10972	Blue Jay	USA
WNV-1/US/BID-V6412/2002	West Nile virus	KJ501285	11016	Pelican	USA
WNV-1/US/BID-V6413/2002	West Nile virus	KJ501286	10979	Northern Goshawk	USA
WNV-1/US/BID-V6418/2001	West Nile virus	KJ501455	10979	Blue Jay	USA
WNV-1/US/BID-V6419/2002	West Nile virus	KJ501287	10979	Blue Jay	USA
WNV-1/US/BID-V6420/2001	West Nile virus	KJ501288	10962	Crow	USA
WNV-1/US/BID-V6422/2001	West Nile virus	KJ501289	10979	Crow	USA
WNV-1/US/BID-V6423/2002	West Nile virus	KJ501290	11019	Crow	USA

WNV-1/US/BID-V6424/2002	West Nile virus	KJ501291	11029	Crow	USA
WNV-1/US/BID-V6425/2001	West Nile virus	KJ501292	10986	Crow	USA
WNV-1/US/BID-V6426/2003	West Nile virus	KJ501456	11034	Crow	USA
WNV-1/US/BID-V6427/2003	West Nile virus	KJ501293	10979	Merlin	USA
WNV-1/US/BID-V6428/2003	West Nile virus	KJ501294	10975	Crow	USA
WNV-1/US/BID-V6429/2003	West Nile virus	KJ501457	10995	Crow	USA
WNV-1/US/BID-V6431/2002	West Nile virus	KJ501295	10982	Blue Jay	USA
WNV-1/US/BID-V6432/2002	West Nile virus	KJ501458	10979	Blue Jay	USA
WNV-1/US/BID-V6433/2002	West Nile virus	KJ501296	11029	Magpie	USA
WNV-1/US/BID-V6434/2003	West Nile virus	KJ501297	10979	Pelican	USA
WNV-1/US/BID-V6436/2002	West Nile virus	KJ501298	11029	Blue Jay	USA
WNV-1/US/BID-V6438/2002	West Nile virus	KJ501299	10989	Crow	USA
WNV-1/US/BID-V6439/2003	West Nile virus	KJ501460	11003	Squirrel	USA
WNV-1/US/BID-V6440/2005	West Nile virus	KJ501461	10995	Shorebird	USA
WNV-1/US/BID-V6441/2002	West Nile virus	KJ501462	10953	Hawk	USA
WNV-1/US/BID-V6442/2002	West Nile virus	KJ501463	10982	Crow	USA
WNV-1/US/BID-V6443/2002	West Nile virus	KJ501300	11029	Blue Jay	USA
WNV-1/US/BID-V6444/2002	West Nile virus	KJ501301	10984	Blue Jay	USA
WNV-1/US/BID-V6445/2002	West Nile virus	KJ501302	11029	Grackle	USA
WNV-1/US/BID-V6446/2002	West Nile virus	KJ501303	11003	Blue Jay	USA
WNV-1/US/BID-V6447/2002	West Nile virus	KJ501304	10997	Crow	USA
WNV-1/US/BID-V6448/2002	West Nile virus	KJ501464	10962	Blue Jay	USA
WNV-1/US/BID-V6449/2003	West Nile virus	KJ501305	11021	Crow	USA
WNV-1/US/BID-V6450/2001	West Nile virus	KJ501465	10979	Crow	USA
WNV-1/US/BID-V6451/2002	West Nile virus	KJ501466	11031	Owl	USA
WNV-1/US/BID-V6452/2002	West Nile virus	KJ501306	10992	Hawk	USA
WNV-1/US/BID-V6453/2002	West Nile virus	KJ501467	10984	Owl	USA
WNV-1/US/BID-V6456/2010	West Nile virus	KJ501308	11026	Pelican	USA
WNV-1/US/BID-V6458/2001	West Nile virus	KJ501468	11028	Starling	USA
WNV-1/US/BID-V6459/2002	West Nile virus	KJ501309	11024	Blue Jay	USA
WNV-1/US/BID-V6460/2002	West Nile virus	KJ501310	11029	Parrot	USA
WNV-1/US/BID-V6461/2002	West Nile virus	KJ501311	11000	Blue Jay	USA
WNV-1/US/BID-V6462/2003	West Nile virus	KJ501469	10975	Shorebird	USA
WNV-1/US/BID-V6463/2003	West Nile virus	KJ501470	11029	Gull	USA
WNV-1/US/BID-V6464/2003	West Nile virus	KJ501312	10981	Crow	USA
WNV-1/US/BID-V6465/2005	West Nile virus	KJ501471	10984	Eagle	USA
WNV-1/US/BID-V6466/2003	West Nile virus	KJ501313	11024	Blackbird	USA
WNV-1/US/BID-V6467/2003	West Nile virus	KJ501314	11034	Finch	USA
WNV-1/US/BID-V6468/2003	West Nile virus	KJ501472	11026	Crow	USA
WNV-1/US/BID-V6469/2001	West Nile virus	KJ501473	10978	Crow	USA
WNV-1/US/BID-V6470/2002	West Nile virus	KJ501474	11028	Owl	USA
WNV-1/US/BID-V6471/2002	West Nile virus	KJ501475	11031	Blue Jay	USA
WNV-1/US/BID-V6472/2002	West Nile virus	KJ501315	11029	Crow	USA
WNV-1/US/BID-V6473/2002	West Nile virus	KJ501476	10979	Magpie	USA
WNV-1/US/BID-V6474/2003	West Nile virus	KJ501477	11018	Crow	USA
WNV-1/US/BID-V6475/2003	West Nile virus	KJ501478	10979	Blue Jay	USA
WNV-1/US/BID-V6477/2002	West Nile virus	KJ501479	10960	Sparrow	USA
WNV-1/US/BID-V6478/2001	West Nile virus	KJ501480	11024	Crow	USA
WNV-1/US/BID-V6479/2001	West Nile virus	KJ501317	10984	Crow	USA
WNV-1/US/BID-V6480/2002	West Nile virus	KJ501318	11026	Crow	USA
WNV-1/US/BID-V6481/2002	West Nile virus	KJ501319	10984	Crow	USA
WNV-1/US/BID-V6482/2003	West Nile virus	KJ501320	11007	Crow	USA
WNV-1/US/BID-V6483/2002	West Nile virus	KJ501481	11033	Crow	USA
WNV-1/US/BID-V6484/2002	West Nile virus	KJ501321	10951	Crow	USA
WNV-1/US/BID-V6485/2002	West Nile virus	KJ501322	11026	Pelican	USA
WNV-1/US/BID-V6486/2003	West Nile virus	KJ501323	10995	Pelican	USA
WNV-1/US/BID-V6487/2003	West Nile virus	KJ501324	11033	Pelican	USA
WNV-1/US/BID-V6489/2003	West Nile virus	KJ501326	10990	Pelican	USA
WNV-1/US/BID-V6490/2003	West Nile virus	KJ501327	11019	Eagle	USA
WNV-1/US/BID-V6491/2002	West Nile virus	KJ501482	10960	Blue Jay	USA
WNV-1/US/BID-V6492/2002	West Nile virus	KJ501483	11028	Blue Jay	USA
WNV-1/US/BID-V6493/2002	West Nile virus	KJ501484	10979	Crow	USA
WNV-1/US/BID-V6495/2006	West Nile virus	KJ501485	10989	Pelican	USA
WNV-1/US/BID-V6496/2001	West Nile virus	KJ501486	11031	Hawk	USA
WNV-1/US/BID-V6497/2005	West Nile virus	KJ501487	10978	Crow	USA
WNV-1/US/BID-V6498/2002	West Nile virus	KJ501328	10951	Hawk	USA
WNV-1/US/BID-V6499/2003	West Nile virus	KJ501488	11029	Pelican	USA
WNV-1/US/BID-V6500/2003	West Nile virus	KJ501329	11010	Goose	USA
WNV-1/US/BID-V6501/2005	West Nile virus	KJ501330	10989	Tern	USA
WNV-1/US/BID-V6502/2002	West Nile virus	KJ501331	10960	Hawk	USA
WNV-1/US/BID-V6505/2001	West Nile virus	KJ501332	10995	Crow	USA
WNV-1/US/BID-V6506/2002	West Nile virus	KJ501489	11010	Crow	USA
WNV-1/US/BID-V6507/2002	West Nile virus	KJ501490	10953	Crow	USA
WNV-1/US/BID-V6508/2002	West Nile virus	KJ501491	11019	Crow	USA
WNV-1/US/BID-V6510/2002	West Nile virus	KJ501333	10913	Blue Jay	USA
WNV-1/US/BID-V6512/2005	West Nile virus	KJ501492	10973	Pelican	USA
WNV-1/US/BID-V6513/2005	West Nile virus	KJ501335	10995	Pelican	USA
WNV-1/US/BID-V6517/2001	West Nile virus	KJ501336	10979	Blue Jay	USA
WNV-1/US/BID-V6518/2002	West Nile virus	KJ501337	10980	Crow	USA
WNV-1/US/BID-V6519/2002	West Nile virus	KJ501338	10795	Blue Jay	USA
WNV-1/US/BID-V6521/2002	West Nile virus	KJ501339	10979	Blue Jay	USA
WNV-1/US/BID-V6522/2003	West Nile virus	KJ501340	10959	Grackle	USA
WNV-1/US/BID-V6523/2005	West Nile virus	KJ501341	10948	Finch	USA
WNV-1/US/BID-V6524/2005	West Nile virus	KJ501342	10975	Vulture	USA
WNV-1/US/BID-V6526/2001	West Nile virus	KJ501493	10978	Mockingbird	USA
WNV-1/US/BID-V6527/2001	West Nile virus	KJ501343	11031	Crow	USA
WNV-1/US/BID-V6528/2001	West Nile virus	KJ501494	10979	Crow	USA
WNV-1/US/BID-V6529/2002	West Nile virus	KJ501344	11017	Crow	USA
WNV-1/US/BID-V6530/2002	West Nile virus	KJ501495	10989	Blue Jay	USA
WNV-1/US/BID-V6531/2001	West Nile virus	KJ501496	11026	Blue Jay	USA
WNV-1/US/BID-V6532/2001	West Nile virus	KJ501497	10985	Accipitridae	USA
WNV-1/US/BID-V6534/2003	West Nile virus	KJ501498	10980	Sparrow	USA
WNV-1/US/BID-V6535/2003	West Nile virus	KJ501499	10980	Merlin	USA
WNV-1/US/BID-V6536/2009	West Nile virus	KJ501500	10977	Gull	USA
WNV-1/US/BID-V6538/2002	West Nile virus	KJ501501	11000	Crow	USA
WNV-1/US/BID-V6539/2002	West Nile virus	KJ501345	10979	Crow	USA
WNV-1/US/BID-V6541/2003	West Nile virus	KJ501346	10986	Pelican	USA
WNV-1/US/BID-V6542/2002	West Nile virus	KJ501502	11033	Crow	USA
WNV-1/US/BID-V6543/2002	West Nile virus	KJ501347	10976	Crow	USA
WNV-1/US/BID-V6544/2003	West Nile virus	KJ501348	10972	Pelican	USA
WNV-1/US/BID-V6545/2002	West Nile virus	KJ501503	10985	Blackbird	USA
WNV-1/US/BID-V6546/2003	West Nile virus	KJ501349	10982	Gull	USA
WNV-1/US/BID-V6547/2003	West Nile virus	KJ501350	10955	Pelican	USA
WNV-1/US/BID-V6548/2003	West Nile virus	KJ501504	10978	Goose	USA
WNV-1/US/BID-V6550/2002	West Nile virus	KJ501505	10953	Crow	USA
WNV-1/US/BID-V6551/2003	West Nile virus	KJ501351	10952	Crow	USA
WNV-1/US/BID-V6552/2003	West Nile virus	KJ501352	10977	Crow	USA
WNV-1/US/BID-V6553/2003	West Nile virus	KJ501506	10974	Crow	USA
WNV-1/US/BID-V6554/2003	West Nile virus	KJ501353	10978	Crow	USA
WNV-1/US/BID-V6555/2003	West Nile virus	KJ501354	10990	Crow	USA
WNV-1/US/BID-V6556/2001	West Nile virus	KJ501507	10978	Pelican	USA
WNV-1/US/BID-V6558/2002	West Nile virus	KJ501355	10972	Blue Jay	USA
WNV-1/US/BID-V6559/2003	West Nile virus	KJ501356	10977	Blue Jay	USA

WNV-1/US/BID-V6560/2003	West Nile virus	KJ501508	10985	Dove	USA
WNV-1/US/BID-V6561/2003	West Nile virus	KJ501357	10951	Owl	USA
WNV-1/US/BID-V6562/2003	West Nile virus	KJ501358	10976	Hawk	USA
WNV-1/US/BID-V6563/2003	West Nile virus	KJ501359	10985	Crow	USA
WNV-1/US/BID-V6565/2003	West Nile virus	KJ501360	10988	Blue Jay	USA
WNV-1/US/BID-V6566/2001	West Nile virus	KJ501361	10990	Blue Jay	USA
WNV-1/US/BID-V6567/2001	West Nile virus	KJ501362	10978	Crow	USA
WNV-1/US/BID-V6568/2001	West Nile virus	KJ501363	11029	Crow	USA
WNV-1/US/BID-V6569/2002	West Nile virus	KJ501364	10979	Crow	USA
WNV-1/US/BID-V6570/2003	West Nile virus	KJ501365	10979	Crow	USA
WNV-1/US/BID-V6571/2003	West Nile virus	KJ501366	10989	Crow	USA
WNV-1/US/BID-V6573/2002	West Nile virus	KJ501368	10953	Crow	USA
WNV-1/US/BID-V6574/2002	West Nile virus	KJ501369	10713	Sparrow	USA
WNV-1/US/BID-V6575/2001	West Nile virus	KJ501509	10978	Blackbird	USA
WNV-1/US/BID-V6584/2012	West Nile virus	KJ501211	10834	Mosquito	USA
WNV-1/US/BID-V6589/2012	West Nile virus	KJ501214	10891	Mosquito	USA
WNV-1/US/BID-V6597/2012	West Nile virus	KJ501217	10957	Mosquito	USA
WNV-1/US/BID-V6598/2012	West Nile virus	KJ501218	10557	Mosquito	USA
WNV-1/US/BID-V6602/2012	West Nile virus	KJ501220	11010	Mosquito	USA
WNV-1/US/BID-V6610/2002	West Nile virus	KJ501370	11029	Blue Jay	USA
WNV-1/US/BID-V6611/2002	West Nile virus	KJ501371	11029	Blue Jay	USA
WNV-1/US/BID-V6612/2002	West Nile virus	KJ501510	11029	Blue Jay	USA
WNV-1/US/BID-V6613/2002	West Nile virus	KJ501511	11029	Blue Jay	USA
WNV-1/US/BID-V6615/2003	West Nile virus	KJ501372	11028	Vulture	USA
WNV-1/US/BID-V6616/2001	West Nile virus	KJ501512	10998	Crow	USA
WNV-1/US/BID-V6617/2002	West Nile virus	KJ501373	11029	Blue Jay	USA
WNV-1/US/BID-V6619/2002	West Nile virus	KJ501514	10979	Crow	USA
WNV-1/US/BID-V6620/2001	West Nile virus	KJ501515	10979	Hawk	USA
WNV-1/US/BID-V6622/2001	West Nile virus	KJ501374	10979	Hawk	USA
WNV-1/US/BID-V6623/2002	West Nile virus	KJ501375	10979	Crow	USA
WNV-1/US/BID-V6625/2001	West Nile virus	KJ501516	11029	Crow	USA
WNV-1/US/BID-V6626/2002	West Nile virus	KJ501517	10979	Crow	USA
WNV-1/US/BID-V6627/2002	West Nile virus	KJ501376	10462	Owl	USA
WNV-1/US/BID-V6628/2001	West Nile virus	KJ501377	10979	Owl	USA
WNV-1/US/BID-V6629/2001	West Nile virus	KJ501378	10970	Crow	USA
WNV-1/US/BID-V6630/2001	West Nile virus	KJ501379	10979	Blue Jay	USA
WNV-1/US/BID-V6631/2002	West Nile virus	KJ501380	11029	Owl	USA
WNV-1/US/BID-V6632/2003	West Nile virus	KJ501381	10976	Pelican	USA
WNV-1/US/BID-V6633/2003	West Nile virus	KJ501382	10977	Cormorant	USA
WNV-1/US/BID-V6634/2002	West Nile virus	KJ501383	10995	Blue Jay	USA
WNV-1/US/BID-V6635/2002	West Nile virus	KJ501384	11029	Blue Jay	USA
WNV-1/US/BID-V6637/2004	West Nile virus	KJ501385	11029	Hawk	USA
WNV-1/US/BID-V6639/2007	West Nile virus	KJ501386	10993	Pelican	USA
WNV-1/US/BID-V6640/2007	West Nile virus	KJ501518	10994	Pelican	USA
WNV-1/US/BID-V6642/2002	West Nile virus	KJ501387	10979	Blue Jay	USA
WNV-1/US/BID-V6643/2002	West Nile virus	KJ501388	11028	Blue Jay	USA
WNV-1/US/BID-V6645/2003	West Nile virus	KJ501389	10831	Pelican	USA
WNV-1/US/BID-V6646/2003	West Nile virus	KJ501390	10982	Pelican	USA
WNV-1/US/BID-V6647/2003	West Nile virus	KJ501391	10975	Pelican	USA
WNV-1/US/BID-V6648/2003	West Nile virus	KJ501392	10971	Pelican	USA
WNV-1/US/BID-V6649/2001	West Nile virus	KJ501393	10947	Crow	USA
WNV-1/US/BID-V6650/2001	West Nile virus	KJ501394	10985	Crow	USA
WNV-1/US/BID-V6651/2001	West Nile virus	KJ501395	11029	Crow	USA
WNV-1/US/BID-V6652/2002	West Nile virus	KJ501519	10973	Crow	USA
WNV-1/US/BID-V6653/2003	West Nile virus	KJ501396	10979	Crow	USA
WNV-1/US/BID-V6654/2002	West Nile virus	KJ501397	10978	Blue Jay	USA
WNV-1/US/BID-V6656/2004	West Nile virus	KJ501398	10991	Pelican	USA
WNV-1/US/BID-V6657/2002	West Nile virus	KJ501399	11029	Finch	USA
WNV-1/US/BID-V6658/2002	West Nile virus	KJ501400	10984	Sparrow	USA
WNV-1/US/BID-V6659/2001	West Nile virus	KJ501401	10979	Crow	USA
WNV-1/US/BID-V6661/2004	West Nile virus	KJ501520	10983	Pelican	USA
WNV-1/US/BID-V6662/2004	West Nile virus	KJ501402	10982	Pelican	USA
WNV-1/US/BID-V6663/2005	West Nile virus	KJ501403	11000	Pelican	USA
WNV-1/US/BID-V6664/2005	West Nile virus	KJ501404	11029	Pelican	USA
WNV-1/US/BID-V6665/2006	West Nile virus	KJ501405	10795	Pelican	USA
WNV-1/US/BID-V6666/2006	West Nile virus	KJ501406	11026	Pelican	USA
WNV-1/US/BID-V6667/2006	West Nile virus	KJ501407	10995	Pelican	USA
WNV-1/US/BID-V6668/2006	West Nile virus	KJ501408	10953	Pelican	USA
WNV-1/US/BID-V6669/2006	West Nile virus	KJ501409	11031	Pelican	USA
WNV-1/US/BID-V6670/2006	West Nile virus	KJ501410	10936	Swan	USA
WNV-1/US/BID-V6672/2006	West Nile virus	KJ501521	10976	Pelican	USA
WNV-1/US/BID-V6673/2008	West Nile virus	KJ501411	10953	Sparrow	USA
WNV-1/US/BID-V6674/2008	West Nile virus	KJ501412	10986	Sparrow	USA
WNV-1/US/BID-V6675/2004	West Nile virus	KJ501413	10964	Crow	USA
WNV-1/US/BID-V6676/2004	West Nile virus	KJ501522	10976	Crow	USA
WNV-1/US/BID-V6678/2005	West Nile virus	KJ501414	10975	Tern	USA
WNV-1/US/BID-V6680/2006	West Nile virus	KJ501523	10971	Tern	USA
WNV-1/US/BID-V6681/2006	West Nile virus	KJ501415	10951	Pelican	USA
WNV-1/US/BID-V6682/2006	West Nile virus	KJ501524	10979	Shorebird	USA
WNV-1/US/BID-V6683/2006	West Nile virus	KJ501416	10978	Shorebird	USA
WNV-1/US/BID-V6685/2008	West Nile virus	KJ501418	10979	Tern	USA
WNV-1/US/BID-V6686/2004	West Nile virus	KJ501419	11027	Pelican	USA
WNV-1/US/BID-V6687/2004	West Nile virus	KJ501420	10984	Pelican	USA
WNV-1/US/BID-V6688/2004	West Nile virus	KJ501421	10977	Pelican	USA
WNV-1/US/BID-V6689/2004	West Nile virus	KJ501525	11029	Pelican	USA
WNV-1/US/BID-V6690/2004	West Nile virus	KJ501422	10973	Pelican	USA
WNV-1/US/BID-V6691/2004	West Nile virus	KJ501423	10979	Pelican	USA
WNV-1/US/BID-V6692/2006	West Nile virus	KJ501526	10958	Tern	USA
WNV-1/US/BID-V6694/2007	West Nile virus	KJ501424	10979	Pelican	USA
WNV-1/US/BID-V6695/2007	West Nile virus	KJ501425	10979	Pelican	USA
WNV-1/US/BID-V6696/2003	West Nile virus	KJ501527	10988	Crow	USA
WNV-1/US/BID-V6698/2002	West Nile virus	KJ501426	10990	Crow	USA
WNV-1/US/BID-V6699/2002	West Nile virus	KJ501427	10979	Kite	USA
WNV-1/US/BID-V6700/2006	West Nile virus	KJ501428	10977	Cormorant	USA
WNV-1/US/BID-V6701/2006	West Nile virus	KJ501529	11029	Pelican	USA
WNV-1/US/BID-V6702/2006	West Nile virus	KJ501429	11029	Pelican	USA
WNV-1/US/BID-V6703/2006	West Nile virus	KJ501430	11029	Pelican	USA
WNV-1/US/BID-V6704/2006	West Nile virus	KJ501431	10868	Pelican	USA
WNV-1/US/BID-V7270/2012	West Nile virus	KJ501432	10936	Crow	USA
WNV-1/US/BID-V7277/2012	West Nile virus	KJ501434	10979	Pelican	USA
WNV-1/US/BID-V7281/2012	West Nile virus	KJ501530	10979	Goose	USA
WNV-1/US/BID-V7286/2012	West Nile virus	KJ501437	10978	Crow	USA
WNV-1/US/BID-V7383/2011	West Nile virus	KJ501096	11028	Mosquito	USA
WNV-1/US/BID-V7384/2008	West Nile virus	KJ501206	11012	Mosquito	USA
WNV-1/US/BID-V7386/2011	West Nile virus	KJ501098	11025	Mosquito	USA
WNV-1/US/BID-V7387/2009	West Nile virus	KJ501099	11028	Mosquito	USA
WNV-1/US/BID-V7388/2009	West Nile virus	KJ501100	11028	Mosquito	USA
WNV-1/US/BID-V7389/2009	West Nile virus	KJ501101	11002	Mosquito	USA
WNV-1/US/BID-V7390/2009	West Nile virus	KJ501102	10960	Mosquito	USA
WNV-1/US/BID-V7391/2003	West Nile virus	KJ501103	11030	Mosquito	USA
WNV-1/US/BID-V7392/2003	West Nile virus	KJ501104	10987	Mosquito	USA
WNV-1/US/BID-V7393/2003	West Nile virus	KJ501105	10891	Mosquito	USA
WNV-1/US/BID-V7394/2008	West Nile virus	KJ501106	10994	Mosquito	USA

	WNV-1/US/BID-V7395/2003	West Nile virus	KJ501107	11033	Mosquito	USA
	WNV-1/US/BID-V7396/2007	West Nile virus	KJ501108	10946	Mosquito	USA
	WNV-1/US/BID-V7397/2008	West Nile virus	KJ501109	11031	Mosquito	USA
	WNV-1/US/BID-V7398/2009	West Nile virus	KJ501110	11033	Mosquito	USA
	WNV-1/US/BID-V7399/2009	West Nile virus	KJ501111	10966	Mosquito	USA
	WNV-1/US/BID-V7400/2007	West Nile virus	KJ501112	10467	Mosquito	USA
	WNV-1/US/BID-V7401/2008	West Nile virus	KJ501113	10912	Mosquito	USA
	WNV-1/US/BID-V7402/2007	West Nile virus	KJ501114	11033	Mosquito	USA
	WNV-1/US/BID-V7403/2005	West Nile virus	KJ501115	10988	Mosquito	USA
	WNV-1/US/BID-V7404/2008	West Nile virus	KJ501116	11005	Mosquito	USA
	WNV-1/US/BID-V7407/2005	West Nile virus	KJ501119	11027	Mosquito	USA
	WNV-1/US/BID-V7411/2004	West Nile virus	KJ501123	11028	Mosquito	USA
	WNV-1/US/BID-V7414/2004	West Nile virus	KJ501125	11026	Mosquito	USA
	WNV-1/US/BID-V7415/2007	West Nile virus	KJ501126	10989	Mosquito	USA
	WNV-1/US/BID-V7416/2008	West Nile virus	KJ501127	11014	Mosquito	USA
	WNV-1/US/BID-V7417/2004	West Nile virus	KJ501128	10807	Mosquito	USA
	WNV-1/US/BID-V7418/2004	West Nile virus	KJ501129	10898	Mosquito	USA
	WNV-1/US/BID-V7419/2008	West Nile virus	KJ501130	11007	Mosquito	USA
	WNV-1/US/BID-V7420/2004	West Nile virus	KJ501131	11028	Mosquito	USA
	WNV-1/US/BID-V7421/2007	West Nile virus	KJ501132	10989	Mosquito	USA
	WNV-1/US/BID-V7422/2008	West Nile virus	KJ501133	11033	Mosquito	USA
	WNV-1/US/BID-V7423/2010	West Nile virus	KJ501134	11031	Mosquito	USA
	WNV-1/US/BID-V7425/2010	West Nile virus	KJ501135	11029	Mosquito	USA
	WNV-1/US/BID-V7426/2010	West Nile virus	KJ501136	10990	Mosquito	USA
	WNV-1/US/BID-V7427/2011	West Nile virus	KJ501137	10984	Mosquito	USA
	WNV-1/US/BID-V7428/2011	West Nile virus	KJ501138	11028	Mosquito	USA
	WNV-1/US/BID-V7429/2011	West Nile virus	KJ501139	10894	Mosquito	USA
	WNV-1/US/BID-V7430/2011	West Nile virus	KJ501140	11028	Mosquito	USA
	WNV-1/US/BID-V7431/2011	West Nile virus	KJ501141	10951	Mosquito	USA
	WNV-1/US/BID-V7432/2011	West Nile virus	KJ501142	11005	Mosquito	USA
	WNV-1/US/BID-V7433/2011	West Nile virus	KJ501143	11031	Mosquito	USA
	WNV-1/US/BID-V7434/2011	West Nile virus	KJ501144	10894	Mosquito	USA
	WNV-1/US/BID-V7436/2011	West Nile virus	KJ501146	10944	Mosquito	USA
	WNV-1/US/BID-V7437/2008	West Nile virus	KJ501147	10865	Mosquito	USA
	WNV-1/US/BID-V7438/2011	West Nile virus	KJ501148	11019	Mosquito	USA
	WNV-1/US/BID-V7441/2011	West Nile virus	KJ501149	11026	Mosquito	USA
	WNV-1/US/BID-V7442/2011	West Nile virus	KJ501150	11033	Mosquito	USA
	WNV-1/US/BID-V7463/2007	West Nile virus	KJ501168	11002	Mosquito	USA
	WNV-1/US/BID-V7465/2011	West Nile virus	KJ501170	10457	Mosquito	USA
	WNV-1/US/BID-V7484/2005	West Nile virus	KJ501186	11005	Mosquito	USA
	WNV-1/US/BID-V7494/2011	West Nile virus	KJ501195	10977	Mosquito	USA
	WNV-1/US/BID-V7773/2012	West Nile virus	KJ501222	11029	Mosquito	USA
	WNV-1/US/BID-V7788/2012	West Nile virus	KJ501224	11033	Mosquito	USA
	WNV-1/US/BID-V7789/2012	West Nile virus	KJ501225	11014	Mosquito	USA
	WNV-1/US/BID-V7790/2012	West Nile virus	KJ501230	11029	Mosquito	USA
	WNV-1/US/BID-V7791/2012	West Nile virus	KJ501226	11033	Mosquito	USA
	WNV-1/US/BID-V7795/2012	West Nile virus	KJ501229	11028	Mosquito	USA
	WNV-1/US/BID-V7815/2012	West Nile virus	KJ501531	10623	Mosquito	USA
	WNV-1/US/BID-V7816/2012	West Nile virus	KJ501532	10622	Mosquito	USA
	WNV-1/US/BID-V7817/2012	West Nile virus	KJ501533	10609	Mosquito	USA
	WNV-1/US/BID-V7818/2011	West Nile virus	KJ501534	10623	Mosquito	USA
	WNV-1/US/BID-V7819/2011	West Nile virus	KJ501535	10464	Mosquito	USA
	WNV-1/US/BID-V7820/2011	West Nile virus	KJ501536	10623	Mosquito	USA
	WNV-1/US/BID-V7821/2011	West Nile virus	KJ501537	10621	Mosquito	USA
	WNV-1/US/BID-V7822/2011	West Nile virus	KJ501538	10627	Mosquito	USA
	WNV-1/US/BID-V7823/2011	West Nile virus	KJ501539	10624	Mosquito	USA
	WNV/USA/BID-G15493	West Nile virus	JX503084	11174	Unknown	USA
	WNV/USA/BID-G15494	West Nile virus	JX503085	11243	Unknown	USA
	WNV/USA/BID-G15495	West Nile virus	JX503086	11186	Unknown	USA
	WNV/USA/BID-G15496	West Nile virus	JX503087	11237	Unknown	USA
	WNV/USA/BID-G15497	West Nile virus	JX503088	11201	Unknown	USA
	WNV/USA/BID-G15498	West Nile virus	JX503089	11185	Unknown	USA
	WNV/USA/BID-G15499	West Nile virus	JX503090	11187	Unknown	USA
	WNV/USA/BID-G15500	West Nile virus	JX503091	11187	Unknown	USA
	WNV/USA/BID-G15501	West Nile virus	JX503092	11191	Unknown	USA
	WNV/USA/BID-G15502	West Nile virus	JX503093	11178	Unknown	USA
	WNV/USA/BID-G15503	West Nile virus	JX503094	11183	Unknown	USA
	WNV/USA/BID-G15504	West Nile virus	JX503095	11244	Unknown	USA
	WNV/USA/BID-G15505	West Nile virus	JX503096	10954	Unknown	USA
	WNV/USA/BID-G15506	West Nile virus	JX503097	11231	Unknown	USA
	WNV/USA/BID-G15507	West Nile virus	JX503098	11183	Unknown	USA
	WNV/USA/BID-G15508	West Nile virus	JX503099	11195	Unknown	USA
	WNV_0043h_ISR00	West Nile virus	HM152773	10864	Human	Israel
	WNV_0304h_ISR00	West Nile virus	HM152775	10980	Human	Israel
	WNVCc	West Nile virus	DQ080060	10972	Raven	Mexico
	XJ11129	West Nile virus	JX442279	11029	Mosquito	China
	H442	West Nile virus	EF429200	11051	Human	South Africa
	SA381/00	West Nile virus	EF429199	11052	Human	South Africa
	SA93/01	West Nile virus	EF429198	11052	Human	South Africa
	SPU116/89	West Nile virus	EF429197	11052	Human	South Africa
	385-99	West Nile virus	AY842931	11029	Unknown	USA
	385-99	West Nile virus	AY848695	11029	Unknown	-N/A-
	385-99	West Nile virus	AY848696	11029	Unknown	-N/A-
	385-99	West Nile virus	AY848697	11029	Unknown	-N/A-
	385-99	West Nile virus	DQ066423	11029	Unknown	-N/A-
	PT5.2	West Nile virus	AJ965628	10977	Unknown	-N/A-
	PT6.16	West Nile virus	AJ965626	10979	Unknown	-N/A-
	West Nile virus isolate RT496, complete genome	West Nile virus	MG004541	10803	Unknown	-N/A-
**USUV**	1477	Usutu virus	KJ438705	11065	Mosquito	Germany
	2742	Usutu virus	KJ438706	11065	Mosquito	Germany
	2794	Usutu virus	KJ438767	11065	Mosquito	Germany
	2985	Usutu virus	KJ438707	11065	Mosquito	Germany
	3125	Usutu virus	KJ438708	11065	Mosquito	Germany
	3169	Usutu virus	KJ438718	11065	Mosquito	Germany
	3358	Usutu virus	KJ438721	11065	Mosquito	Germany
	4356	Usutu virus	KJ438737	11065	Mosquito	Germany
	5660	Usutu virus	KJ438709	11065	Mosquito	Germany
	5684	Usutu virus	KJ438716	11065	Mosquito	Germany
	6424	Usutu virus	KJ438736	11065	Mosquito	Germany
	6950	Usutu virus	KJ438768	11065	Mosquito	Germany
	ArB1803	Usutu virus	KC754958	10745	Mosquito	Central African Republic
	ArD101291	Usutu virus	KC754956	10837	Mosquito	Senegal
	ArD192495	Usutu virus	KC754957	10825	Mosquito	Senegal
	ArD19848	Usutu virus	KC754954	10837	Mosquito	Senegal
	BAT1USUTU-BNI	Usutu virus	KJ859682	11065	Bat	Germany
	BAT2USUTU-BNI	Usutu virus	KJ859683	11065	Bat	Germany
	BH65/11-02-03	Usutu virus	HE599647	11003	Blackbird	Germany
	Bonn	Usutu virus	KM659877	11065	Blackbird	Germany
	Budapest	Usutu virus	EF206350	11065	Blackbird	Hungary
	HB81P08	Usutu virus	KC754955	10800	Human	Central African Republic
	Italia 2009	Usutu virus	JF266698	11065	Blackbird	Italy
	Meise H	Usutu virus	JQ219843	11047	Blue Tit	Austria
	SAAR-1776	Usutu virus	AY453412	11064	Unknown	South Africa

	Spain MB119/06	Usutu virus	KF573410	11064	Mosquito	Spain
	V1	Usutu virus	KJ438719	11065	Blackbird	Germany
	V10	Usutu virus	KJ438760	11032	Blackbird	Germany
	V102	Usutu virus	KJ438728	11065	Blackbird	Germany
	V11	Usutu virus	KJ438738	11065	Blackbird	Germany
	V13	Usutu virus	KJ438750	11065	Blackbird	Germany
	V15	Usutu virus	KJ438727	11065	Blackbird	Germany
	V16	Usutu virus	KJ438751	11065	Blackbird	Germany
	V18	Usutu virus	KJ438730	11065	Blackbird	Germany
	V19	Usutu virus	KJ438710	11065	Blackbird	Germany
	V190	Usutu virus	KJ438744	11065	Blackbird	Germany
	V191	Usutu virus	KJ438779	11060	Blackbird	Germany
	V192	Usutu virus	KJ438733	11065	Blackbird	Germany
	V20	Usutu virus	KJ438769	11065	Blackbird	Germany
	V210	Usutu virus	KJ438763	11065	Blackbird	Germany
	V211	Usutu virus	KJ438764	11046	Blackbird	Germany
	V23	Usutu virus	KJ438732	11065	Blackbird	Germany
	V234	Usutu virus	KJ438749	11065	Blackbird	Germany
	V24	Usutu virus	KJ438752	11065	Blackbird	Germany
	V245	Usutu virus	KJ438747	11065	Blackbird	Germany
	V248	Usutu virus	KJ438774	11065	Blackbird	Germany
	V249	Usutu virus	KJ438748	11065	Blackbird	Germany
	V256	Usutu virus	KJ438722	11065	Blackbird	Germany
	V260	Usutu virus	KJ438735	11065	Blackbird	Germany
	V261	Usutu virus	KJ438717	11065	Blackbird	Germany
	V266	Usutu virus	KJ438729	11065	Blackbird	Germany
	V28	Usutu virus	KJ438777	11065	Blackbird	Germany
	V297	Usutu virus	KJ438754	11065	Blackbird	Germany
	V312	Usutu virus	KJ438771	11065	Blackbird	Germany
	V32	Usutu virus	KJ438753	11065	Blackbird	Germany
	V321	Usutu virus	KJ438766	11066	Blackbird	Germany
	V34	Usutu virus	KJ438725	11065	Blackbird	Germany
	V36	Usutu virus	KJ438726	11065	Blackbird	Germany
	V38	Usutu virus	KJ438745	11065	Blackbird	Germany
	V382	Usutu virus	KJ438758	11065	Blackbird	Germany
	V387	Usutu virus	KJ438757	11065	Blackbird	Germany
	V389	Usutu virus	KJ438713	11065	Blackbird	Germany
	V39	Usutu virus	KJ438765	11049	Blackbird	Germany
	V390	Usutu virus	KJ438741	11065	Blackbird	Germany
	V391	Usutu virus	KJ438739	11065	Blackbird	Germany
	V399	Usutu virus	KJ438742	11065	Blackbird	Germany
	V41	Usutu virus	KJ438743	11065	Blackbird	Germany
	V43	Usutu virus	KJ438731	11065	Blackbird	Germany
	V44	Usutu virus	KJ438780	11049	Blackbird	Germany
	V45	Usutu virus	KJ438770	11065	Blackbird	Germany
	V46	Usutu virus	KJ438755	11065	Thrush	Germany
	V49	Usutu virus	KJ438756	11065	Blackbird	Germany
	V5	Usutu virus	KJ438723	11065	Blackbird	Germany
	V50	Usutu virus	KJ438724	11065	Blackbird	Germany
	V51	Usutu virus	KJ438778	11065	Blackbird	Germany
	V52	Usutu virus	KJ438740	11065	Blackbird	Germany
	V53	Usutu virus	KJ438775	11065	Blackbird	Germany
	V54	Usutu virus	KJ438772	11065	Blackbird	Germany
	V58	Usutu virus	KJ438714	11065	Blackbird	Germany
	V60	Usutu virus	KJ438711	11065	Blackbird	Germany
	V66	Usutu virus	KJ438715	11065	Sparrow	Germany
	V68	Usutu virus	KJ438776	11065	Blackbird	Germany
	V69	Usutu virus	KJ438712	11065	Blackbird	Germany
	V70	Usutu virus	KJ438759	11065	Blackbird	Germany
	V72	Usutu virus	KJ438761	11065	Blackbird	Germany
	V82	Usutu virus	KJ438773	11065	Blackbird	Germany
	V83	Usutu virus	KJ438734	11065	Starling	Germany
	V84	Usutu virus	KJ438720	11065	Blackbird	Germany
	V86	Usutu virus	KJ438781	11065	Blackbird	Germany
	V9	Usutu virus	KJ438762	11065	Blackbird	Germany
	V93	Usutu virus	KJ438746	11065	Blackbird	Germany
	Vienna 2001	Usutu virus	AY453411	11066	Unknown	Austria
	Vienna 2001	Usutu virus	NC_006551 *	11066	Unknown	Austria
**SLEV**	79V-2533	St. Louis encephalitis virus	FJ753287	10936	Mosquito	Argentina
	Argentine 66	St. Louis encephalitis virus	AY632544	10293	Unknown	-N/A-
	BeH355964	St. Louis encephalitis virus	KM267635	10892	Human	Brazil
	CbaAr-4005	St. Louis encephalitis virus	FJ753286	10936	Mosquito	Argentina
	FLU3632	St. Louis encephalitis virus	KF589299	10839	Human	Peru
	Hubbard	St. Louis encephalitis virus	EU566860	10939	Human	USA
	Imperial Valley	St. Louis encephalitis virus	JF460774	10939	Mosquito	USA
	Kern217	St. Louis encephalitis virus	DQ525916	10940	Unknown	USA
	Kern217	St. Louis encephalitis virus	NC_007580 *	10940	Unknown	USA
	MSI-7	St. Louis encephalitis virus	DQ359217	10652	Unknown	USA
	Palenque-A770	St. Louis encephalitis virus	JQ957869	10938	Mosquito	Mexico
	Palenque-C475	St. Louis encephalitis virus	JQ957868	10913	Mosquito	Mexico
**YFV**	10A	Yellow fever virus	KM388816	10236	Monkey	Venezuela
	17D	Yellow fever virus	JX949181	10815	Unknown	USA
	17D Flavimun TVX	Yellow fever virus	JN628281	10862	Unknown	-N/A-
	17D Flavimun WSL	Yellow fever virus	JN628280	10862	Unknown	-N/A-
	17D RKI	Yellow fever virus	JN628279	10862	Unknown	-N/A-
	17D vaccine	Yellow fever virus	NC_002031 *	10862	Unknown	-N/A-
	17D vaccine	Yellow fever virus	X03700	10862	Unknown	-N/A-
	17D YF-VAX Vero adapted	Yellow fever virus	JN811140	10862	Unknown	-N/A-
	17D YF-VAX Vero adapted	Yellow fever virus	JN811141	10862	Unknown	-N/A-
	17D YF-VAX Vero adapted	Yellow fever virus	JN811142	10862	Unknown	-N/A-
	17D YF-VAX Vero adapted	Yellow fever virus	JN811143	10862	Unknown	-N/A-
	17D-204	Yellow fever virus	KF769015	10838	Unknown	-N/A-
	17D-204-South Africa vaccine	Yellow fever virus	AF052444	10862	Unknown	-N/A-
	17D-204-South Africa vaccine	Yellow fever virus	AF052445	10862	Unknown	-N/A-
	17D-204-South Africa vaccine	Yellow fever virus	AF052446	10862	Unknown	-N/A-
	17D-204-USA vaccine	Yellow fever virus	AF052437	10862	Unknown	-N/A-
	17D-204-USA vaccine	Yellow fever virus	AF052438	10862	Unknown	-N/A-
	17D-204-USA vaccine	Yellow fever virus	AF052439	10862	Unknown	-N/A-
	17DD-Brazil	Yellow fever virus	DQ100292	10862	Unknown	Brazil
	2A	Yellow fever virus	KM388817	10236	Monkey	Venezuela
	6A	Yellow fever virus	KM388814	10236	Human	Venezuela
	85-82H Ivory Coast	Yellow fever virus	U54798	10862	Unknown	-N/A-
	88/1999	Yellow fever virus	KF907504	10657	Human	Bolivia
	8A	Yellow fever virus	KM388818	10236	Monkey	Venezuela
	9A	Yellow fever virus	KM388815	10236	Monkey	Venezuela
	Angola71	Yellow fever virus	AY968064	10823	Human	Angola
	ArD114896	Yellow fever virus	JX898871	10859	Mosquito	Senegal
	ArD114972	Yellow fever virus	JX898872	10859	Mosquito	Senegal
	ArD121040	Yellow fever virus	JX898870	10862	Mosquito	Senegal
	ArD149194	Yellow fever virus	JX898874	10861	Mosquito	Senegal
	ArD149214	Yellow fever virus	JX898873	10862	Mosquito	Senegal
	ArD149815	Yellow fever virus	JX898875	10857	Mosquito	Senegal
	ArD156468	Yellow fever virus	JX898876	10859	Mosquito	Senegal

	ArD181250	Yellow fever virus	JX898878	10862	Mosquito	Senegal
	ArD181439	Yellow fever virus	JX898881	10862	Mosquito	Senegal
	ArD181464	Yellow fever virus	JX898877	10862	Mosquito	Senegal
	ArD181564	Yellow fever virus	JX898880	10862	Mosquito	Senegal
	ArD181676	Yellow fever virus	JX898879	10862	Mosquito	Senegal
	ASIBI	Yellow fever virus	AY640589	10862	Unknown	-N/A-
	ASIBI	Yellow fever virus	KF769016	10833	Unknown	-N/A-
	BeAr378600	Yellow fever virus	JF912179	10889	Mosquito	Brazil
	BeAr513008	Yellow fever virus	JF912185	11008	Mosquito	Brazil
	BeAR646536	Yellow fever virus	JF912189	11008	Mosquito	Brazil
	BeH394880	Yellow fever virus	JF912180	11008	Human	Brazil
	BeH413820	Yellow fever virus	JF912181	10795	Human	Brazil
	BeH422973	Yellow fever virus	JF912182	10860	Human	Brazil
	BeH423602	Yellow fever virus	JF912183	10852	Human	Brazil
	BeH463676	Yellow fever virus	JF912184	11004	Human	Brazil
	BeH526722	Yellow fever virus	JF912186	11004	Human	Brazil
	BeH622205	Yellow fever virus	JF912187	11008	Human	Brazil
	BeH622493	Yellow fever virus	JF912188	10817	Human	Brazil
	BeH655417	Yellow fever virus	JF912190	10859	Human	Brazil
	case #1	Yellow fever virus	GQ379162	10862	Human	Peru
	case #2	Yellow fever virus	GQ379163	10862	Human	Peru
	Couma	Yellow fever virus	DQ235229	10823	Unknown	Ethiopia
	DakArAmt7	Yellow fever virus	JX898869	10848	Mosquito	Cote dIvoire
	French neurotropic virus	Yellow fever virus	U21055	10862	Unknown	-N/A-
	French viscerotropic virus	Yellow fever virus	U21056	10862	Unknown	-N/A-
	Gambia 2001	Yellow fever virus	AY572535	10862	Unknown	-N/A-
	HD117294	Yellow fever virus	JX898868	10862	Human	Senegal
	Ivory Coast 1999	Yellow fever virus	AY603338	10862	Unknown	Cote dIvoire
	Pasteur 17D-204 yellow fever vaccine	Yellow fever virus	X15062	10862	Unknown	-N/A-
	patent WO2007093472_7	Yellow fever virus	CS805341	10862	Unknown	-N/A-
	Trinidad 79A	Yellow fever virus	AF094612	10760	Unknown	-N/A-
	TVP11767	Yellow fever virus	HM582851	10236	Monkey	Trinidad and Tobago
	Uganda 2010	Yellow fever virus	JN620362	10820	Human	Uganda
	Uganda48a	Yellow fever virus	AY968065	10823	Human	Uganda
	UNKNOWN-FU267585	Yellow fever virus	FU267585	10862	Unknown	-N/A-
	UNKNOWN-FV537409	Yellow fever virus	FV537409	10862	Unknown	-N/A-
	UNKNOWN-FV537410	Yellow fever virus	FV537410	10862	Unknown	-N/A-
	UNKNOWN-FV537411	Yellow fever virus	FV537411	10862	Unknown	-N/A-
	UNKNOWN-FV537412	Yellow fever virus	FV537412	10862	Unknown	-N/A-
	UNKNOWN-JA630638	Yellow fever virus	JA630638	11564	Unknown	-N/A-
	UNKNOWN-JA784080	Yellow fever virus	JA784080	10776	Unknown	-N/A-
	UNKNOWN-JA784081	Yellow fever virus	JA784081	10788	Unknown	-N/A-
	UNKNOWN-JA784082	Yellow fever virus	JA784082	10811	Unknown	-N/A-
	UNKNOWN-JA784083	Yellow fever virus	JA784083	10785	Unknown	-N/A-
	UNKNOWN-JA784086	Yellow fever virus	JA784086	10862	Unknown	-N/A-
	UNKNOWN-JB319328	Yellow fever virus	JB319328	10776	Unknown	-N/A-
	UNKNOWN-JB319329	Yellow fever virus	JB319329	10788	Unknown	-N/A-
	UNKNOWN-JB319330	Yellow fever virus	JB319330	10811	Unknown	-N/A-
	UNKNOWN-JB319331	Yellow fever virus	JB319331	10785	Unknown	-N/A-
	UNKNOWN-JB319334	Yellow fever virus	JB319334	10862	Unknown	-N/A-
	vaccine strain 17D-213	Yellow fever virus	U17067	10862	Unknown	-N/A-
	vaccine strain 17DD	Yellow fever virus	U17066	10862	Unknown	-N/A-
	YF-AVD2791-93F/04	Yellow fever virus	DQ118157	10862	Unknown	Spain
	YF/Vaccine/USA/Sanofi-Pasteur-17D- 204/UF795AA/YFVax	Yellow fever virus	JX503529	10811	Unknown	-N/A-
	17D-Tiantan	Yellow fever virus	FJ654700	10862	Unknown	China
**IGUV**	Iguape virus strain SPAn 71686		AY632538	10922	Unknown	-N/A-
